# Bioorthogonal Gold-Catalyzed Hydrothiolation Leading to Amide Bond Cleavage of Ethynylated Biarylbutanamide Precursors

**DOI:** 10.1021/jacs.6c06841

**Published:** 2026-06-10

**Authors:** Jing Huang, Yufei Li, Jianghui Du, Yiling Liu, Kenward Vong

**Affiliations:** Department of Chemistry, The Hong Kong University of Science and Technology, Clear Water Bay, Kowloon, Hong Kong 999077, China

## Abstract

Dissociative bioorthogonal metal-catalyzed reactions have steadily been applied in various *in vivo* applications, particularly with the development of prodrug strategies. This work introduces the ethynylated biarylbutanamide (EBB) group, which was designed to undergo gold-catalyzed hydrothiolation, cyclization, and hydrolysis to release amines under mild physiological conditions. To highlight its application for anticancer prodrug therapies, this study then utilized lectin-directed artificial metalloenzymes for the gold-catalyzed activation of an EBB-masked prodrug against targeted hypersialylated cancer cells.

## Graphical abstract

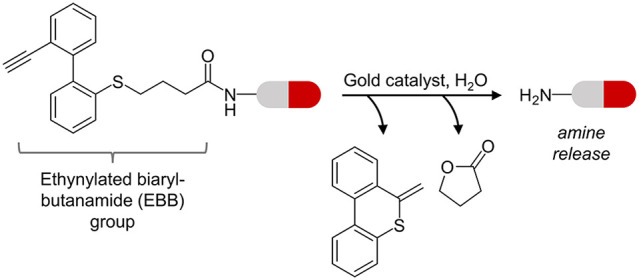

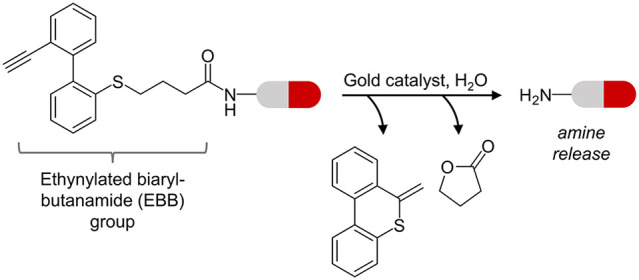

## Introduction

Bioorthogonal chemistry can be described as chemical transformations that are performed within living organisms that have no biological equivalent.^1^ As a result, these reactions are able to proceed without interference from other classes of biomolecules,^2^ thereby enhancing their *in vivo* selectivity and lowering the risk of provoking unintended background reactions. Among these reactions, ligation reactions (bond-forming) have gained the most popularity and have been used extensively in applications such as cell and protein labeling.^3,4^ In parallel, there has also been rising interest in the development of dissociative reactions (bond-breaking),^5–7^ which has its most apparent utility as mechanisms for prodrug activation.

To develop these bioorthogonal reactions, abiotic transition metal catalysts have been extensively investigated and tested *in vitro*/*in vivo*.^8–12^ With regard to dissociative reactions, most examples have been adapted for the metal-triggered uncaging of prodrug molecules to release bioactive drugs. For example, the release of amine- and alcohol-containing molecules can be achieved via depropargylation,^13–44^ deallylation,^43–58^ deallenylation,^59^ and cyclization involving diolefinic carbamates.^60^ In addition, a recent breakthrough has even seen drug release via C–C bond cleavage.^61^ In general, all these examples have relied on abiotic metal triggers such as palladium,^16–47,52,53,55,59,61^ ruthenium,^44,48–51,54,56–58,60^ platinum,^62^ or gold.^13–15,63^

The principal aim of this study is to develop an alternative metal-catalyzed dissociative bioorthogonal reaction that can be used for prodrug activation with the recently developed multivalent lectin-directed artificial metalloenzymes (ArM) (Figure 1A).^64^ At present, reactions that can cleave amide bonds to release amine-containing drugs have thus far been limited to the usage of 2-alkynylbenzamides,^63^ pentynoyl amides,^62^ and pentenoic amides.^65^ However, these reactions are known to suffer from poor catalytic activity; 2-alkynylbenzamides and pentenoic amides are not described as catalytic, while pentynoyl amides possess slow kinetics (turnover of 3.3 over 72 h). As a result, there is a demand to create alternative dissociative bioorthogonal reactions that can cleave amide bonds in a catalytic manner.

**Figure 1:**
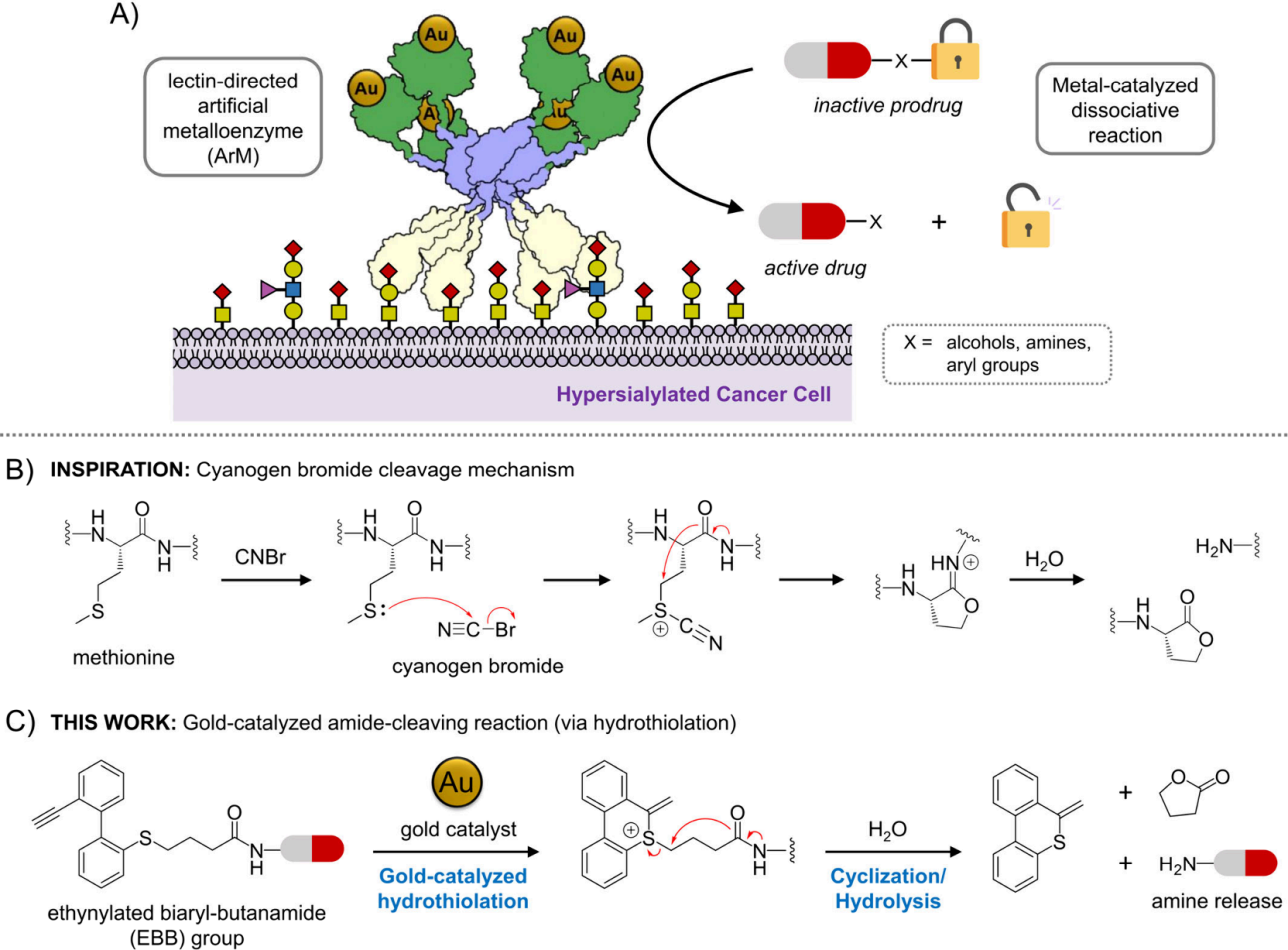
(A) Artificial metalloenzyme (ArM) prodrug therapies require two major aspects: a cancer-targeting ArM and a metal-catalyzed dissociative bioorthogonal reaction that can be used to release bioactive drugs from a prodrug precursor. Multivalent lectin-directed artificial metalloenzymes embedded with a gold­(III) catalyst has previously been developed to target hypersialylated cancer cells. (B) In the mechanism of cyanogen bromide-induced protein cleavage, charged sulfonium intermediates at methionine residues are produced. Subsequent carbonyl oxygen attack is facilitated, leading to cyclization and hydrolysis that results in polypeptide cleavage. (C) The EBB group was developed so that a charged sulfonium intermediate could be produced following gold-catalyzed hydrothiolation. With a γ-positioned amide group, cyclization via carbonyl oxygen attack should then be possible, leading to amide bond cleavage following hydrolysis. EBB chemistry should be amenable for creating gold-responsive prodrugs that mask amine-containing bioactive molecules.

Cyanogen bromide has been known for decades as a site selective method for the cleavage of polypeptides at methionine residues. From a mechanistic standpoint (Figure 1B), cyanogen bromide first acts by nitrile functionalization of the thioether. This creates a sulfonium intermediate where the neighboring carbon becomes highly electrophilic, thereby allowing attack of the residue’s carbonyl oxygen to form a five-membered ring. Subsequent hydrolysis of the intermediate is then known to facilitate peptide backbone cleavage.

Interested in mimicking the positively charged sulfonium intermediate that is central to the proteolytic mechanism of cyanogen bromide, this study identified gold-catalyzed alkyne hydrothiolation as a suitable reaction. However, current literature examples of this reaction are limited and have only been carried out under dry organic solvents.^66,67^ Thus, a crucial first step in this work was to test the water compatibility of gold-catalyzed intramolecular alkyne hydrothiolation. Following the confirmation of this reaction, the ethynylated biarylbutanamide (EBB) group was designed. In the proposed mechanism (Figure 1C), gold catalysis is thought to promote intramolecular hydrothiolation, leading to a positively charged sulfonium intermediate. Given its reactivity, cyclization is expected to occur where the nearby carbonyl oxygen attacks the adjacent carbon of the sulfonium intermediate, thereby creating a 5-membered ring along with the expulsion of a dibenzothiopyran. Eventual hydrolysis should then elicit the release of the amine-containing portion of the molecule, effectively resulting in amide bond cleavage. With the establishment of EBB chemistry, the focus of this study then shifted toward creating a working example of an anticancer prodrug therapy using an EBB-protected doxorubicin prodrug.

## Results and Discussion

### Reaction Development and Catalyst Screening

During preliminary investigations, hydrothiolation catalyzed by transition metals first needed to be confirmed as water compatible. To do this, substrate **1** was synthesized according to Scheme S1 and then screened against a variety of transition metals (i.e., Au, Pt, Pd, Ag, Ru, and Cu) under aqueous conditions (Figure 2A, Table S2). Additionally, two different molar equivalents of metals were used to help identify any differences in reactivity. From these data, hydrothiolation leading to product **2** appeared to only proceed when exposed to a gold­(III) catalyst, which gave a turnover (TON) of about 2.5 at 40 mol % of catalyst. No other transition metal was observed to catalyze any significant levels of hydrothiolation under these conditions.

**Figure 2:**
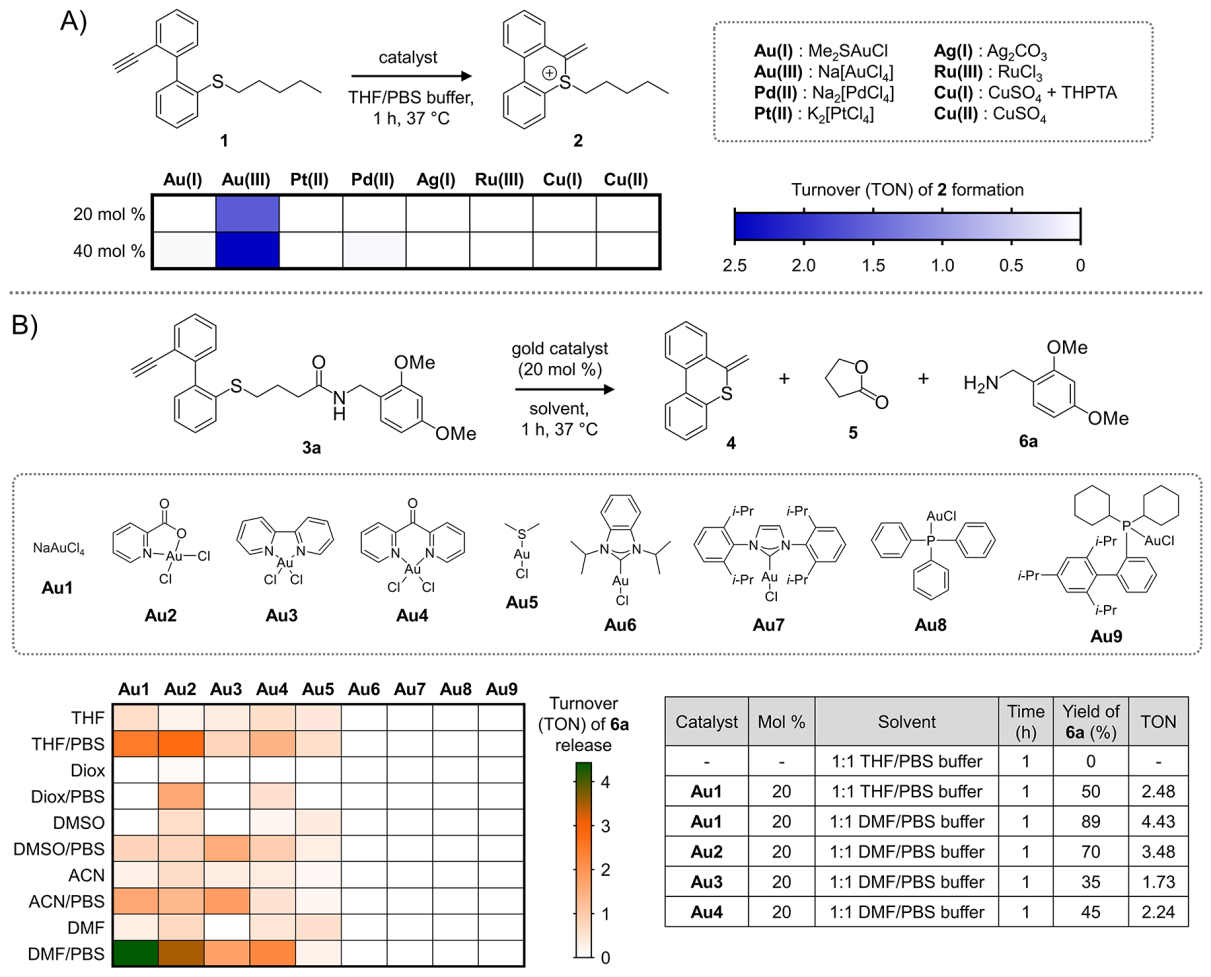
(A) Reactivity studies were carried out to screen intramolecular hydrothiolation of model substrate **1** under aqueous conditions. To identify an appropriate catalyst, both the molar equivalents and transition metal types were varied. (B) Reactivity studies conducted with EBB precursor **3a** was designed to test the gold-catalyzed release of amines using various gold catalysts (20 mol % of **Au1**–**Au9**). For all reactivity studies, product yields of formation/release were determined and then converted to turnover. A heat map summary is shown to highlight the TON numbers obtained from differing conditions. When using aqueous solvent mixtures, 1:1 solutions of organic solvent with PBS buffer pH 7.4 were chosen as a way to help promote substrate solubility.

Following confirmation of gold-catalyzed hydrothiolation, investigations were then made into the hypothesized amide-cleavage properties of the EBB group. Substrate **3a** was synthesized according to Scheme S2 and then screened against a variety of gold­(I) and gold­(III) catalysts under aqueous conditions (Figure 2B, Table S3). During preliminary testing, substrate **3a** was indeed revealed to undergo successful release of its corresponding amine **6a** when exposed to **Au1** in THF/PBS buffer conditions after 1 h. Furthermore, varying the percentage of gold (5–40%) was found to correlate well with reactivity (entries 18–21, Table S3). From these data, it was decided that further experiments would be conducted at 20 mol % of catalyst under a 1 h reaction time frame.

In the next step, a screen of compatible solvent systems and gold catalysts (**Au1**–**Au9**) was carried out, as summarized by the heat map in Figure 2B (details in Table S3). From these results, two major observations can be made. First, reactivity was overall much better with gold­(III) catalysts **Au1**–**Au4**, with TONs that averaged ∼1.8 under aqueous conditions (excluding dioxane-containing reactions). This contrasts with gold­(I) catalysts **Au5**–**Au9**, which consistently gave overall TONs lower than 0.62. Second, reactivity appears to be promoted by the presence of water. In all cases involving gold­(III) catalysts, TONs under aqueous conditions were significantly higher than TONs acquired in pure organic solvent (e.g., **Au1** gave TON of 4.43 in DMF/PBS buffer, but only TON of 0.3 in pure DMF). This observation can be simply explained by the fact that hydrolysis is a key step in the proposed reaction mechanism.

### Substrate Scope Investigation

To gain more insight into the reactivity of the EBB group, a number of modifications were introduced to probe changes in amine release yields (Figure 3, details in Tables S4–S11). One of the first alterations that were made focused on the types of tolerable substituents on the alkyne moiety. As a reference, the original EBB group of **3a** contained a terminal alkyne, which gave a TON value of 2.48. However, under similar conditions using a series of internal alkynes (**3b**–**d**), TON values were found to be lowered. For example, using a nonbulky methyl substituent (**3b**) was only found to slightly lower activity to a TON of 1.53. However, once more sterically bulky groups were used, such as a butyl (**3c**) or phenyl (**3d**) group, the TON values dropped to 0.11 and 0.24, respectively. A likely explanation for this effect is that bulkier alkyne substituents may hinder the formation of the hydrothiolation product, which is the key intermediate in the cascade leading to amine release.

**Figure 3:**
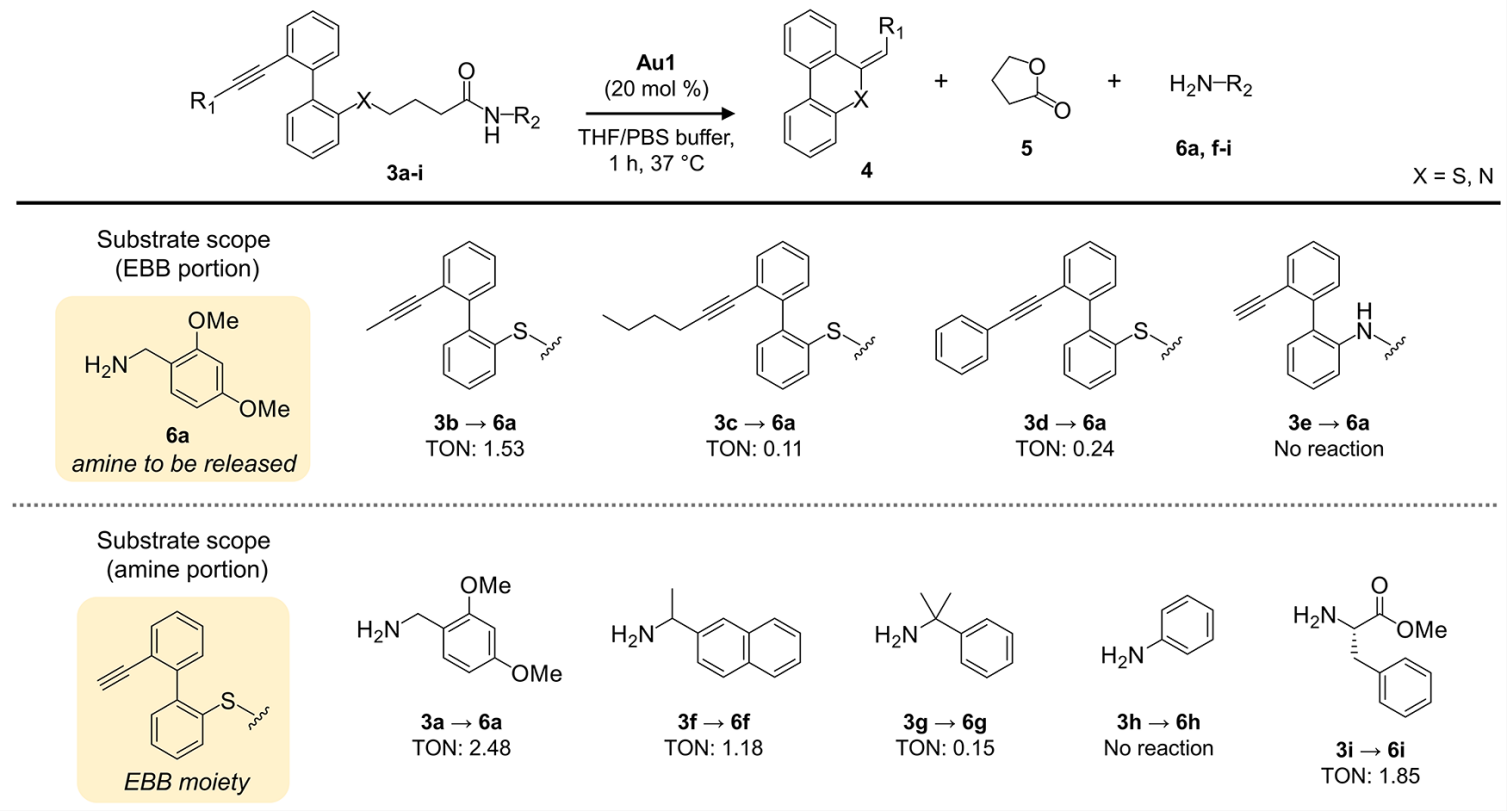
Substrate scope studies to probe the tolerability of the EBB group to different types of modifications, which include changes to the alkyne, thiol, and amine moieties. TON values were calculated from the release of corresponding amines when substrates **3a**–**i** were exposed to 20 mol % of **Au1** in THF/PBS buffer conditions for 1 h.

Another substrate modification that was explored centered on proving the necessity of the hydrothiolation product intermediate. In the proposed EBB mechanism, a charged sulfonium intermediate is needed to elicit cyclization via a carbonyl oxygen attack. Thus, the thiol should be considered essential in the structure of EBB. To confirm this, substrate **3e** was prepared, which replaced the thiol with an amine. Subsequent tests showed that **3e** could not induce an amine release, thereby confirming the mechanistic importance of the hydrothiolation step.

Finally, EBB-protected amines **3f**–**i** were prepared to compare their differential amine release activities. From these data, it can be observed that the highest release yields occurred for amines adjacent to primary carbons (TON of 2.48 for **3a**), followed by secondary carbons (TON of 1.18 for **3f**) and then tertiary carbons (TON of 0.15 for **3g**). An explanation for this effect likely stems from bulkier moieties sterically shielding the amide bond from nucleophilic attack, thereby lowering the rates of hydrolysis. Another observation is that the lone pair electrons on the nitrogen are needed to promote cyclization, as an EBB substrate meant for the release of aniline (**3h**) showed no observable reactivity. As a final check on the utility of the EBB group, the release of biomolecules such as glycine was confirmed by using substrate **3i**.

### Mechanistic Analysis

Since EBB was inspired by cyanogen bromide, the driving force behind the cyclization step in EBB chemistry is already well established. However, two important mechanistic considerations in EBB chemistry need to be discussed. First, there are two possible major rate-limiting steps, which is either hydrothiolation or cyclization/hydrolysis. To determine this, substrate **3j** was tested, which is an EBB analogue capable of hydrothiolation but incapable of amide bond cleavage. This property of **3j** exists due to the loss of a carbon in the linker moiety, thereby preventing cyclization since a highly disfavored 4-membered ring needs to be produced. Reactivity tests with **3j** produced a TON of 3.8 ± 0.3 for the hydrothiolation product (Figure 4A), which is similar to the TON obtained for the hydrothiolation/amide cleavage of **3a**. Thus, these data suggest that hydrothiolation is likely the rate-limiting step.

**Figure 4:**
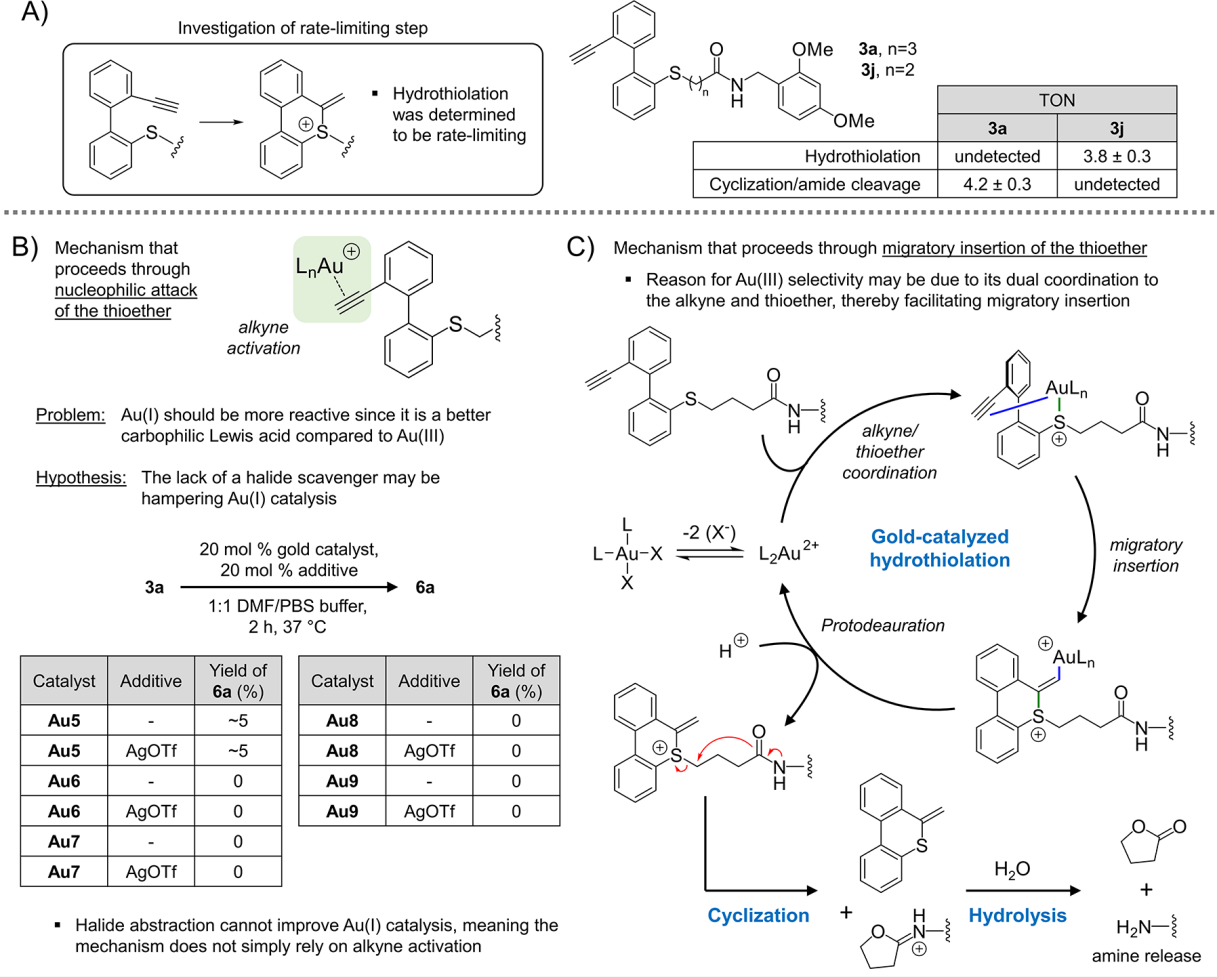
Mechanistic investigations into EBB chemistry. (A) To determine the rate-limiting step, analogue **3j** was prepared, which is unable to undergo the cyclization step leading to amide cleavage (due to the higher steric strain). The TON (for hydrothiolation) of **3j** was revealed to be similar to the TON (for amide cleavage) of **3a**. This suggests that hydrothiolation is likely the rate-limiting step for EBB chemistry. (B) One proposed mechanistic pathway in this study is that gold acts as a Lewis π-acid to activate the alkyne, thereby allowing nucleophilic attack by the thioether moiety to create the sulfonium intermediate. To validate this rationale, the reactivities of **Au5**–**Au9** were further tested in the presence of a halide scavenger. The absence of any reactivity in the presence of strong carbophilic Au­(I) catalysts suggests a mechanism that does not rely simply on alkyne activation. (C) Another proposed mechanistic pathway in this study is that gold acts to dually coordinate with both alkyne and thioether. Migratory insertion will then occur to generate the sulfonium intermediate by gold-catalyzed hydrothiolation. In this manner, tetradentate Au­(III) should have greater reactivity compared to bidentate Au­(I). Afterward, cyclization initiated by carbonyl oxygen attack by the γ-positioned amide group should then lead to a hydrolyzable intermedate that can release amine-containing molecules.

Another mechanistic consideration is the observation that Au­(III) catalysts were significantly more reactive than Au­(I) catalysts. To understand this selectivity, the mechanistic pathway of hydrothiolation needed to be uncovered. One possible mechanism to promote hydrothiolation is that gold acts as a Lewis π-acid to activate the alkyne (Figure 4B), followed by the nucleophilic attack of the thioether to create the sulfonium intermediate. The problem with this rationale is that Au­(I) is a softer, more superior carbophilic Lewis acid for activating alkynes compared to Au­(III). A possible reason for the lack of Au­(I)-activated EBB decaging may be due to the absence of a sufficient halide scavenger. Although literature reports have identified that water can fulfill this role for Au­(I) catalysts under aqueous conditions,^58^ it was theorized that the addition of a silver halide scavenger (i.e., AgOTf) could possibly improve reactivity. Unfortunately, EBB-decaging experiments carried out using **Au5**–**Au9** could not be restored with AgOTf addition. As a result, this data puts into question whether alkyne activation by a lewis acid alone can properly facilitate hydrothiolation.

In the literature, there have been increasing instances of gold-catalyzed reactions that operate through migratory insertion of unsaturated C–C bonds.^68,69^ Thus, the most feasible mechanism for the hydrothiolation step in EBB chemistry is that gold first coordinates with both the alkyne and thioether, followed by migratory insertion (Figure 4C). In this manner, the selectivity of the Au­(III) catalysts used in this study can be explained due to their capacity to lose two chlorine anions to coordinate both the alkyne and thioether. Conversely, Au­(I) catalysts ligated with strong σ-donors (i.e., phosphines, NHC) are unlikely to promote the dual coordination needed for reactivity.

### Kinetic Studies

In the next stage of this study, an investigation was launched to evaluate the reaction kinetics of EBB chemistry. To perform these experiments, the naphthalimide-based photoinduced electron transfer (PET) probe **7** was modified with an EBB group to give profluorophore **8a** (Figure 5A). In this manner, amide bond cleavage can be charted through real-time fluorescent monitoring of **7** at an excitation and emission wavelength of 390 and 530 nm, respectively (Figure 5B). Additionally, profluorophores derived from other bioorthogonal gold-triggered reactions were prepared as comparative controls. This includes compound **8b**, which relies on pentenoic amide chemistry for activation,^65^ as well as compound **8c**, which is protected with a 2-alkynylbenzamide group.^63^ All profluorophores were prepared according to Scheme S6. The nonfluorescent nature of profluorophores **8a**–**c** was further confirmed by determination of their quantum yields (Figure 5C, Figures S8 and S9), which was found to have values 7.3- to 18.6-fold lower than the quantum yield of known fluorophore **7**.

**Figure 5:**
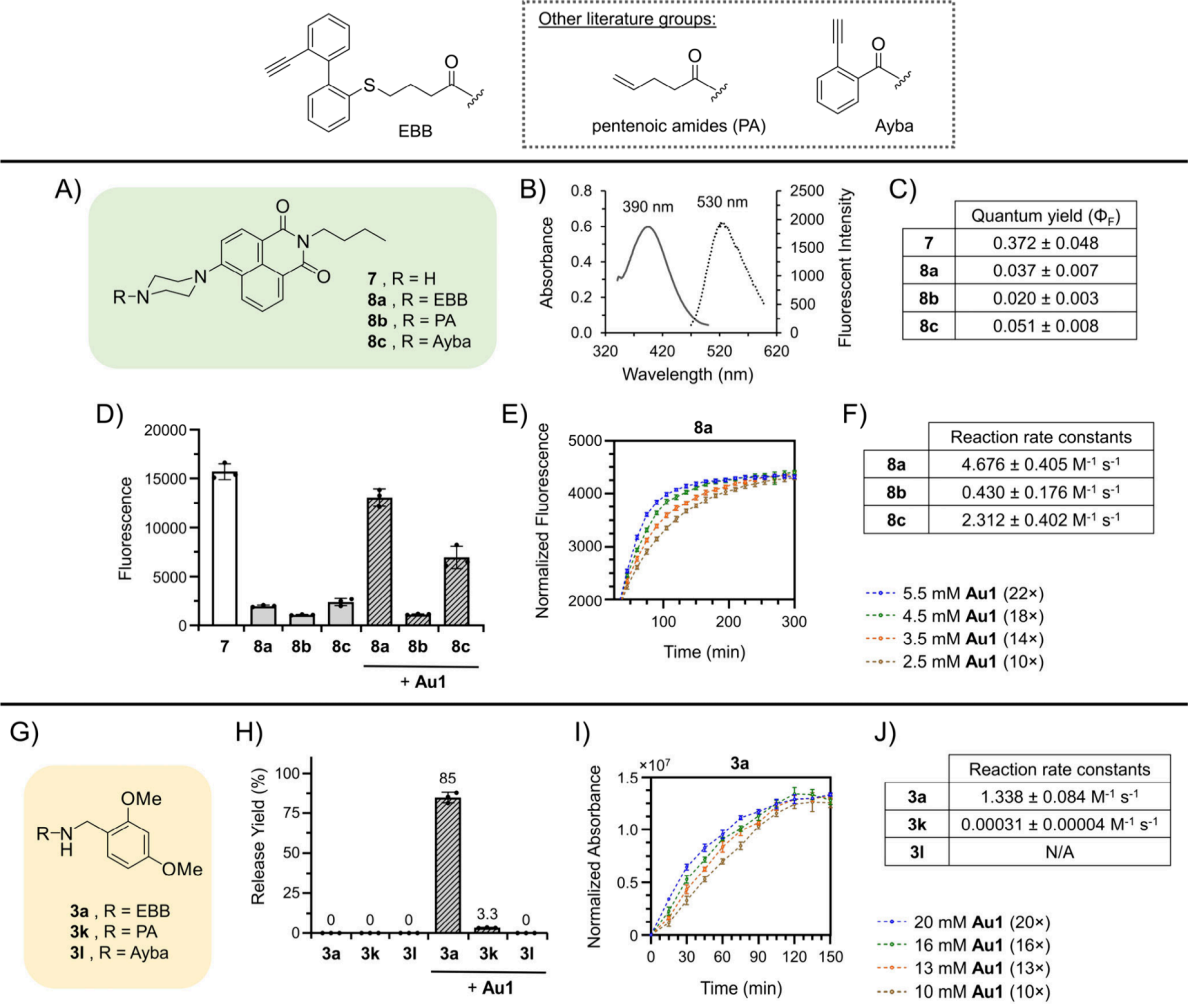
Comparative studies to determine the reactivity of the EBB group compared to other literature amide-cleaving groups (i.e., pentenoic amides and Ayba). (A) Profluorophores **8a**–**c** were designed to undergo gold-catalyzed cleavage to release the secondary amine-containing fluorophore **7**. (B) Absorption and emission spectra of fluorophore **7** were collected in 50% DMF/H_2_O (λ_EX_ = 390 nm, λ_EM_ = 530 nm). (C) Table of quantum yield values obtained for **8a**–**c** and **7**. (D) To gauge general reactivity, profluorophores **8a**–**c** (100 μM) were exposed to **Au1** (100 μM) in 50% DMF/H_2_O. Fluorescent readings were then taken after 1 h of incubation at room temperature. (E) Averaged curves obtained during the time-dependent fluorescent monitoring of **8a** (250 μM) incubated with varying concentrations of **Au1** in 50% DMF/H_2_O at room temperature. (F) Table of reaction rate constants for the profluorophores **8a**–**c**. (G) Substrates **3a**,**k**,**l** were all designed to under gold-catalyzed cleavage to release the primary amine **6a**. (H) To gauge general reactivity, substrates **3a**,**k**,**l** (1 mM) were exposed to **Au1** (10 mM) in 50% DMF/H_2_O. Product yields were determined by HPLC analysis after 2 h of incubation at 37 °C. (I) Averaged curves obtained during time-dependent HPLC analysis of **3a** (1 mM) incubated with varying concentrations of **Au1** in 50% DMF/H_2_O at room temperature. (J) Table of reaction rate constants for substrates **3a**,**k**,**l**.

To test their comparative reactivities, gold catalyst **Au1** was added to solutions containing profluorophores **8a**–**c** (Figure 5D). After a 1 h reaction time frame at room temperature, EBB-protected **8a** could be activated to achieve a near maximal fluorescent signal (compared to **7**). On the other hand, **8b** and **8c** produced much lower levels of fluorescence.

To acquire more reactivity data, profluorophore **8a** was monitored under varying concentrations of **Au1** at room temperature (Figure 5E). From these measurements (Figure 5F, Figures S10–S12), the second-order rate constant of **8a** unmasking was calculated to be ∼4.676 M^–1^ s^–1^. Again, these results attest to the higher reactivity of EBB chemistry compared to literature controls, which could only give lower second-order rate constants for **8b** (∼0.430 M^–1^ s^–1^) and **8c** (∼2.312 M^–1^ s^–1^).

Since profluorophores **8a**–**c** can only monitor the gold-catalyzed release of secondary amines, an additional study was carried out using substrates **3a**, **3k**, and **3l** to measure rates of primary amine release (Figure 5G). As a preliminary comparative test, mixtures of **3a**, **3k**, or **3l** were incubated with gold catalyst **Au1** for 2 h at 37 °C (Figure 5H). This experiment yet again showed that EBB cleavage could proceed the fastest, as the yield for primary amine release from **3a** was over 25-fold greater than the yields acquired from **3k** and **3l**.

As a final evaluation, the time-dependent cleavage of EBB from substrate **3a** was monitored under varying concentrations of **Au1** (Figure 5I). From these measurements (Figure 5J, Figures S13–S15), the second-order rate constant of **3a** unmasking was calculated to be ∼1.338 M^–1^ s^–1^. This rate is again higher than the values acquired for **3k** (∼0.000 31 M^–1^ s^–1^) and **3l** (no reaction). Overall, these kinetic investigations prove that EBB chemistry for the gold-catalyzed release of primary and secondary amines under aqueous conditions is the fastest among literature examples.

### EBB Reactivity with Gold-Based ArMs

Up until now, reactivity tests have been conducted with high percentages of organic solvent, which is attributed to the hydrophobic nature of the gold catalysts under investigation. To properly test EBB chemistry under more biological conditions, the next set of experiments employed a gold-based artificial metalloenzyme (ArM). In the field of ArM development, one approach has been to install an abiotic metal catalyst into a protein scaffold so that the metal’s catalytic ability could be either improved stereoselectively, or shuttle it for localized activity. Some examples of protein scaffolds used for the development of ArMs include streptavidin,^70–76^ LmrR,^77–83^ nitrobindin,^84–89^ and the HaloTag protein.^90,91^ To conduct further reactivity experiments in this study, a gold-bound HaloTag (**Ht-Au**) ArM was prepared. This was done by incubating a mutated HaloTag protein with a bromoalkane-containing **Au10** (Figure 6A). Upon reacting with Asp106, the gold moiety of **Au10** will be covalently embedded inside the protein with minimal solvent exposure, as predicted by docking calculations (Figure 6B, Figure S16). To confirm catalyst loading, prepared ArMs were submitted to ICP-MS analysis, which found an approximate Au occupancy level of 87%.

**Figure 6:**
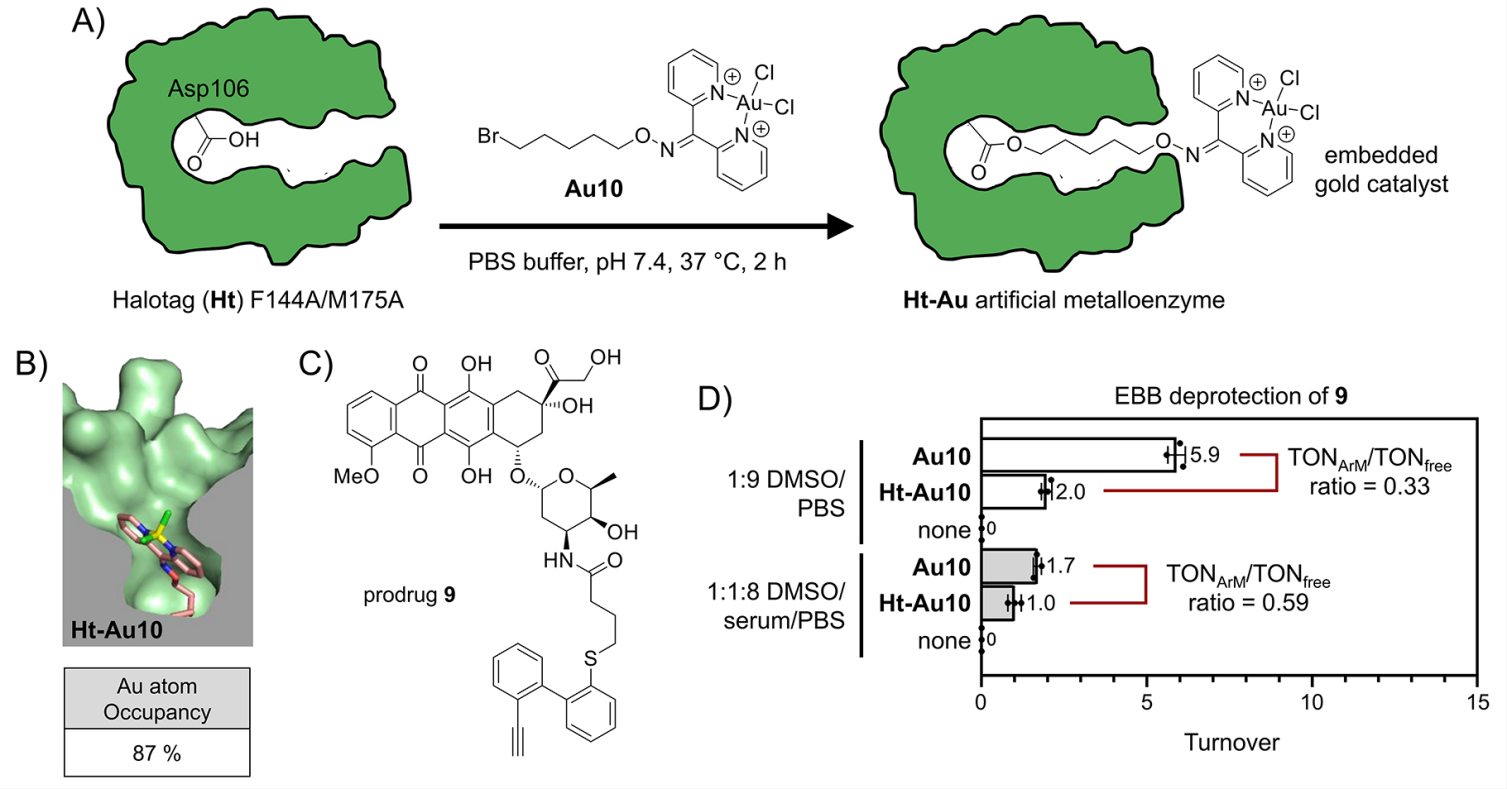
EBB reactivity studies using gold-embedded Halotag ArMs. (A) Catalyst **Au10** contains a chloroalkane moiety, which allows it to be covalently linked to a HaloTag (**Ht**) protein after a 2 h incubation at 37 °C. The resultant **Ht-Au10** ArM was used to test the reactivity of EBB under more biologically relevant conditions. (B) Once anchored to Asp106, the ligated gold moiety of **Au10** is expected to position itself in the **Ht** binding pocket as predicted by covalent docking studies. The occupancy of gold in **Ht-Au10** is expected to be about 87%, as quantitatively determined by ICP-MS. (C) Structure of prodrug **9** (EBB-protected doxorubicin) used for this study. (D) Turnovers for EBB deprotection of substrate **9** were acquired using the **Ht-Au10** ArM (or related controls) after 12 h incubations using 1 mol % of catalyst in relevant biological conditions.

To conduct activity tests with the prepared **Ht-Au10**, EBB-protected doxorubicin **9** was used (Figure 6C). During preliminary investigations (Figure 6D), EBB removal using **Ht-Au10** under typical buffered conditions (10% DMSO in PBS buffer pH 7.4) was found to proceed with a TON of ∼2.0. Since a TON of ∼5.9 was acquired under similar conditions using the free metal catalyst **Au10**, that would put the TON_ArM_/TON_free_ ratio at about 0.33. This result suggests that the embedded metal in the HaloTag protein is being slightly deactivated or suffers from impaired access to the available substrates in the media.

To mimic more biologically relevant conditions, reactions were run under similar conditions supplemented with serum. In the case of the free metal catalyst **Au10**, the TON dropped to about 1.7, which indicates that the serum components likely quench the gold catalyst. On the other hand, the **Ht-Au10** ArM showed only a moderate decline to a TON of ∼1.0, thus producing a TON_ArM_/TON_free_ ratio at a higher value of 0.59. This trend is similar to what was observed before,^64^ which can be interpreted that while the catalytic activity of the ArM is lower than the free metal catalyst, its protein scaffold may offer a moderate degree of protection to the embedded metal catalyst.

### Validation of EBB Prodrugs

Through a scan of the literature, one method to perform metal uncaging of prodrugs has been to utilize an ArM functionalized with cancer-targeting capabilities. This strategy has been employed for protein scaffolds such as albumin,^92–96^ Herceptin,^33,97^ and most recently with a Halotag-PduU-ACG lectin fusion protein (**HtPA**).^64^ To validate EBB chemistry with an anticancer ArM prodrug therapy, this study employed **HtPA-Au10**, which is a **HtPA** protein embedded with the gold­(III) catalyst **Au10** (Figure 7A). The targeting mechanism of **HtPA-Au10** is designed to undergo oligomerization to create hexameric protein complexes that benefit from lectin multivalency to bind hypersialylated cancer cells with a high affinity. Previously, **HtPA** ArMs were shown to selectively bind sialic acid-rich cell lines such as MDA-MB-231 (triple-negative breast cancer cells).^64^

**Figure 7:**
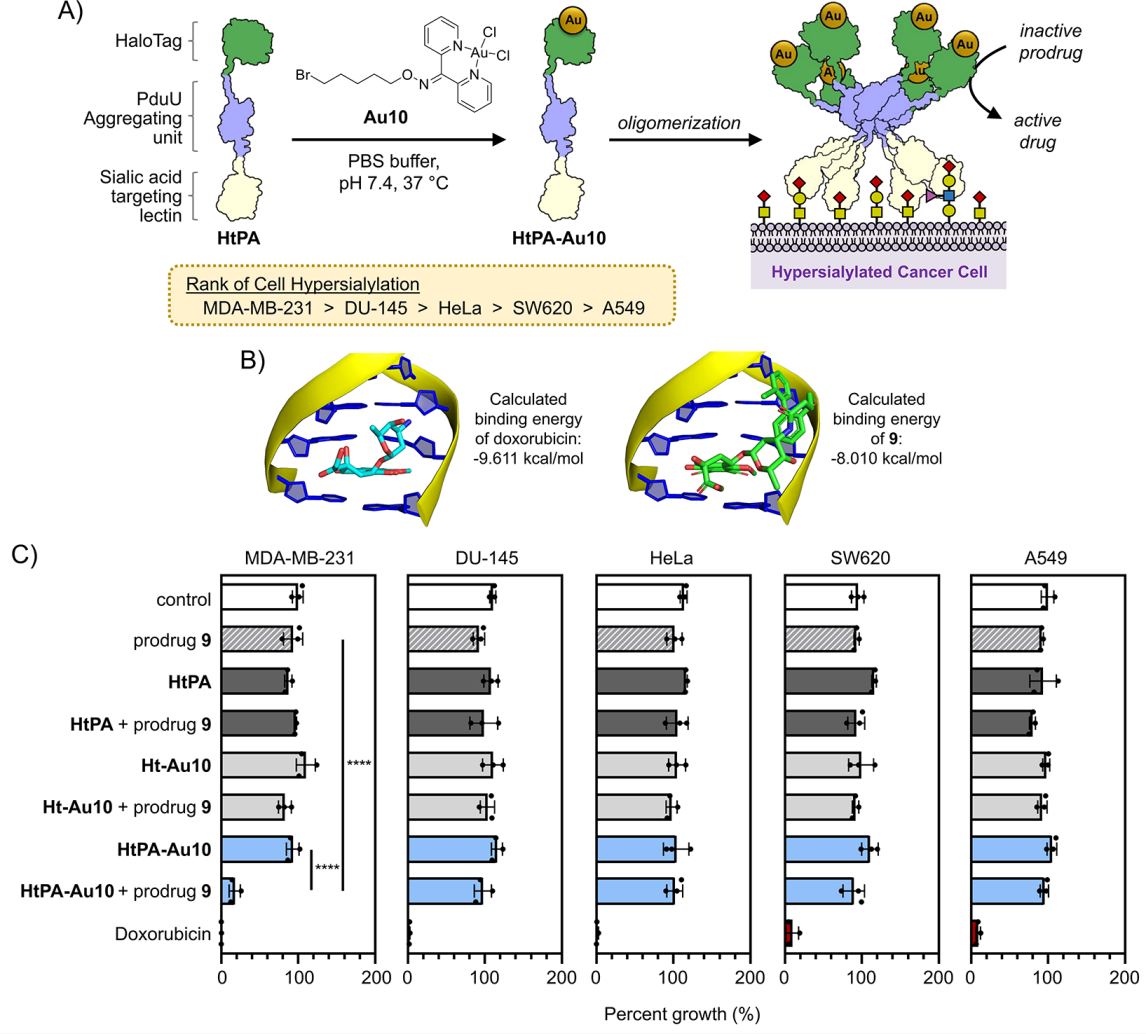
Cell assay investigations into the EBB-based prodrug system. (A) To target hypersialyated cancer cells, the lectin-directed halotag artificial metalloenzyme (**HtPA-Au10**) can be used. To prepare this ArM, **HtPA** was incubated with the gold catalyst **Au10** in PBS buffer for 2 h at 37 °C. (B) Modeling studies were performed to probe differences in calculated binding energies for prodrug **9** and doxorubicin when intercalated within double stranded DNA (sourced from PDB 1D12). (C) Cell viability experiments were done to test the ArM prodrug therapy against different cancer cell lies (i.e., MDA-MB-231, DU-145, SW620, HeLa, and A549). In these experiments, cells were incubated with varying mixtures of prodrug **9** (10 μM) with either **HtPA** (1 μM), **Ht-Au10** (1 μM), or **HtPA-Au10** (1 μM).

Moving onto the final stage of this study, cell viability assays were performed using the EBB-protected doxorubicin prodrug **9**. Doxorubicin is a widely known anticancer agent that mainly exerts its anticancer activities through DNA intercalation. Numerous reports have shown that the NH_2_ group at the C3′ position of the daunosamine moiety is critical for cytotoxicity, as analogues that cage the free amine consistently display lower DNA binding affinities.^98^

As a preliminary test to probe whether prodrug **9** would have weaker DNA binding, modeling studies were carried out (Figure 7B, Figure S17). Docking with prodrug **9** produced a configuration with a calculated binding affinity of −8.010 kcal/mol. This value indicates lower binding compared with a similar pose generated through the docking of doxorubicin (−9.611 kcal/mol), which served as a control. In addition, prodrug **9** is modeled to have only partial DNA intercalation within double-stranded DNA helices (Figure S17D), likely due to its increased steric bulk. As such, it is expected that prodrug **9** will exhibit lower levels of cytotoxicity because of poorer DNA intercalation.

Using MDA-MB-231 cells as the targeted cell system, the designed anticancer ArM prodrug therapy was next tested in both cell-based assays. Starting with preliminary cell viability studies (Figure S19A), the IC_50_ for prodrug **9** was measured to be about 41 μM, which represented a 1950-fold decrease in activity compared to the IC_50_ of doxorubicin (21 nM). In addition, **HtPA-Au10** (1 μM) alone did not show any cytotoxic effects (Figure S19B).

To probe the efficacy of the ArM prodrug therapy, the viability of various cancer cell lines was next tested with different treatment conditions (Figure 7C). Not only was prodrug **9** treated with the targeting **HtPA-Au10** ArM, but control experiments were also run with the nontargeting ArM (**Ht-Au10**) and a gold-deficient ArM (**HtPA**). For the hypersialylated MDA-MB-231 cell line, a clear reduction in cell viability (∼82% decrease) was observed with the **HtPA-Au10**/prodrug **9** mixture. This contrasts with the control mixtures, which produced no reduction of cell viability. All relevant controls (**HtPA** only, **Ht-Au10** only, **HtPA-Au10** only, and prodrug **9** only) also did not produce any cytotoxic effects. Additionally, no cell death was induced in other cell lines with lower levels of sialylation (DU-145, HeLa, SW620, A549).

## Conclusions

This study successfully developed the EBB group, which was found to undergo gold-catalyzed hydrothiolation, cyclization, and hydrolysis to release amines under mild physiological conditions. Inspired by the proteolytic activity of cyanogen bromide, the unique mechanism of amide-bond cleavage in EBB is likely what allows this reaction to proceed catalytically using gold­(III) catalysts. In addition, the bioorthogonality of gold-catalyzed hydrothiolation was also proven in this study, which was never previously reported. Overall, we believe that this work will be a useful addition to the growing library of new-to-nature reactions. Not only does EBB chemistry have applicability in the development of prodrug therapies (as displayed in this study), but its chemoselectivity and amide-bond breaking properties could also be potentially adapted into chemical biology tools used to create new methods to explore and manipulate living biological systems. Future work will also look to adopt EBB chemistry in a deeper investigation of its *in vivo* efficacy by testing related prodrugs in animal models.

## Supplementary Material



## Notes

Detailed materials and methods, compound synthesis and characterization, ^1^H and ^13^C NMR spectra, experimental procedures for biological studies, and supplementary figures, tables, and schemes, including Figure S1–S19, Table S1–S12, and Scheme S1–S8 (PDF)
