# Supplementary material for: Bioorthogonal Gold-Catalyzed Hydrothiolation Leading to Amide Bond Cleavage of Ethynylated Biarylbutanamide Precursors
Source: J Am Chem Soc. 2026 Jun 10;148(24):25152–62. doi: 10.1021/jacs.6c06841 (PMC13307356; doi:10.1021/jacs.6c06841)
Supplement: Supplementary file 1 [file ja6c06841_si_001.pdf]

Supporting Information

Bioorthogonal gold-catalyzed hydrothiolation leading to amide bond cleavage of ethynylated biarylbutanamide precursors

Jing Huang,[1‡] Yufei Li,[1‡] Jianghui Du,[1] Yiling Liu,[1] Kenward Vong[1*]

1 Department of Chemistry, The Hong Kong University of Science and Technology, Clear Water Bay, Kowloon, Hong Kong, China

Table of Contents

1. Synthetic Protocols 2
   1.1 General Information 2
   1.2 Preparation of Model Substrates and Prodrug....................2
   1.3 Preparation of Gold Catalysts 29
2. Reactivity Studies 31
   2.1 HPLC Methods and Standard Curves 31
   2.2 Hydrothiolation Substrate Screening 35
   2.3 EBB-based Model Substrate Screening 36
3. Photophysical Characterizations....................43
   3.1 Justification of Solvent Conditions 43
   3.2 Quantum Yield Determination....................45
4. Kinetic Analyses 47
   4.1 Methodology for Reaction Rate Constants 47
   4.2 Determination of Reaction Rate Constants 47
5. Modelling studies....................50
   5.1 General Information 50
   5.2 Covalent docking of gold catalyst to the HaloTag protein....................50
   5.3 Docking of doxorubicin-based prodrug to dsDNA 51
6. Protein preparations and experiments....................52
   6.1 General Information 52
   6.2 Recombinant protein expression and purification....................52
   6.3 Artificial metalloenzyme preparation....................53
   6.4 Artificial metalloenzyme characterization 53
   6.5 Reactivity studies using artificial metalloenzymes 54
7. Cell-based Assays 55
   7.1 General Cell Culture....................55
   7.2 Statistical analysis 55
   7.3 Cell Cytotoxicity Studies 55
8. NMR spectra 57
9. References....................104

1. Synthetic Protocols

1.1 General Information

All chemicals were purchased from Sigma-Aldrich, TCI chemicals, JK Chemicals, or Macklin without further purification. TLC analysis (F-254) was performed with 60 Å silica gel from Merck Millipore. ^{1}H and ^{13}C NMR spectra were measured on a Bruker AVIII-400 spectrometer (400 MHz) with the solvent peaks as internal standards. High-resolution mass spectroscopy (HRMS) measurements were carried out at the Hong Kong University of Science and Technology Mass Spectrometry Service Center on either an Agilent GC/MS 5975C system or an API QSTAR XL System.

1.2 Preparation of Model Substrates and Prodrug

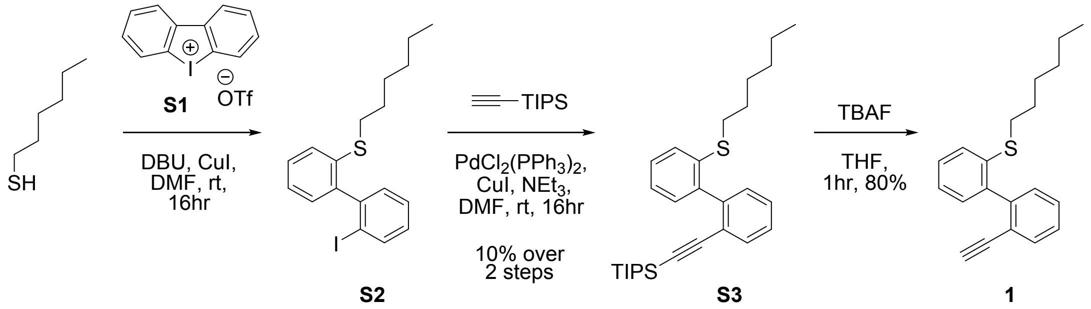

Scheme S1. Synthesis of model substrate **1**.

Preparation of **S1**

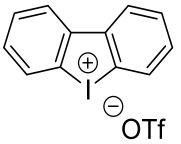

Synthesis followed literature protocols.[1] Meta-chloroperoxybenzoic acid (3.013g, 17.5 mmol) was dissolved in DCM (30 ml). 2-Iodobiphenyl (1.92ml, 11.1 mmol) was added dropwisely and the mixture was stirred in ice bath for 5 min. trifluoromethanesulfonic acid (5g, 33.3 mmol) was added dropwisely and the mixture was stirred in ice bath for 5 min, and then stirred at room temperature for 2 hr. To work up, the mixture was evaporated in vacuum and then added cold diethyl ether (20 ml) and stirred at room temperature for 20 min. The mixture was filtered to obtain fine gray powder. Yield: 5.305g, 71%. ^{1}H NMR (400 MHz, Methanol-d_4) δ 8.40 (d, J = 9.3 Hz, 2H), 8.15 (d, J = 8.3 Hz, 2H), 7.88 (t, J = 8.1 Hz, 2H), 7.77 – 7.67 (m, 2H); ^{13}C NMR (101 MHz, MeOD) δ 142.35, 131.06, 130.95, 130.19, 126.99, 120.06. HRMS for $C_{12}H_8I$ $[M]^+$ calcd. 278.9665, found 278.9669.

Preparation of **S3**

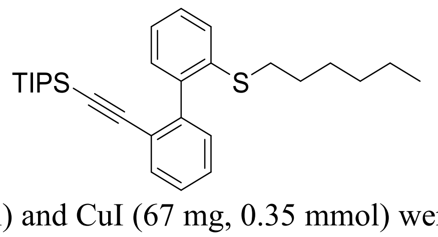

1-Hexanethiol (0.5 mL, 3.52 mmol) and CuI (67 mg, 0.35 mmol) were added to a solution of compound **S1** (2.03 g, 3.52 mmol) in anhydrous DMF (10 mL) under argon. Then DBU (1.0 mL, 7.04 mmol) was added dropwise at room temp. After overnight stirring, the reaction mixture was diluted with H_2O (30 mL) and extracted with EtOAc (20 mL). The organic layer was wash with brine, dried over Na_2SO_4 and concentrated under vacuum. Flash column chromatography (0-5% EtOAc/Hex) was used to isolate compound **S2** as a crude mixture. To carry out the next step, **S2** (420 mg, 1.06 mmol), CuI (20 mg, 0.10 mmol), and $PdCl_2(PPh_3)_2$ (74 mg, 0.10 mmol) were dissolved in anhydrous DMF (4 ml) and NEt_3 (2 ml) under argon. (Triisopropylsilyl)acetylene (0.48 ml, 2.12 mmol) was then added dropwise. After stirring

overnight at room temp. overnight, the reaction mixture was diluted with EtOAc, which was then washed with H_2O and brine. The organic layer was dried over Na_2SO_4 and concentrated under vacuum. Flash column chromatography (100% Hexane) was used to purify the desired compound **S3** (157 mg, 33%). ^{1}H NMR ($CDCl_3$, 400 MHz): δ 7.58 (dd, *J* = 7.4, 1.7 Hz, 1H), 7.43 – 7.28 (m, 5H), 7.25 – 7.22 (m, 1H), 7.17 (td, *J* = 7.4, 1.3 Hz, 1H), 2.69 (td, *J* = 7.3, 2.4 Hz, 2H), 1.50 (p, *J* = 7.2 Hz, 2H), 1.33 – 1.19 (m, 6H), 0.92 (s, 21H), 0.85 (t, *J* = 6.9 Hz, 3H). ^{13}C NMR ($CDCl_3$, 101 MHz): δ 143.85, 141.72, 136.24, 132.77, 130.62, 130.09, 128.77, 128.05, 127.90, 127.40, 125.41, 123.44, 105.82, 93.95, 33.88, 31.50, 29.03, 28.74, 22.69, 18.65, 14.17, 11.31. HRMS for $C_{29}H_{42}NaSSi$ $[M+Na]^+$ calcd. 473.2674, found 473.2678.

Preparation of **1**

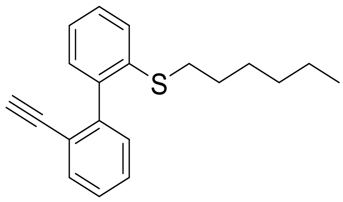

Compound **S3** (157 mg, 0.35 mmol) was dissolved in anhydrous THF (1 ml). TBAF (0.87 mL of 1.0 M solution in THF, 0.87 mmol) was then added and the mixture was stirred overnight at room temp under argon. The reaction was quenched with saturated NH_4Cl solution and extracted by EtOAc. The organic layer was then dried over Na_2SO_4 and concentrated under vacuum. Flash column chromatography (2% EtOAc/Hex) was used to purify the desired compound **1** (82 mg, 80%). ^{1}H NMR ($CDCl_3$, 400 MHz): δ 7.61 (dd, *J* = 7.6, 1.5 Hz, 1H), 7.46 – 7.29 (m, 5H), 7.25 – 7.22 (m, 2H), 2.93 (s, 1H), 2.74 (td, *J* = 7.2, 1.6 Hz, 2H), 1.54 – 1.48 (m, 2H), 1.34 – 1.20 (m, 6H), 0.85 (t, *J* = 6.9 Hz, 3H). ^{13}C NMR ($CDCl_3$, 101 MHz): δ 143.90, 141.30, 136.26, 133.06, 130.47, 130.23, 128.76, 128.47, 128.27, 127.53, 125.37, 121.99, 82.71, 80.22, 33.86, 31.48, 28.97, 28.69, 22.65, 14.16. HRMS for $C_{20}H_{22}NaS$ $[M+Na]^+$ calcd. 317.1340, found 317.1339.

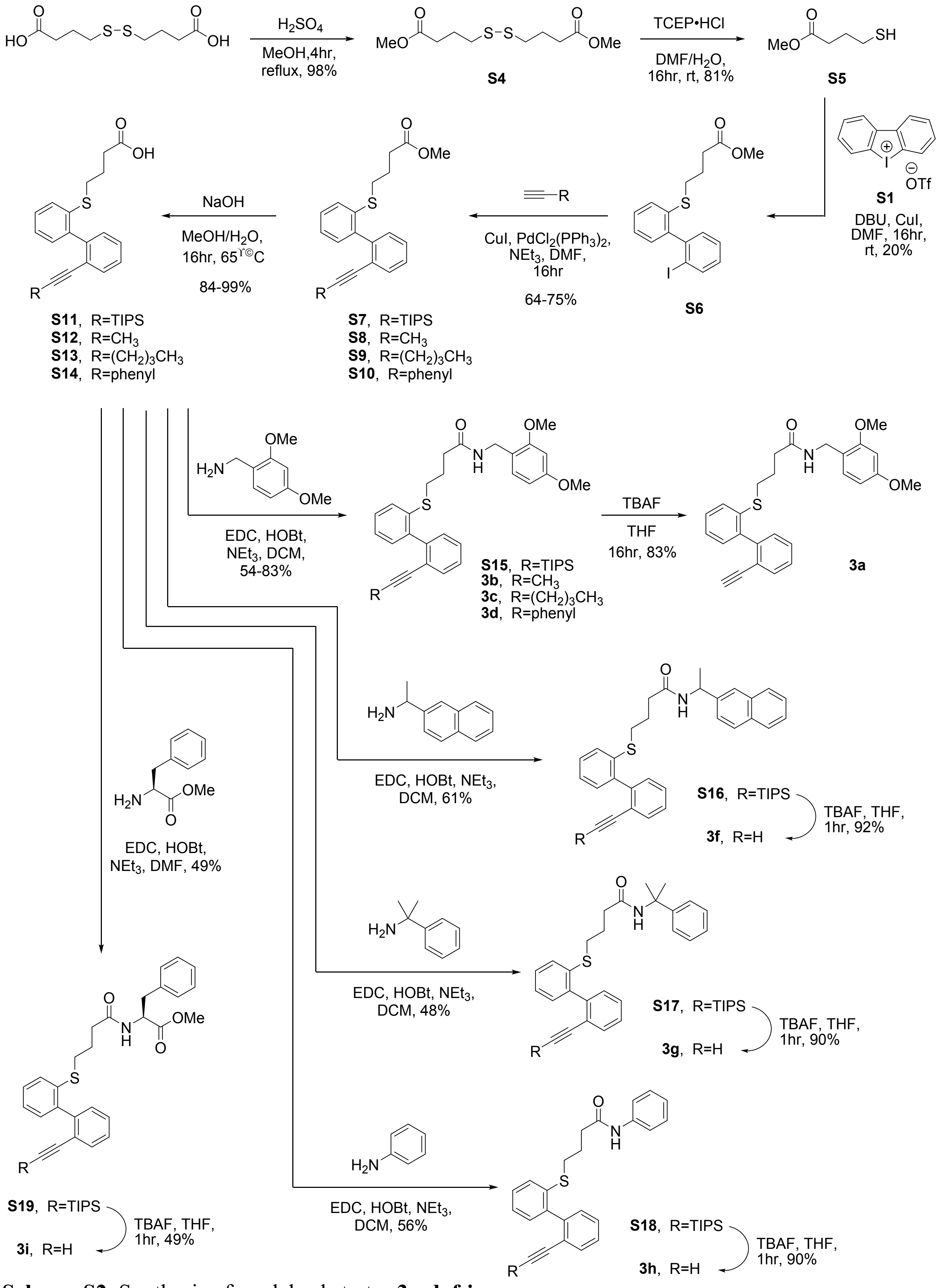

Scheme S2. Synthesis of model substrates **3a-d**, **f-i**.

Preparation of **S4**

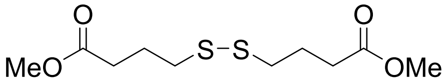

Sulfuric acid (1 mL) was added to a solution of 4, 4'-dithiodibutyric acid (2.00 g, 8.39 mmol) in MeOH (15 mL). After refluxing for 4 h, the methanol was evaporated under reduced pressure. The crude was diluted with EtOAc and washed with H_2O and brine. The organic phase was dried over Na_2SO_4 and then concentrated under vacuum to afford **S4** (2.20 g, 98%). ^{1}H NMR ($CDCl_3$, 400 MHz): δ 3.58 (s, 6H), 2.63 (t, *J* = 7.1 Hz, 4H), 2.36 (t, *J* = 7.3 Hz, 4H), 1.93 (p, *J* = 7.2 Hz, 4H). ^{13}C NMR ($CDCl_3$, 101 MHz): δ 173.14, 51.48, 37.62, 32.20, 24.05. HRMS for $C_9H_{14}NaO_4S_2$ $[M+Na]^+$ calcd. 289.0554, found 289.0551.

Preparation of **S5**

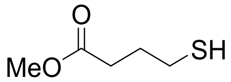

A solution of TCEP·HCl (4.73g, 16.52 mmol) in H_2O (10 mL) was added to compound **S4** in DMF (10 mL). After stirring overnight at room temp the reaction mixture was diluted with H_2O (50 mL) and extracted with EtOAc (30 mL). The organic layer was washed with brine and dried over Na_2SO_4. The solvent was removed under vacuum. Flash column chromatography (10% EtOAc/Hex) was used to purify the desired compound **S5** (1.80 g, 81%). ^{1}H NMR ($CDCl_3$, 400 MHz): δ 3.67 (s, 3H), 2.57 (dt, *J* = 8.1, 7.0 Hz, 2H), 2.46 (t, *J* = 7.3 Hz, 2H), 1.93 (p, *J* = 7.2 Hz, 2H), 1.34 (t, *J* = 8.1 Hz, 1H). ^{13}C NMR ($CDCl_3$, 101 MHz): δ 173.58, 51.79, 32.52, 29.12, 24.12. HRMS for $C_5H_{11}NaO_2S$ $[M+Na]^+$ calcd. 157.0299, found 157.0297.

Preparation of **S6**

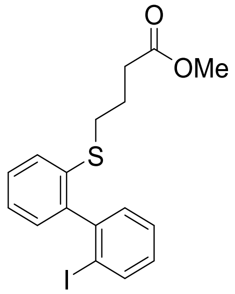

Compound **S5** (1.80 g, 13.41 mmol) and CuI (255 mg, 1.34 mmol) were added to a solution of compound **S1** (8.52 g, 14.75 mmol) in anhydrous DMF (10 mL) under argon. Then DBU (4.0 mL, 26.83 mmol) was added dropwise at room temp. After overnight stirring, the reaction mixture was diluted with H_2O (30 mL) and extracted with EtOAc (20 mL). The organic layer was wash with brine, dried over Na_2SO_4 and concentrated under vacuum. Flash column chromatography (5% EtOAc/Hex) was used to purify the desired compound **S6** (1.10 g, 20%). ^{1}H NMR ($CDCl_3$, 400 MHz): δ 7.94 (dd, *J* = 7.9, 1.2 Hz, 1H), 7.45 – 7.34 (m, 4H), 7.24 (dd, *J* = 2.9, 1.6 Hz, 1H), 7.14 – 7.04 (m, 2H), 3.65 (s, 3H), 2.85 (td, *J* = 7.1, 1.6 Hz, 2H), 2.40 (t, *J* = 7.3 Hz, 2H), 1.91 (p, *J* = 7.0 Hz, 2H). ^{13}C NMR ($CDCl_3$, 101 MHz): δ 173.51, 145.54, 144.77, 139.06, 135.39, 130.40, 130.27, 129.25, 128.68, 128.15, 128.02, 125.66, 100.36, 51.72, 32.82, 32.45, 24.18. HRMS for $C_{17}H_{17}INaO_2S$ $[M+Na]^+$ calcd. 434.9892, found 434.9890.

Preparation of **S7**

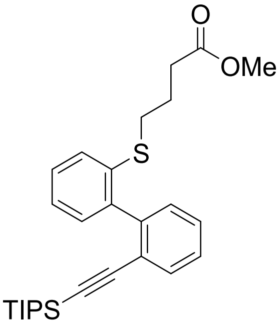

Compound **S6** (616 mg, 1.49 mmol), CuI (28 mg, 0.15 mmol), and $PdCl_2(PPh_3)_2$ (105 mg, 0.15 mmol) were dissolved in anhydrous DMF (3 mL) and NEt_3 (1 mL) under argon. (Triisopropylsilyl)acetylene (0.67 mL, 2.99 mmol) was then added dropwise. After stirring overnight at 60 °C overnight, the reaction

mixture was diluted with EtOAc, which was then washed with H_2O and brine. The organic layer was dried over Na_2SO_4 and concentrated under vacuum. Flash column chromatography (2-5% EtOAc/Hex) was used to purify the desired compound **S7** (510 mg, 73%). ^{1}H NMR ($CDCl_3$, 400 MHz): δ 7.62 – 7.54 (m, 1H), 7.41 (dd, *J* = 7.8, 1.3 Hz, 1H), 7.37 – 7.17 (m, 6H), 3.63 (s, 3H), 2.72 (q, *J* = 7.1 Hz, 2H), 2.34 – 2.26 (m, 2H), 1.81 (p, *J* = 7.2 Hz, 2H), 0.92 (s, 21H). ^{13}C NMR ($CDCl_3$, 101 MHz): δ 173.55, 143.74, 142.29, 135.20, 132.73, 130.72, 130.02, 129.59, 128.18, 127.92, 127.44, 125.94, 123.45, 105.74, 94.05, 51.63, 33.32, 32.69, 24.16, 18.62, 11.27. HRMS for $C_{28}H_{38}NaO_2SSi$ $[M+Na]^+$ calcd. 489.2259, found 489.2260.

Preparation of **S8**

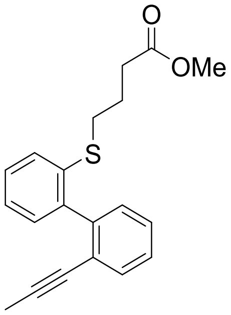

Compound **S6** (200 mg, 0.49 mmol), CuI (9 mg, 0.05 mmol), and $PdCl_2(PPh_3)_2$ (34 mg, 0.05 mmol) were dissolved in anhydrous DMF (3 mL) and NEt_3 (1 mL) under argon. Propyne (0.97 mL, 1.0 M solution in hexane, 0.97 mmol) was then added dropwise. After stirring overnight at r.t. overnight, the reaction mixture was diluted with EtOAc, which was then washed with H_2O and brine. The organic layer was dried over Na_2SO_4 and concentrated under vacuum. Flash column chromatography (5% EtOAc/Hex) was used to purify the desired compound **S8** (100 mg, 64%). ^{1}H NMR ($CDCl_3$, 400 MHz): δ 7.51 – 7.44 (m, 2H), 7.34 – 7.28 (m, 3H), 7.26 – 7.23 (m, 3H), 3.63 (s, 3H), 2.76 (td, *J* = 7.1, 1.4 Hz, 2H), 2.31 (t, *J* = 7.4 Hz, 2H), 1.86 – 1.76 (m, 5H). ^{13}C NMR ($CDCl_3$, 101 MHz): δ 173.65, 143.18, 142.69, 135.07, 132.31, 130.76, 130.05, 129.79, 128.10, 127.49, 127.22, 125.92, 123.90, 89.28, 78.85, 51.66, 33.38, 32.73, 24.24, 4.46. HRMS for $C_{22}H_{20}NaO_2S$ $[M+Na]^+$ calcd. 347.1082, found 347.1080.

Preparation of **S9**

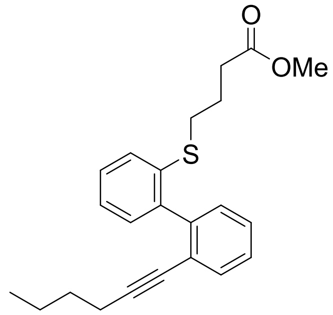

Compound **S6** (100 mg, 0.24 mmol), CuI (5 mg, 0.02 mmol), and $PdCl_2(PPh_3)_2$ (17 mg, 0.02 mmol) were dissolved in anhydrous DMF (2 mL) and NEt_3 (0.5 mL) under argon. 1-Hexyne (0.06 mL, 0.49 mmol) was then added dropwise. After stirring overnight at r.t. overnight, the reaction mixture was diluted with EtOAc, which was then washed with H_2O and brine. The organic layer was dried over Na_2SO_4 and concentrated under vacuum. Flash column chromatography (5% EtOAc/Hex) was used to purify the desired compound **S9** (67 mg, 75%). ^{1}H NMR ($CDCl_3$, 400 MHz): δ 7.51 – 7.40 (m, 2H), 7.34 – 7.27 (m, 3H), 7.27 – 7.22 (m, 3H), 3.63 (s, 3H), 2.75 (td, *J* = 7.0, 3.5 Hz, 2H), 2.31 (t, *J* = 7.3 Hz, 2H), 2.18 (t, *J* = 6.8 Hz, 2H), 1.81 (p, *J* = 7.4 Hz, 2H), 1.33 – 1.22 (m, 2H), 1.19 – 1.06 (m, 2H), 0.77 (t, *J* = 7.3 Hz, 3H). ^{13}C NMR ($CDCl_3$, 101 MHz): δ 173.64, 143.32, 142.72, 135.08, 131.99, 130.71, 129.87, 129.65, 128.02, 127.49, 127.17, 125.88, 123.96, 93.94, 79.77, 51.67, 33.31, 32.69, 30.55, 24.16, 21.63, 19.14, 13.77. HRMS for $C_{23}H_{26}NaO_2S$ $[M+Na]^+$ calcd. 389.1550, found 389.1551.

Preparation of **S10**

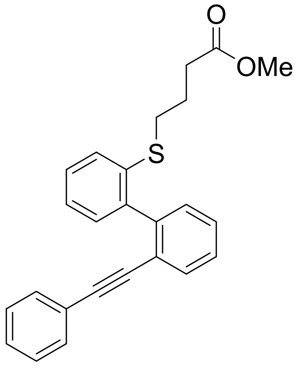

Compound **S6** (75 mg, 0.18 mmol), CuI (3 mg, 0.02 mmol), and $PdCl_2(PPh_3)_2$ (13 mg, 0.02 mmol) were dissolved in anhydrous DMF (2 mL) and NEt_3 (0.5 mL) under argon. Phenylacetylene (0.04 mL, 0.36 mmol) was then added dropwise. After stirring overnight at r.t. overnight, the reaction mixture was diluted with EtOAc, which was then washed with H_2O and brine. The organic layer was dried over Na_2SO_4 and concentrated under vacuum. Flash column chromatography (5% EtOAc/Hex) was used to purify the desired compound **S10** (49 mg, 70%). ^1H NMR ($CDCl_3$, 400 MHz): δ 7.65 – 7.61 (m, 1H), 7.51 (dd, J = 7.8, 1.4 Hz, 1H), 7.42 – 7.27 (m, 6H), 7.22 (dt, J = 4.9, 2.9 Hz, 3H), 7.17 – 7.11 (m, 2H), 3.56 (s, 3H), 2.82 – 2.70 (m, 2H), 2.27 (td, J = 7.2, 1.3 Hz, 2H), 1.80 (p, J = 7.2 Hz, 2H). ^{13}C NMR ($CDCl_3$, 101 MHz): δ 173.56, 143.64, 142.47, 135.29, 131.84, 131.49, 130.91, 130.05, 129.84, 128.31, 128.28, 128.14, 128.01, 127.65, 125.97, 123.45, 123.20, 92.94, 88.96, 51.59, 33.37, 32.62, 24.17. HRMS for $C_{25}H_{22}NaO_2S$ $[M+Na]^+$ calcd. 409.1238, found 409.1246.

Preparation of **S11**

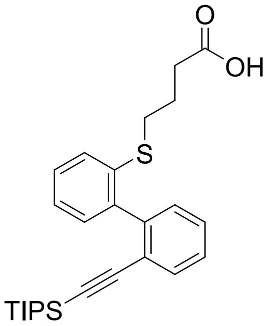

Compound **S7** (510 mg, 1.09 mmol) was dissolved in methanol (8 mL). A solution of NaOH (437 mg, 10.93 mmol) in H_2O (5 mL) was added to the reaction. After stirring at 65 °C overnight, the reaction mixture was evaporated under vacuum and acidify with 1.0 M HCl to pH 1-2. The aqueous solution was extracted by EtOAc. The organic layer was washed with brine, dried over Na_2SO_4, and then concentrated under vacuum. Flash column chromatography (20% EtOAc/Hex) was used to purify the desired compound **S11** (490 mg, 99%). ^1H NMR ($CDCl_3$, 400 MHz): δ 7.62 – 7.56 (m, 1H), 7.45 – 7.40 (m, 1H), 7.38 – 7.19 (m, 6H), 2.73 (q, J = 6.9 Hz, 2H), 2.34 (t, J = 7.3 Hz, 2H), 1.80 (p, J = 7.2 Hz, 2H), 0.92 (s, 21H). ^{13}C NMR ($CDCl_3$, 101 MHz): δ 179.17, 143.73, 142.51, 134.97, 132.77, 130.80, 130.04, 129.95, 128.21, 127.95, 127.49, 126.13, 123.46, 105.74, 94.13, 33.33, 32.57, 23.83, 18.63, 11.29. HRMS for $C_{27}H_{36}NaO_2SSi$ $[M+Na]^+$ calcd. 475.2103, found 475.2101.

Preparation of **S12**

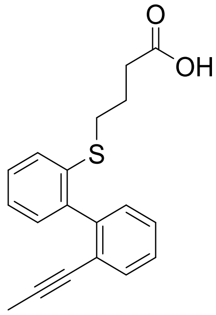

Compound **S8** (100 mg, 0.31 mmol) was dissolved in methanol (5 mL). A solution of NaOH (123 mg, 3.08 mmol) in H_2O (5 mL) was added to the reaction. After stirring at 75 °C overnight, the reaction mixture was evaporated under vacuum and acidify with 1.0 M HCl to pH 1-2. The aqueous solution was extracted by EtOAc. The organic layer was washed with brine, dried over Na_2SO_4, and then concentrated under vacuum. The oil **S12** (84 mg, 84%) was directly used for next step without purification. ^1H NMR ($CDCl_3$, 400 MHz): δ 7.51 – 7.44 (m, 2H), 7.34 – 7.28 (m, 3H), 7.26 – 7.23 (m, 3H), 2.81 – 2.71 (m, 2H), 2.34 (t, *J* = 7.4 Hz, 2H), 1.86 – 1.76 (m, 5H). ^{13}C NMR ($CDCl_3$, 101 MHz): δ 179.68, 143.09, 142.82, 134.79, 132.28, 130.76, 130.01, 129.52, 128.07, 127.49, 127.22, 126.04, 123.83, 89.29, 78.82, 33.28, 32.62, 23.85, 4.40. HRMS for $C_{19}H_{17}O_2S$ $[M-H]^+$ calcd. 309.0953, found 309.0949.

Preparation of **S13**

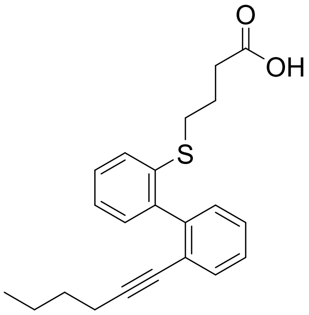

Compound **S9** (67 mg, 0.18 mmol) was dissolved in methanol (4 mL). A solution of NaOH (73 mg, 1.83 mmol) in H_2O (1 mL) was added to the reaction. After stirring at 65 °C for 1 hr, the reaction mixture was evaporated under vacuum and acidify with 1.0 M HCl to pH 1-2. The aqueous solution was extracted by EtOAc. The organic layer was washed with brine, dried over Na_2SO_4, and then concentrated under vacuum. The oil **S13** (60 mg, 93%) was directly used for next step without purification. ^1H NMR ($CDCl_3$, 400 MHz): δ 7.51 – 7.40 (m, 3H), 7.34 – 7.27 (m, 3H), 7.27 – 7.22 (m, 2H), 2.75 (td, *J* = 7.0, 3.5 Hz, 2H), 2.35 (t, *J* = 7.3 Hz, 2H), 2.19 (t, *J* = 6.8 Hz, 2H), 1.81 (p, *J* = 7.4 Hz, 2H), 1.33 – 1.22 (m, 2H), 1.19 – 1.06 (m, 2H), 0.78 (t, *J* = 7.3 Hz, 3H). ^{13}C NMR ($CDCl_3$, 101 MHz): δ 179.64, 143.28, 142.90, 134.80, 131.97, 130.73, 129.95, 129.86, 128.03, 127.49, 127.16, 126.02, 123.91, 93.97, 79.76, 33.26, 32.60, 30.54, 23.80, 21.62, 19.12, 13.75. HRMS for $C_{22}H_{23}O_2S$ $[M-H]^+$ calcd. 351.1419, found 351.1416.

Preparation of **S14**

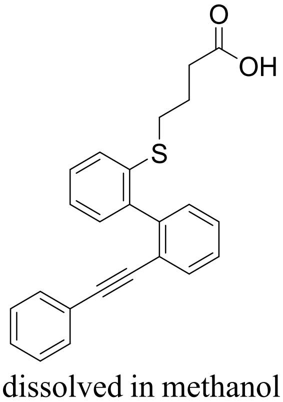

Compound **S10** (49 mg, 0.13 mmol) was dissolved in methanol (5 mL). A solution of NaOH (53 mg, 1.34 mmol) in H_2O (3 mL) was added to the reaction. After stirring at 65 °C for 1 hr, the reaction mixture was evaporated under vacuum and acidify with 1.0 M HCl to pH 1-2. The aqueous solution was extracted by EtOAc. The organic layer was washed with brine, dried over Na_2SO_4, and then concentrated under vacuum. The oil **S14** (44 mg, 93%) was directly used for next step without purification. ^{1}H NMR ($CDCl_3$, 400 MHz): δ 7.65 – 7.61 (m, 1H), 7.51 (d, *J* = 9.3 Hz, 1H), 7.42 – 7.27 (m, 6H), 7.22 – 7.18 (m, 3H), 7.17 – 7.11 (m, 2H), 2.75 (t, *J* = 7.1 Hz, 2H), 2.28 (td, *J* = 7.2, 1.3 Hz, 2H), 1.78 (p, *J* = 7.2 Hz, 2H). ^{13}C NMR ($CDCl_3$, 101 MHz): δ 173.45, 143.58, 142.59, 135.02, 131.81, 131.45, 130.93, 130.01, 130.00, 128.32, 128.27, 128.15, 128.03, 127.67, 126.09, 123.36, 123.15, 92.96, 88.92, 33.25, 33.47, 23.74. HRMS for $C_{24}H_{20}O_2S$ $[M+Na]^+$ calcd. 395.1087, found 395.1082.

Preparation of **S15**

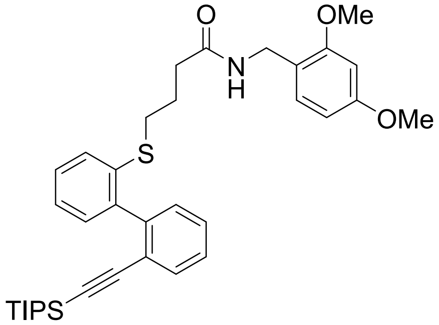

Compound **S11** (400 mg, 0.88 mmol) and 2,4-dimethoxybenzylamine (0.54 mL, 3.53 mmol) were dissolved in anhydrous DCM (3 mL) under argon. EDC (678 mg, 3.53 mmol), HOBt (478 mg, 3.53 mmol) and NEt_3 (0.49 mL, 3.53 mmol) were then added and the mixture was stirred overnight at room temp. To workup, H_2O (15 mL) was added to the mixture and extracted with DCM (10 mL). The organic layer was washed with brine, dried over Na_2SO_4, and then evaporated under vacuum. Flash column chromatography (20% EtOAc/Hex) was used to purify the desired compound **S15** (390 mg, 73%). ^{1}H NMR ($CDCl_3$, 400 MHz): δ 7.59 – 7.53 (m, 1H), 7.38 (dd, *J* = 8.1, 1.2 Hz, 1H), 7.34 – 7.27 (m, 2H), 7.26 – 7.16 (m, 4H), 7.14 (d, *J* = 8.1 Hz, 1H), 6.46 – 6.39 (m, 2H), 5.73 (t, *J* = 6.8 Hz, 1H), 4.30 (dd, *J* = 5.7, 2.4 Hz, 2H), 3.79 (s, 6H), 2.72 (q, *J* = 6.9 Hz, 2H), 2.16 – 2.08 (m, 2H), 1.82 (p, *J* = 7.0 Hz, 2H), 0.91 (s, 21H). ^{13}C NMR ($CDCl_3$, 101 MHz): δ 171.77, 160.67, 158.69, 143.85, 142.21, 135.28, 132.71, 130.72, 130.65, 130.07, 129.56, 128.23, 127.89, 127.41, 125.88, 123.51, 119.02, 105.69, 104.02, 98.75, 94.20, 55.56, 55.47, 39.07, 35.04, 33.48, 24.57, 18.64, 11.29. HRMS for $C_{36}H_{47}NNaO_3SSi$ $[M+Na]^+$ calcd. 624.2944, found 624.2944.

Preparation of **S16**

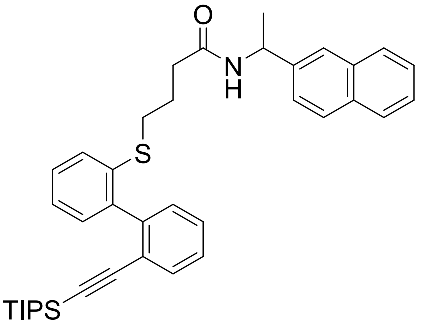

Compound **S11** (50 mg, 0.11 mmol) and (S)-1-(Naphthalen-2-yl)ethanamine (76 mg, 0.44 mmol) were dissolved in anhydrous DCM (3 mL) under argon. EDC (85 mg, 0.44 mmol), HOBt (60 mg, 0.44 mmol) and NEt_3 (0.06 mL, 0.44 mmol) were then added and the mixture was stirred overnight at room temp. To workup, H_2O (15 mL) was added to the mixture and extracted with DCM (10 mL). The organic layer was washed with brine, dried over Na_2SO_4, and then evaporated under vacuum. Flash column chromatography (20% EtOAc/Hex) was used to purify the desired compound **S16** (41 mg, 61%). ^{1}H NMR ($CDCl_3$, 400 MHz): δ 7.83 – 7.77 (m, 3H), 7.69 (s, 1H), 7.55 (d, *J* = 7.3 Hz, 1H), 7.51 – 7.43 (m, 2H), 7.42 – 7.34 (m, 2H), 7.27 – 7.16 (m, 6H), 5.63 (d, *J* = 7.8 Hz, 1H), 5.24 (p, *J* = 7.0 Hz, 1H), 2.79 – 2.69 (m, 2H), 2.20 – 2.11 (m, 2H), 1.84 (q, *J* = 6.5 Hz, 2H), 1.52 (d, *J* = 6.8 Hz, 3H), 0.91 (s, 21H). ^{13}C NMR ($CDCl_3$, 101 MHz): δ 171.35, 143.80, 142.18, 140.67, 134.96, 133.44, 132.73, 130.73, 130.15, 130.10, 130.08, 129.65, 128.63, 128.26, 128.05, 127.91, 127.75, 127.47, 126.37, 126.02, 125.96, 124.84, 124.60, 105.65, 48.81, 34.87, 33.29, 24.17, 21.88, 18.63, 11.26. HRMS for $C_{39}H_{47}NNaOSSi$ $[M+Na]^+$ calcd. 628.3040, found 628.3045.

Preparation of **S17**

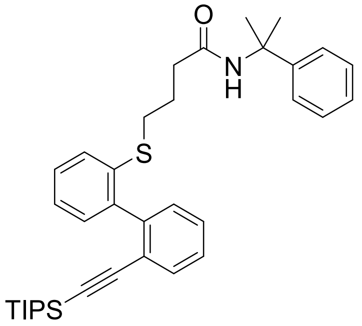

Compound **S11** (33 mg, 0.07 mmol) and 2-Phenylpropan-2-amine (39 mg, 0.29 mmol) were dissolved in anhydrous DCM (3 mL) under argon. EDC (56 mg, 0.29 mmol), HOBt (39 mg, 0.29 mmol) and NEt_3 (0.04 mL, 0.29 mmol) were then added and the mixture was stirred overnight at room temp. To workup, H_2O (15 mL) was added to the mixture and extracted with DCM (10 mL). The organic layer was washed with brine, dried over Na_2SO_4, and then evaporated under vacuum. Flash column chromatography (20% EtOAc/Hex) was used to purify the desired compound **S17** (20 mg, 48%). ^{1}H NMR ($CDCl_3$, 400 MHz): δ 7.60 – 7.56 (m, 1H), 7.42 – 7.38 (m, 1H), 7.37 – 7.25 (m, 9H), 7.24 – 7.18 (m, 2H), 5.60 (s, 1H), 2.76 (t, *J* = 6.8 Hz, 2H), 2.15 – 2.07 (m, 2H), 1.81 (q, *J* = 6.9 Hz, 2H), 1.64 (s, 6H), 0.93 (s, 21H). ^{13}C NMR ($CDCl_3$, 101 MHz): δ 171.21, 147.00, 143.85, 142.18, 135.15, 132.78, 130.74, 130.20, 129.53, 128.50, 128.26, 127.92, 127.47, 126.77, 125.89, 124.85, 123.47, 105.72, 94.31, 55.88, 35.43, 33.29, 24.15, 18.65, 11.30. HRMS for $C_{36}H_{47}NNaOSSi$ $[M+Na]^+$ calcd. 592.3045, found 592.3049.

Preparation of **S18**

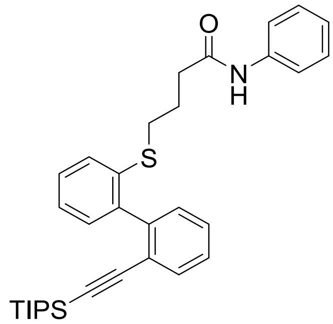

Compound **S11** (170 mg, 0.38 mmol) and aniline (0.14 mL, 1.50 mmol) were dissolved in anhydrous DCM (3 mL) under argon. EDC (288 mg, 1.50 mmol), HOBt (203 mg, 1.50 mmol) and NEt_3 (0.21 mL, 1.50 mmol) were then added and the mixture was stirred overnight at room temp. To workup, H_2O (15 mL) was added to the mixture and extracted with DCM (10 mL). The organic layer was washed with brine, dried over Na_2SO_4, and then evaporated under vacuum. Flash column chromatography (10% EtOAc/Hex) was used to purify the desired compound **S18** (110 mg, 56%). ^{1}H NMR ($CDCl_3$, 400 MHz): δ 7.60 (dd, *J* = 7.4, 1.8 Hz, 1H), 7.48 – 7.40 (m, 3H), 7.39 – 7.27 (m, 5H), 7.25 – 7.20 (m, 1H), 7.18 – 7.13 (m, 1H), 7.12 – 7.06 (m, 1H), 7.02 (s, 1H), 2.82 (t, *J* = 6.6 Hz, 2H), 2.28 (td, *J* = 7.1, 2.0 Hz, 2H), 1.91 (p, *J* = 7.0 Hz, 2H), 0.92 (s, 21H). ^{13}C NMR ($CDCl_3$, 101 MHz): δ 170.60, 143.80, 142.38, 137.91, 134.79, 132.74, 130.78, 130.21, 129.94, 129.40, 129.05, 128.31, 127.94, 127.48, 126.11, 124.29, 119.85, 118.66, 115.22, 105.66, 94.44, 35.71, 33.52, 24.09, 18.61, 11.25. HRMS for $C_{33}H_{40}NOSSi$ $[M-H]^+$ calcd. 526.2600, found 526.2601.

Preparation of **S19**

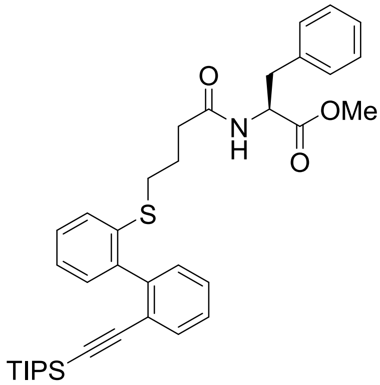

Compound **S11** (90 mg, 0.20 mmol) and N-Methylphenylalanine (39 mg, 0.22 mmol) were dissolved in anhydrous DMF (3 mL) under argon. EDC (42 mg, 0.22 mmol), HOBt (30 mg, 0.22 mmol) and NEt_3 (0.11 mL, 0.22 mmol) were then added and the mixture was stirred overnight at room temp. To workup, H_2O (15 mL) was added to the mixture and extracted with EtOAc (10 mL). The organic layer was washed with brine, dried over Na_2SO_4, and then evaporated under vacuum. Flash column chromatography (20% EtOAc/Hex) was used to purify the desired compound **S19** (60 mg, 49%). ^{1}H NMR ($CDCl_3$, 400 MHz): δ 7.58 (dd, *J* = 6.7, 2.4 Hz, 1H), 7.40 (d, *J* = 8.1 Hz, 1H), 7.36 – 7.29 (m, 2H), 7.29 – 7.17 (m, 7H), 7.07 – 7.03 (m, 2H), 5.76 (d, *J* = 7.9 Hz, 1H), 4.85 (dt, *J* = 8.0, 5.9 Hz, 1H), 3.71 (s, 3H), 3.18 – 2.97 (m, 2H), 2.78 – 2.60 (m, 2H), 2.13 (t, *J* = 7.4 Hz, 2H), 1.78 (pd, *J* = 7.0, 2.4 Hz, 2H), 0.92 (s, 21H). ^{13}C NMR ($CDCl_3$, 101 MHz): δ 172.16, 171.82, 143.83, 142.39, 135.94, 135.15, 132.74, 130.71, 130.07, 129.82, 129.35, 128.75, 128.23, 127.93, 127.45, 127.28, 126.00, 123.53, 105.73, 94.18, 53.09, 52.45, 38.06, 34.69, 33.46, 24.35, 18.64, 11.29. HRMS for $C_{37}H_{47}NNaO_3SSi$ $[M+Na]^+$ calcd. 636.2944, found 636.2943.

Preparation of **3a**

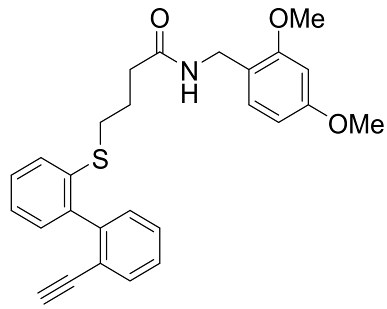

Compound **S15** (0.39 g, 0.65 mmol) was dissolved in anhydrous THF (2 mL). TBAF (1.62 mL of 1.0 M solution in THF, 1.62 mmol) was then added and the mixture was stirred overnight at room temp under argon. The reaction was quenched with saturated NH_4Cl solution and extracted by EtOAc. The organic layer was then dried over Na_2SO_4 and concentrated under vacuum. Flash column chromatography (20% EtOAc/Hex) was used to purify the desired compound **3a** (240 mg, 83%). ^{1}H NMR ($CDCl_3$, 400 MHz): δ 7.60 – 7.55 (m, 1H), 7.44 – 7.40 (m, 1H), 7.39 – 7.29 (m, 3H), 7.27 – 7.22 (m, 3H), 7.14 (d, *J* = 8.1 Hz, 1H), 6.46 – 6.38 (m, 2H), 5.78 (s, 1H), 4.30 (d, *J* = 5.7 Hz, 2H), 3.79 (s, *J* = 3.7 Hz, 3H), 3.78 (s, *J* = 3.7 Hz, 3H), 2.87 (s, 1H), 2.77 (td, *J* = 6.8, 4.0 Hz, 2H), 2.18 – 2.09 (m, 2H), 1.84 (td, *J* = 7.1, 2.8 Hz, 2H). ^{13}C NMR ($CDCl_3$, 101 MHz): δ 171.79, 160.67, 158.67, 143.91, 141.70, 135.22, 133.01, 130.76, 130.53, 130.29, 129.40, 128.50, 128.46, 127.57, 125.78, 121.92, 118.99, 104.04, 98.75, 82.71, 80.42, 55.56, 55.47, 39.05, 35.10, 33.34, 24.63. HRMS for $C_{27}H_{27}NNaO_3S$ $[M+Na]^+$ calcd. 468.1608, found 468.1609.

Preparation of **3b**

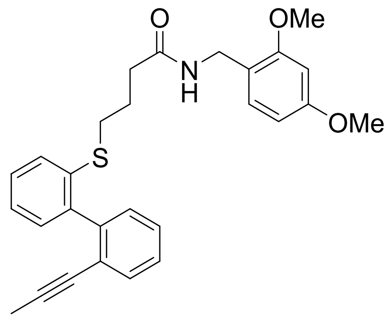

Compound **S12** (80 mg, 0.26 mmol) and 2,4-dimethoxybenzylamine (0.16 mL, 1.03 mmol) were dissolved in anhydrous DCM (3 mL) under argon. EDC (198 mg, 1.03 mmol), HOBt (139 mg, 1.03 mmol) and NEt_3 (0.14 mL, 1.03 mmol) were then added and the mixture was stirred overnight at room temp. To workup, H_2O (15 mL) was added to the mixture and extracted with DCM (10 mL). The organic layer was washed with brine, dried over Na_2SO_4, and then evaporated under vacuum. Flash column chromatography (40% EtOAc/Hex) was used to purify the desired compound **3b** (87 mg, 73%). ^{1}H NMR ($CDCl_3$, 400 MHz): δ 7.48 – 7.37 (m, 2H), 7.32 – 7.26 (m, 3H), 7.25 – 7.20 (m, 3H), 7.14 (d, *J* = 8.1 Hz, 1H), 6.46 – 6.38 (m, 2H), 5.80 (s, 1H), 4.31 (dd, *J* = 5.8, 1.6 Hz, 2H), 3.79 (s, 3H), 3.78 (s, 3H), 2.75 (t, *J* = 6.9 Hz, 2H), 2.12 (t, *J* = 7.3 Hz, 2H), 1.87 – 1.74 (m, 5H). ^{13}C NMR ($CDCl_3$, 101 MHz): δ 171.83, 160.63, 158.64, 143.19, 142.47, 135.11, 132.24, 130.65, 130.63, 130.09, 129.67, 128.13, 127.41, 127.15, 125.77, 123.83, 118.99, 104.02, 98.70, 89.37, 78.84, 55.53, 55.42, 39.00, 35.01, 33.51, 24.63, 4.41. HRMS for $C_{28}H_{28}NO_3S$ $[M-H]^+$ calcd. 458.1790, found 458.1788.

Preparation of **3c**

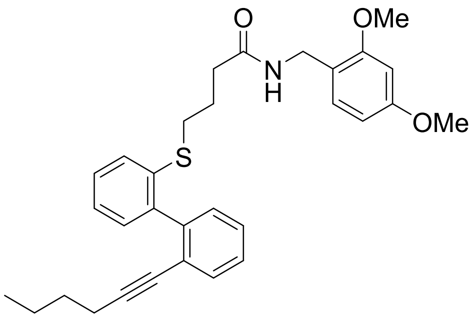

Compound **S13** (60 mg, 0.17 mmol) and 2,4-dimethoxybenzylamine (0.10 mL, 0.68 mmol) were dissolved in anhydrous DCM (3 mL) under argon. EDC (130 mg, 0.68 mmol), HOBt (92 mg, 0.68 mmol) and NEt_3 (0.09 mL, 0.68 mmol) were then added, and the mixture was stirred overnight at room temp. To workup, DCM was removed under vacuum. The residue was diluted with EtOAc (15 mL) and washed with H_2O (15 mL). The organic layer was then washed with brine, dried over Na_2SO_4, and then evaporated under vacuum. Flash column chromatography (40% EtOAc/Hex) was used to purify the desired compound **3c** (67 mg, 78%). ^{1}H NMR ($CDCl_3$, 400 MHz): δ 7.48 – 7.37 (m, 2H), 7.31 – 7.26 (m, 3H), 7.24 – 7.20 (m, 3H), 7.14 (d, *J* = 8.2 Hz, 1H), 6.48 – 6.37 (m, 2H), 5.78 (s, 1H), 4.30 (dd, *J* = 5.8, 2.9 Hz, 2H), 3.79 (s, 3H), 3.78 (s, 3H), 2.74 (t, *J* = 6.8 Hz, 2H), 2.13 (dt, *J* = 17.3, 7.2 Hz, 4H), 1.83 (h, *J* = 7.7 Hz, 2H), 1.27 – 1.23 (m, 2H), 1.10 (dq, *J* = 14.2, 7.2 Hz, 2H), 0.76 (t, *J* = 7.3 Hz, 3H). ^{13}C NMR ($CDCl_3$, 101 MHz): δ 171.81, 160.62, 158.64, 143.40, 142.60, 135.13, 131.94, 130.67, 130.62, 129.96, 129.65, 128.08, 127.43, 127.11, 125.78, 123.93, 118.97, 103.97, 98.69, 94.05, 79.78, 55.54, 55.43, 39.01, 35.00, 33.54, 30.52, 24.56, 21.61, 19.10, 13.76. HRMS for $C_{31}H_{34}NO_3S$ $[M-H]^+$ calcd. 500.2259, found 500.2260.

Preparation of **3d**

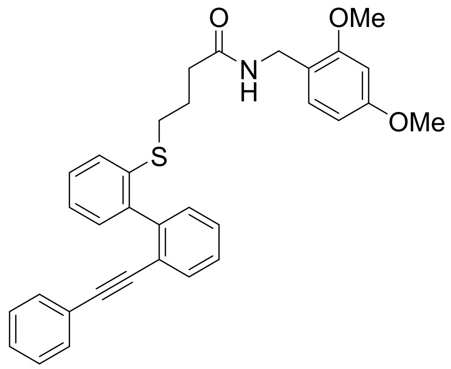

Compound **S14** (44 mg, 0.12 mmol) and 2,4-dimethoxybenzylamine (0.08 mL, 0.50 mmol) were dissolved in anhydrous DCM (3 mL) under argon. EDC (96 mg, 0.50 mmol), HOBt (67 mg, 0.50 mmol) and NEt_3 (0.07 mL, 0.50 mmol) were then added and the mixture was stirred overnight at room temp. To workup, H_2O (15 mL) was added to the mixture and extracted with DCM (10 mL). The organic layer was washed with brine, dried over Na_2SO_4, and then evaporated under vacuum. Flash column chromatography (40% EtOAc/Hex) was used to purify the desired compound **3d** (34 mg, 54%). ^{1}H NMR ($CDCl_3$, 400 MHz): δ 7.62 – 7.57 (m, 1H), 7.47 (dd, *J* = 7.9, 1.4 Hz, 1H), 7.40 – 7.27 (m, 6H), 7.24 – 7.17 (m, 3H), 7.15 – 7.08 (m, 3H), 6.48 – 6.29 (m, 2H), 5.68 (s, 1H), 4.24 (dd, *J* = 8.1, 5.7 Hz, 1H), 3.79 (s, 3H), 3.76 (s, 3H), 2.76 (td, *J* = 6.8, 1.3 Hz, 2H), 2.11 – 2.02 (m, 2H), 1.81 (q, *J* = 6.8 Hz, 2H). ^{13}C NMR ($CDCl_3$, 101 MHz): δ 171.79, 160.61, 158.63, 143.71, 142.29, 135.40, 131.78, 131.47, 131.45, 130.83, 130.64, 130.10, 129.72, 128.37, 128.33, 128.32, 128.22, 128.00, 127.62, 125.86, 123.34, 123.18, 118.98, 103.95, 98.69, 92.97, 88.97, 55.56, 55.43, 38.99, 34.96, 33.51, 24.63. HRMS for $C_{33}H_{31}NNaO_3S$ $[M+Na]^+$ calcd. 544.1922, found 544.1922.

Preparation of **3f**

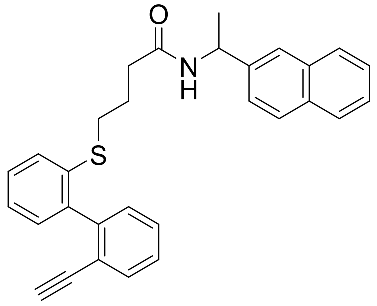

Compound **S16** (41 mg, 0.06 mmol) was dissolved in anhydrous THF (1 mL). TBAF (0.10 mL of 1.0 M solution in THF, 0.10 mmol) was then added and the mixture was stirred for 1 hr at room temp under argon. The reaction was quenched with saturated NH_4Cl solution and extracted by EtOAc. The organic layer was then dried over Na_2SO_4 and concentrated under vacuum. Flash column chromatography (30% EtOAc/Hex) was used to purify the desired compound **3f** (28 mg, 92%). ^{1}H NMR ($CDCl_3$, 400 MHz): δ 7.82 – 7.76 (m, 3H), 7.68 (s, 1H), 7.56 (d, *J* = 5.9 Hz, 1H), 7.50 – 7.23 (m, 10H), 5.72 (s, 1H), 5.23 (p, *J* = 7.0 Hz, 1H), 2.89 – 2.83 (m, 1H), 2.78 (t, *J* = 6.8 Hz, 2H), 2.17 (t, *J* = 6.9 Hz, 2H), 1.93 – 1.82 (m, 2H), 1.51 (d, *J* = 6.9 Hz, 3H). ^{13}C NMR ($CDCl_3$, 101 MHz): δ 171.37, 143.89, 141.71, 140.69, 135.06, 133.44, 133.02, 132.82, 130.57, 130.34, 129.48, 128.63, 128.51, 128.49, 128.04, 127.74, 127.60, 126.37, 126.02, 125.84, 124.83, 124.59, 121.88, 82.74, 80.54, 48.82, 34.97, 33.26, 24.37, 21.86. HRMS for $C_{30}H_{27}NNaOS$ $[M+Na]^+$ calcd. 472.1716, found 472.1711.

Preparation of **3g**

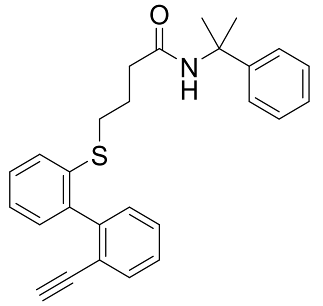

Compound **S17** (20 mg, 0.04 mmol) was dissolved in anhydrous THF (1 mL). TBAF (0.05 mL of 1.0 M solution in THF, 0.05 mmol) was then added and the mixture was stirred for 1 hr at room temp under argon. The reaction was quenched with saturated NH_4Cl solution and extracted by EtOAc. The organic layer was then dried over Na_2SO_4 and concentrated under vacuum. Flash column chromatography (20% EtOAc/Hex) was used to purify the desired compound **3g** (13 mg, 90%). ^{1}H NMR ($CDCl_3$, 400 MHz): δ 7.60 (dd, *J* = 7.4, 1.8 Hz, 1H), 7.46 – 7.42 (m, 1H), 7.42 – 7.29 (m, 7H), 7.29 – 7.18 (m, 4H), 5.66 (s, 1H), 2.91 (s, 1H), 2.79 (td, *J* = 6.8, 3.3 Hz, 2H), 2.13 (t, *J* = 7.3 Hz, 2H), 1.83 (qt, *J* = 6.9, 3.1 Hz, 2H), 1.64 (s, 6H). ^{13}C NMR ($CDCl_3$, 101 MHz): δ 171.19, 146.96, 143.91, 141.61, 135.18, 133.03, 130.55, 130.47, 130.34, 129.63, 129.27, 128.53, 128.49, 128.11, 127.60, 126.77, 125.77, 124.83, 121.91, 82.71, 80.51, 55.86, 35.45, 33.19, 29.20, 29.16, 24.24. HRMS for $C_{27}H_{27}NNaOS$ $[M+Na]^+$ calcd. 436.1717, found 436.1711.

Preparation of **3h**

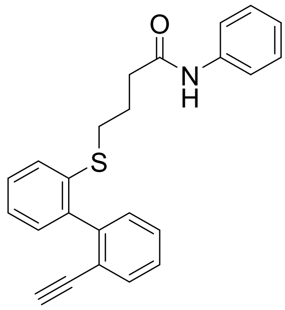

Compound **S18** (110 mg, 0.21 mmol) was dissolved in anhydrous THF (1 mL). TBAF (0.31 mL of 1.0 M solution in THF, 0.31 mmol) was then added and the mixture was stirred for 1 hr at room temp under argon. The reaction was quenched with saturated NH_4Cl solution and extracted by EtOAc. The organic layer was then dried over Na_2SO_4 and concentrated under vacuum. Flash column chromatography (20% EtOAc/Hex) was used to purify the desired compound **3h** (70 mg, 90%). ^{1}H NMR ($CDCl_3$, 400 MHz): δ 7.62 (dd, *J* = 7.4, 1.9 Hz, 1H), 7.49 (d, *J* = 8.5 Hz, 1H), 7.45 – 7.27 (m, 9H), 7.11 – 7.05 (m, 2H), 2.94 (s, 1H), 2.86 (q, *J* = 6.7 Hz, 2H), 2.32 (t, *J* = 7.1 Hz, 2H), 1.93 (h, *J* = 6.9 Hz, 2H). ^{13}C NMR ($CDCl_3$, 101 MHz): δ 170.58, 143.90, 141.87, 137.89, 134.89, 133.09, 130.66, 130.45, 129.79, 129.10, 128.60, 128.58, 127.67, 126.04, 124.34, 121.81, 119.85, 82.83, 80.61, 35.75, 33.43, 24.27. HRMS for $C_{24}H_{20}NOS$ [M-H]$^+$ calcd. 370.1266, found 370.1265.

Preparation of **3i**

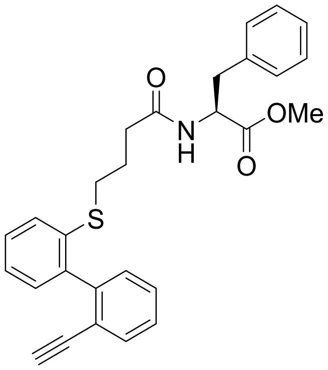

Compound **S19** (49 mg, 0.08 mmol) was dissolved in anhydrous THF (1 mL). TBAF (0.10 mL of 1.0 M solution in THF, 0.10 mmol) was then added and the mixture was stirred for 1 hr at room temp under argon. The reaction was quenched with saturated NH_4Cl solution and extracted by EtOAc. The organic layer was then dried over Na_2SO_4 and concentrated under vacuum. Flash column chromatography (40% EtOAc/Hex) was used to purify the desired compound **3i** (18 mg, 49%). ^{1}H NMR ($CDCl_3$, 400 MHz): δ 7.60 (dd, *J* = 7.6, 1.5 Hz, 1H), 7.44 (d, *J* = 7.8 Hz, 1H), 7.41 – 7.30 (m, 3H), 7.27 – 7.23 (m, 6H), 7.09 – 7.01 (m, 2H), 5.80 (d, *J* = 7.6 Hz, 1H), 4.84 (dt, *J* = 7.8, 5.9 Hz, 1H), 3.71 (s, 3H), 3.16 – 2.99 (m, 2H), 2.93 – 2.87 (m, 1H), 2.80 – 2.69 (m, 2H), 2.14 (t, *J* = 4.9 Hz, 2H), 1.81 (p, *J* = 6.5, 5.8 Hz, 2H). ^{13}C NMR ($CDCl_3$, 101 MHz): δ 172.19, 171.85, 143.91, 141.86, 135.94, 135.07, 133.05, 130.60, 130.29, 129.59, 129.36, 128.76, 128.54, 128.45, 127.61, 127.30, 125.91, 121.97, 82.71, 80.53, 53.10, 52.47, 38.04, 34.83, 33.26, 24.45. HRMS for $C_{28}H_{27}NNaO_3S$ [M+Na]$^+$ calcd. 480.1609, found 480.1609.

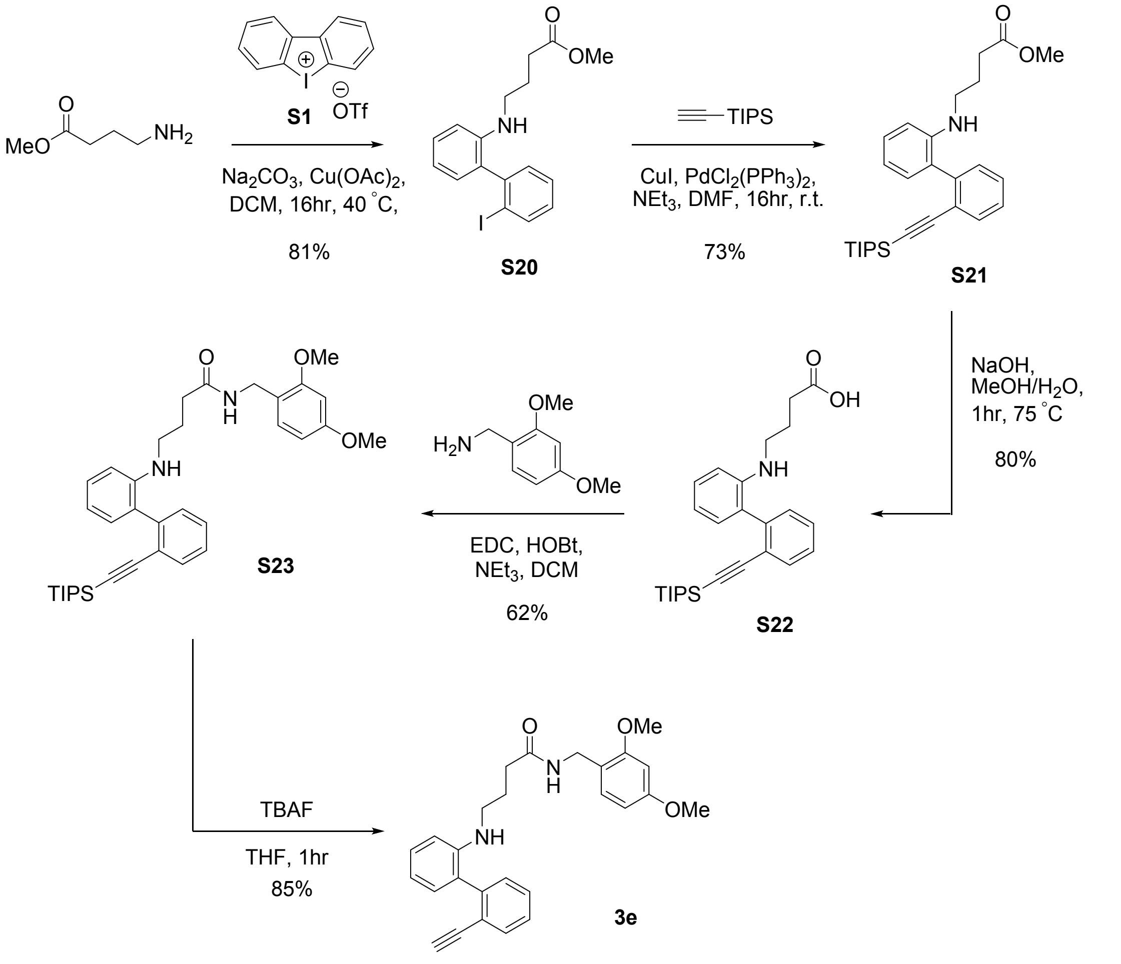

Scheme S3. Synthesis of model substrate **3e**.

Preparation of **S20**

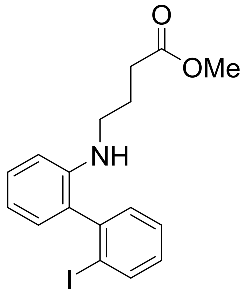

To a stirred solution of compound **S1** (200 mg, 0.47 mmol) in anhydrous DCM (5 mL) was added methyl 4-aminobenzoate hydrochloride (144 mg, 0.93 mmol), sodium carbonate (149 mg, 1.40 mmol) and $Cu(OAc)_2$ (25 mg, 0.14 mmol). The reaction was allowed to proceed at 40 °C under N_2 overnight. To workup, DCM was removed under vacuum. The remaining mixture was dissolved in EtOAc and washed with H_2O. The organic layer was wash with brine, dried over Na_2SO_4 and concentrated under vacuum to afford the solid **S20** (149 mg, 81%). ^{1}H NMR ($CDCl_3$, 400 MHz): δ 7.99 (d, *J* = 8.0 Hz, 1H), 7.48 – 7.41 (m, 1H), 7.34 – 7.27 (m, 2H), 7.11 – 7.05 (m, 1H), 6.95 (d, *J* = 7.4 Hz, 1H), 6.83 – 6.69 (m, 2H), 3.65 (s, 3H), 3.19 (t, *J* = 7.1 Hz, 2H), 2.37 (t, *J* = 7.3 Hz, 2H), 1.89 (t, *J* = 7.1 Hz, 2H). ^{13}C NMR ($CDCl_3$, 101 MHz): δ 173.74, 144.73, 144.11, 139.62, 131.00, 130.23, 129.92, 129.36, 129.30, 128.86, 116.69, 110.44, 101.19, 51.66, 43.24, 31.61, 24.58. HRMS for $C_{17}H_{19}INO_2$ $[M+H]^+$ calcd. 396.0460, found 396.0468.

Preparation of **S21**

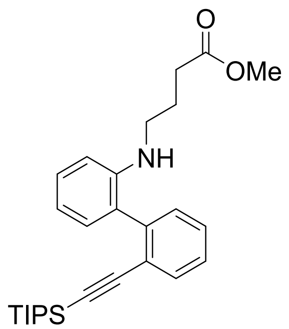

Compound **S20** (149 mg, 0.38 mmol), CuI (7 mg, 0.04 mmol), and $PdCl_2(PPh_3)_2$ (26 mg, 0.04 mmol) were dissolved in anhydrous DMF (3 mL) and NEt_3 (1 mL) under argon. (Triisopropylsilyl)acetylene (0.17 mL, 0.75 mmol) was then added dropwise. After stirring overnight at r.t. overnight, the reaction mixture was diluted with EtOAc, which was then washed with H_2O and brine. The organic layer was dried over Na_2SO_4 and concentrated under vacuum. Flash column chromatography (5% EtOAc/Hex) was used to purify the desired compound **S21** (124 mg, 73%). ^{1}H NMR ($CDCl_3$, 400 MHz): δ 7.61 (dd, J = 7.8, 1.5 Hz, 1H), 7.40 – 7.35 (m, 1H), 7.34 – 7.27 (m, 2H), 7.21 – 7.15 (m, 1H), 7.04 (dd, J = 7.4, 1.6 Hz, 1H), 6.73 – 6.68 (m, 1H), 6.65 (d, J = 7.2 Hz, 1H), 3.62 (s, 3H), 3.11 (q, J = 7.0 Hz, 2H), 2.32 (t, J = 7.3 Hz, 2H), 1.90 – 1.76 (m, 2H), 0.93 (s, 21H). ^{13}C NMR ($CDCl_3$, 101 MHz): δ 173.86, 145.28, 142.25, 133.43, 130.47, 130.38, 128.90, 128.85, 127.44, 126.88, 124.01, 116.83, 110.43, 105.55, 93.98, 51.69, 43.48, 31.71, 24.74, 18.63, 11.30. HRMS for $C_{28}H_{40}NO_2Si$ $[M+H]^+$ calcd. 450.2828, found 450.2828.

Preparation of **S22**

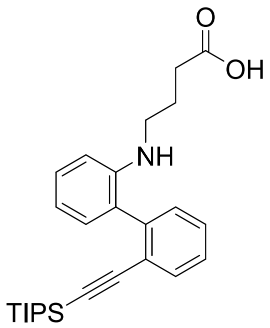

Compound **S21** (124 mg, 0.28 mmol) was dissolved in methanol (4 mL). A solution of NaOH (110 mg, 2.76 mmol) in H_2O (1 mL) was added to the reaction. After stirring at 75 °C for 1 hr, the reaction mixture was evaporated under vacuum and acidify with 1.0 M HCl to pH 1-2. The aqueous solution was extracted by EtOAc. The organic layer was washed with brine, dried over Na_2SO_4, and then concentrated under vacuum. The oil **S22** (96 mg, 80%) was directly used for next step without purification. ^{1}H NMR ($CDCl_3$, 400 MHz): δ 7.61 (dd, J = 7.6, 1.7 Hz, 1H), 7.40 – 7.35 (m, 1H), 7.34 – 7.27 (m, 2H), 7.22 – 7.16 (m, 1H), 7.06 (dd, J = 7.4, 1.7 Hz, 1H), 6.73 (t, J = 7.4 Hz, 1H), 6.67 (d, J = 7.1 Hz, 1H), 3.15 (q, J = 6.8 Hz, 2H), 2.37 (t, J = 7.3 Hz, 2H), 1.91 – 1.81 (m, 2H), 0.94 (s, 21H). ^{13}C NMR ($CDCl_3$, 101 MHz): δ 179.31, 145.13, 142.15, 133.42, 130.44, 130.39, 128.91, 128.86, 127.48, 127.04, 123.94, 117.04, 110.60, 105.51, 94.02, 43.41, 31.66, 24.44, 18.62, 11.28. HRMS for $C_{27}H_{38}NO_2Si$ $[M+H]^+$ calcd. 436.2672, found 436.2672.

Preparation of **S23**

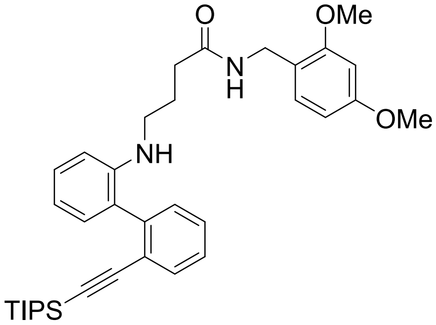

Compound **S22** (96 mg, 0.22 mmol) and 2,4-dimethoxybenzylamine (0.13 mL, 0.88 mmol) were dissolved in anhydrous DCM (4 mL) under argon. EDC (169 mg, 0.88 mmol), HOBt (119 mg, 0.88 mmol) and NEt_3 (0.12 mL, 0.88 mmol) were then added and the mixture was stirred overnight at room temp. To workup, the mixture was evaporated under vacuum to remove solvent. The residue was diluted with EtOAc and washed with H_2O. The organic layer was washed with brine, dried over Na_2SO_4, and then evaporated under vacuum. Flash column chromatography (30% EtOAc/Hex) was used to purify the desired compound **S23** (80 mg, 62%). ^1H NMR ($CDCl_3$, 400 MHz): δ 7.58 (d, J = 7.6 Hz, 1H), 7.44 (d, J = 7.4 Hz, 1H), 7.40 – 7.32 (m, 2H), 7.28 (m, 1H), 7.15 – 7.12 (m, 1H), 7.03 (d, J = 7.5 Hz, 1H), 6.69 (t, J = 7.4 Hz, 1H), 6.61 (d, J = 8.3 Hz, 1H), 6.46 – 6.38 (m, 2H), 5.76 (s, 1H), 4.31 (d, J = 5.8 Hz, 2H), 3.78 (s, 3H), 3.79 (s, 3H), 3.09 (q, J = 6.7, 6.3 Hz, 2H), 2.14 (t, J = 7.6 Hz, 2H), 1.88 – 1.79 (m, 2H), 0.93 (s, 21H). ^{13}C NMR ($CDCl_3$, 101 MHz): δ 171.96, 160.69, 158.70, 145.35, 142.26, 138.82, 133.44, 132.89, 132.04, 130.76, 130.41, 129.74, 128.92, 128.18, 128.03, 127.62, 127.45, 123.94, 119.04, 104.10, 98.79, 55.56, 55.46, 39.05, 34.08, 31.31, 25.32, 18.62, 11.30. HRMS for $C_{36}H_{49}N_2O_3Si$ $[M+H]^+$ calcd. 585.3513, found 585.3512.

Preparation of **3e**

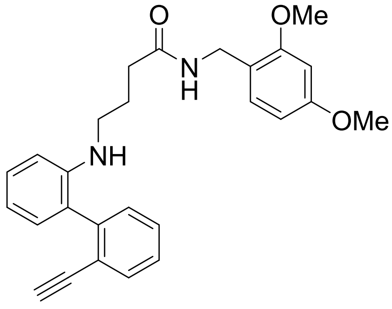

Compound **S23** (80 mg, 0.14 mmol) was dissolved in anhydrous THF (1 mL). TBAF (0.34 mL of 1.0 M solution in THF, 0.34 mmol) was then added and the mixture was stirred for 1 hr at room temp under argon. The reaction was quenched with saturated NH_4Cl solution and extracted by EtOAc. The organic layer was then dried over Na_2SO_4 and concentrated under vacuum. Flash column chromatography (40% EtOAc/Hex) was used to purify the desired compound **3e** (50 mg, 85%). ^1H NMR ($CDCl_3$, 400 MHz): δ 7.60 (dd, J = 7.7, 1.6 Hz, 1H), 7.41 (td, J = 7.6, 1.5 Hz, 1H), 7.35 – 7.27 (m, 2H), 7.26 – 7.21 (m, 1H), 7.15 (d, J = 8.1 Hz, 1H), 7.05 (dd, J = 7.4, 1.7 Hz, 1H), 6.75 (td, J = 7.3, 1.1 Hz, 1H), 6.68 (d, J = 1.2 Hz, 1H), 6.46 – 6.38 (m, 2H), 5.86 (s, 1H), 4.31 (d, J = 5.7 Hz, 2H), 3.79 (s, 3H), 3.77 (s, 3H), 3.13 (q, J = 3.7 Hz, 2H), 2.92 (s, 1H), 2.17 (t, J = 7.3 Hz, 2H), 1.87 (hept, J = 6.8 Hz, 2H). ^{13}C NMR ($CDCl_3$, 101 MHz): δ 171.96, 160.67, 158.66, 145.42, 142.26, 133.74, 130.79, 130.71, 130.39, 129.52, 129.19, 127.61, 126.36, 122.25, 119.00, 116.72, 110.67, 104.08, 98.79, 82.56, 80.27, 55.55, 55.45, 43.24, 39.03, 34.00, 25.30. HRMS for $C_{27}H_{29}N_2O_3$ $[M+H]^+$ calcd. 429.2173, found 429.2178.

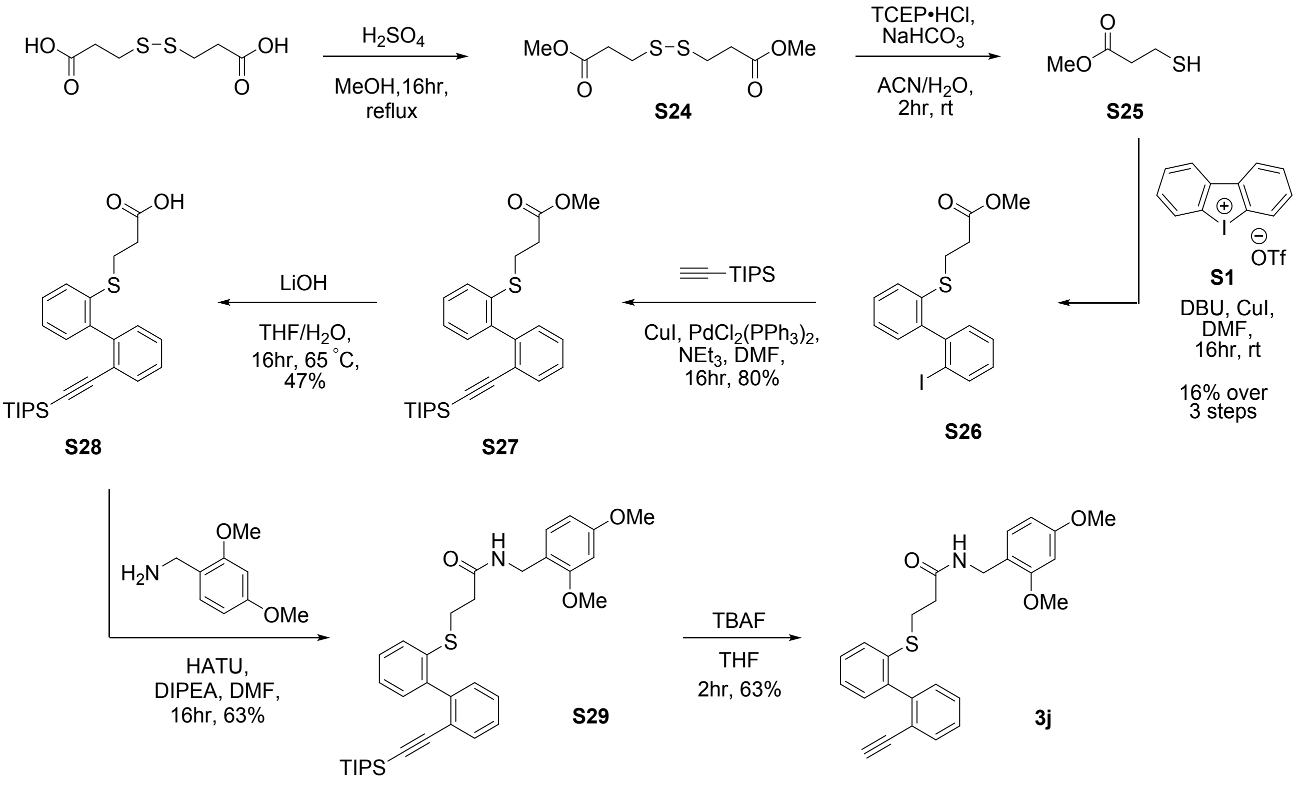

Scheme S4. Synthesis of model substrate **3j**.

Preparation of **S26**

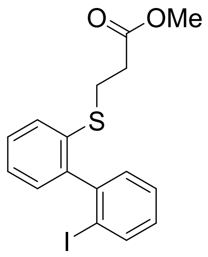

Sulfuric acid (1 mL) was added to a solution of 3,3'-Dithiodipropionic acid (905 mg, 3.8 mmol) in MeOH (15 mL). After refluxing overnight, the methanol was evaporated under reduced pressure. The crude was diluted with EtOAc and washed with H_2O and brine. The organic phase was dried over Na_2SO_4 and then concentrated under vacuum to afford **S24** as a crude mixture. A solution of TCEP·HCl (1.6 g, 5.6 mmol) and $NaHCO_3$ (622 mg, 7.4 mmol) in H_2O (1 mL) was added to crude **S24** (444 mg, 3.7 mmol) in ACN (1 mL). After stirring for 2 hr at room temp, the reaction mixture was diluted with H_2O and extracted with EtOAc. The organic layer was washed with brine and dried over Na_2SO_4. The solvent was removed under vacuum to give compound **S25** as a crude mixture. Compound **S1** (1422 g, 3.3 mmol), CuI (57 mg, 0.3 mmol), and crude **S25** (361 mg, 3 mmol) were mixed together in anhydrous DMF (3 mL) and chilled to 0°C in an ice bath under argon. DBU (0.9 mL, 6 mmol) was then added dropwise. The mixture was allowed to warm to room temp and then stirred overnight. To workup, the reaction mixture was diluted with H_2O and then extracted with EtOAc. The organic layer was wash with brine, dried over Na_2SO_4 and concentrated under vacuum. Flash column chromatography (20% EtOAc/Hex) was used to purify the desired compound **S26** (170 mg, 16%). ^{1}H NMR ($CDCl_3$, 400 MHz): δ 7.95 (d, J = 7.9, 1H), 7.46 – 7.36 (m, 3H), 7.30 – 7.22 (m, 2H), 7.13 – 7.04 (m, 2H), 3.66 (s, 3H), 3.10 – 3.01 (m, 2H), 2.60 – 2.56 (m, 2H). ^{13}C NMR ($CDCl_3$, 101 MHz): δ 172.17, 145.31, 145.10, 138.94, 134.51, 130.31, 130.28, 129.17, 128.65, 128.63, 127.91, 126.01, 100.20, 51.81, 34.05, 28.13. HRMS for $C_{16}H_{15}INaO_2S$ $[M+Na]^+$ calcd. 420.9735, found 420.9749.

Preparation of **S27**

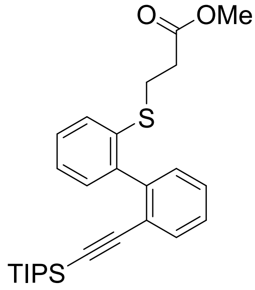

Compound **S26** (170 mg, 0.43 mmol), CuI (16.4 mg, 0.086mmol), and $PdCl_2(PPh_3)_2$ (30 mg, 0.043mmol) were dissolved in anhydrous DMF (3 mL) and NEt_3 (0.2 mL) under argon. (Triisopropylsilyl)acetylene (157 mg, 0.86 mmol) was then added dropwise. After stirring overnight at 60 °C overnight, the reaction mixture was diluted with EtOAc, which was then washed with H_2O and brine. The organic layer was dried over Na_2SO_4 and concentrated under vacuum. Flash column chromatography (10% EtOAc/Hex) was used to purify the desired compound **S27** (156 mg, 80%). ^{1}H NMR ($CDCl_3$, 400 MHz): δ 7.60 – 7.57 (m, 1H), 7.46 – 7.42 (m, 1H), 7.38 – 7.30 (m, 2H), 7.30 – 7.27 (m, 2H), 7.25 – 7.20 (m, 2H), 3.62 (s, 3H), 2.92 (t, J = 7.6, 2H), 2.51 – 2.45 (m, 2H), 0.92 (s, 21H). ^{13}C NMR ($CDCl_3$, 101 MHz): δ 172.39, 143.60, 142.70, 134.39, 132.75, 130.87, 130.18, 130.01, 128.26, 127.92, 127.48, 126.39, 123.40, 105.69, 94.18, 51.81, 34.16, 29.06, 18.62, 11.27. HRMS for $C_{27}H_{36}NaO_2SSi$ $[M+Na]^+$ calcd. 475.2103, found 475.2109.

Preparation of **S28**

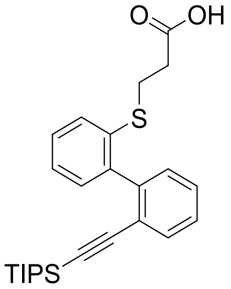

Compound **S27** (156 mg, 0.34 mmol) was dissolved in a 9:1 TMF/MeOH mixture (3 mL). LiOH (144 mg, 3.4 mmol) was then added to the reaction. After stirring at 65 °C overnight, the reaction mixture was evaporated under vacuum and acidify with 1.0 M HCl to pH 1-2. The aqueous solution was extracted by EtOAc. The organic layer was washed with brine, dried over Na_2SO_4, and then concentrated under vacuum. Flash column chromatography (30% EtOAc/Hex) was used to purify the desired compound **S28** (70 mg, 47%). ^{1}H NMR ($CDCl_3$, 400 MHz): δ 7.60 – 7.57 (m, 1H), 7.47 – 7.43 (m, 1H), 7.38 – 7.31 (m, 2H), 7.30 – 7.20 (m, 4H), 2.90 (t, J = 7.6, 2H), 2.55 – 2.48 (m, 2H), 0.92 (s, 21H). ^{13}C NMR ($CDCl_3$, 101 MHz): δ 177.60, 143.56, 142.90, 134.12, 132.79, 130.97, 130.49, 129.99, 128.31, 127.97, 127.54, 126.62, 123.42, 105.67, 94.28, 34.07, 28.74, 18.63, 11.28. HRMS for $C_{26}H_{35}O_2SSi$ $[M+Na]^+$ calcd. 439.2127, found 439.2120.

Preparation of **S29**

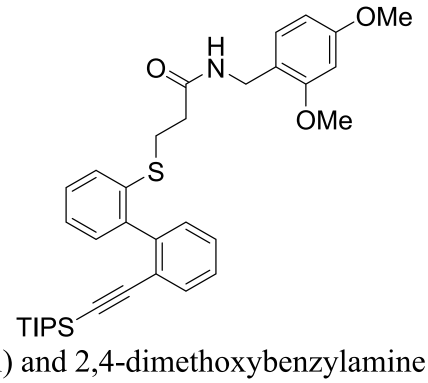

Compound **S28** (70 mg, 0.16 mmol) and 2,4-dimethoxybenzylamine (54 mg, 0.32 mmol) were dissolved in anhydrous DMF (2 mL) under argon. HATU (72 mg, 0.19 mmol) and DIPEA (62 mg, 0.48 mmol) were then added and the mixture was stirred overnight at 45°C. To workup, H_2O was added to the mixture and extracted with EtOAc. The organic layer was washed with a 1M HCl solution, a $NaHCO_3$ solution, a

LiCl solution, brine, and then dried over Na_2SO_4. The organic solvent was then evaporated under vacuum. Flash column chromatography (30% EtOAc/Hex) was used to purify the desired compound **S29** (59 mg, 63%). ^{1}H NMR ($CDCl_3$, 400 MHz): δ 7.56 – 7.51 (m, 1H), 7.46 – 7.41 (m, 1H), 7.35 – 7.27 (m, 4H), 7.25 – 7.19 (m, 2H), 7.11 (d, J = 8.1, 1H), 6.44 – 6.39 (m, 2H), 5.82 (s, 1H), 4.29 (d, J = 5.8, 2H), 3.80 (s, 3H), 3.79 (s, 3H), 2.96 (t, J = 7.6, 2H), 2.32 – 2.27 (m, 2H), 0.91 (s, 21H). ^{13}C NMR ($CDCl_3$, 101 MHz): δ ^{13}C NMR ($CDCl_3$, 101 MHz): δ 170.69, 160.58, 158.59, 143.66, 142.49, 134.56, 132.77, 130.85, 130.55, 130.19, 130.11, 128.34, 127.99, 127.48, 126.27, 123.23, 118.85, 105.74, 103.99, 98.66, 94.37, 55.55, 55.44, 38.98, 36.33, 30.16, 18.62, 11.27. HRMS for $C_{35}H_{45}NNaO_3SSi$ $[M+Na]^+$ calcd. 610.2787, found 610.2797.

Preparation of **3j**

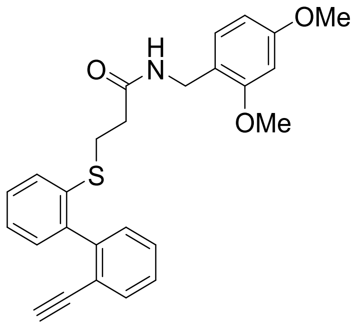

Compound **S29** (59 mg, 0.1 mmol) was dissolved in anhydrous THF (2 mL). TBAF (0.2 mL of 1.0 M solution in THF, 0.2 mmol) was then added and the mixture was stirred for 2 hr at room temp under argon. The reaction was quenched with saturated NH_4Cl solution and extracted by EtOAc. The organic layer was then dried over Na_2SO_4 and concentrated under vacuum. Flash column chromatography (50% EtOAc/Hex) was used to purify the desired compound **3j** (27 mg, 63%). ^{1}H NMR ($CDCl_3$, 400 MHz): δ 7.56 – 7.53 (m, 1H), 7.49 – 7.46 (m, 1H), 7.40 – 7.30 (m, 3H), 7.29 – 7.24 (m, 3H), 7.14 (d, J = 8.1, 1H), 6.44 – 6.40 (m, 2H), 5.89 (s, 1H), 4.29 (d, J = 5.8, 2H), 3.80 (s, 3H), 3.80 (s, 3H), 3.04 – 2.96 (m, 2H), 2.90 (s, 1H), 2.33 – 2.28 (m, 2H). ^{13}C NMR ($CDCl_3$, 101 MHz): δ 170.62, 160.64, 158.63, 143.77, 142.09, 134.52, 133.08, 130.72, 130.69, 130.31, 130.17, 128.58, 128.55, 127.62, 126.24, 121.73, 118.85, 104.03, 98.71, 82.80, 80.47, 55.57, 55.47, 39.04, 36.35, 29.95. HRMS for $C_{26}H_{25}NNaO_3S$ $[M+Na]^+$ calcd. 454.1453, found 454.1454.

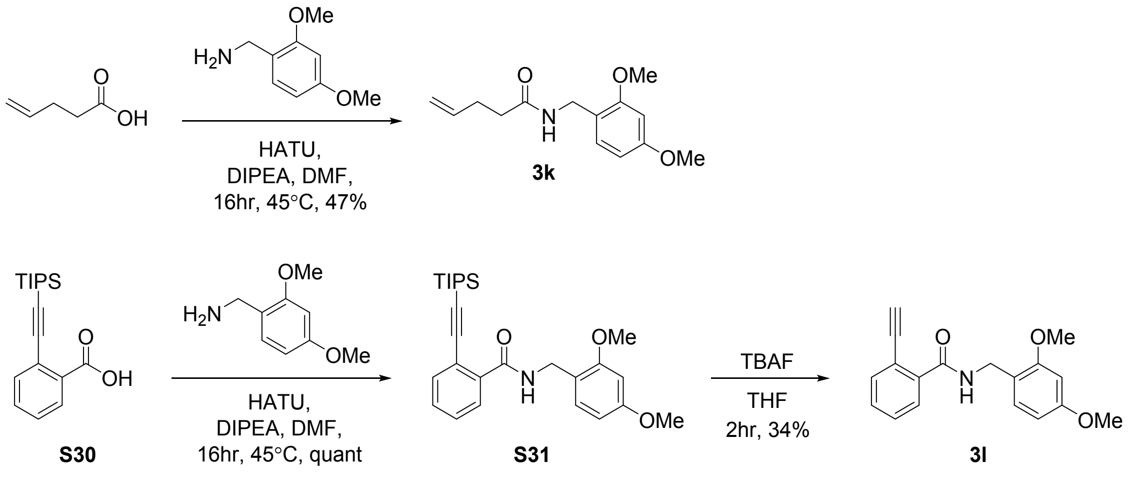

Scheme S5. Synthesis of model substrates **3k-l**.

Preparation of **S31**

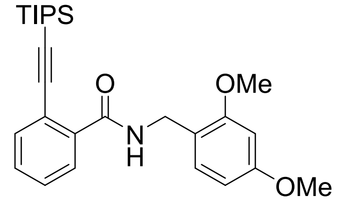

DIPEA (60 mg, 0.46 mmol) was added to the mixture of 2,4-dimethoxybenzylamine (50 mg, 0.3 mmol), **S30** (47 mg, 0.15 mmol) and HATU (84 mg, 0.22 mmol) in anhydrous DMF (2 mL) under argon. The reaction mixture was stirred overnight at 45°C. To workup, the mixture was diluted with EtOAc and washed with H_2O. Then the organic layer was washed by brine, dried over Na_2SO_4 and concentrated under vacuum. Flash column chromatography (30% EtOAc/Hex) was used to purify the desired compound **S31** (69 mg, quant). ^1H NMR ($CDCl_3$, 400 MHz): δ 8.08 – 8.05 (m, 1H), 7.86 – 7.80 (m, 1H), 7.56 – 7.53 (m, 1H), 7.43 – 7.35 (m, 2H), 7.28 – 7.25 (m, 1H), 6.45 – 6.40 (m, 2H), 4.60 (d, *J* = 5.8, 2H), 3.79 (s, 3H), 3.78 (s, 3H), 1.07 (s, 21H). ^{13}C NMR ($CDCl_3$, 101 MHz): δ 166.07, 160.49, 158.55, 135.84, 134.56, 130.48, 130.35, 130.29, 128.99, 119.87, 118.88, 105.49, 104.01, 98.58, 98.55, 55.51, 55.41, 39.11, 18.75, 11.34. HRMS for $C_{27}H_{37}NNaO_3S$ $[M+Na]^+$ calcd. 474.2440, found 474.2442.

Preparation of **3k**

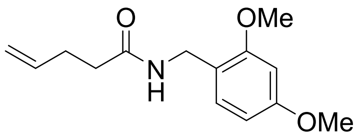

DIPEA (194 mg, 1.5 mmol) was added to the mixture of 2,4-dimethoxybenzylamine (100 mg, 0.6 mmol), 4-Pentenoic acid (50 mg, 0.5 mmol), and HATU (228 mg, 0.6 mmol) in anhydrous DMF (2 mL) under argon. The reaction mixture was stirred overnight at 45°C. To workup, the mixture was diluted with EtOAc and washed with H_2O. Then the organic layer was washed by brine, dried over Na_2SO_4 and concentrated under vacuum. Flash column chromatography (50% EtOAc/Hex) was used to purify the desired compound **3k** (70 mg, 47%). ^1H NMR ($CDCl_3$, 400 MHz): δ 7.13 (d, *J* = 8.2, 1H), 6.43 – 6.37 (m, 2H), 6.10 (s, 1H), 5.82 – 5.72 (m, 1H), 5.03 – 4.91 (m, 2H), 4.32 (d, *J* = 5.7, 2H), 3.78 (s, 3H), 3.75 (s, 3H), 2.39 – 2.30 (m, 2H), 2.26 – 2.17 (m, 2H). ^{13}C NMR ($CDCl_3$, 101 MHz): δ 146.84, 141.12, 127.10, 126.80, 125.59(2), 119.75(2), 63.24, 55.08(2), 44.98, 26.42, 24.80. HRMS for $C_{14}H_{19}NNaO_3$ $[M+Na]^+$ calcd. 272.1263, found 272.1284.

Preparation of **3l**

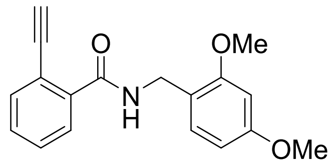

Compound **S31** (69 mg, 0.15 mmol) was dissolved in anhydrous THF (2 mL). TBAF (0.3 mL of 1.0 M solution in THF, 0.3 mmol) was then added and the mixture was stirred for 2 hr at room temp under argon. The reaction was quenched with saturated NH_4Cl solution and extracted by EtOAc. The organic layer was then dried over Na_2SO_4 and concentrated under vacuum. Flash column chromatography (40% EtOAc/Hex) was used to purify the desired compound **3l** (15 mg, 34%). ^{1}H NMR ($CDCl_3$, 400 MHz): δ 7.87 (d, J = 7.4, 1H), 7.67 (d, J = 7.6, 1H), 7.60 – 7.48 (m, 2H), 7.00 (d, J = 8.4, 1H), 6.45 (d, J = 2.4, 1H), 6.39 – 6.36 (m, 1H), 5.13 (s, 1H), 4.96 (s, 2H), 3.87 (s, 3H), 3.78 (s, 3H). ^{13}C NMR ($CDCl_3$, 101 MHz): δ 167.53, 160.25, 157.74, 141.73, 136.57, 131.99, 129.61, 129.47, 129.06, 123.34, 119.97, 117.55, 104.43, 98.45, 90.07, 55.54, 55.49, 37.24. HRMS for $C_{18}H_{17}NNaO_3$ $[M+Na]^+$ calcd. 318.1106, found 318.1098.

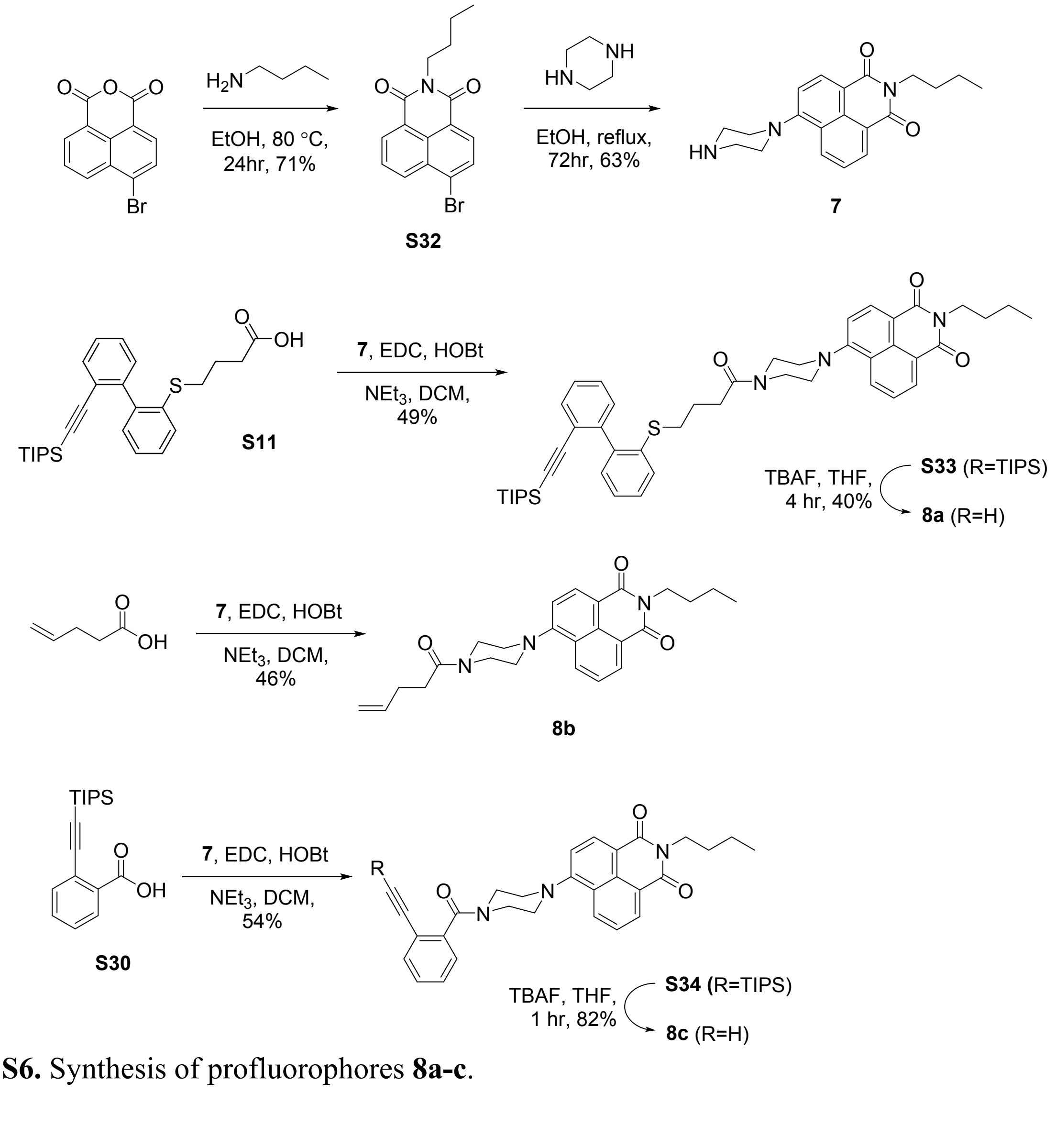

Scheme S6. Synthesis of profluorophores **8a-c**.

Preparation of **S32**

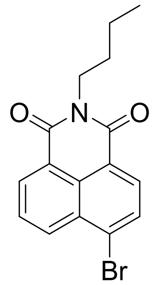

4-bromo-1,8-naphthalic anhydride (1.00 g, 3.61 mmol) was dissolved in EtOH (10 mL) and butyl-amine (251 mg, 3.61 mmol) was added. The solution was stirred at 80 °C for 24 hours. After cooling down to room temperature, the product was filtered, washed with H_2O and dried in vacuum to give product **S32** (1.41g, 71%). ^{1}H NMR ($CDCl_3$, 400 MHz): δ 8.65 (dd, J = 7.3, 1.2 Hz, 1H), 8.55 (dd, J = 8.5, 1.2 Hz, 1H), 8.40 (d, J = 7.9 Hz, 1H), 8.03 (d, J = 7.9 Hz, 1H), 7.84 (dd, J = 8.5, 7.3 Hz, 1H), 4.23 – 4.11 (m, 2H), 1.76 – 1.66 (m, 3H), 1.44 (h, J = 7.4 Hz, 2H), 0.97 (t, J = 7.4 Hz, 3H). ^{13}C NMR ($CDCl_3$, 101 MHz): δ 163.79, 163.76, 133.35, 132.15, 131.34, 131.23, 130.77, 130.33, 129.15, 128.22, 123.31, 122.45, 40.53, 30.31, 20.51, 13.97. HRMS for $C_{16}H_{15}BrNO_2$ $[M+H]^+$ calcd. 332.0286, found 332.0289.

Preparation of **7**

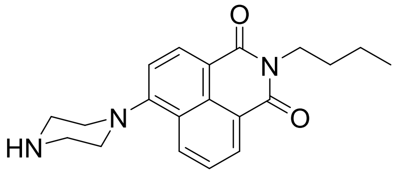

Piperazine (1.83 g, 21.22 mmol) was added to the solution of **S32** in EtOH (15 mL). The reaction mixture was stirred at reflux for 72 hr. To workup, the reaction mixture was concentrated under vacuum. The residue was dissolved in DCM (2 mL). The product was then isolated as yellow powder (906 mg, 63%) after trituration from hexane (20 mL). ^{1}H NMR ($CDCl_3$, 400 MHz): δ 8.58 (dd, J = 7.3, 1.2 Hz, 1H), 8.52 (d, J = 8.1 Hz, 1H), 8.41 (dd, J = 8.5, 1.2 Hz, 1H), 7.69 (dd, J = 8.5, 7.3 Hz, 1H), 7.21 (d, J = 8.1 Hz, 1H), 4.23 – 4.10 (m, 2H), 3.30 – 3.16 (m, 8H), 1.72 – 1.65 (m, 2H), 1.44 (dq, J = 14.8, 7.4 Hz, 2H), 0.97 (t, J = 7.3 Hz, 3H). ^{13}C NMR ($CDCl_3$, 101 MHz): δ 164.68, 164.22, 156.41, 132.69, 131.22, 130.37, 130.05, 126.36, 125.80, 123.49, 116.99, 115.13, 54.47(2), 46.34(2), 40.25, 30.42, 20.56, 14.02. HRMS for $C_{20}H_{24}N_3O_2$ $[M+H]^+$ calcd. 338.1869, found 338.1876.

Preparation of **S33**

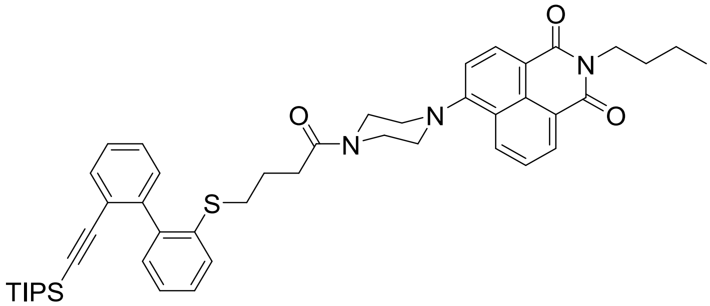

Triethylamine (0.06 mL, 0.40 mmol) was added to the mixture of **7** (89 mg, 0.27 mmol), **S11** (60 mg, 0.13 mmol), EDC (51 mg, 0.27 mmol) and HOBt (36 mg, 0.27 mmol) in anhydrous DCM (3 mL) under argon. The reaction mixture was stirred overnight at room temp. To workup, the mixture was diluted with DCM (10 mL) and washed with H_2O (10 mL). Then the organic layer was washed by brine, dried over Na_2SO_4 and concentrated under vacuum. Flash column chromatography (30% EtOAc/Hex) was used to purify the desired compound **S33** (50 mg, 49%). ^{1}H NMR ($CDCl_3$, 400 MHz): δ 8.61 (dd, J = 7.3, 1.2 Hz, 1H), 8.53 (d, J = 7.9 Hz, 1H), 8.40 (dd, J = 8.4, 1.2 Hz, 1H), 7.74 (dd, J = 8.4, 7.2 Hz, 1H), 7.62 – 7.54 (m, 1H), 7.49 – 7.42 (m, 1H), 7.37 – 7.26 (m, 5H), 7.24 – 7.20 (m, 1H), 7.18 (d, J = 8.1 Hz, 1H), 4.21 – 4.13 (m, 2H), 3.96 – 3.80 (m, 2H), 3.68 – 3.55 (m, 2H), 3.22 – 3.06 (m, 4H), 2.85 (t, J = 6.5 Hz, 2H), 2.48 – 2.23 (m, 2H), 1.89 (q, J = 7.0 Hz, 2H), 1.77 – 1.67 (m, 2H), 1.53 – 1.36 (m, 2H), 0.97 (t, J = 7.3 Hz, 3H), 0.92 (s, 21H). ^{13}C NMR ($CDCl_3$, 101 MHz): δ 171.13, 164.51, 164.06, 155.24, 143.93, 142.38,

134.93, 132.72, 132.47, 131.36, 130.82, 130.29, 129.96, 129.84, 129.82, 128.32, 127.87, 127.36, 126.41, 126.21, 126.03, 123.62, 123.52, 117.82, 115.49, 105.69, 94.36, 53.22, 53.17, 45.69, 41.79, 40.29, 33.72, 30.91, 30.40, 23.79, 20.54, 18.66, 14.00, 11.30. HRMS for $C_{47}H_{57}N_3NaO_3SSi$ $[M+Na]^+$ calcd. 794.3788, found 794.3788.

Preparation of **S34**

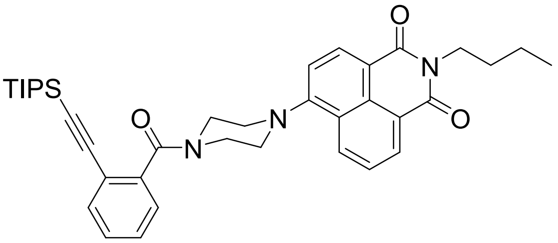

Triethylamine (0.14 mL, 0.99 mmol) was added to the mixture of **7** (112 mg, 0.33 mmol), **S30** (100 mg, 0.33 mmol), EDC (76 mg, 0.40 mmol) and HOBt (54 mg, 0.40 mmol) in anhydrous DMF (3 mL) under argon. The reaction mixture was stirred overnight at room temp. To workup, the mixture was diluted with EtOAc (10 mL) and washed with H_2O (10 mL). Then the organic layer was washed by brine, dried over Na_2SO_4 and concentrated under vacuum. Flash column chromatography (40% EtOAc/Hex) was used to purify the desired compound **S34** (110 mg, 54%). ^{1}H NMR ($CDCl_3$, 400 MHz): δ 8.59 (dd, J = 7.3, 1.3 Hz, 1H), 8.52 (d, J = 8.0 Hz, 1H), 8.37 (dd, J = 8.4, 1.3 Hz, 1H), 7.71 (dd, J = 8.5, 7.3 Hz, 1H), 7.58 – 7.53 (m, 1H), 7.43 – 7.31 (m, 3H), 7.19 (d, J = 8.2 Hz, 1H), 4.21 – 4.14 (m, 2H), 4.00 – 3.81 (m, 1H), 3.75 – 3.49 (m, 2H), 3.48 – 3.19 (m, 4H), 3.12 – 2.98 (m, 1H), 1.76 – 1.65 (m, 2H), 1.50 – 1.37 (m, 2H), 1.15 (s, 21H), 0.97 (t, J = 7.3 Hz, 3H). ^{13}C NMR ($CDCl_3$, 101 MHz): δ 168.95, 164.51, 164.08, 155.23, 138.86, 133.44, 132.43, 131.37, 129.95, 129.78, 129.07, 129.03, 126.52, 126.40, 126.22, 123.60, 120.58, 117.81, 115.49, 104.08, 95.29, 53.44, 52.89, 47.10, 41.84, 40.30, 30.39, 20.54, 18.92, 14.02, 11.50. HRMS for $C_{38}H_{47}N_3NaO_3Si$ $[M+Na]^+$ calcd. 644.3284, found 644.3286.

Preparation of **8a**

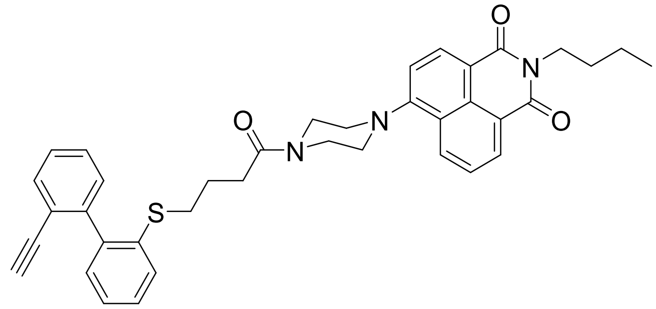

Compound **S33** (50 mg, 0.06 mmol) was dissolved in anhydrous THF (2 mL). TBAF (0.10 mL of 1.0 M solution in THF, 0.10 mmol) was then added and the mixture was stirred for 4 hr at room temp under argon. The reaction was quenched with saturated NH_4Cl solution and extracted by EtOAc. The organic layer was then dried over Na_2SO_4 and concentrated under vacuum. Flash column chromatography (40% EtOAc/Hex) was used to purify the desired compound **8a** (16 mg, 40%). ^{1}H NMR ($CDCl_3$, 400 MHz): δ 8.61 (dd, J = 7.3, 1.3 Hz, 1H), 8.53 (d, J = 8.0 Hz, 1H), 8.40 (dd, J = 8.5, 1.4 Hz, 1H), 7.76 – 7.71 (m, 1H), 7.59 (dd, J = 7.6, 1.7 Hz, 1H), 7.50 (d, J = 7.8 Hz, 1H), 7.42 – 7.28 (m, 4H), 7.26 (s, 2H), 7.18 (d, J = 8.1 Hz, 1H), 4.21 – 4.15 (m, 2H), 3.95 – 3.83 (m, 2H), 3.68 – 3.61 (m, 2H), 3.25 – 3.05 (m, 4H), 2.93 (s, 1H), 2.89 (q, J = 6.6 Hz, 2H), 2.39 (t, J = 7.2 Hz, 2H), 1.91 (p, J = 7.6, 7.2 Hz, 2H), 1.74 – 1.68 (m, 2H), 1.51 – 1.38 (m, 2H), 0.97 (t, J = 7.4 Hz, 3H). ^{13}C NMR ($CDCl_3$, 101 MHz): δ 171.13, 164.51, 164.06, 155.21, 143.99, 141.81, 134.98, 132.99, 132.47, 131.36, 130.66, 130.40, 129.95, 129.84, 129.49, 128.53, 128.50, 127.55, 126.40, 126.22, 125.91, 123.61, 121.99, 117.82, 115.50, 82.66, 80.53, 53.23, 53.11, 45.69, 41.81, 40.30, 33.50, 31.19, 30.40, 23.88, 20.54, 14.01. HRMS for $C_{38}H_{37}N_3NaO_3S$ $[M+Na]^+$ calcd. 638.2453, found 638.2458.

Preparation of **8b**

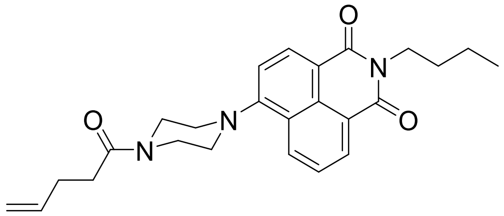

Triethylamine (0.12 mL, 0.89 mmol) was added to the mixture of **7** (100 mg, 0.30 mmol), 4-Pentenoic acid (0.06 mL, 0.59 mmol), EDC (57 mg, 0.30 mmol) and HOBt (40 mg, 0.30 mmol) in anhydrous DCM (3 mL) under argon. The reaction mixture was stirred for 1 hr at room temp. To workup, the mixture was diluted with DCM (10 mL) and washed with H_2O (10 mL). Then the organic layer was washed by brine, dried over Na_2SO_4 and concentrated under vacuum. Flash column chromatography (40% EtOAc/Hex) was used to purify the desired compound **8b** (57 mg, 46%). ^{1}H NMR ($CDCl_3$, 400 MHz): δ 8.60 (dd, J = 7.3, 1.3 Hz, 1H), 8.52 (d, J = 7.9 Hz, 1H), 8.42 (dd, J = 8.4, 1.3 Hz, 1H), 7.73 (dd, J = 8.5, 7.3 Hz, 1H), 7.22 (d, J = 8.1 Hz, 1H), 5.97 – 5.82 (m, 1H), 5.10 (dd, J = 17.2, 1.7 Hz, 1H), 5.03 (dd, J = 10.2, 1.8 Hz, 1H), 4.21 – 4.13 (m, 2H), 4.03 – 3.89 (m, 2H), 3.86 – 3.72 (m, 2H), 3.31 – 3.14 (m, 4H), 2.55 – 2.49 (m, 2H), 2.49 – 2.43 (m, 2H), 1.76 – 1.64 (m, 2H), 1.51 – 1.38 (m, 2H), 0.97 (t, J = 7.3 Hz, 3H). ^{13}C NMR ($CDCl_3$, 101 MHz): δ 171.23, 164.51, 164.06, 155.20, 137.41, 132.48, 131.37, 129.96, 129.84, 126.43, 126.25, 123.62, 117.88, 115.65, 115.52, 53.31, 53.24, 45.84, 41.89, 40.30, 32.72, 30.40, 29.39, 20.54, 14.00. HRMS for $C_{25}H_{30}N_3O_3$ $[M+H]^+$ calcd. 420.2287, found 420.2284.

Preparation of **8c**

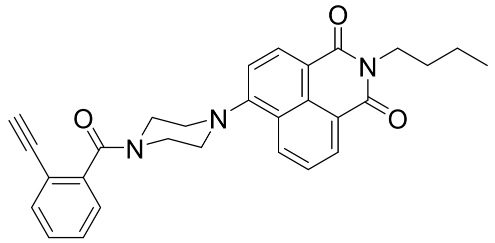

Compound **S34** (200 mg, 0.33 mmol) was dissolved in anhydrous THF (3 mL). TBAF (0.83 mL of 1.0 M solution in THF, 0.83 mmol) was then added and the mixture was stirred for 1 hr at room temp under argon. The reaction was quenched with saturated NH_4Cl solution and extracted by EtOAc. The organic layer was then dried over Na_2SO_4 and concentrated under vacuum. Flash column chromatography (40% EtOAc/Hex) was used to purify the desired compound **8c** (122 mg, 82%). ^{1}H NMR ($CDCl_3$, 400 MHz): δ 8.60 (dd, J = 7.3, 1.3 Hz, 1H), 8.52 (d, J = 8.0 Hz, 1H), 8.41 (dd, J = 8.5, 1.3 Hz, 1H), 7.72 (dd, J = 8.5, 7.3 Hz, 1H), 7.61 – 7.55 (m, 1H), 7.49 – 7.34 (m, 3H), 7.22 (d, J = 8.0 Hz, 1H), 4.21 – 4.14 (m, 2H), 3.72 – 3.54 (m, 2H), 3.39 – 3.31 (m, 2H), 3.28 (s, 1H), 3.23 – 3.11 (m, 2H), 1.76 – 1.64 (m, 2H), 1.51 – 1.37 (m, 2H), 0.97 (t, J = 7.3 Hz, 3H). ^{13}C NMR ($CDCl_3$, 101 MHz): δ 164.49, 155.20, 154.74, 149.72, 142.95, 139.34, 134.09, 133.21, 132.47, 131.39, 129.93, 129.82, 129.57, 129.25, 126.57, 126.37, 126.25, 119.22, 115.50, 81.41, 78.90, 53.34, 53.06, 47.23, 41.99, 40.30, 30.39, 20.54, 14.02. HRMS for $C_{29}H_{27}N_3NaO_3$ $[M+Na]^+$ calcd. 488.1950, found 488.1952.

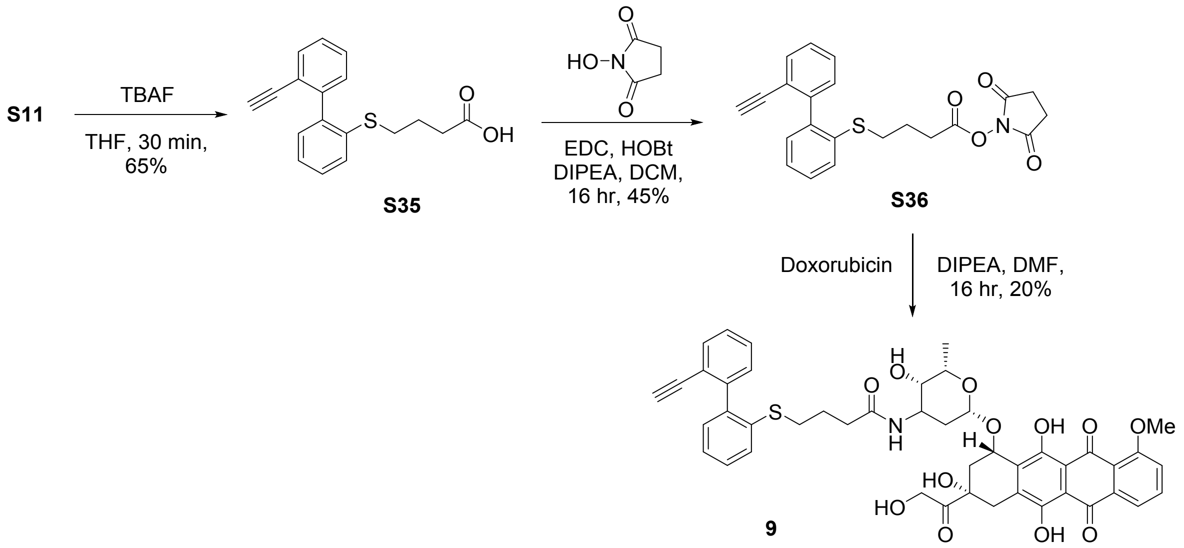

Scheme S7. Synthesis of prodrug **9**.

Preparation of **S35**

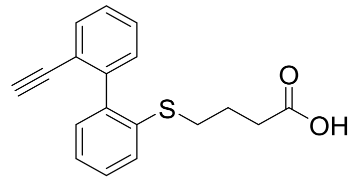

Compound **S11** (47 mg, 0.104 mmol) was dissolved in anhydrous THF (1 mL). TBAF (0.26 mL of 1.0 M solution in THF, 0.26 mmol) was then added and the mixture was stirred for 30 min at room temp under argon. The reaction was then quenched with saturated NH_4Cl solution and extracted by EtOAc. The organic layer was then washed with H_2O, brine, dried over Na_2SO_4, and concentrated under vacuum. Flash column chromatography (20% EtOAc/Hex) was used to purify the desired compound **S35** (20 mg, 65%). ^{1}H NMR ($CDCl_3$, 400 MHz): δ 7.61 (dd, J = 7.3, 1.8 Hz, 1H), 7.49 – 7.44 (m, 1H), 7.43 – 7.31 (m, 3H), 7.30 – 7.27 (m, 2H), 7.26 – 7.25 (m, 1H), 2.93 (s, 1H), 2.78 (t, J = 7.0 Hz, 2H), 2.36 (td, J = 7.2, 1.1 Hz, 2H), 1.82 (pd, J = 7.1, 1.6 Hz, 2H). ^{13}C NMR ($CDCl_3$, 101 MHz): δ 178.31, 133.04, 130.63, 130.20, 130.14, 129.70, 128.57, 128.55, 128.45, 127.68, 127.64, 127.55, 126.06, 82.36, 80.42, 33.16, 32.47, 23.81. HRMS for $C_{18}H_{15}O_2S$ $[M-H]^+$ calcd. 295.0793, found 295.0798.

Preparation of **S36**

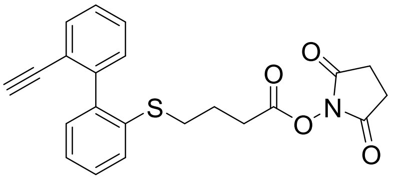

Compound **S35** (20 mg, 0.067 mmol), N-Hydroxysuccinimide (12 mg, 0.101 mmol), EDC (26 mg, 0.135 mmol), HOBt (18 mg, 0.135 mmol) were first dissolved in DCM (2 mL) under argon. Following addition of DIPEA (0.04 mL, 0.270 mmol), the mixture was stirred for 16 hr at room temp. To workup, the mixture was first diluted with DCM. The organic mixture was then washed with H_2O, dried over Na_2SO_4, and concentrated under vacuum. Flash column chromatography (50% EtOAc/Hex) was used to purify the desired compound **S36** (12 mg, 45%). ^{1}H NMR ($CDCl_3$, 400 MHz): δ 7.62 (dd, J = 7.4, 1.8 Hz, 1H), 7.51 – 7.46 (m, 1H), 7.43 – 7.39 (m, 1H), 7.38 – 7.33 (m, 2H), 7.31 – 7.26 (m, 3H), 3.00 (s, 1H), 2.88 – 2.76 (m, 6H), 2.68 – 2.56 (m, 2H), 1.98 – 1.87 (m, 2H). ^{13}C NMR ($CDCl_3$, 101 MHz): δ 177.15, 133.06, 130.64, 130.20, 130.16, 129.96, 128.65, 128.55, 128.45, 127.70, 127.62, 127.59, 126.28, 82.15, 80.65, 32.90, 29.72, 25.71, 23.78. HRMS for $C_{22}H_{19}NNaO_4S$ $[M+Na]^+$ calcd. 416.0932, found 416.0934.

Preparation of **9**

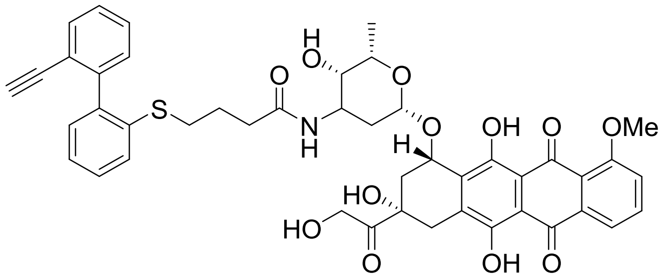

Compound **S36** (12 mg, 0.030 mmol) and doxorubicin HCl (18 mg, 0.034 mmol) were first dissolved in DMF (1 ml) under argon. Following addition of DIPEA (0.02 ml, 0.091 mmol), the mixture was stirred for 16 hr at room temp. To workup, the mixture was first diluted with ethyl acetate. The organic mixture was then washed with H_2O, brine, dried over Na_2SO_4, and concentrated under vacuum. Flash column chromatography (5% MeOH/DCM) was used to purify the desired compound **9** (5mg, 20%). ^{1}H NMR ($CDCl_3$, 400 MHz): δ 14.00 (s, 1H), 13.27 (s, 1H), 8.06 (dd, *J* = 7.8, 1.0 Hz, 1H), 7.83 – 7.76 (m, 1H), 7.62 – 7.58 (m, 1H), 7.49 – 7.27 (m, 7H), 7.25 – 7.22 (m, 2H), 5.67 (d, *J* = 8.7 Hz, 1H), 5.48 (s, 1H), 5.29 (s, 1H), 4.79 – 4.71 (m, 2H), 4.51 (s, 1H), 4.14 (d, *J* = 7.0 Hz, 1H), 4.09 (s, 3H), 3.58 (d, 1H), 3.30 (dd, *J* = 18.8 Hz, 1H), 3.08 – 2.95 (m, 3H), 2.91 (d, *J* = 18.1 Hz, 1H), 2.80 – 2.71 (m, 2H), 2.41 – 2.29 (m, 2H), 2.17 (dd, *J* = 14.8, 4.0 Hz, 1H), 2.11 (t, *J* = 7.3 Hz, 2H), 1.86 – 1.75 (m, 4H), 1.29 – 1.25 (m, 3H). ^{13}C NMR ($CDCl_3$, 101 MHz): δ 214.06, 187.37, 186.93, 171.61, 161.23, 156.34, 155.87, 135.95, 135.72, 133.75, 133.06, 130.61, 130.34, 128.55, 128.48, 127.63, 125.89, 120.04, 118.58, 111.61, 100.82, 80.55, 69.85, 69.66, 67.31, 65.72, 56.85, 45.16, 35.85, 34.86, 34.18, 33.35, 30.05, 24.44, 16.98, 1.17, 0.14. HRMS for $C_{45}H_{43}NNaO_{12}S$ $[M+Na]^+$ calcd. 844.2404, found 844.2406.

1.3 Preparation of Gold Catalysts

Catalysts **Au1-Au2**, **Au5-Au9** were purchased from commercial sources. Certain catalysts were synthesized according to literature procedures, such as **Au3.**[2] Catalysts **Au4** and **Au10** were prepared as outlined in Scheme S8.

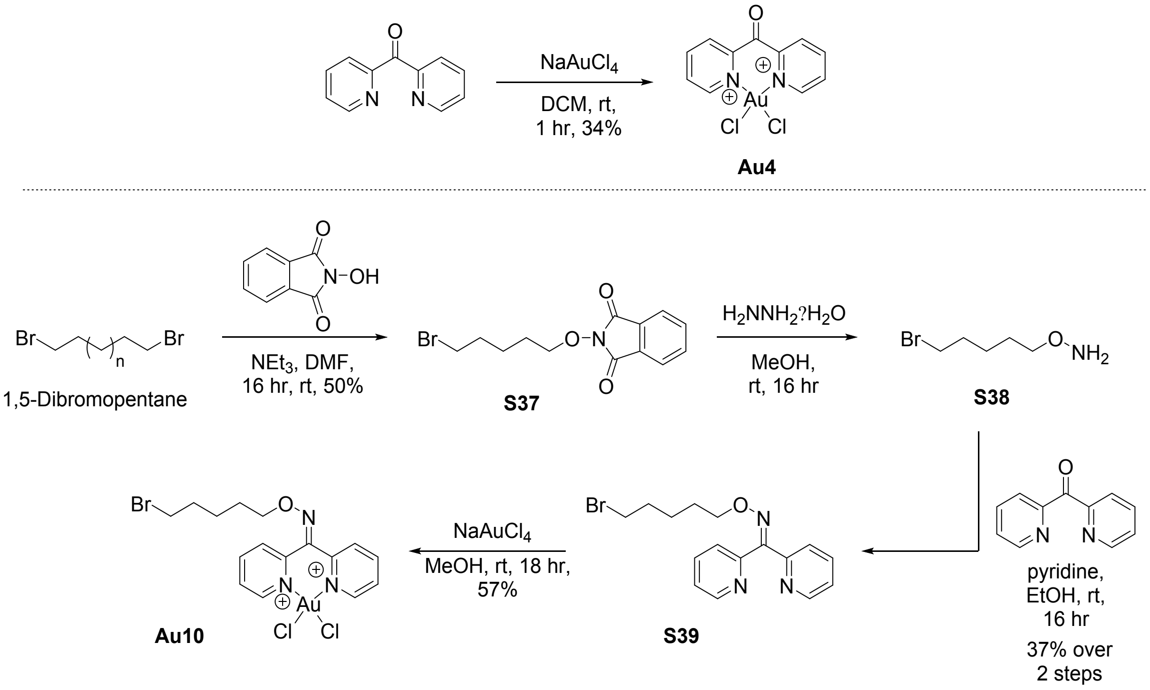

Scheme S8. Synthesis of gold catalysts **Au4** and **Au10**.

Preparation of **Au4**

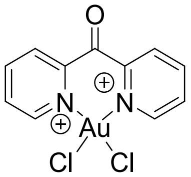

Sodium tetrachloroaurate dihydrate (50 mg, 0.126 mmol) was added to a solution of di(2-pyridyl) ketone (24 mg, 0.130 mmol) dissolved in DCM (3 ml). After 1 hr of stirring at room temp, the reaction mixture was concentrated. To crash out the product, hexane was then added. The solid was then filtered, washed with ether, and then collected by eluting with acetone. Removal of the solvent can then give the desired compound **Au4** (43 mg, 34%). ^{1}H NMR (DMSO-d_6, 400 MHz): δ 8.68 (dt, J = 4.8, 1.4 Hz, 1H), 8.51 (d, J = 3.6 Hz, 1H), 8.03 (dd, J = 7.7, 1.6 Hz, 1H), 7.97 (dt, J = 7.8, 1.3 Hz, 2H), 7.86 (d, J = 7.5 Hz, 1H), 7.64 (ddd, J = 7.5, 4.8, 1.4 Hz, 1H), 7.49 – 7.43 (m, 1H). ^{13}C NMR (DMSO-d_6, 101 MHz): δ 153.23, 148.46, 138.18, 127.21, 124.91. HRMS for $C_{11}H_8AuCl_2N_2O$ calcd. 450.9674, found 450.9680.

Preparation of **S37**

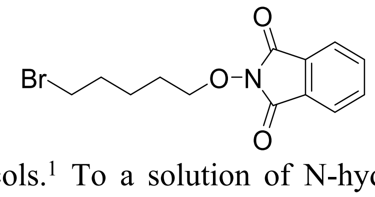

Synthesis followed literature protocols.[1] To a solution of N-hydroxy-phthalimide (1.0 g, 6.13 mmol) dissolved in DMF (4 mL), reagents 1,5-dibromopentane (1 mL, 7.34 mmol) and triethylamine (2.3 mL, 16.5 mmol) were added. The reaction mixture was then stirred for 18 hr at room temp. The red precipitate formed was then filtered off, followed by dilution of the filtrate with DCM (50 mL). After washing the organic solution with water (3×50 mL), the solvent was removed under vacuum. The crude product was purified by column chromatography on silica gel (Ethyl Acetate/Hexane= 1/9) to give **S37** as a white

solid (495 mg, 50%). ^{1}H NMR ($CDCl_3$, 400 MHz): δ 7.81 (dd, J = 5.5, 3.0 Hz, 2H), 7.74 (dd, J = 5.4, 3.1 Hz, 2H), 4.19 (t, J = 6.4 Hz, 2H), 3.42 (t, J = 6.8 Hz, 2H), 1.93 (dt, J = 14.5, 6.9 Hz, 2H), 1.84 – 1.75 (m, 2H), 1.70 – 1.60 (m, 2H). ^{13}C NMR ($CDCl_3$, 100 MHz): δ 163.01, 133.89 (2 C), 128.24, 122.88 (2 C), 77.47, 32.92, 31.72, 26.70, 23.69. HRMS for $C_{13}H_{14}BrNNaO_3$ $[M+Na]^+$ calcd. 334.0057, found 334.0055.

Preparation of **S39**

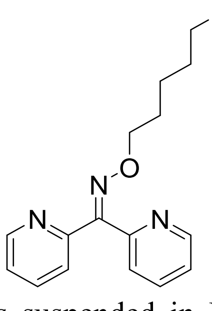

Compound **S37** (50 mg, 0.16 mmol) was suspended in MeOH (2 mL), followed by the addition of hydrazine (0.02 mL, 0.24 mmol). The mixture was then stirred for 18 hr at room temp. To workup, the mixture was diluted with EtOAc (10 mL), then washed with water (20 mL) and brine (20 mL). The organic layer was then dried over sodium sulfate, followed by the removal of the solvent under vacuum to produce **S38** as crude. To a solution of crude **S38** (20 mg, 0.11 mmol) and di(2-pyridyl) ketone (16 mg, 0.09 mmol) in EtOH (2 mL), pyridine (27 μL, 0.33 mmol) was added. The reaction mixture was then stirred overnight at room temp. To workup, the solvent and pyridine were removed under vacuum. The crude was purified by PTLC (70% EtOAc/Hexane) to give **S39** (7 mg, 13%). ^{1}H NMR ($CDCl_3$, 400 MHz): δ 8.64 (dd, J = 58.5, 5.1 Hz, 2H), 7.80 (dt, J = 8.0, 3.8 Hz, 2H), 7.70 (t, J = 7.7 Hz, 1H), 7.57 (d, J = 7.9 Hz, 1H), 7.35 – 7.28 (m, 1H), 7.28 – 7.19 (m, 1H), 4.25 (t, J = 6.5 Hz, 2H), 3.38 (t, J = 6.7 Hz, 2H), 1.85 (p, J = 6.9 Hz, 2H), 1.78 – 1.63 (m, 2H), 1.50 (q, J = 8.3 Hz, 2H). ^{13}C NMR ($CDCl_3$, 100 MHz): δ 155.83, 154.31, 152.28, 149.74, 149.56, 136.46, 136.04, 125.60, 123.69, 123.48, 122.66, 75.09, 33.83, 32.52, 28.31, 24.76. HRMS for $C_{16}H_{19}BrNO_3$ $[M+H]^+$ calcd. 348.0712, found 348.0718.

Preparation of **Au10**

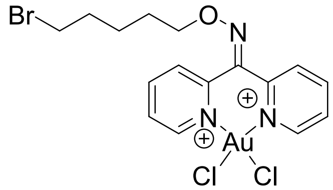

Sodium tetrachloroaurate dihydrate (8 mg, 20 μmol) was added to a solution of crude **S39** (7 mg, 20 μmol) dissolved in MeOH (2 mL). After overnight stirring at room temp, the reaction mixture was concentrated under vacuum and crash out the product by adding Et_2O (7 mL). The collected solid was then filtered, washed with Et_2O, and then collected to give compound **Au10** as a yellow solid (7 mg, 57%). ^{1}H NMR (DMSO-d_6, 400 MHz): δ 8.68 (d, J = 4.9 Hz, 1H), 8.50 (d, J = 4.9 Hz, 1H), 8.04 (td, J = 7.8, 1.8 Hz, 1H), 7.98 – 7.87 (m, 2H), 7.66 (d, J = 7.9 Hz, 1H), 7.58 – 7.51 (m, 1H), 7.47 (ddd, J = 6.8, 4.9, 1.6 Hz, 1H), 4.20 (t, J = 6.5 Hz, 2H), 3.51 (t, J = 6.6 Hz, 2H), 1.85 – 1.74 (m, 2H), 1.73 – 1.61 (m, 2H), 1.41 (tt, J = 9.9, 6.3 Hz, 2H). ^{13}C NMR (DMSO-d_6, 101 MHz): δ 148.24, 147.31, 139.98, 139.09, 126.73, 125.29, 122.66, 75.41, 45.61, 35.41, 32.02, 27.81. HRMS for $C_{16}H_{18}AuBrClN_3O$ $[M-Cl]^+$ calcd. 578.9976, found 578.9984.

2. Reactivity Studies

2.1 HPLC Methods and Standard Curves

To determine yields from reverse-phase HPLC analysis, calibration curves were first constructed using product standards of known amounts with the indicated elution method (Table S1). To do this, a Shimadzu system (Japan) equipped with two LC-40D solvent pumps, a SPD-40 UV/Visible detector, a RF-20Axs fluorescence detector, and a SIL-40 autosampler were used. The attached column was an analytical 4.6 × 250 mm Cosmosil 5C_{18}-AR-300 from Nacalai Tesque (Japan). Samples were eluted using a combination of mobile phases A (H_2O with 0.1% TFA), and B (acetonitrile with 0.1% TFA). The detector was set to 214 and 254 nm. After running reaction mixtures into the HPLC, product identification was determined by MS analysis of collected peaks and retention time comparison to known product standards. Following peak integration, yields can be calculated based on the product calibration curve (Figure S1-S6).

Table S1. Gradient profiles for HPLC studies

	**Flow rate (ml/min)**	**Time (min)**	**%A (H_2O + 0.1% TFA)**	**%B (ACN + 0.1% TFA)**
Method 1	1.0	0	95	5
C_{18} column		5	95	5
		25	10	90
		35	10	90
		37	95	5
		40	95	5
Method 2	1.0	0	95	5
C_{18} column		10	95	5
		20	10	90
		30	10	90
		35	95	5
		40	95	5
Method 3	1.0	0	95	5
C_{18} column		5	95	5
		20	10	90
		30	10	90
		32	95	5
		40	95	5

Product Structure	Calibration Curve

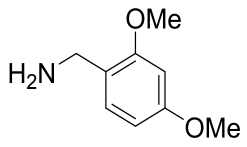

6a

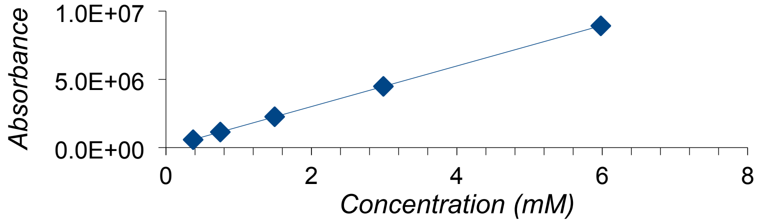

HPLC Method: 1

$R^2 = 0.9990$		Value
	Slope	1,487,065
	Intercept	31,972

HPLC trace:

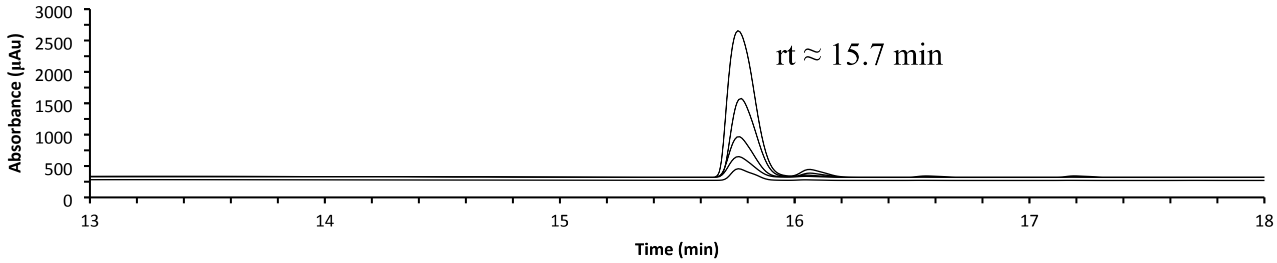

Figure S1. HPLC calibration curve for released product **6a**.

Product Structure	Calibration Curve

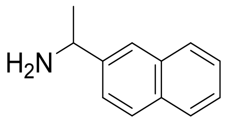

6f

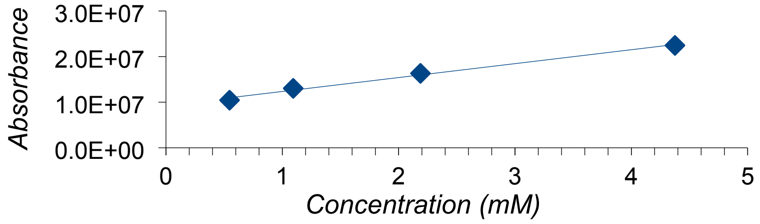

HPLC Method: 2

$R^2 = 0.9900$		Value
	Slope	3,050,876
	Intercept	9,300,800

HPLC trace:

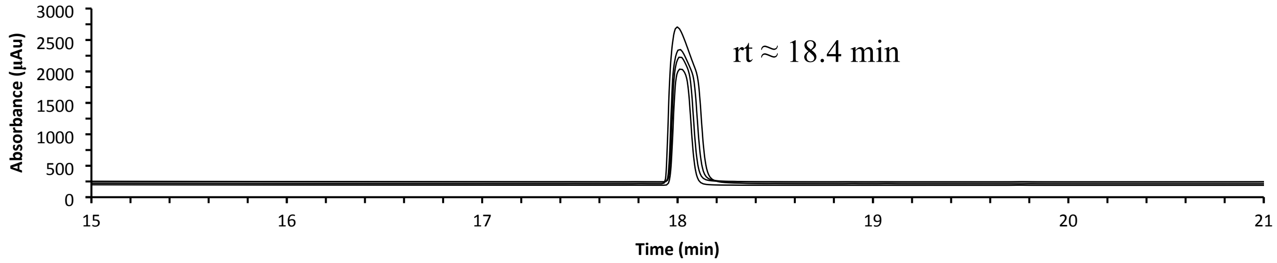

Figure S2. HPLC calibration curve of released product **6f**.

Product Structure	Calibration Curve

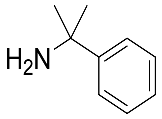

6g

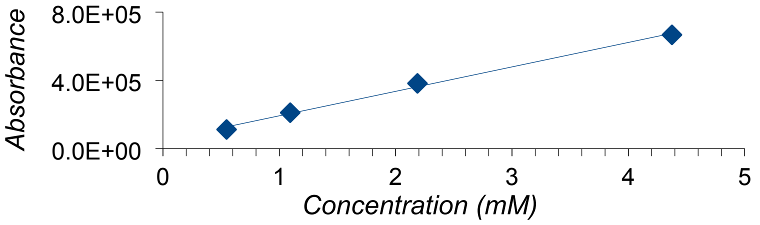

HPLC Method: 2

R^2 = 0.9959	Value
Slope	143,282
Intercept	49,083

HPLC trace:

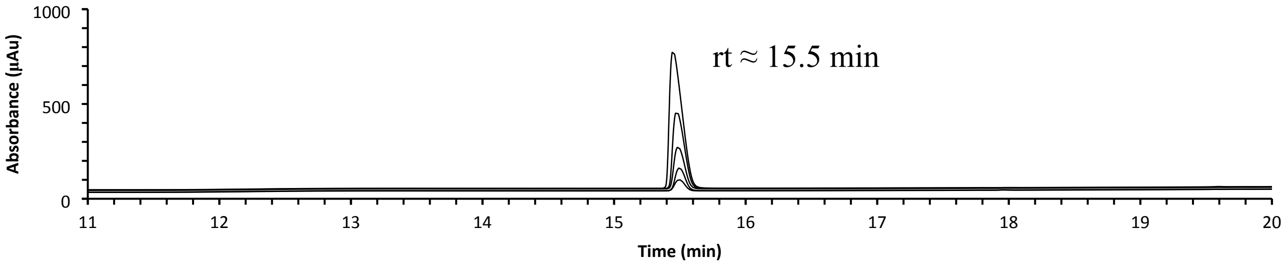

Figure S3. HPLC calibration curve of released product **6g**.

Product Structure	Calibration Curve

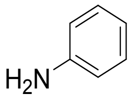

6h

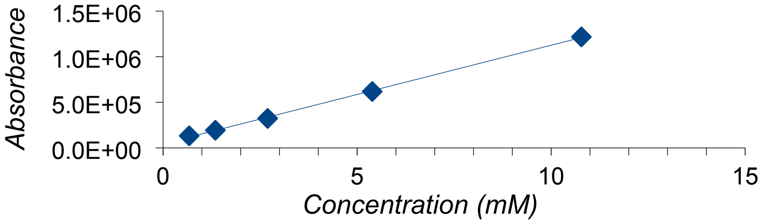

HPLC Method: 3

R^2 = 0.9993	Value
Slope	108,034
Intercept	45,896

HPLC trace:

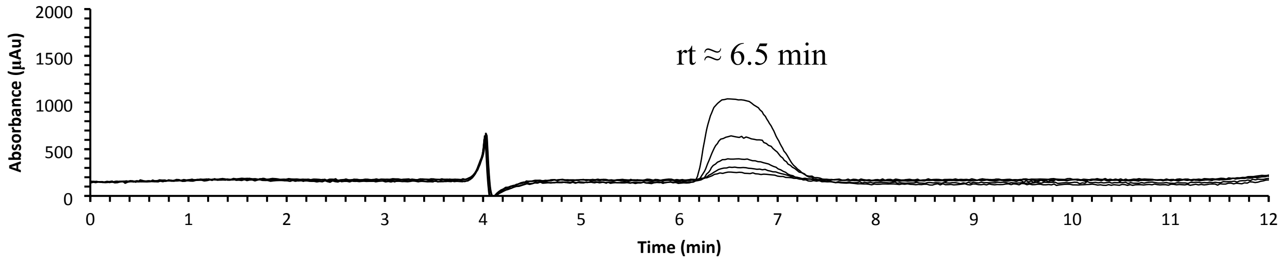

Figure S4. HPLC calibration curve of released product **6h**.

Product Structure	Calibration Curve

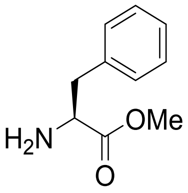

6i

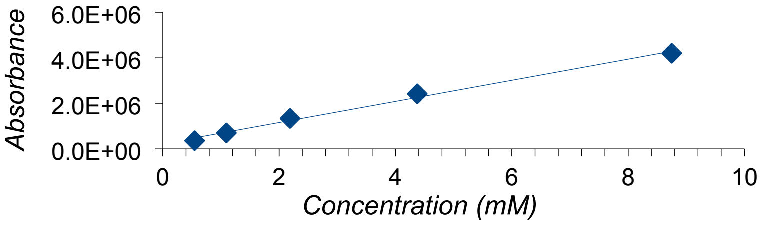

HPLC Method: 2

R^2 = 0.9939		Value
	Slope	464,192
	Intercept	227,431

HPLC trace:

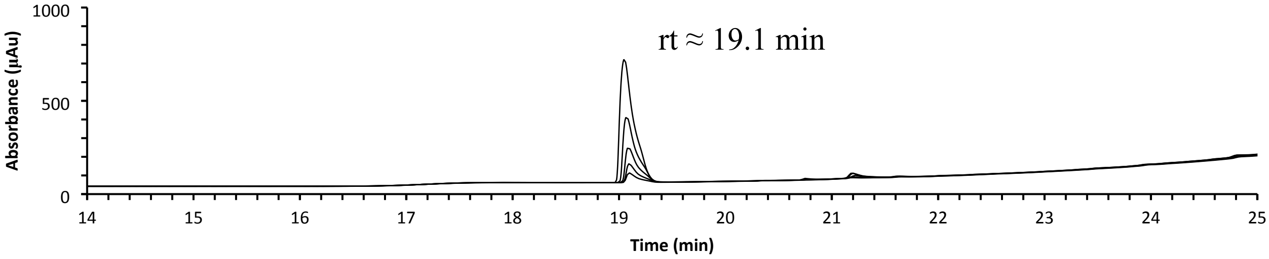

Figure S5. HPLC calibration curve of released product **6i**.

Product Structure	Calibration Curve

Doxorubicin

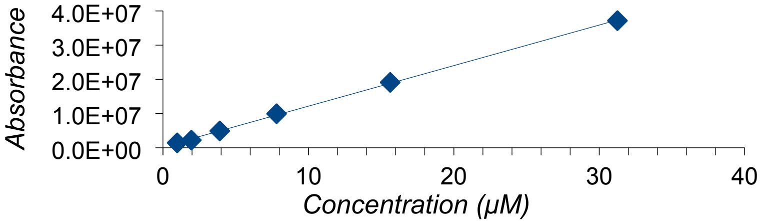

HPLC Method: 1

R^2 = 0.9996		Value
	Slope	1,186,015
	Intercept	302,118

HPLC trace:

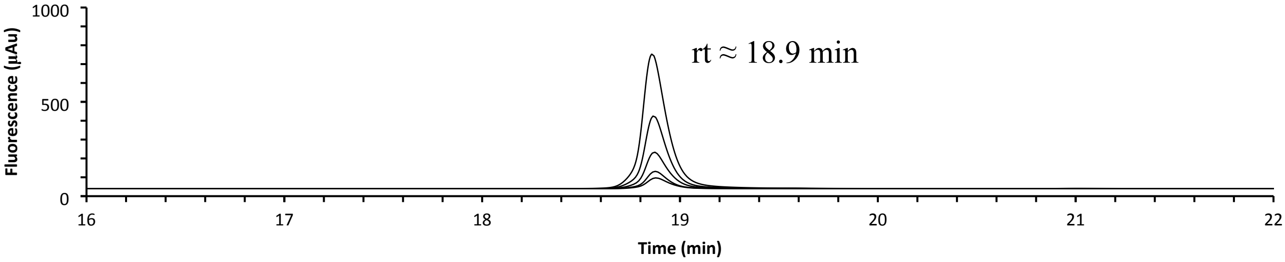

Figure S6. HPLC calibration curve of doxorubicin

2.2 Hydrothiolation Substrate Screening

To test reactivity, **1** (1 mg, 3.40 μmol) was dissolved in 25 μl of organic solvent. Depending on the situation, transition metal catalysts (mol% adjusted as indicated) were dissolved in either 25 μl of either organic solvent or PBS buffer. The substrate and catalyst were then mixed, along with sufficient solvent to make up a total volume of 100 μl (unless otherwise stated). These mixtures were then placed in a sand bath at an adjusted temperature of 37°C without stirring. To workup, mixtures were quenched with 1 mM dodecanethiol in ACN (100 μl) and 1.0M HCl (50 μl). The solutions were then filtered and injected (10 μl) onto a HPLC with an autosampler. Data regarding the formation of **2** from substrate **1** is shown in Table S2.

Table S2. Gold-catalyzed hydrothiolation of compound **1**

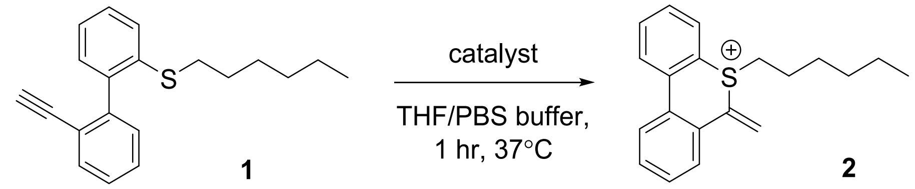

Entry	Catalyst	Mol%	Yield of **2** (%)[a]	Turnover (TON)
1	Me_2SAuCl	40	0	-
2	Me_2SAuCl	20	0	-
3	$NaAuCl_4$	40	100	2.5
4	$NaAuCl_4$	20	32	1.6
5	$PdCl_2$	40	0	-
6	$PdCl_2$	20	0	-
7	K_2PtCl_4	40	0	-
8	K_2PtCl_4	20	0	-
9	Ag_2CO_3	40	0	-
10	Ag_2CO_3	20	0	-
11	$RuCl_3$	40	0	-
12	$RuCl_3$	20	0	-
13	$CuSO_4$ + THPTA	40	0	-
14	$CuSO_4$ + THPTA	20	0	-
15	$CuSO_4$	40	0	-
16	$CuSO_4$	20	0	-

[a]Yields determined by HPLC (peak retention times compared to product standards, followed by MS analysis for confirmation, and then calculation of yields based on product standard curves). All reactions were standardized to 3.40 μmol of **1** in 100 μl of solvent.

2.3 EBB-based Model Substrate Screening

To test reactivity, substrates **3a-i** (1 mg, 1.92-2.70 μmol) were dissolved in 25 μl of organic solvent. Depending on the situation, catalysts **Au1-Au9** (mol% adjusted as indicated) were dissolved in either 25 μl of either organic solvent or PBS buffer. The substrate and catalyst were then mixed, along with sufficient solvent to make up a total volume of 100 μl. These mixtures were then placed in a sand bath at an adjusted temperature of 37°C without stirring. To workup, mixtures were quenched with 1 mM dodecanethiol in ACN (100 μl) and 1.0M HCl (50 μl). The solutions were then filtered and injected (10 μl) onto a HPLC with an autosampler. Data regarding release of **6a** from substrate **3a** is shown in Table S3. Data regarding release of **6a** and **6f-i** from derivative substrates **3a-i** are shown in Tables S4-S11.

Table S3. Gold-catalyzed reactivity of compound **3a** for the release of amine **6a**

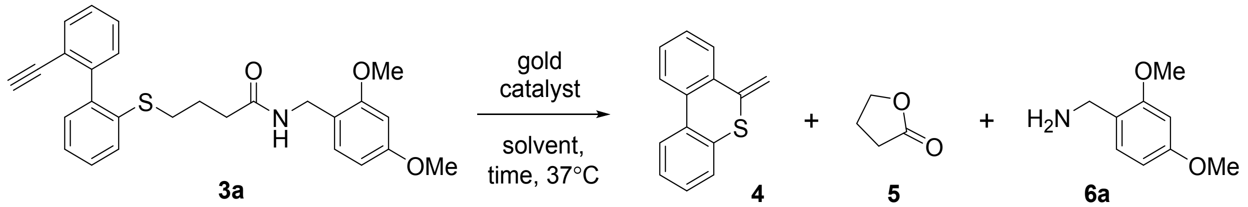

Entry	Catalyst	Mol%	Solvent	Time (hr)	Release yield of **6a** (%)[a]	Turnover (TON)
17	-	-	50% THF in PBS buffer	1	0	-
18	**Au1**	5	50% THF in PBS buffer	1	3	0.63
19	**Au1**	10	50% THF in PBS buffer	1	17	1.72
20	**Au1**	20	50% THF in PBS buffer	1	50	2.48
21	**Au1**	40	50% THF in PBS buffer	1	100	2.50
22	**Au1**	20	THF	1	13	0.64
23	**Au1**	20	Dioxane	1	0	-
24	**Au1**	20	50% Dioxane in PBS buffer	1	0	-
25	**Au1**	20	DMSO	1	0	-
26	**Au1**	20	50% DMSO in PBS buffer	1	16	0.81
27	**Au1**	20	ACN	1	5	0.27
28	**Au1**	20	50% ACN in PBS buffer	1	33	1.65
29	**Au1**	20	DMF	1	6	0.30
30	**Au1**	20	50% DMF in PBS buffer	1	89	4.43
31	**Au2**	20	THF	1	4	0.22
32	**Au2**	20	50% THF in PBS buffer	1	56	2.78
33	**Au2**	20	Dioxane	1	2	0.09
34	**Au2**	20	50% Dioxane in PBS buffer	1	33	1.65
35	**Au2**	20	DMSO	1	13	0.63
36	**Au2**	20	50% DMSO in PBS buffer	1	16	0.79
37	**Au2**	20	ACN	1	13	0.65
38	**Au2**	20	50% ACN in PBS buffer	1	27	1.33
39	**Au2**	20	DMF	1	14	0.72
40	**Au2**	20	50% DMF in PBS buffer	1	70	3.48
41	**Au3**	20	THF	1	7	0.35
42	**Au3**	20	50% THF in PBS buffer	1	16	0.78
43	**Au3**	20	Dioxane	1	0	-
44	**Au3**	20	50% Dioxane in PBS buffer	1	0	-
45	**Au3**	20	DMSO	1	0	-

Continue →

Table S3 (continued). Gold-catalyzed reactivity of compound **3a** for the release of amine **6a**

Entry	Catalyst	Mol%	Solvent	Time (hr)	Release yield of **6a** (%)[a]	Turnover (TON)
46	**Au3**	20	50% DMSO in PBS buffer	1	31	1.56
47	**Au3**	20	ACN	1	7	0.33
48	**Au3**	20	50% ACN in PBS buffer	1	37	1.84
49	**Au3**	20	DMF	1	0	-
50	**Au3**	20	50% DMF in PBS buffer	1	35	1.73
51	**Au4**	20	THF	1	12	0.62
52	**Au4**	20	50% THF in PBS buffer	1	28	1.40
53	**Au4**	20	Dioxane	1	0	-
54	**Au4**	20	50% Dioxane in PBS buffer	1	11	0.57
55	**Au4**	20	DMSO	1	3	0.16
56	**Au4**	20	50% DMSO in PBS buffer	1	18	0.92
57	**Au4**	20	ACN	1	7	0.35
58	**Au4**	20	50% ACN in PBS buffer	1	10	0.52
59	**Au4**	20	DMF	1	9	0.46
60	**Au4**	20	50% DMF in PBS buffer	1	45	2.24
61	**Au5**	20	THF	1	8	0.42
62	**Au5**	20	50% THF in PBS buffer	1	12	0.62
63	**Au5**	20	Dioxane	1	0	-
64	**Au5**	20	50% Dioxane in PBS buffer	1	0	-
65	**Au5**	20	DMSO	1	8	0.38
66	**Au5**	20	50% DMSO in PBS buffer	1	6	0.32
67	**Au5**	20	ACN	1	3	0.14
68	**Au5**	20	50% ACN in PBS buffer	1	3	0.16
69	**Au5**	20	DMF	1	11	0.57
70	**Au5**	20	50% DMF in PBS buffer	1	5	0.24
71	**Au6**	20	THF	1	0	-
72	**Au6**	20	50% THF in PBS buffer	1	0	-
73	**Au6**	20	Dioxane	1	0	-
74	**Au6**	20	50% Dioxane in PBS buffer	1	0	-
75	**Au6**	20	DMSO	1	0	-
76	**Au6**	20	50% DMSO in PBS buffer	1	0	-
77	**Au6**	20	ACN	1	0	-
78	**Au6**	20	50% ACN in PBS buffer	1	0	-
79	**Au6**	20	DMF	1	0	-
80	**Au6**	20	50% DMF in PBS buffer	1	0	-
81	**Au7**	20	THF	1	0	-
82	**Au7**	20	50% THF in PBS buffer	1	0	-
83	**Au7**	20	Dioxane	1	0	-
84	**Au7**	20	50% Dioxane in PBS buffer	1	0	-
85	**Au7**	20	DMSO	1	0	-
86	**Au7**	20	50% DMSO in PBS buffer	1	0	-
87	**Au7**	20	ACN	1	0	-
88	**Au7**	20	50% ACN in PBS buffer	1	0	-
89	**Au7**	20	DMF	1	0	-
90	**Au7**	20	50% DMF in PBS buffer	1	0	-

Continue →

Table S3 (continued). Gold-catalyzed reactivity of compound **3a** for the release of amine **6a**

Entry	Catalyst	Mol%	Solvent	Time (hr)	Release yield of **6a** (%)[a]	Turnover (TON)
91	**Au8**	20	THF	1	0	-
92	**Au8**	20	50% THF in PBS buffer	1	0	-
93	**Au8**	20	Dioxane	1	0	-
94	**Au8**	20	50% Dioxane in PBS buffer	1	0	-
95	**Au8**	20	DMSO	1	0	-
96	**Au8**	20	50% DMSO in PBS buffer	1	0	-
97	**Au8**	20	ACN	1	0	-
98	**Au8**	20	50% ACN in PBS buffer	1	0	-
99	**Au8**	20	DMF	1	0	-
100	**Au8**	20	50% DMF in PBS buffer	1	0	-
101	**Au9**	20	THF	1	0	-
102	**Au9**	20	50% THF in PBS buffer	1	0	-
103	**Au9**	20	Dioxane	1	0	-
104	**Au9**	20	50% Dioxane in PBS buffer	1	0	-
105	**Au9**	20	DMSO	1	0	-
106	**Au9**	20	50% DMSO in PBS buffer	1	0	-
107	**Au9**	20	ACN	1	0	-
108	**Au9**	20	50% ACN in PBS buffer	1	0	-
109	**Au9**	20	DMF	1	0	-
110	**Au9**	20	50% DMF in PBS buffer	1	0	-

[a]Yields determined by HPLC (peak retention times compared to product standards, followed by MS analysis for confirmation, and then calculation of yields based on product standard curves). [b]Catalyst added as a suspension. All reactions were standardized to 2.24 µmol of **3a** in 100 µl of solvent.

Table S4. Gold-catalyzed reactivity of compound **3b** for the release of amine **6a**

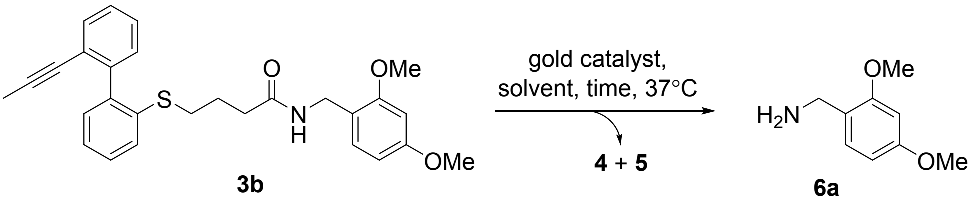

3b **6a**

Entry	Catalyst	Mol%	Solvent	Time (hr)	Yield of **6a** (%)[a]	Turnover (TON)[a]
111	-	-	50% THF in PBS buffer	1	0	-
112	**Au1**	20	50% THF in PBS buffer	1	31	1.53
113	**Au1**	20	50% DMSO in PBS buffer	1	9	0.46
114	**Au1**	20	50% DMF in PBS buffer	1	18	0.90
115	**Au2**	20	50% THF in PBS buffer	1	16	0.81
116	**Au2**	20	50% DMSO in PBS buffer	1	13	0.67
117	**Au2**	20	50% DMF in PBS buffer	1	24	1.18

[a]Yields determined by HPLC (peak retention times compared to product standards, followed by MS analysis for confirmation, and then calculation of yields based on product standard curves). All reactions were standardized to 2.18 μmol of **3b** in 100 μl of solvent.

Table S5. Gold-catalyzed reactivity of compound **3c** for the release of amine **6a**

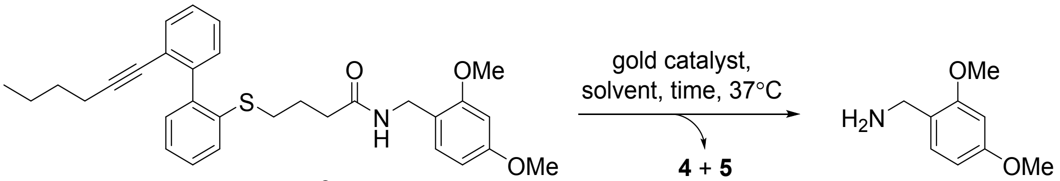

3c **6a**

Entry	Catalyst	Mol%	Solvent	Time (hr)	Yield of **6a** (%)[a]	Turnover (TON)[a]
118	-	-	50% THF in PBS buffer	1	0	-
119	**Au1**	20	50% THF in PBS buffer	1	2	0.11
120	**Au1**	20	50% DMSO in PBS buffer	1	6	0.30
121	**Au1**	20	50% DMF in PBS buffer	1	7	0.35
122	**Au2**	20	50% THF in PBS buffer	1	4	0.21
123	**Au2**	20	50% DMSO in PBS buffer	1	12	0.58
124	**Au2**	20	50% DMF in PBS buffer	1	16	0.78

[a]Yields determined by HPLC (peak retention times compared to product standards, followed by MS analysis for confirmation, and then calculation of yields based on product standard curves). All reactions were standardized to 2.00 μmol of **3c** in 100 μl of solvent.

Table S6. Gold-catalyzed reactivity of compound **3d** for the release of amine **6a**

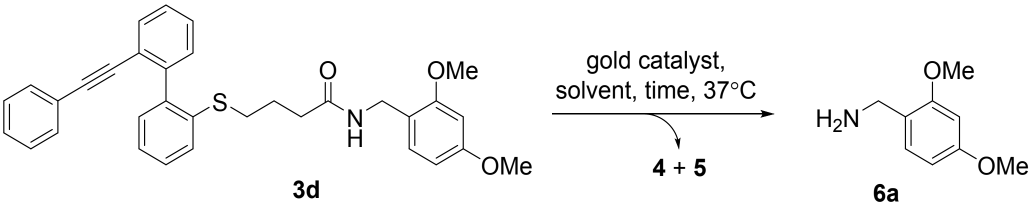

Entry	Catalyst	Mol%	Solvent	Time (hr)	Yield of **6a** (%)[a]	Turnover (TON)[a]
125	**-**	-	50% THF in PBS buffer	1	0	-
126	**Au1**	20	50% THF in PBS buffer	1	5	0.24
127	**Au1**	20	50% DMSO in PBS buffer	1	3	0.14
128	**Au1**	20	50% DMF in PBS buffer	1	12	0.58
129	**Au2**	20	50% THF in PBS buffer	1	16	0.78
130	**Au2**	20	50% DMSO in PBS buffer	1	20	1.02
131	**Au2**	20	50% DMF in PBS buffer	1	26	1.29

[a]Yields determined by HPLC (peak retention times compared to product standards, followed by MS analysis for confirmation, and then calculation of yields based on product standard curves). All reactions were standardized to 1.92 µmol of **3d** in 100 µl of solvent.

Table S7. Gold-catalyzed reactivity of compound **3e** for the release of amine **6a**

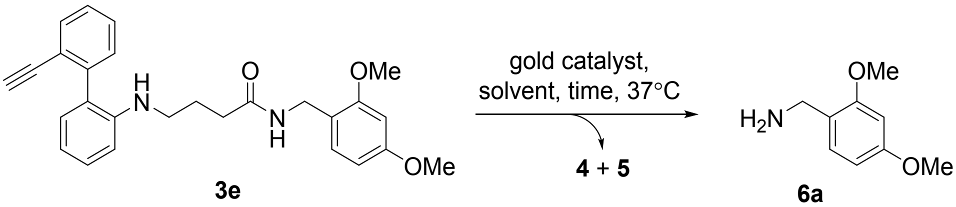

Entry	Catalyst	Mol%	Solvent	Time (hr)	Yield of **6a** (%)[a]	Turnover (TON)[a]
132	**-**	-	50% THF in PBS buffer	1	0	-
133	**Au1**	20	50% THF in PBS buffer	1	0	-
134	**Au1**	20	50% DMSO in PBS buffer	1	0	-
135	**Au1**	20	50% DMF in PBS buffer	1	0	-
136	**Au2**	20	50% THF in PBS buffer	1	0	-
137	**Au2**	20	50% DMSO in PBS buffer	1	0	-
138	**Au2**	20	50% DMF in PBS buffer	1	0	-

[a]Yields determined by HPLC (peak retention times compared to product standards, followed by MS analysis for confirmation, and then calculation of yields based on product standard curves). All reactions were standardized to 2.34 µmol of **3e** in 100 µl of solvent.

Table S8. Gold-catalyzed reactivity of compound **3f** for the release of amine **6f**

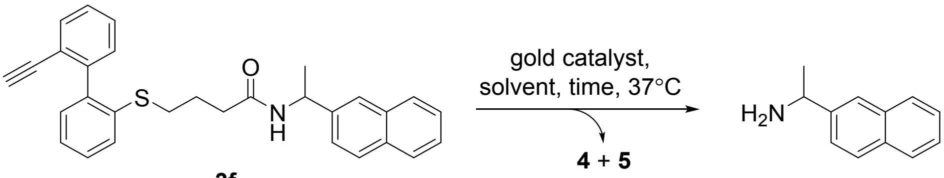

3f **6f**

Entry	Catalyst	Mol%	Solvent	Time (hr)	Yield of **6f** (%)[a]	Turnover (TON)[a]
139	-	-	50% THF in PBS buffer	1	0	-
140	**Au1**	20	50% THF in PBS buffer	1	24	1.18
141	**Au1**	20	50% DMSO in PBS buffer	1	11	0.55
142	**Au1**	20	50% DMF in PBS buffer	1	25	1.24
143	**Au2**	20	50% THF in PBS buffer	1	15	0.73
144	**Au2**	20	50% DMSO in PBS buffer	1	18	0.91
145	**Au2**	20	50% DMF in PBS buffer	1	9	0.44

[a]Yields determined by HPLC (peak retention times compared to product standards, followed by MS analysis for confirmation, and then calculation of yields based on product standard curves). All reactions were standardized to 2.23 μmol of **3f** in 100 μl of solvent.

Table S9. Gold-catalyzed reactivity of compound **3g** for the release of amine **6g**

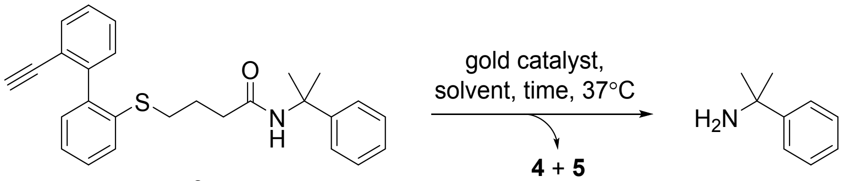

3g **6g**

Entry	Catalyst	Mol%	Solvent	Time (hr)	Yield of **6g** (%)[a]	Turnover (TON)[a]
146	-	-	50% THF in PBS buffer	1	0	-
147	**Au1**	20	50% THF in PBS buffer	1	3	0.15
148	**Au1**	20	50% DMSO in PBS buffer	1	2	0.08
149	**Au1**	20	50% DMF in PBS buffer	1	25	1.24
150	**Au2**	20	50% THF in PBS buffer	1	0	-
151	**Au2**	20	50% DMSO in PBS buffer	1	22	1.08
152	**Au2**	20	50% DMF in PBS buffer	1	5	0.24

[a]Yields determined by HPLC (peak retention times compared to product standards, followed by MS analysis for confirmation, and then calculation of yields based on product standard curves). All reactions were standardized to 2.53 μmol of **3g** in 100 μl of solvent.

Table S10. Gold-catalyzed reactivity of compound **3h** for the release of amine **6h**

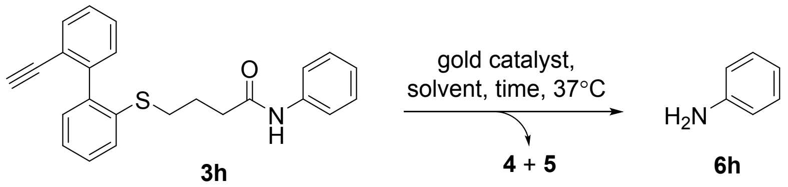

Entry	Catalyst	Mol%	Solvent	Time (hr)	Yield of **6h** (%)[a]	Turnover (TON)[a]
153	-	-	50% THF in PBS buffer	1	0	-
154	**Au1**	20	50% THF in PBS buffer	1	0	-
155	**Au1**	20	50% DMSO in PBS buffer	1	0	-
156	**Au1**	20	50% DMF in PBS buffer	1	0	-
157	**Au2**	20	50% THF in PBS buffer	1	0	-
158	**Au2**	20	50% DMSO in PBS buffer	1	0	-
159	**Au2**	20	50% DMF in PBS buffer	1	0	-

[a]Yields determined by HPLC (peak retention times compared to product standards, followed by MS analysis for confirmation, and then calculation of yields based on product standard curves). All reactions were standardized to 2.70 μmol of **3h** in 100 μl of solvent.

Table S11. Gold-catalyzed reactivity of compound **3i** for the release of amine **6i**

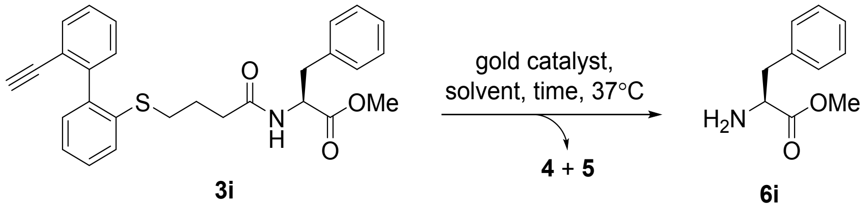

Entry	Catalyst	Mol%	Solvent	Time (hr)	Yield of **6i** (%)[a]	Turnover (TON)[a]
160	-	-	50% THF in PBS buffer	1	0	-
161	**Au1**	20	50% THF in PBS buffer	1	37	1.85
162	**Au1**	20	50% DMSO in PBS buffer	1	18	0.90
163	**Au1**	20	50% DMF in PBS buffer	1	25	1.25
164	**Au2**	20	50% THF in PBS buffer	1	36	1.78
165	**Au2**	20	50% DMSO in PBS buffer	1	10	0.50
166	**Au2**	20	50% DMF in PBS buffer	1	8	0.39

[a]Yields determined by HPLC (peak retention times compared to product standards, followed by MS analysis for confirmation, and then calculation of yields based on product standard curves). All reactions were standardized to 2.27 μmol of **3i** in 100 μl of solvent.

3. Photophysical Characterizations

3.1 Justification of Solvent Conditions

To determine the appropriate solvent system to monitor the reactivities of profluorophores **8a-c**, these compounds and **7** were dissolved in different solvent mixtures of DMF/H_2O (ranging from 10-90% of DMF in H_2O) and then analyzed for emitted fluorescence (at an excitation of wavelength of 390 nm). As expected, fluorophore **7** displayed an increase in fluorescence correlated with an increase in H_2O content (Figure S7D). This can be explained by the ability of water to protonate the free secondary amine of **7**, thereby suppressing photoinduced electron transfer (PET). On the other hand, profluorophore **8b**, which has previously been reported,[3] instead showed a decrease in fluorescence correlated with an increase in H_2O content (Figure S7B). These inverse properties of the substrate (i.e. **8b**) and product (i.e. **7**) is what has allowed effective real-time monitoring of profluorophore reactivity.

One of the aims of this study was to monitor the reactivity of profluorophore **8a** so comparisons can be made to literature equivalents (i.e. **8b** and **8c**). However, when **8a** was dissolved in different solvent mixtures of DMF/H_2O, fluorescence analysis gave unexpected results (Figure S7A). Starting from high DMF concentrations (90% to 50%), a typical trend of decreasing fluorescence was observed. However, once DMF concentrations further lowered from 50% to 10%, an unexpected increase in fluorescence was consistently recorded. This H_2O-dependent change in fluorescence was also observed with profluorophore **8c**, albeit at a lower level (Figure S7C). At very high water levels (i.e. 10% DMF in H_2O), **8c** deviated from the decreasing trend and instead showed a sudden increase in fluorescence.

To explain these observations, it is hypothesized that profluorophores **8a** and **8c** are capable of undergoing aggregation-induced emission (AIE) under aqueous conditions. A quick scan of the literature shows that many naphthalimide-based compounds have exhibited these types of properties.[4] It is likely that the bulkier and aromatic EBB- and Ayba-based protecting groups of **8a** and **8c** contribute to interactions leading to aggregation.

Considering these results, it was thus deemed impractical to conduct reactivity assays under low solvent conditions (i.e. 10% DMF in H_2O). Moving forward, it was decided that solvent conditions would be maintained at 50% DMF in H_2O.

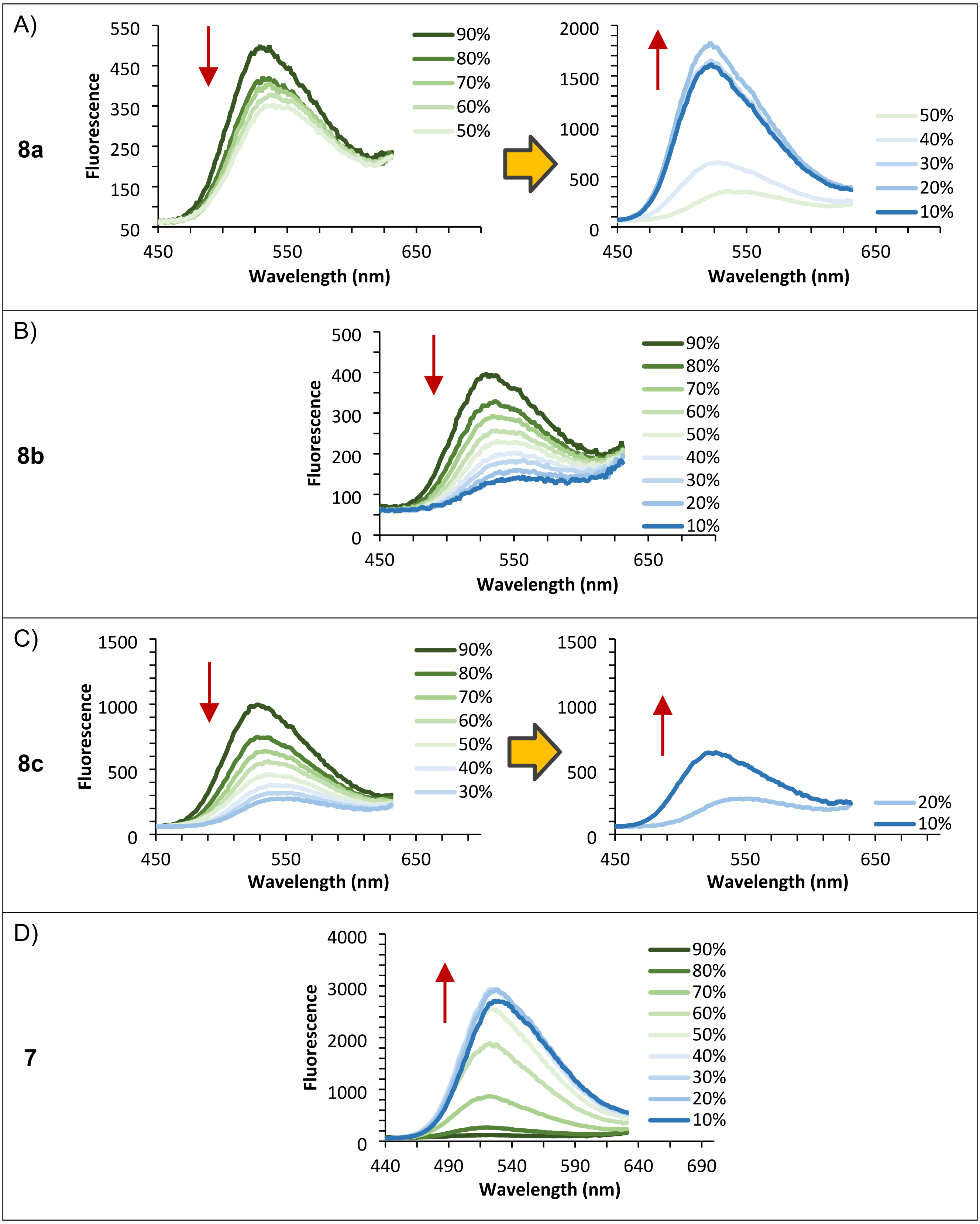

Figure S7. Effect of increasing solvent water composition to the fluorescence of compounds A) **8a**, B) **8b**, C) **8c**, and D) **7**. Each profluorophore (50 µM) was dissolved in varying DMF/H_2O solvent conditions, followed by the acquisition of the fluorescence spectra obtained after excitation at 390 nm. The red arrows are used to highlight either the increase or decrease of observed fluorescence with increasing water composition in the solvent.

3.2 Quantum Yield Determination

The photophysical properties and quantum yields of **8a-c** and their released fluorophore **7** were obtained. During quantum yield measurements, quinine sulfate was used as a control. Extinction coefficients and excitation wavelengths were first determined by plotting a standard Beer-Lambert plot. Reported values were determined from the measurement of three different concentrations. Compounds **8a-c** and **7** were dissolved in 50% DMF in H_2O, while quinine sulfate was dissolved in 1N H_2SO_4. Absorbance spectra, excitation wavelengths, and calculated extinction coefficients are shown in Figure S8.

$$A = \varepsilon c l$$

Note: absorbance (A), extinction coefficient (ε), concentration (c), path length (l).

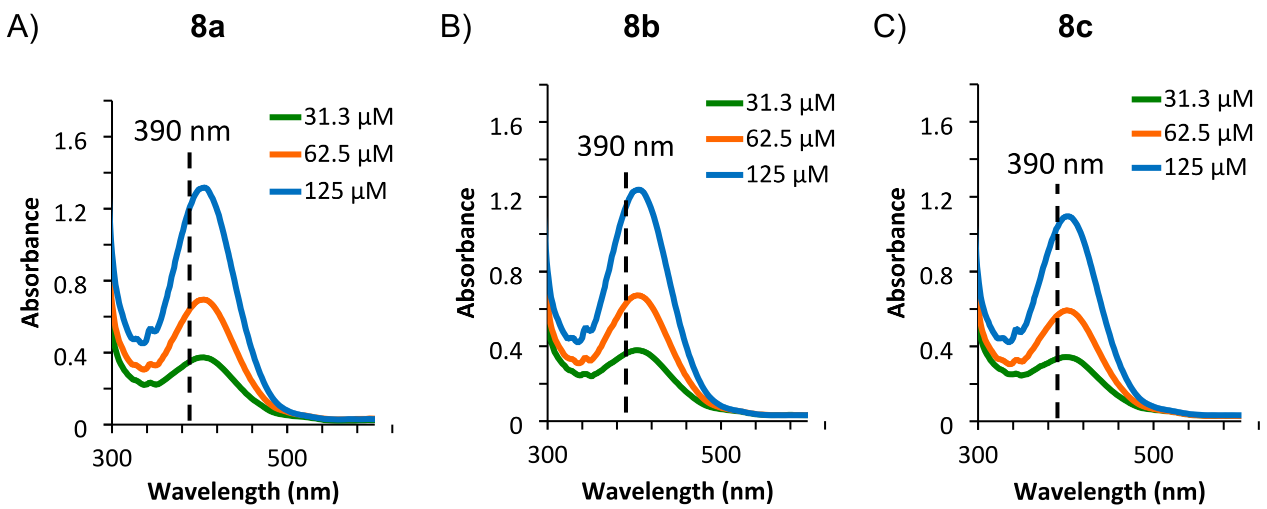

extinction coefficient (ε)	extinction coefficient (ε)	extinction coefficient (ε)
10518 ± 764	10262 ± 1216	9341 ± 1237

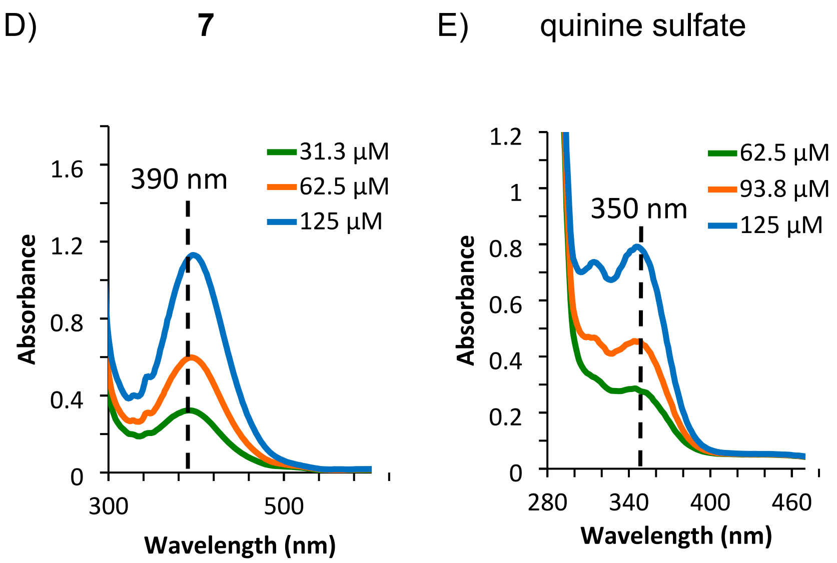

extinction coefficient (ε)	extinction coefficient (ε)
9580 ± 732	7377 ± 1356

Figure S8. Absorbance spectra profiles alongside the measured excitation wavelengths and calculated extinction coefficients for A) **8a**, B) **8b**, C) **8c**, D) **7**, and E) quinine sulfate.

Relative fluorescence quantum yields were determined according to the method of Fery-Forgues.[5] The reported values and standard deviations were determined from triplicate emission spectrums. Literature values used for calculations include the refractive index of 50% DMF in H_2O ($n_X = 1.4165$),[6] the refractive index of 1N H_2SO_4 ($n_S = 1.346$), and the quantum yield of quinine sulfate ($\Phi_{F(S)} = 0.546$ in 1N H_2SO_4). Using the measured areas under the emission curves, as shown in Figure S9, the quantum yields of **8a-c** and **7** were determined.

$$\Phi_{F(X)} = \left(\frac{A_S}{A_X}\right)\left(\frac{F_X}{F_S}\right)\left(\frac{n_X}{n_S}\right)^2 \Phi_{F(S)}$$

Note: quantum yield (Φ_F), absorbance (A), area under the emission spectrum (F), refractive index of solvent (n). Subscripts used were in relation to the unknown (X) and standard (S).

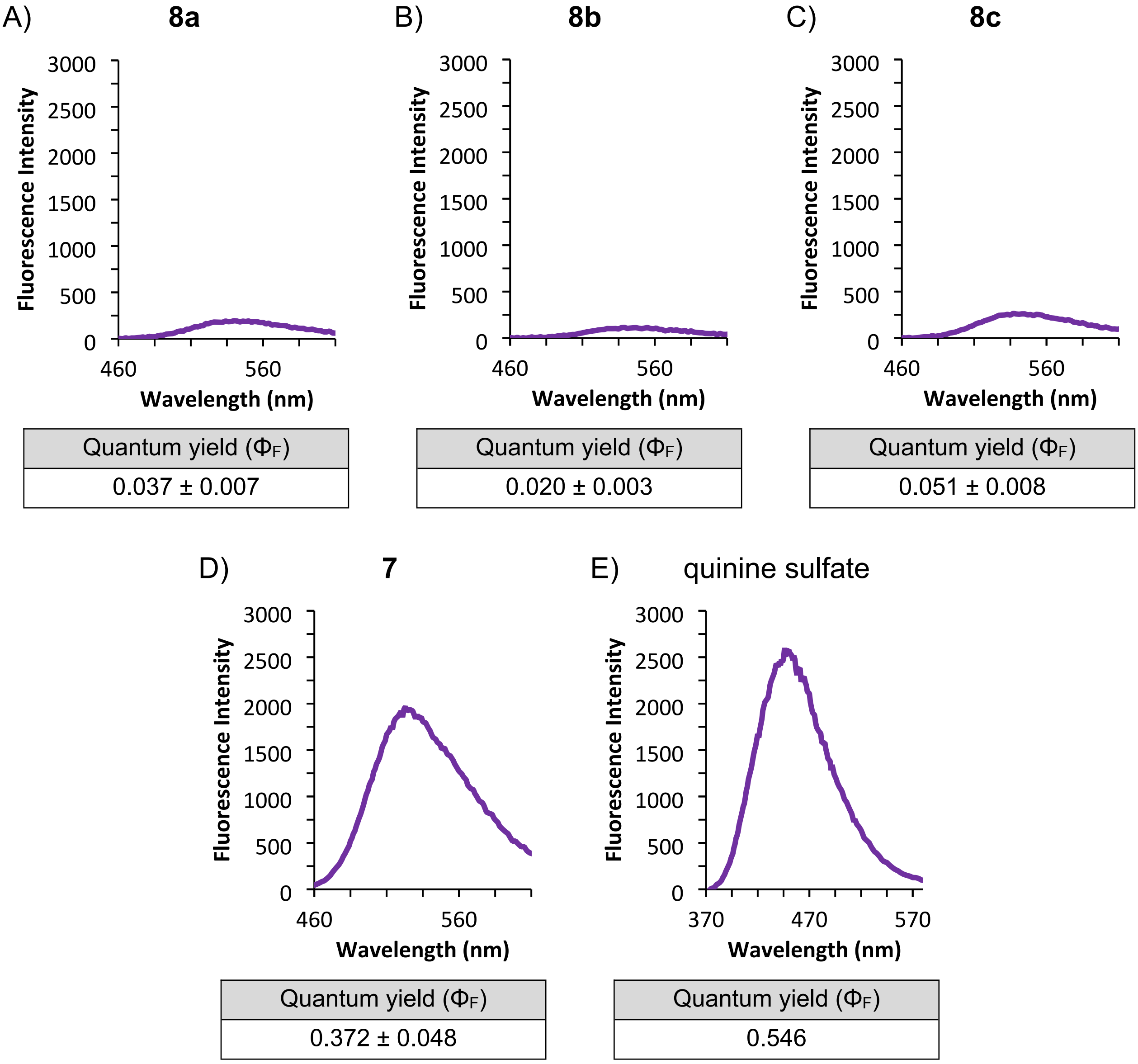

Figure S9. Emission spectra profiles and quantum yield calculations for A) **8a**, B) **8b**, C) **8c**, D) **7**, and E) quinine sulfate. All emission spectra were obtained at a fixed concentration of 31 µM in triplicate. **8a-c** and **7** were excited at a wavelength of 390 nm, while quinine sulfate was excited at a wavelength of 350 nm.

4. Kinetic Analyses

4.1 Methodology for Reaction Rate Constants

Bimolecular reactions in principle follow second-order kinetics since the reaction rates will depend on the concentration of both involved species (Eq 1). To measure these rates, we can adjust the conditions of the reaction to behave like a first-order reaction (pseudo first-order reaction) by having one reactant (**Au1**) applied in excess to assume the relative concentration remains constant (Eq 2). Subsequent equation rearrangement can lead to Ep 3 and Eq 4. By taking the acquired data and integrating it into a plot defined by Eq 4, the pseudo first-order rate constant k' can then be determined.

$$\text{rate} = k[\mathbf{substrate}]\,[\mathbf{Au1}] \tag{1}$$

$$\text{rate} = \frac{d[\mathbf{substrate}]}{dt} = k'[\mathbf{substrate}] \tag{2}$$

$$\ln[\mathbf{substrate}]_\mathrm{t} = -\,k'\mathrm{t} + \ln[\mathbf{substrate}]_0 \tag{3}$$

$$[\mathbf{substrate}]_\mathrm{t} = [\mathbf{substrate}]_0 \times e^{-k'\mathrm{t}} \tag{4}$$

Note: rate constant (k), pseudo first order rate constant (k'), concentration of **substrate** at any time ($[\mathbf{substrate}]_\mathrm{t}$), initial concentration of **substrate** ($[\mathbf{substrate}]_0$)

By plotting the pseudo first-order rate constant k' with the concentration of [**Au1**], which is a constant, the apparent second-order rate constant k ($M^{-1}min^{-1}$) for the bimolecular reaction can then be determined via the relationship set by Eq 5.

$$k' = k[\mathbf{Au1}] \tag{5}$$

Note: pseudo first order rate constant (k'), apparent second-order rate constant (k)

4.2 Determination of Reaction Rate Constants

For experiments with **8a-8c**, stock solutions of **8a-8c** (2.5 mM in DMF) and **Au1** (5, 7, 9, and 11 mM in H_2O) were first made. This was followed by mixing 10 µl of each profluorophore stock solution with 40 µl of DMF in a 96-well black plate. To initiate the reaction, 50 µl of each **Au1** stock solution was added to each respective well. The concentration of released fluorophore **7** was measured by recording the fluorescent intensities (λ_{EX} = 390 nm, λ_{EM} = 535 nm) of the mixtures every 15 minutes (for a total of 900 minutes) on a VANTAstar Microplate Reader (BMG) with a gain setting of 707. Obtained fluorescent values were later converted to concentrations based on interpolation with a product standard curve. Analysis of the data gave the kinetic rates shown in Figure S10-S12.

For experiments with **3a**,**k**,**l**, stock solutions of **3a**,**k**,**l** (2 mM in DMF) and **Au1** (20, 26, 32, and 40 mM in H_2O) were first made. This was followed by mixing 50 µl of each substrate stock solution with 50 µl of each **Au1** stock solution into each respective well of a 96-well black plate. Mixtures were then placed in a sand bath at an adjusted temperature of 37°C without stirring. To workup, mixtures were quenched with 1 mM dodecanethiol in ACN (100 µl) and 1.0M HCl (50 µl). The solutions were then filtered and injected (10 µl) onto a HPLC with an autosampler. Yields were determined by HPLC where relevant peak areas were converted to concentrations based on interpolation with a product standard curve. Analysis of the data gave the kinetic rates shown in Figure S13-S15.

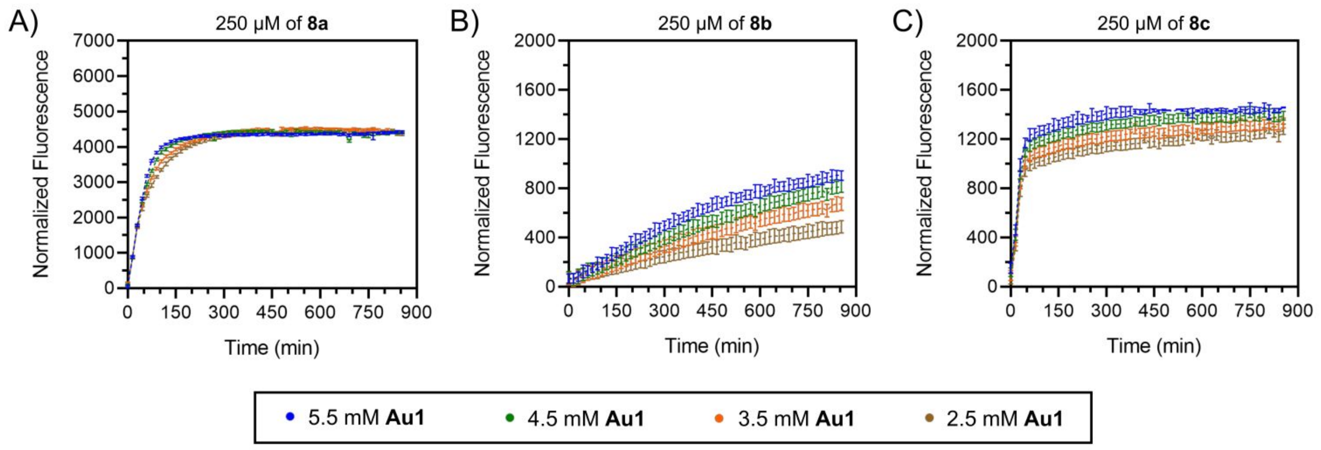

Figure S10. Time-dependent fluorescent monitoring of profluorophores A) **8a**, B) **8b**, and C) **8c** when incubated with **Au1** (2.5, 3.5, 4.5, and 5.5 mM) in a 50% DMF/H_2O mixture at room temperature. Each profluorophore was maintained at a concentration of 250 μM.

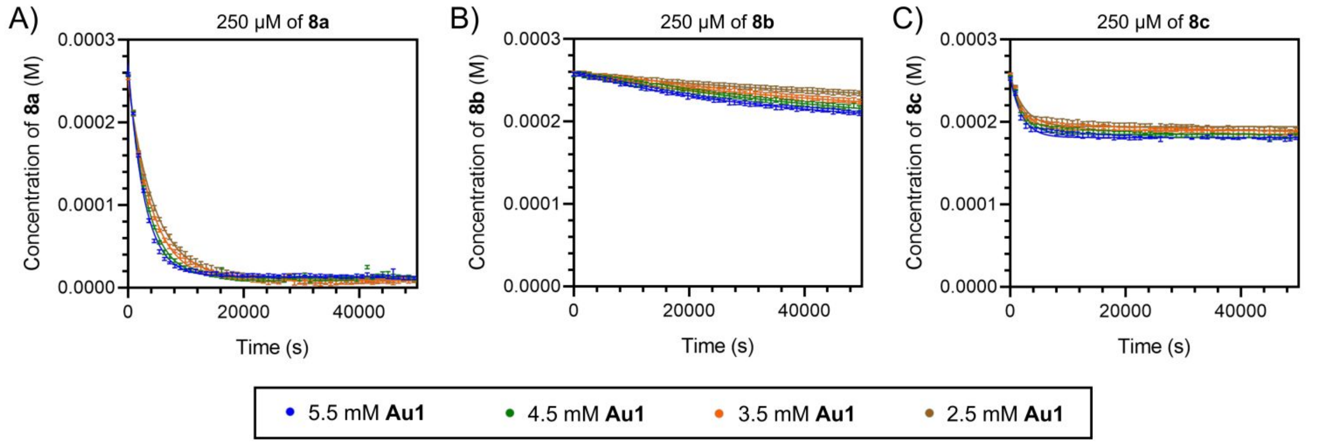

Figure S11. Pseudo first-order kinetics of the reaction between profluorophores A) **8a**, B) **8b**, and C) **8c** and various concentrations of **Au1** (2.5, 3.5, 4.5, and 5.5 mM) in a 50% DMF/H_2O mixture at room temperature. Each profluorophore was maintained at a concentration of 250 μM.

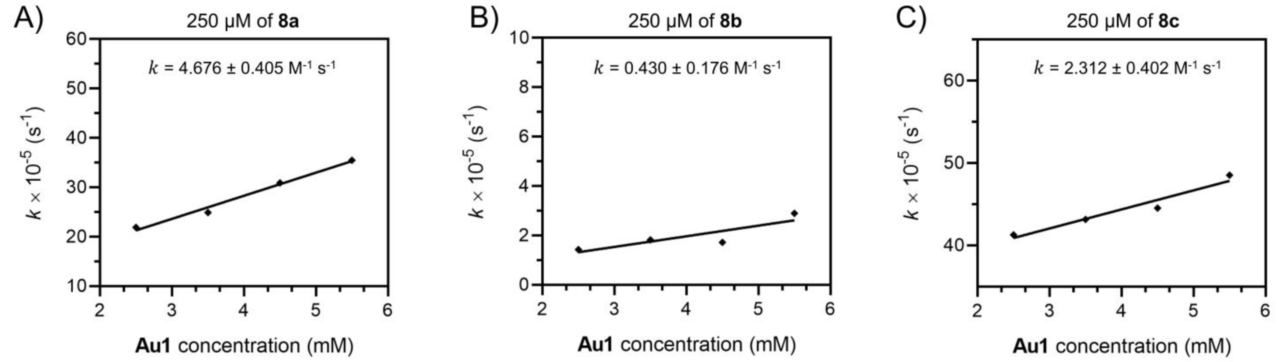

Figure S12. Plot of the pseudo first-order rate constants k' versus varying concentrations of **Au1** for profluorophores A) **8a**, B) **8b**, and C) **8c**. The apparent second-order rate constant k can be determined by extrapolation with Eq 5.

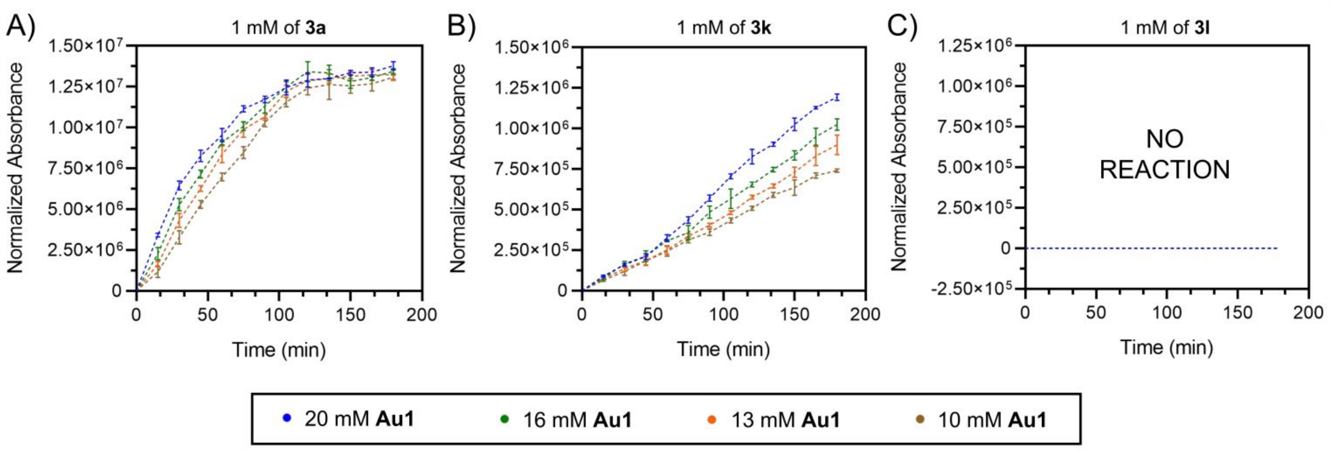

Figure S13. Time-dependent monitoring of the released product **6a** from substrates A) **3a**, B) **3k**, and C) **3l** when incubated with **Au1** (10, 13, 16, and 20 mM) in a 50% DMF/H_2O mixture at 37 °C. Each substrate was maintained at a concentration of 1 mM.

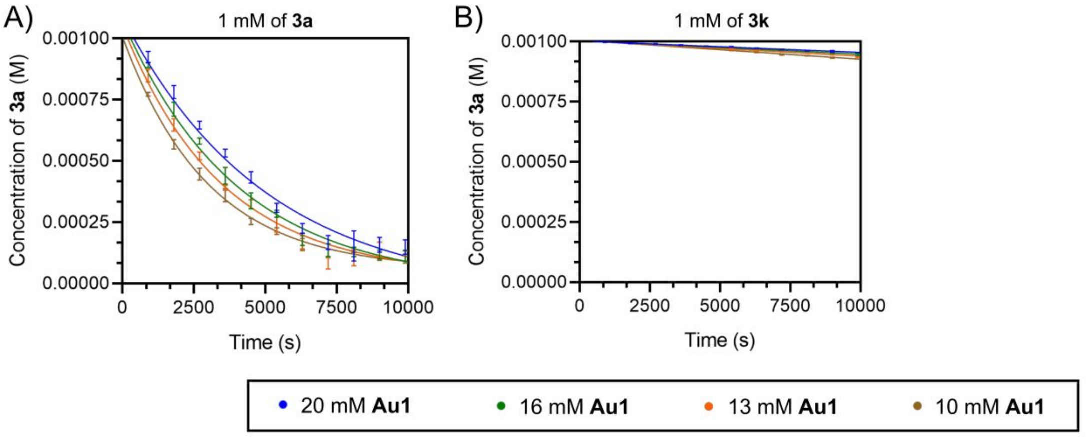

Figure S14. Pseudo first-order kinetics of the reaction between substrate A) **3a**, B) **3k**, and various concentrations of **Au1** (10, 13, 16, and 20 mM) in a 50% DMF/H_2O mixture at 37 °C. Each substrate was maintained at a concentration of 1 mM.

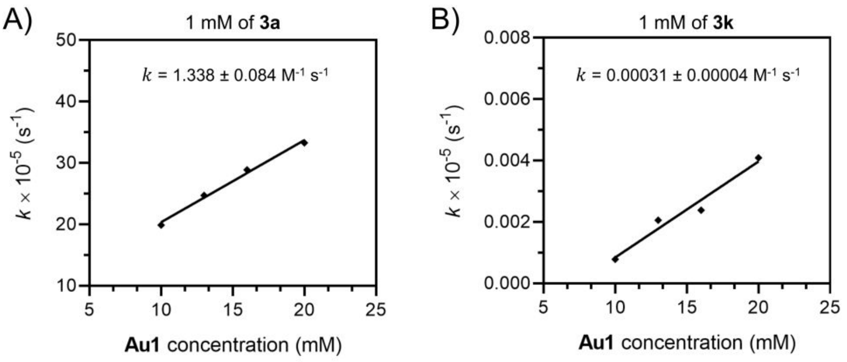

Figure S15. Plot of the pseudo first-order rate constants k' versus varying concentrations of **Au1** for substrate A) **3a** and B) **3k**. The apparent second-order rate constant k can be determined by extrapolation with Eq 5.

5. Modelling studies

5.1 General Information

In the following modeling studies, the three-dimensional X-ray structures of the HaloTag protein (entry 6U32) and doxorubicin-bound DNA (entry 1D12) was taken from the Protein Data Bank. Molecular docking of covalently bound ligands in the HaloTag protein was carried out using AutoDockFR software.[7,8] With the covalently bound tetramethylrhodamine ligand acting as a reference, the docking space was calculated by AutoGridFR.[9] These calculations gave a grid box of 20.25×22.50×19.50 that is centered at 80.114, 48.737, 8.495 (x,y,z) with a spacing of 0.375 Å. Molecular docking of doxorubicin or prodrug **9** to doubled stranded DNA was carried out using Autodock Vina software (version 1.1.2).[10] With the crystallized doxorubicin ligand acting as a reference, the docking space was calculated by AutoGridFR.[9] These calculations gave a grid box of 17.50×16.75×23.50 that is centered at 12.678, 7.553, -20.902 (x,y,z) with a spacing of 0.375 Å. All docking was implemented through the graphical user interface AutoDockTools (v1.5.6),[11] which was used to setup the receptors: all polar hydrogens and Kollman charges were added. The Au parameters were set as r = 3.29 Å and the van der Waals well depth at 0.039 kcal/mol. The 3D structures of **Au10**, doxorubicin, and prodrug **9** were acquired on ChemDraw (v18.1) software, before being cleaned up and saved in PDB format with the aid of the Discovery Studio Visualizer (v17.2.0.16349) program. These molecules were further modified by AutoDockTools to give the corresponding pdbqt files. For the docking runs, default parameters were typically used along with a global search exhaustiveness set to 30.

5.2 Covalent docking of gold catalyst to the HaloTag protein

In this study, covalent docking was performed using **Au10** anchored to Asp106 of the HaloTag protein (PDB 6U32). Following docking, the distance from the Asp106 anchor point to the gold atom was measured to be approximately 11.4 Å (Figure S16), which places it within the protein binding pocket.

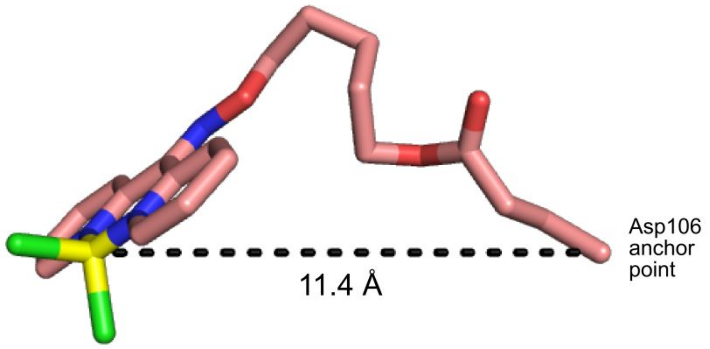

Figure S16. Docking studies were carried out to predict the configuration of **Au10** (pink) once bound inside the binding pocket of the HaloTag protein. The measured length is made from the Asp106 anchor point to the gold atom of the catalyst.

5.3 Docking of doxorubicin-based prodrug to dsDNA

In this study, prodrug **9** was designed as a non-toxic, masked doxorubicin analogue. Since doxorubicin is an anticancer agent that mainly exerts its cytotoxic effects through DNA intercalation, binding studies were carried out with prodrug **9** to dsDNA (Figure S17). As a control to first validate the docking methodology, doxorubicin was modelled with dsDNA and compared to its known crystallized binding mode. Overlaying the docked and crystallized doxorubicin (from literature) clearly shows their similar binding configurations (Figure S17A). Furthermore, full DNA intercalation is expected as both docked and crystallized doxorubicin show positioning centralized within the double stranded DNA helix (Figure S17B). Moving on, prodrug **9** was modelled and then overlayed with crystallized doxorubicin, which shows different binding configurations (Figure S17C). In the context of DNA binding, prodrug **9** displays only partial DNA intercalation within the double stranded DNA helix (Figure S17D). This is enough evidence to suggest that prodrug **9** may possess less cytotoxicity compared to doxorubicin due to poorer DNA intercalation.

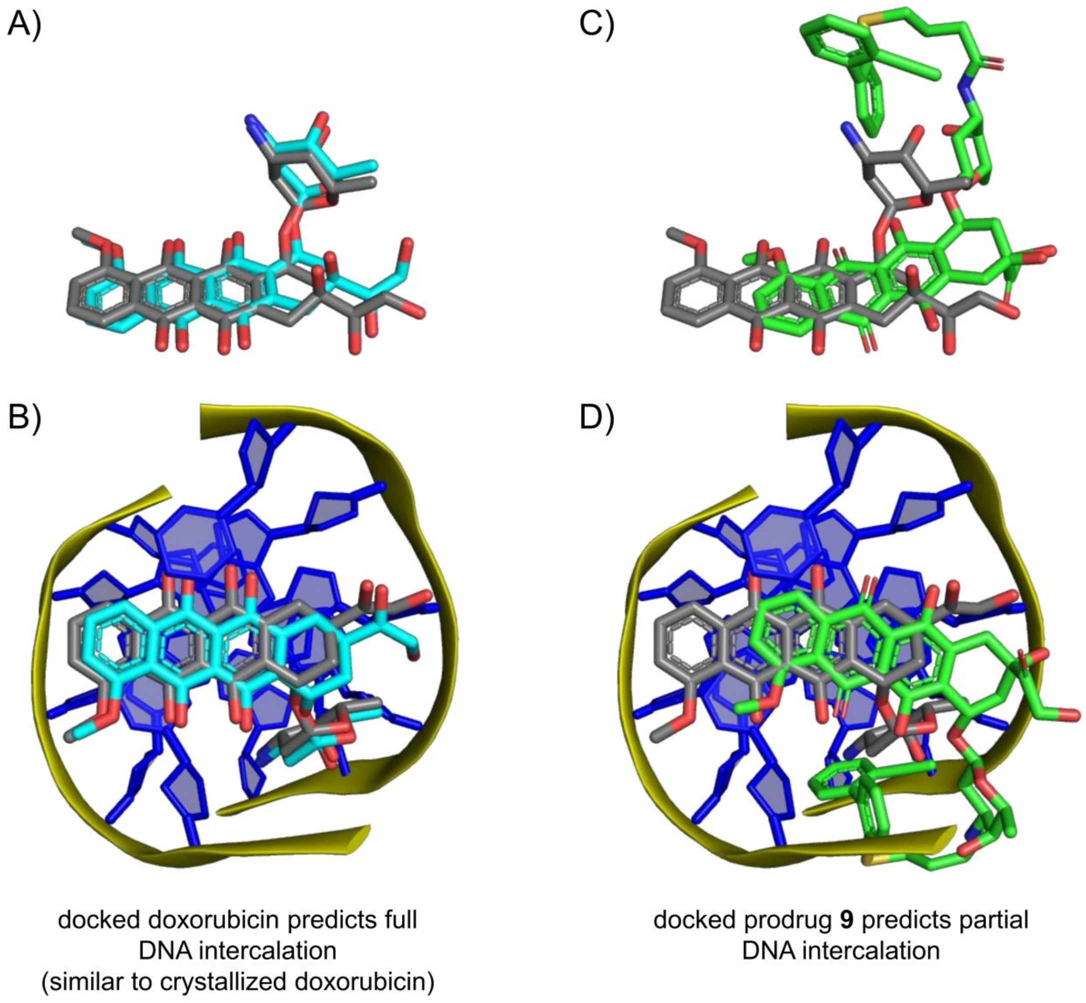

Figure S17. Modelled docking of prodrug **9** and doxorubicin into dsDNA carried out in this study. A) Overlay of docked doxorubicin (cyan) and crystallized doxorubicin (gray) to highlight their similar binding configurations. B) The positioning of docked doxorubicin within double stranded DNA (similar to crystallized doxorubicin) suggests full DNA intercalation. C) Overlay of docked prodrug **9** (green) and crystallized doxorubicin (gray) to highlight their different binding configurations. D) The positioning of docked prodrug **9** within double stranded DNA suggests only partial DNA intercalation.

6. Protein preparations and experiments

6.1 General Information

General reagents and buffer components were purchased from Sigma-Aldrich, Scharlau, or Oxoid without further purification. For plasmid transformations, One Shot™ BL21(DE3) Chemically Competent *E. coli* were acquired from ThermoFisher. For protein expression and purification, items such as Pierce protease inhibitor tablets, HisPur Ni-NTA Cartridges, 10K MWCO protein concentrators, and the Pierce Bradford Protein Assay Kit were all acquired from ThermoFisher.

6.2 Recombinant protein expression and purification

The plasmid expressing the HaloTag protein (**Ht**) and the Halotag-PduU-ACG lectin fusion protein (**HtPA**) has been reported in one of our previous studies.[1] To perform the bacterial expression of **Ht** and **HtPA**, plasmids were transformed into BL21(DE3) *E. coli* and then incubated on Luria-Bertani (LB) Agar plates with ampicillin (50 µg/mL) overnight at 37 °C. Isolated colonies were picked and cultured in 7 mL LB broth with ampicillin (50 µg/mL) overnight in shaking incubators at 37 °C. These overnight cultures were then used to inoculate larger LB cultures (500 mL), which were grown in shaking incubators at 37 °C until an O.D. reading (at 600 nm) of 0.6 was reached. To induce protein expression, cultures were supplemented with 0.5 mM isopropyl β-D-1-thiogalactopyranoside (IPTG) and then grown for an additional 4 hours at 28 °C. Bacterial pellets were obtained through centrifugation (7,350 rpm at 4 °C for 10 min) and then resuspended in lysis buffer (20 mM Tris, 300 mM NaCl, 1 mM PMSF, pH 7.4) supplemented with a protease inhibitor tablet. Sonication was performed (10s on/15s off for 15 min), followed by centrifugation (12,000 rpm at 4 °C for 20 min) to isolate the supernatant. To perform protein purification, the the supernatant was loaded onto a 5 mL Ni-NTA column connected to an ÄKTA start FPLC system (Cytiva). At least 10 column volumes of an equilibration buffer (20 mM Tris, 300 mM NaCl, pH 7.4) was used to wash the column. An imidazole gradient (0–300 mM) was then applied to the column by mixing the equilibration buffer with an elution buffer (20 mM Tris, 300 mM NaCl, 300 mM imidazole, pH 7.4). Eluted protein fractions were analyzed by SDS-PAGE, with appropriate fractions then collected and combined. Following volume reduction using 30K MWCO concentrators, buffer exchange using PBS buffer was done to remove any remaining eluting agents. Final protein concentrations were determined via a Bradford protein assay.

6.3 Artificial metalloenzyme preparation

Figure S18 depicts the general steps taken to produce the ArMs used in this study (**Ht-Au10**, **HtPA-Au10**). To block any potential free cysteine residues on protein surfaces, purified proteins (83 nmol, 500 µl from a 165 µM stock solution in PBS buffer) were first mixed with *N*-ethylmaleimide (30 µmol, 200 µl from a 148 mM stock solution in H_2O). The solution was then mildly mixed and incubated at room temp for 2 hr. To confirm cysteine blockage, a small sample of the solution was removed (1 µl) and mixed with Ellman's reagent (9 mM, 9 µl from a 10 mM stock solution in PBS buffer). Following a short 1 min incubation, the absorbance at 412 nm was measured. Under these conditions, there is typically a significant observed signal reduction compared to unprotected protein. After buffer exchange using 10K MWCO protein concentrators to remove excess NEM, the protein solution was diluted to create a 100 µM stock solution. In the next step, NEM-protected proteins (10 nmol, 100 µl from a 100 µM stock solution in PBS buffer) were then mixed with 260 µl of PBS buffer and **Au10** (20 nmol, 40 µl from a 500 µM stock solution in DMSO). The solution was then mildly mixed and incubated at room temp for 2 hr. Afterwards, buffer exchange/volume reduction was carried using 10K MWCO protein concentrators to obtain the desired ArM concentrations.

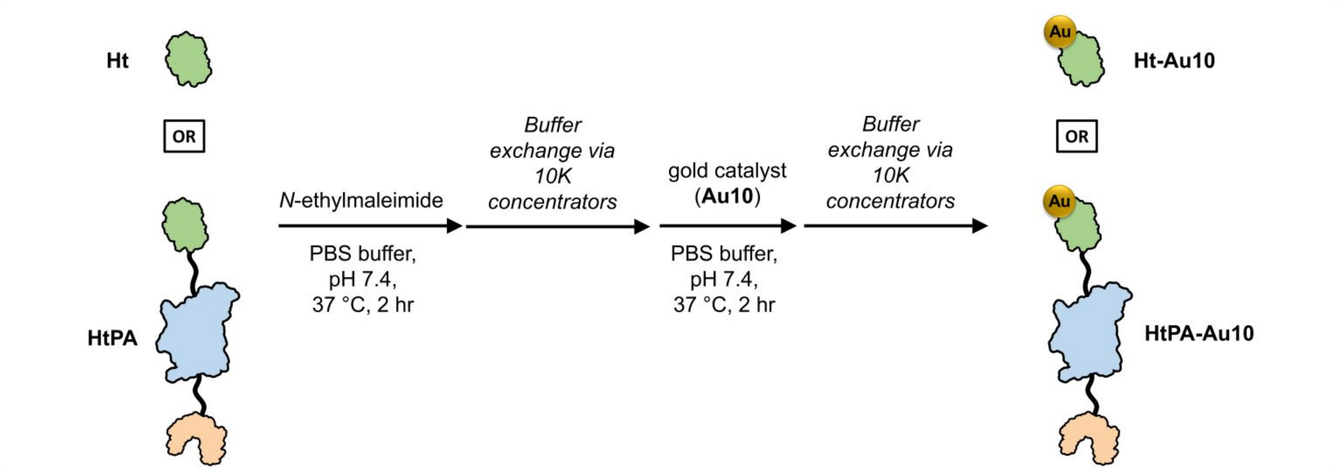

Figure S18. Preparation of ArMs (**Ht-Au10**, **HtPA-Au10**) used in this study.

6.4 Artificial metalloenzyme characterization

To determine the Au content in the prepared artificial metalloenzyme, 200 µl of **Ht-Au10** (100 µM) was submitted to the Health, Safety, and Environment Office (HSEO) laboratory services of HKUST. Samples were first digested with nitric acid, before analysis on an Inductively Coupled Plasma Mass Spectrometer (ICP-MS) Agilent 7900.

6.5 Reactivity studies using artificial metalloenzymes

A HaloTag ArM (**Ht-Au10**) was used to evaluate EBB reactivity by reacting with substrate **9** for the release of doxorubicin. To initiate the reaction, 10 µl of **9** (1 mM stock solution in DMSO) was mixed with 50 µl of **Ht-Au10** (2 µM stock solution in PBS buffer), 30 µl of PBS buffer, and 10 µl of relevant media. These mixtures were then incubated for 12 hours in a sand bath at an adjusted temperature of 37°C without stirring. To workup, mixtures were quenched with 1 mM dodecanethiol in ACN (25 µl). The solutions were then filtered and injected (100 µl) onto a HPLC with an autosampler using various HPLC method 1. Data regarding the decaging of **9** is shown in Table S12.

Table S12. Artificial metalloenzyme catalyzed decaging of **9** for the release of doxorubicin

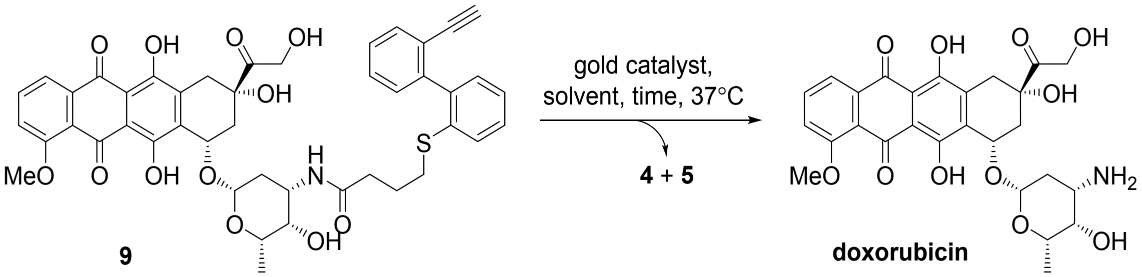

Entry	Catalyst	Mol%	Solvent	Yield of doxorubicin (%)[a]	Turnover (TON)[a]
167	**Au10**	1	1:9 DMSO/PBS buffer	5.90 ± 0.26	~5.9
168	**Ht-Au10**	1	1:9 DMSO/PBS buffer	1.97 ± 0.16	~2.0
169	none	1	1:9 DMSO/PBS buffer	undetected	~0
170	**Au10**	1	1:1:8 DMSO/serum/PBS buffer	1.69 ± 0.12	~1.7
171	**Ht-Au10**	1	1:1:8 DMSO/serum/PBS buffer	1.00 ± 0.20	~1
172	none	1	1:1:8 DMSO/serum/PBS buffer	undetected	~0

[a]Yields determined by HPLC (peak retention times compared to product standards, followed by MS analysis for confirmation, and then calculation of yields based on product standard curves). All reactions were standardized to 0.01 µmol of **9** in 100 µl of solvent.

7. Cell-based Assays

7.1 General Cell Culture

In this study, the cancer cell lines SW620, and DU-145 were obtained from the Japanese Collection of Research Bioresources cell bank (Japan), A549 was obtained from the iCell Bioscience (China), while HeLa and MDA-MB-231 were obtained from ATCC (USA) via donation from Prof. Randy YC Poon. Cells were incubated at 37 °C in a humidified incubator with an atmosphere of 5% CO2 and 95% air. Specific growth media used are indicated as follows:

Name	Type	Medium	FBS	Penicillin-Streptomycin
HeLa	human cervix adenocarcinoma cells	DMEM	10%	1%
MDA-MB-231	human breast adenocarcinoma cells	DMEM	10%	1%
KKU-213	human cholangiocarcinoma cell	DMEM	10%	1%
SW620	human colon adenocarcinoma cells	RPMI 1640	10%	1%
DU-145	human prostate carcinoma cells	DMEM	10%	1%
A549	human lung adenocarcinoma cells	DMEM	10%	1%

7.2 Statistical analysis

For the cell assays conducted in this study, statistical analysis was performed using a one-way ANOVA with Tukey's multiple comparisons test. All numerical data is presented as mean ± s.e.m. of three replicates. *P<0.03, **P<0.002, ***P<0.0002, ****P<0.0001, ns = not significant.

7.3 Cell Cytotoxicity Studies

Cell viability was determined using a colorimetric MTS Assay Kit (Abcam). Based on cell titration experiments (data not shown), cells were first seeded onto 96-well plates at a density of 2500 cells per well and grown overnight at 37 °C. The media was then removed, followed by the addition of varying compound mixtures (20 µl) and growth media (80 µl). For the mixtures, these contained differing concentrations of prodrug **9** and ArM (**HtPA**, **Ht-Au10**, **HtPA-Au10)**. Following an incubation time of 4 days, the media was then removed and replaced with 20 µl of MTS reagent and 80 µl of growth media. After cells were further incubated at 37 ºC for 2 hr, cell viability was determined by the absorbance at 490 nm measured using a VANTAstar Microplate Reader (BMG). The background control for this assay was the incubation of 20 µl MTS reagent and 80 µl media in the absence of cells. Growth curves for MDA-MB-231 treated with doxorubicin or prodrug **9** only are shown in Figure S19A. The toxicity of **HtPA-Au10** was also tested up to a concentration of 1 µM (Figure S19B).

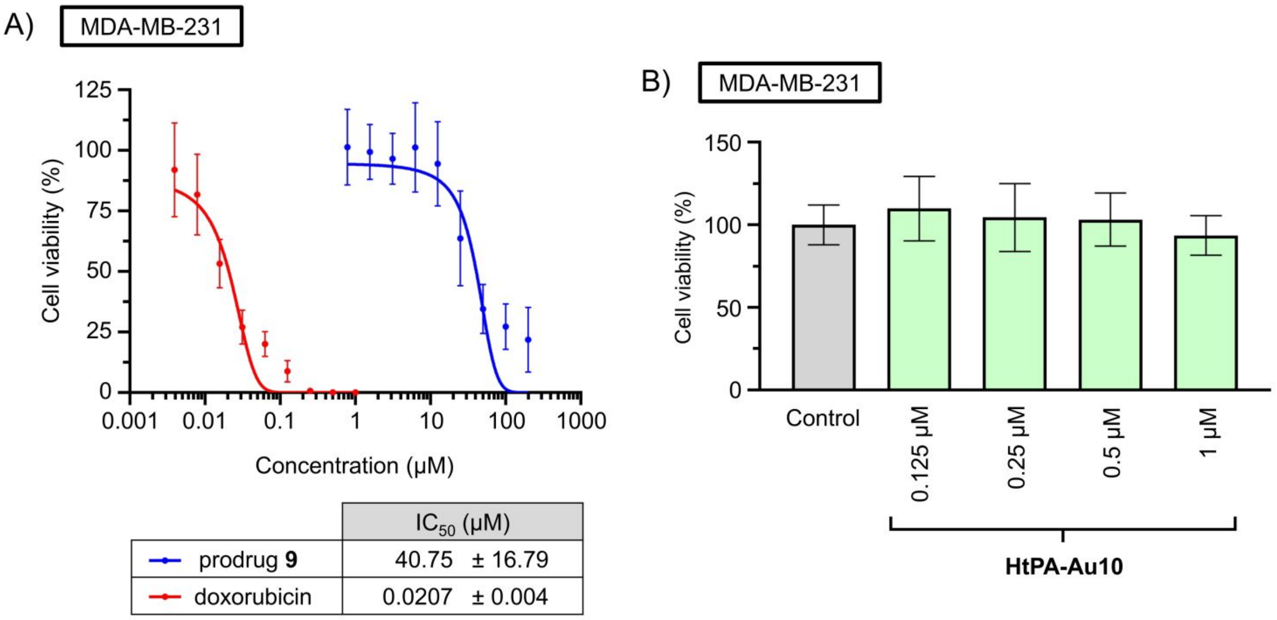

	IC_{50} (μM)
prodrug **9**	40.75 ± 16.79
doxorubicin	0.0207 ± 0.004

Figure S19. Cell cytotoxicity assays. A) Growth curves were constructed for MDA-MB-231 cancer cells treated with either doxorubicin (red) or prodrug **9** (blue). Subsequent analysis allowed determination of the IC_{50} for each compound. B) Cell viability tests for MDA-MB-231 cancer cells treated with **HtPA-Au10** to determine its intrinsic toxicity.

8. NMR spectra

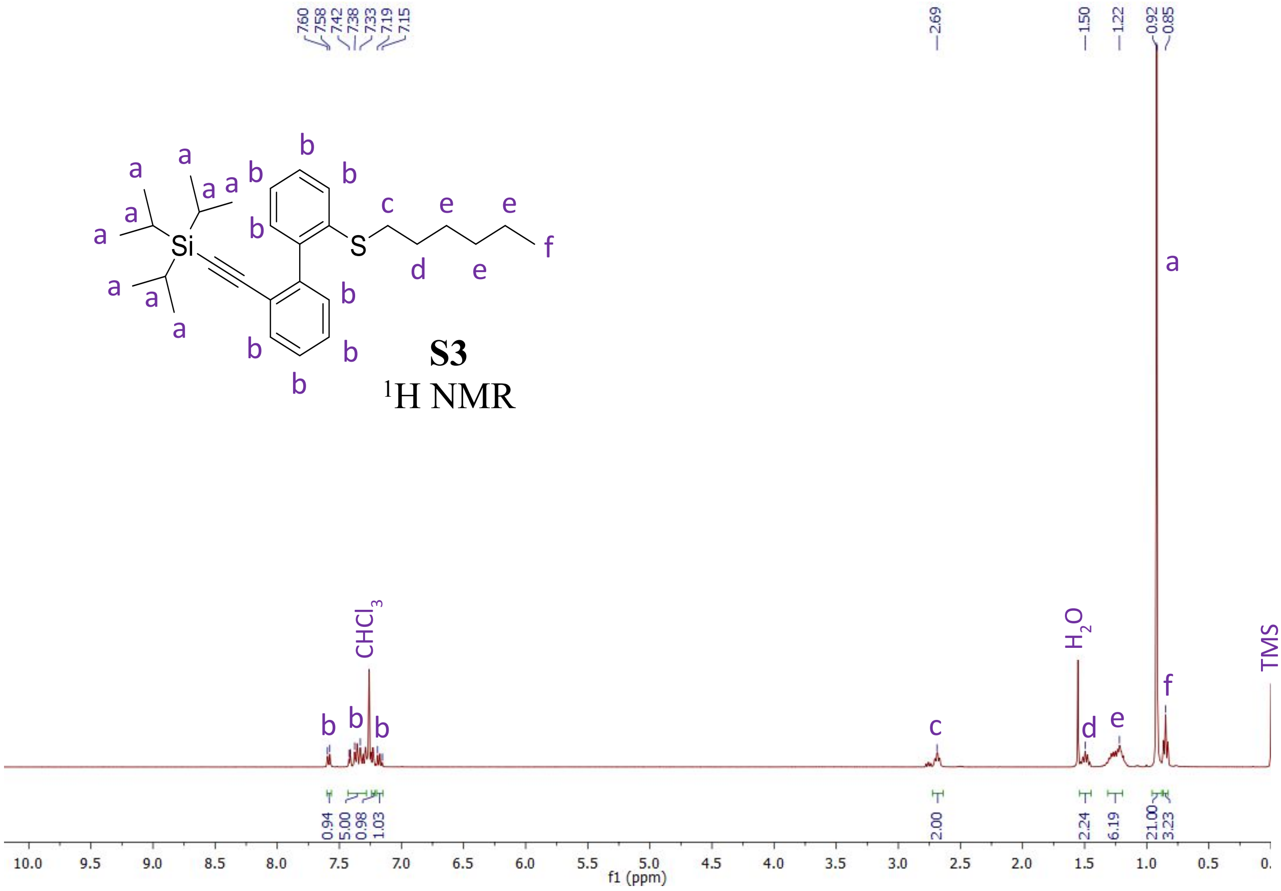

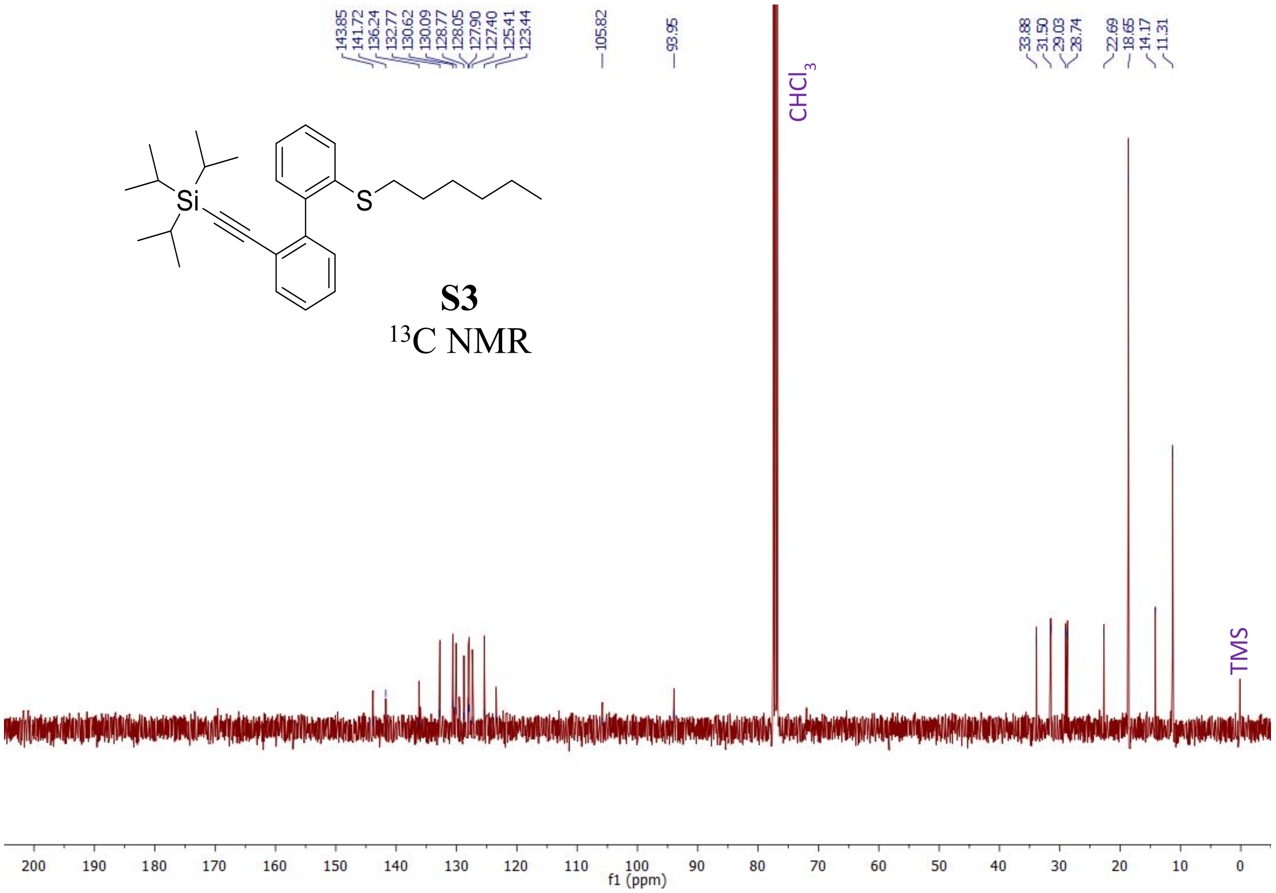

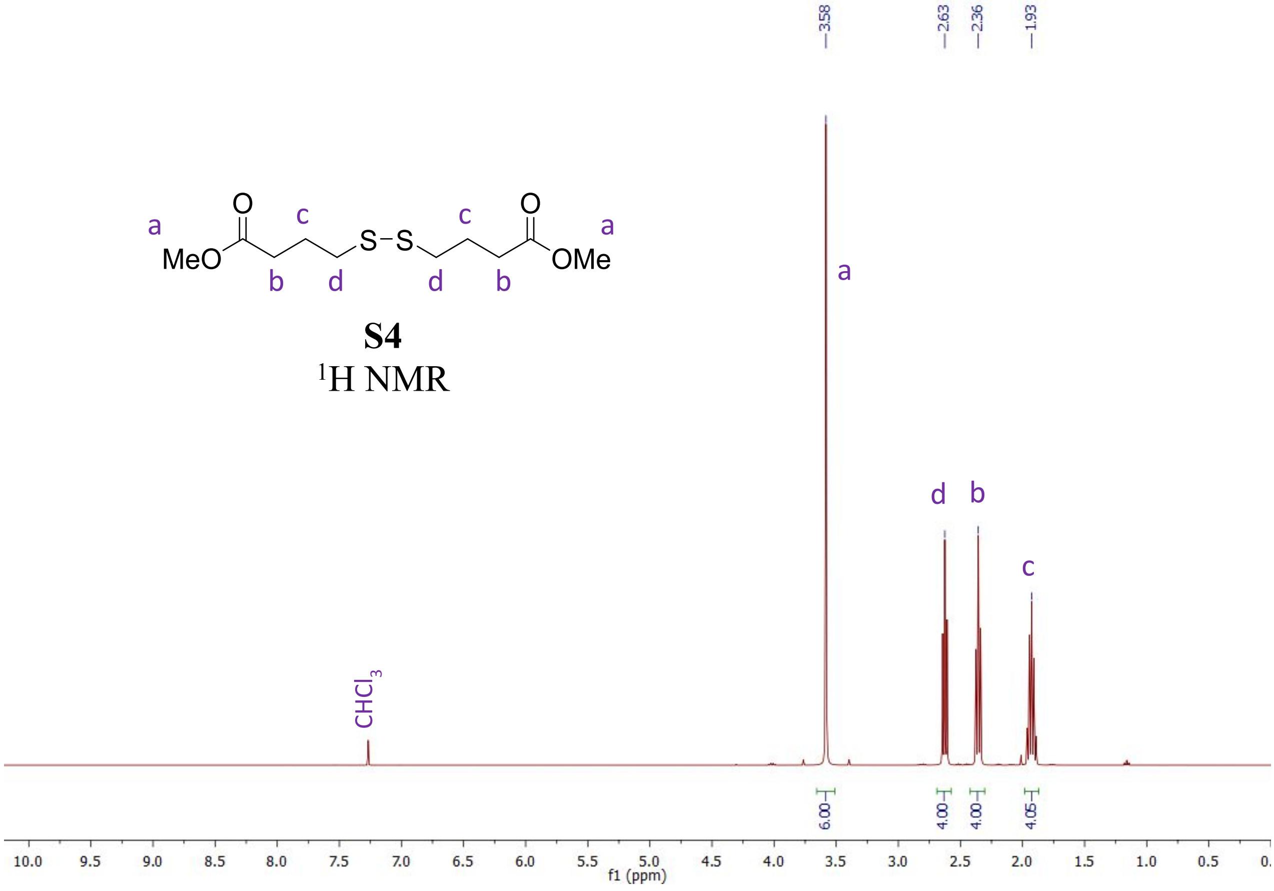
S4
1H NMR
a
b
c
d
CHCl3
3.58
2.63
2.36
1.93
6.00
4.00
4.00
4.05
f1 (ppm)

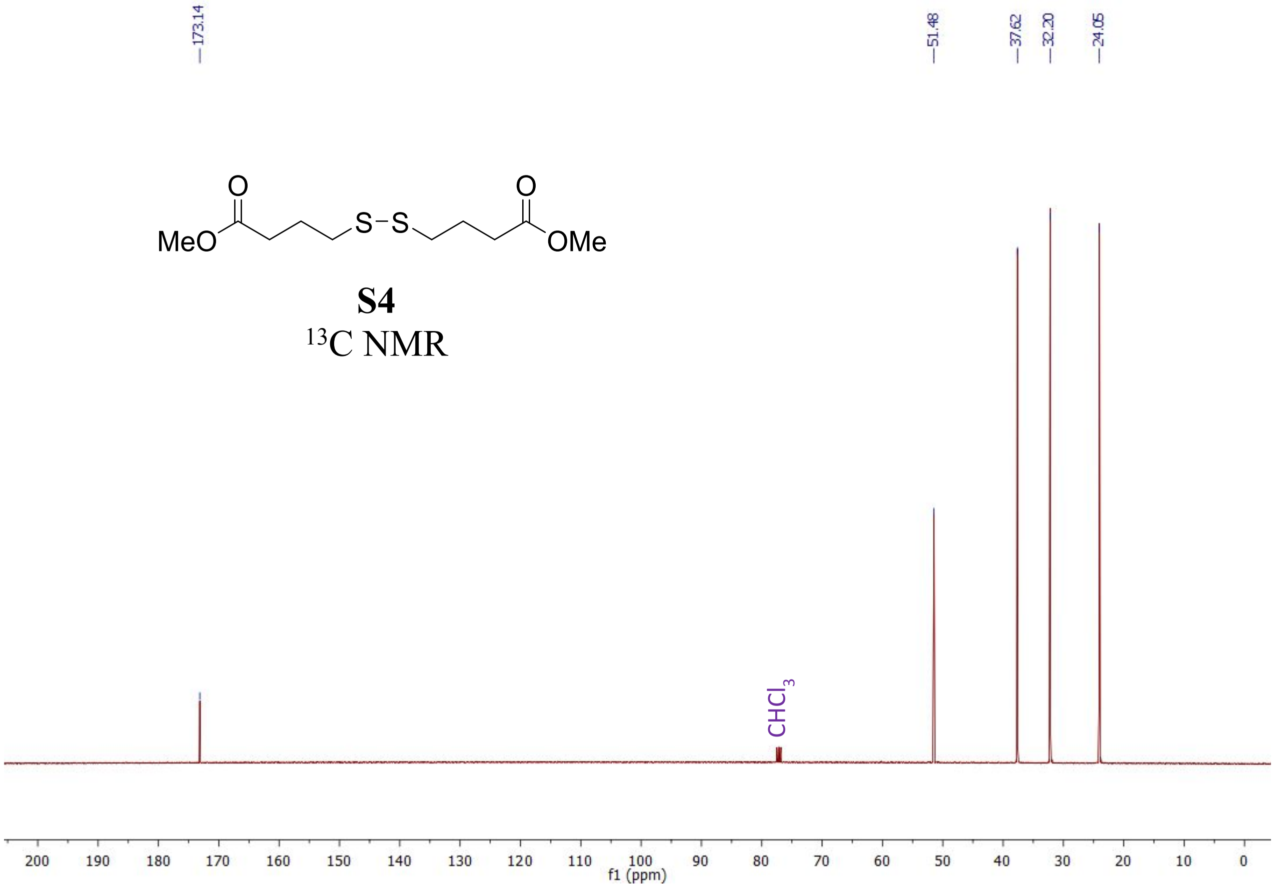
S4
13C NMR
CHCl3
173.14
51.48
37.62
32.20
24.05
f1 (ppm)

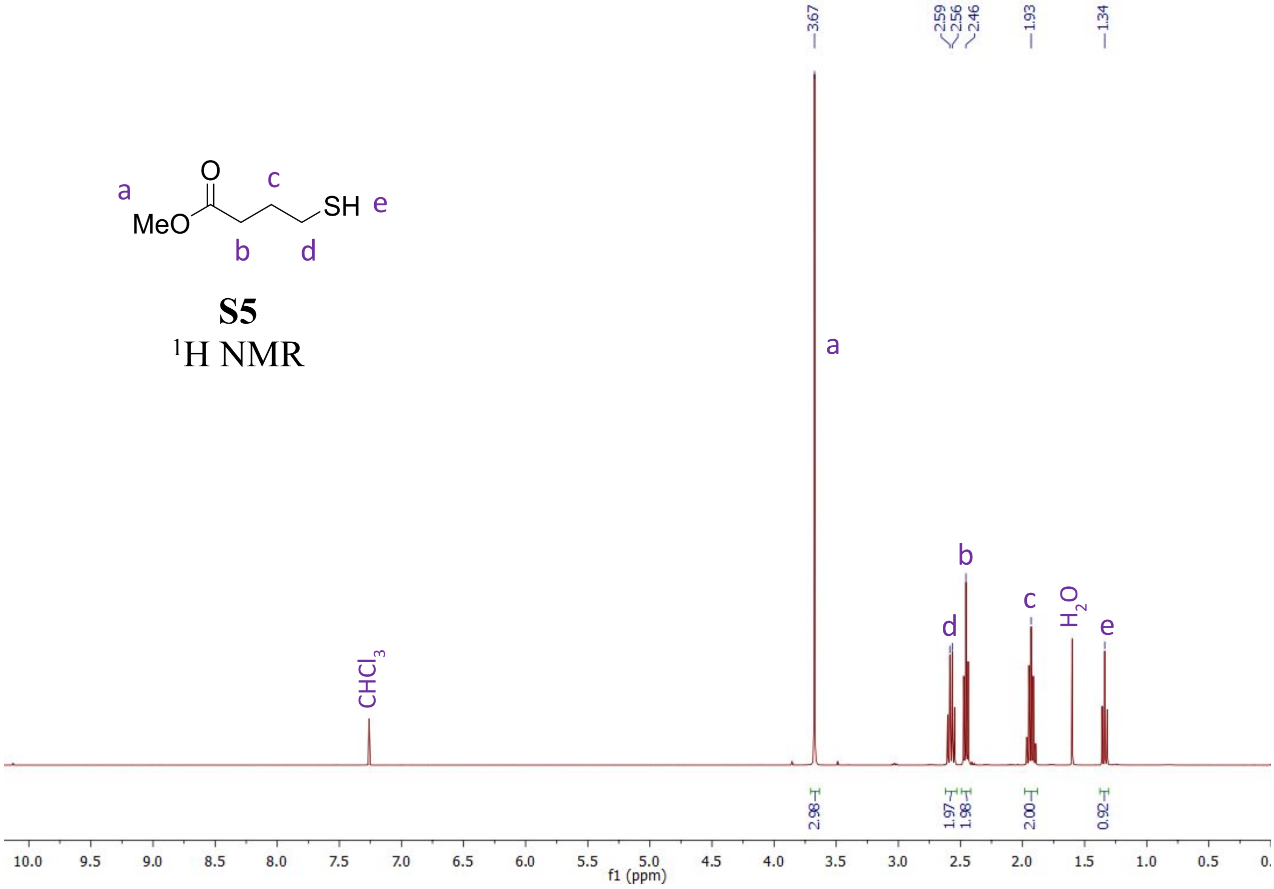

a
c
MeO
SH
e
b
d
S5
¹H NMR
3.67
2.59
2.56
2.46
1.93
1.34
a
b
c
d
e
H_2O
$CHCl_3$
2.98
1.97
1.98
2.00
0.92
f1 (ppm)

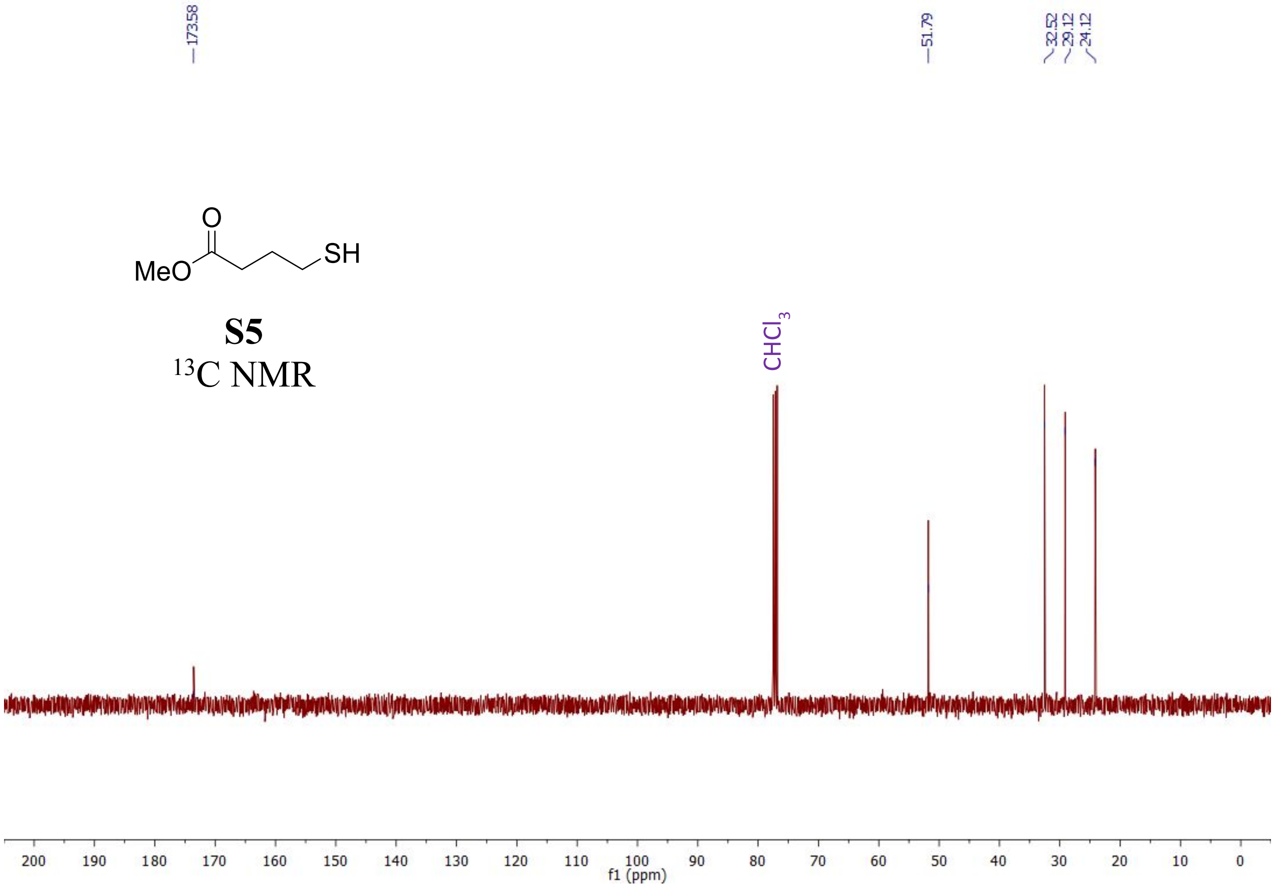

173.58
51.79
32.52
29.12
24.12
MeO
SH
S5
¹³C NMR
$CHCl_3$
f1 (ppm)

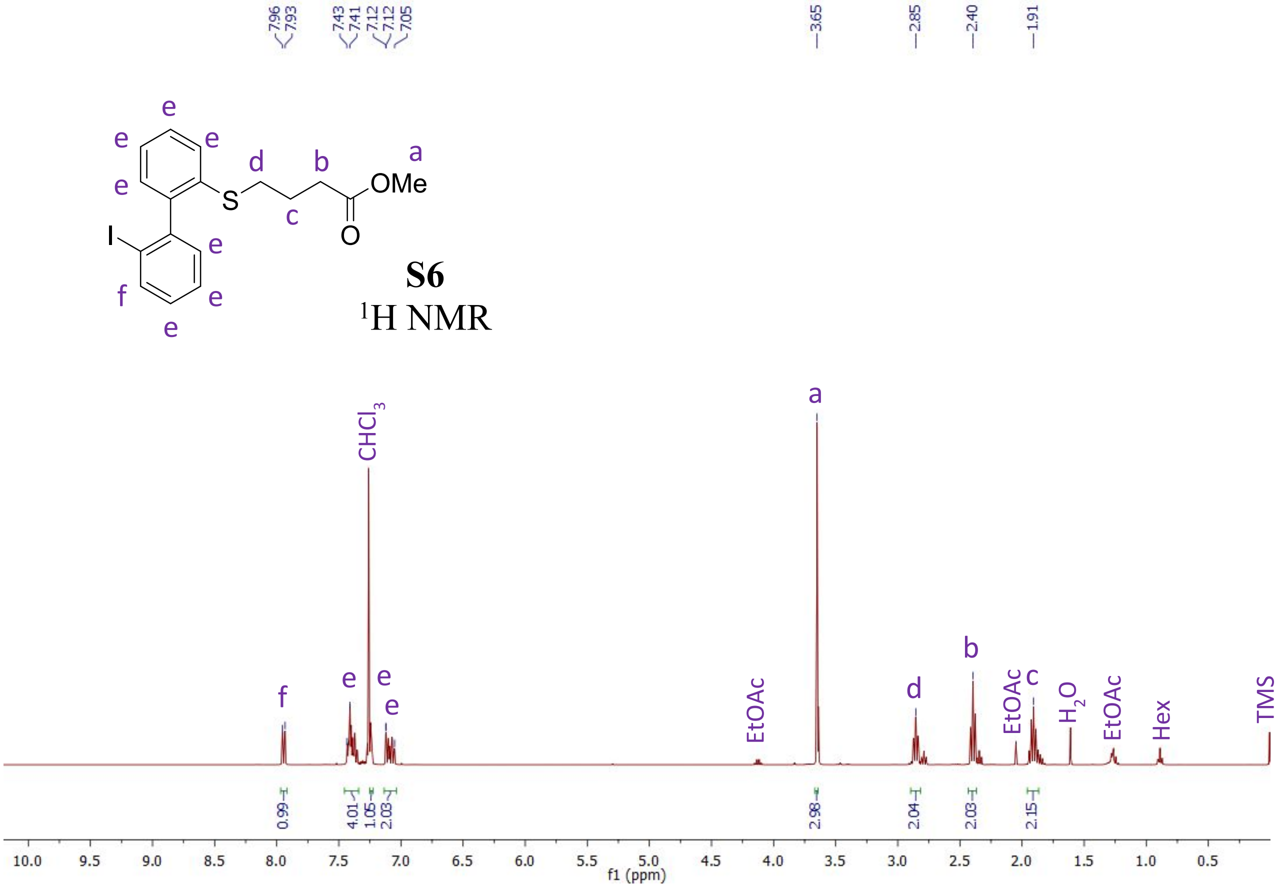
7.96
7.93
7.43
7.41
7.12
7.12
7.05
3.65
2.85
2.40
1.91
S6
1H NMR
OMe
S
I
O
a
b
c
d
e
f
CHCl3
EtOAc
H2O
Hex
TMS
0.99
4.01
1.05
2.03
2.98
2.04
2.03
2.15
f1 (ppm)

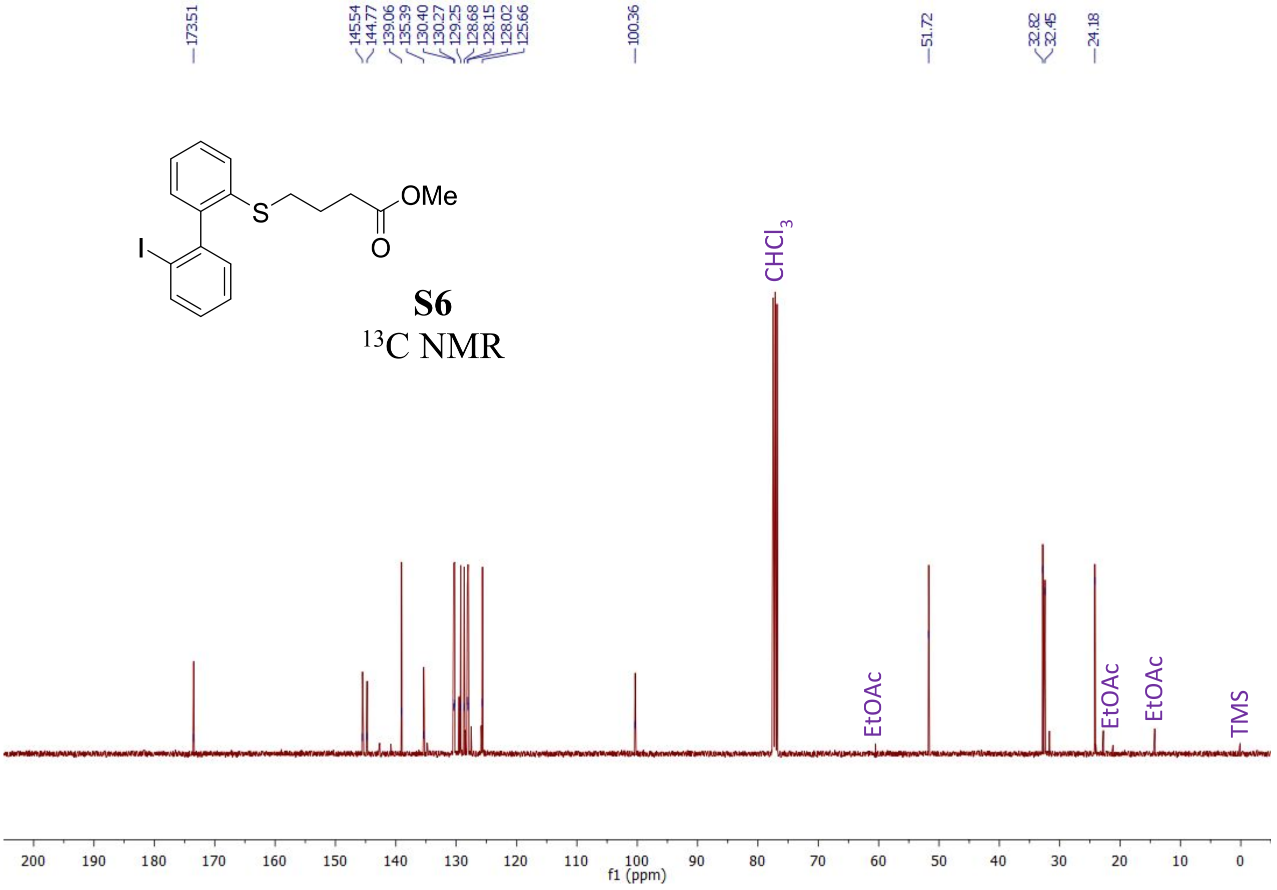
173.51
145.54
144.77
139.06
135.39
130.40
130.27
129.25
128.68
128.15
128.02
125.66
100.36
51.72
32.82
32.45
24.18
S6
13C NMR
OMe
S
I
O
CHCl3
EtOAc
EtOAc
EtOAc
TMS
f1 (ppm)

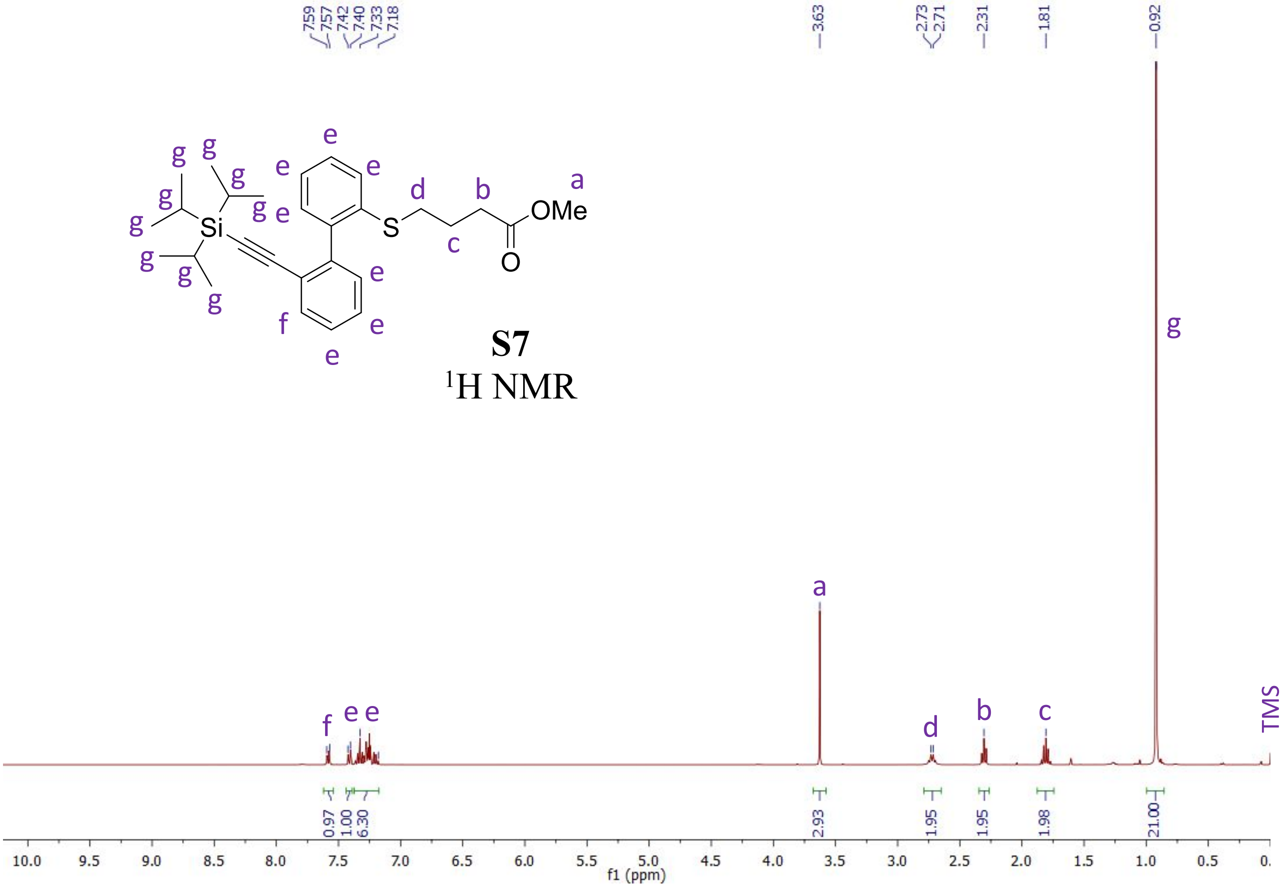
7.59
7.57
7.42
7.40
7.33
7.18
3.63
2.73
2.71
2.31
1.81
0.92
S7
^{1}H NMR
a
b
c
d
e
f
g
TMS
0.97
1.00
6.30
2.93
1.95
1.95
1.98
21.00
f1 (ppm)

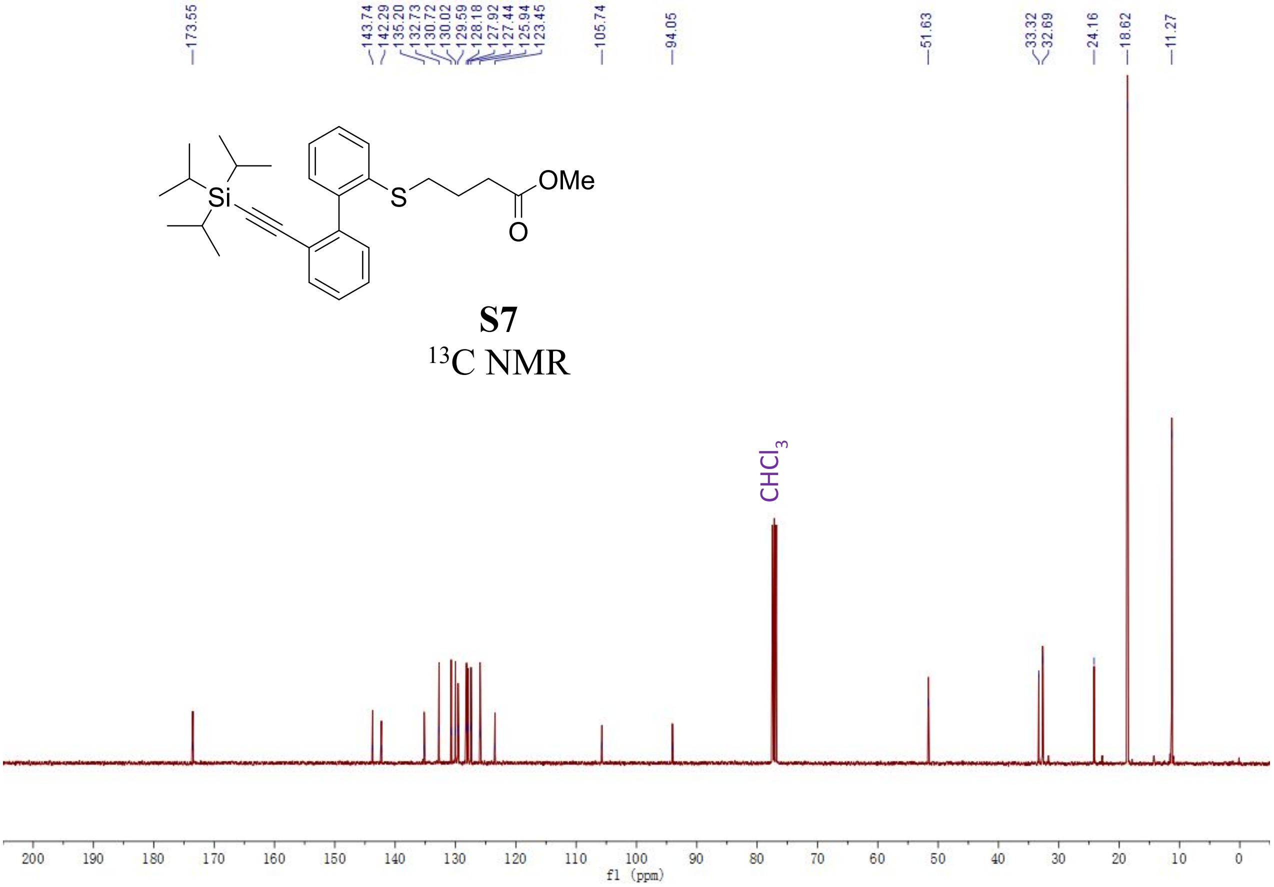
173.55
143.74
142.29
135.20
132.73
130.72
130.02
129.59
128.18
127.92
127.44
125.94
123.45
105.74
94.05
51.63
33.32
32.69
24.16
18.62
11.27
S7
^{13}C NMR
$CHCl_3$
f1 (ppm)

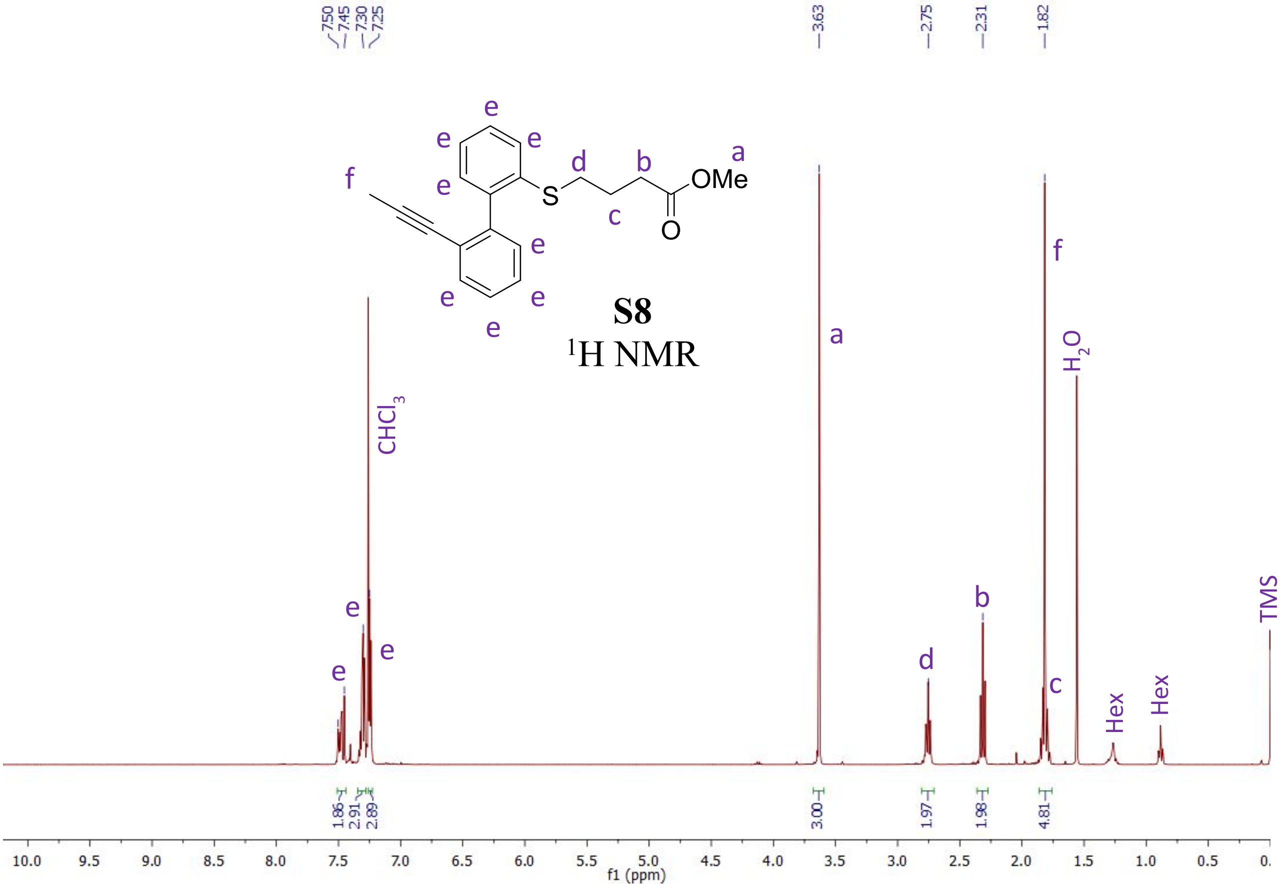
7.50
7.45
7.30
7.25
3.63
2.75
2.31
1.82
S8
^{1}H NMR
OMe
O
S
a
b
c
d
e
f
CHCl$_3$
H_2O
Hex
TMS
1.86
2.91
2.89
3.00
1.97
1.98
4.81
f1 (ppm)

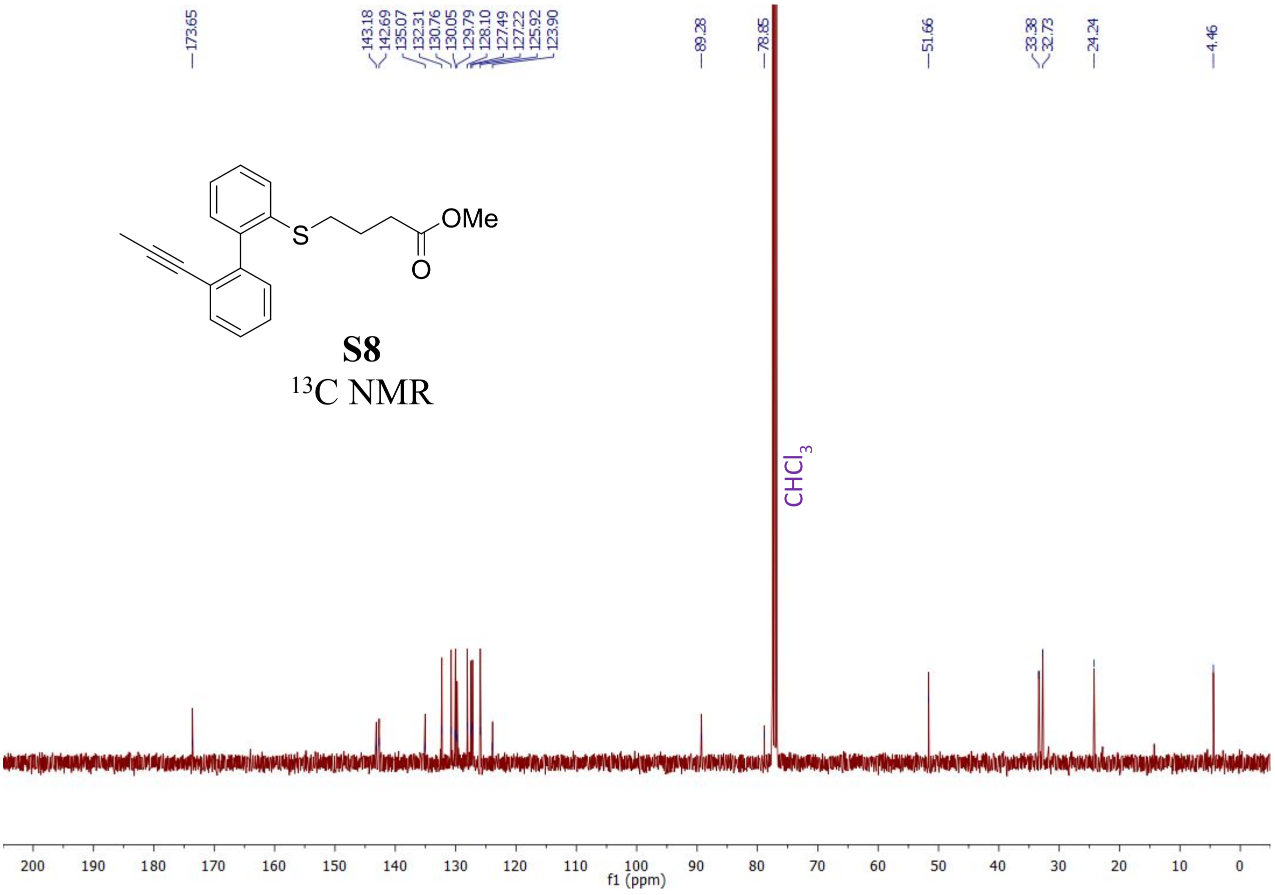
173.65
143.18
142.69
135.07
132.31
130.76
130.05
129.79
128.10
127.49
127.22
125.92
123.90
89.28
78.85
51.66
33.38
32.73
24.24
4.46
S8
^{13}C NMR
OMe
O
S
CHCl$_3$
f1 (ppm)

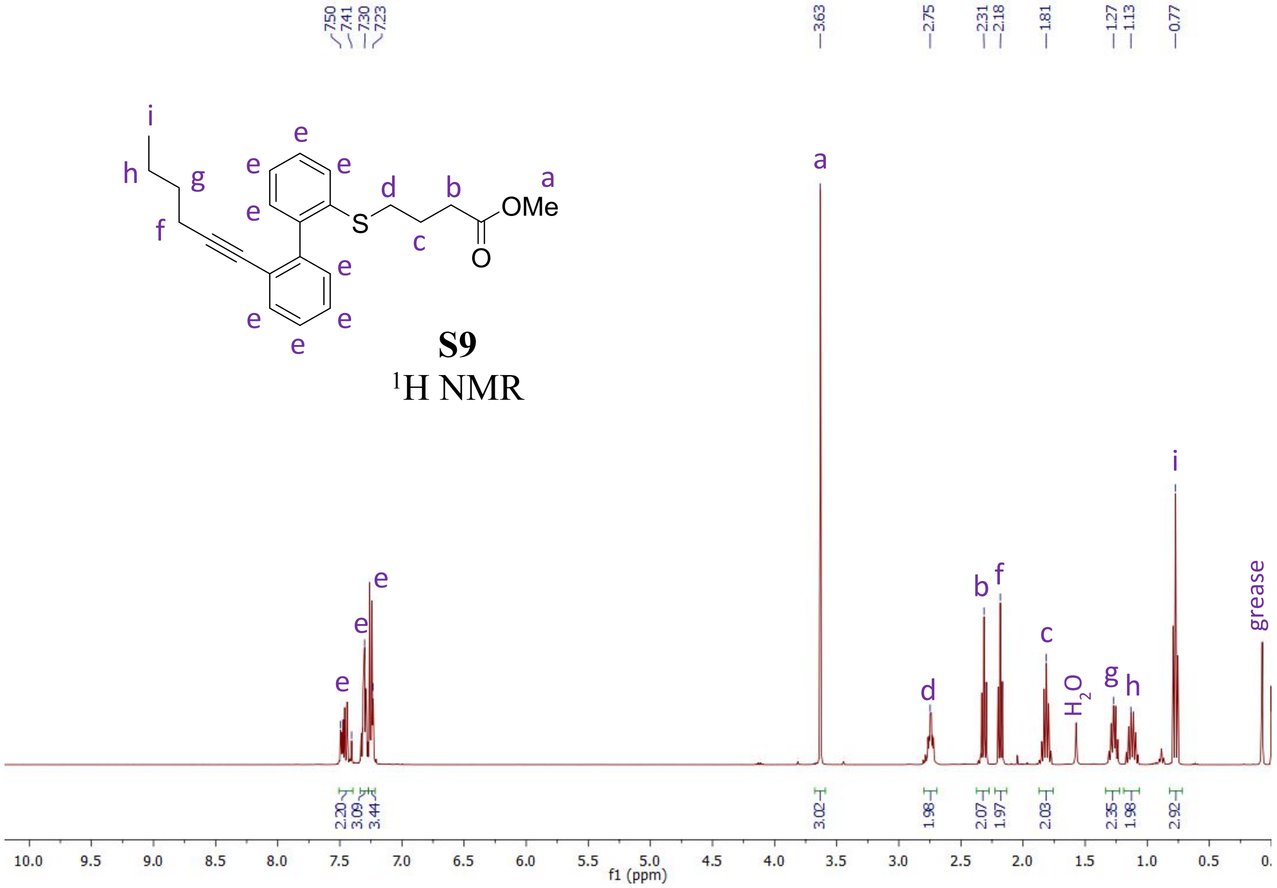

S9
^{1}H NMR
OMe
S
a
b
c
d
e
f
g
h
i
H$_2$O
grease
7.50
7.41
7.30
7.23
3.63
2.75
2.31
2.18
1.81
1.27
1.13
0.77
2.20
3.09
3.44
3.02
1.98
2.07
1.97
2.03
2.35
1.98
2.92
f1 (ppm)

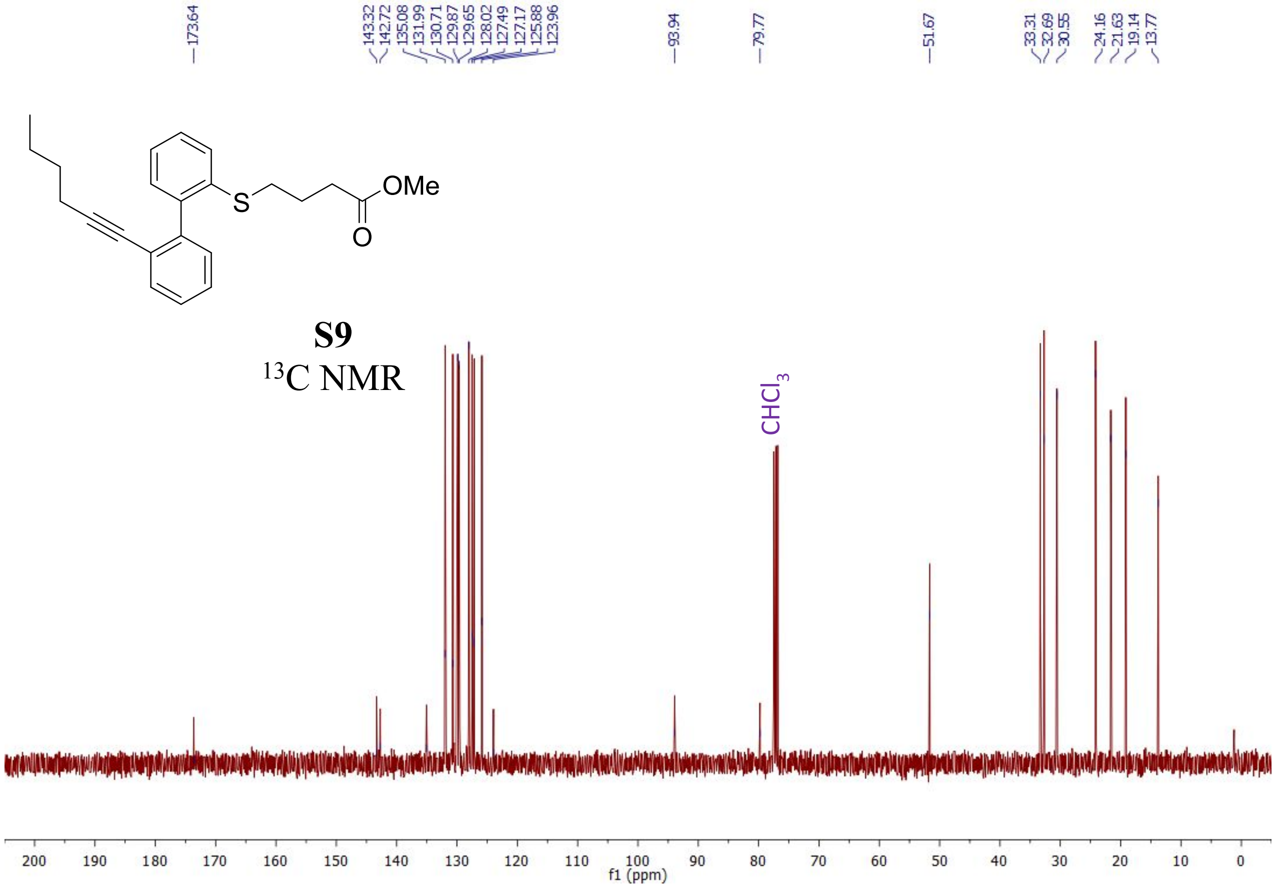

S9
^{13}C NMR
OMe
S
O
CHCl$_3$
173.64
143.32
142.72
135.08
131.99
130.71
129.87
129.65
128.02
127.49
127.17
125.88
123.96
93.94
79.77
51.67
33.31
32.69
30.55
24.16
21.63
19.14
13.77
f1 (ppm)

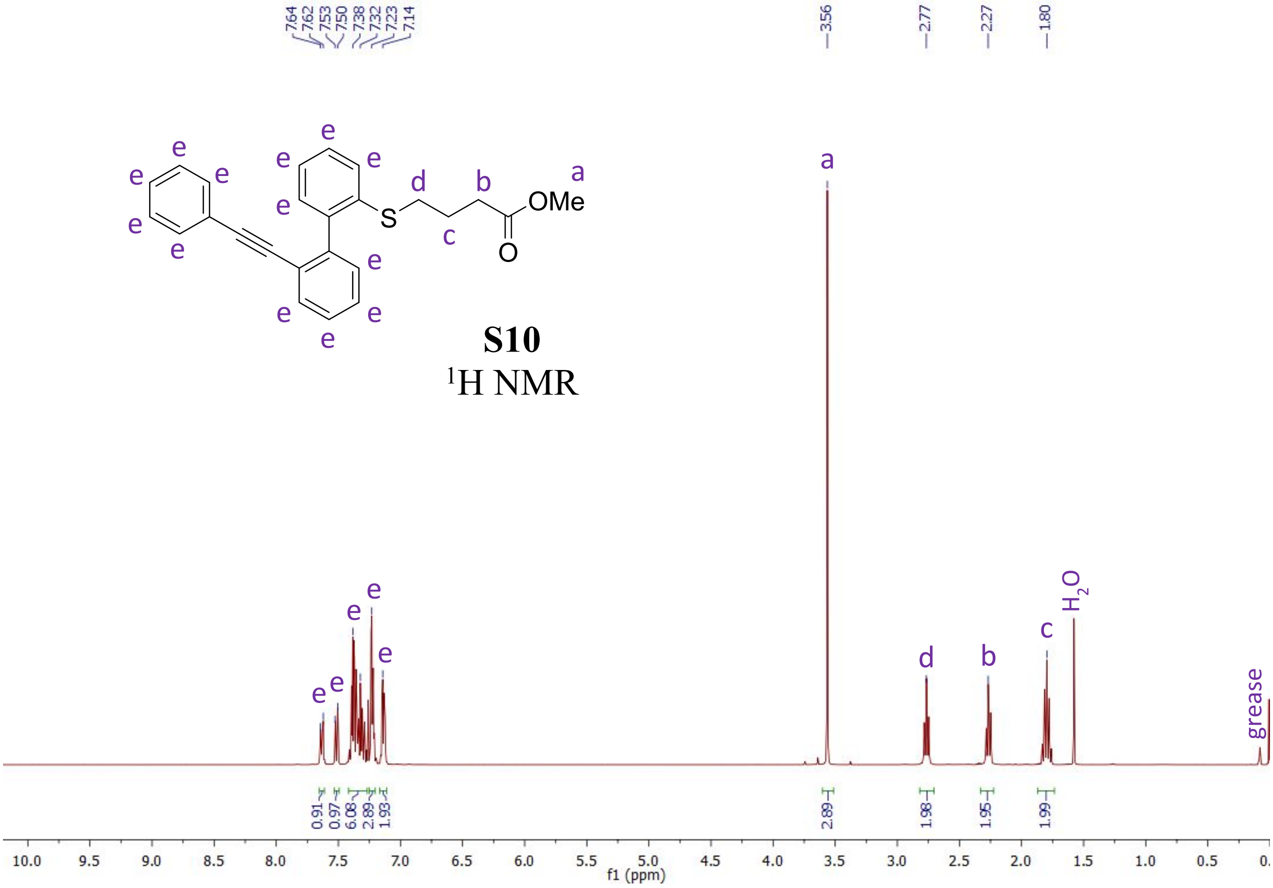
7.64
7.62
7.53
7.50
7.38
7.32
7.23
7.14
3.56
2.77
2.27
1.80
S10
^{1}H NMR
OMe
S
O
a
b
c
d
e
H_2O
grease
0.91
0.97
6.08
2.89
1.93
2.89
1.98
1.95
1.99
f1 (ppm)

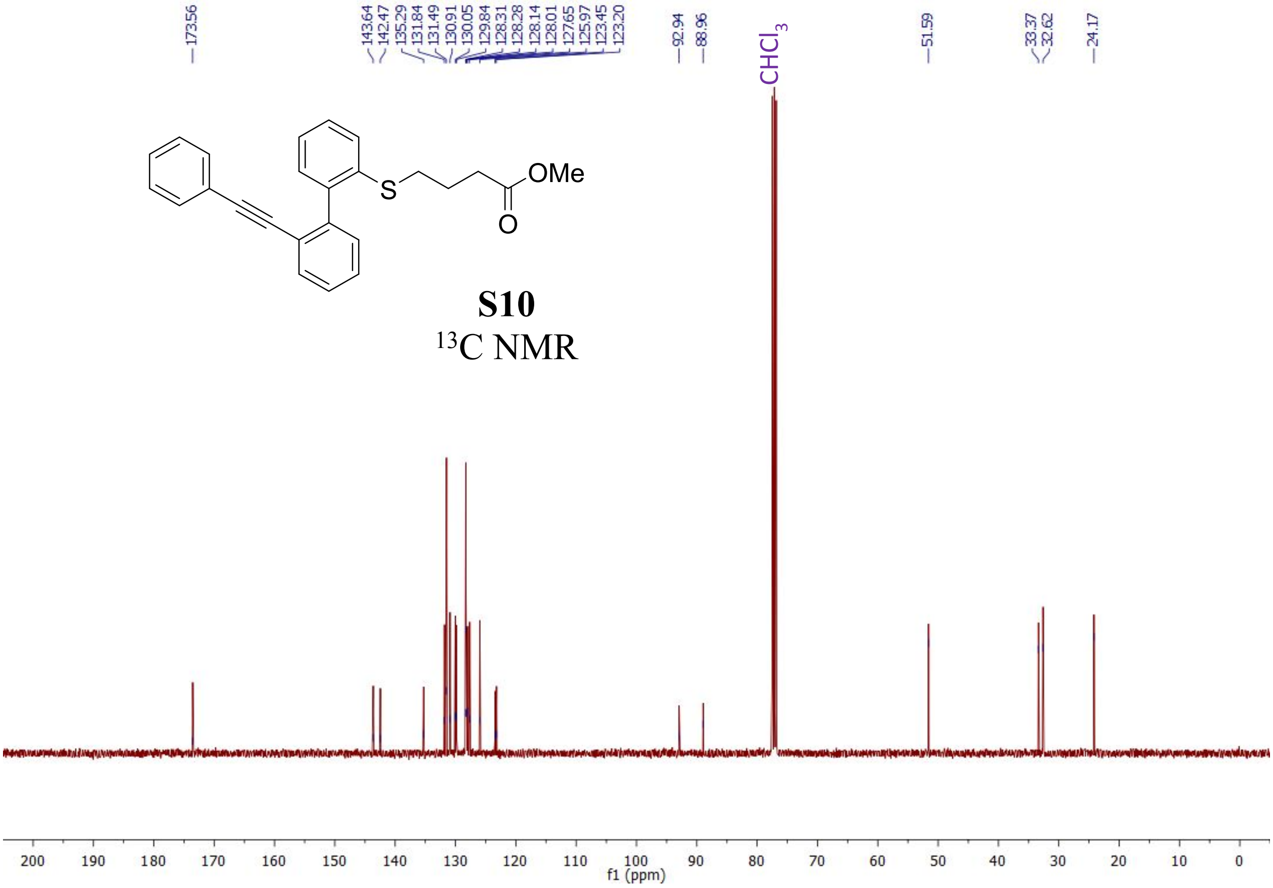
173.56
143.64
142.47
135.29
131.84
131.49
130.91
130.05
129.84
128.31
128.28
128.14
128.01
127.65
125.97
123.45
123.20
92.94
88.96
$CHCl_3$
51.59
33.37
32.62
24.17
S10
^{13}C NMR
OMe
S
O
f1 (ppm)

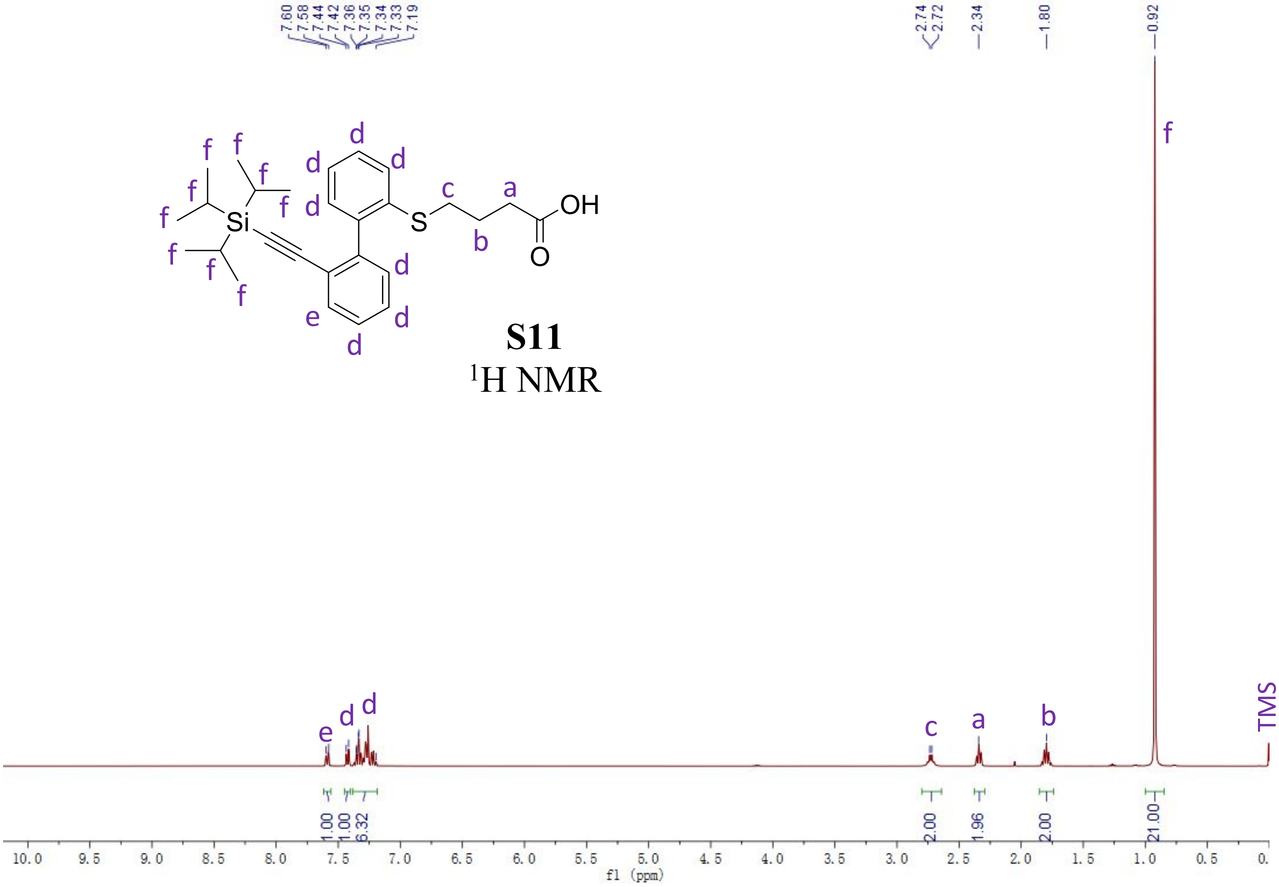

S11

^{1}H NMR

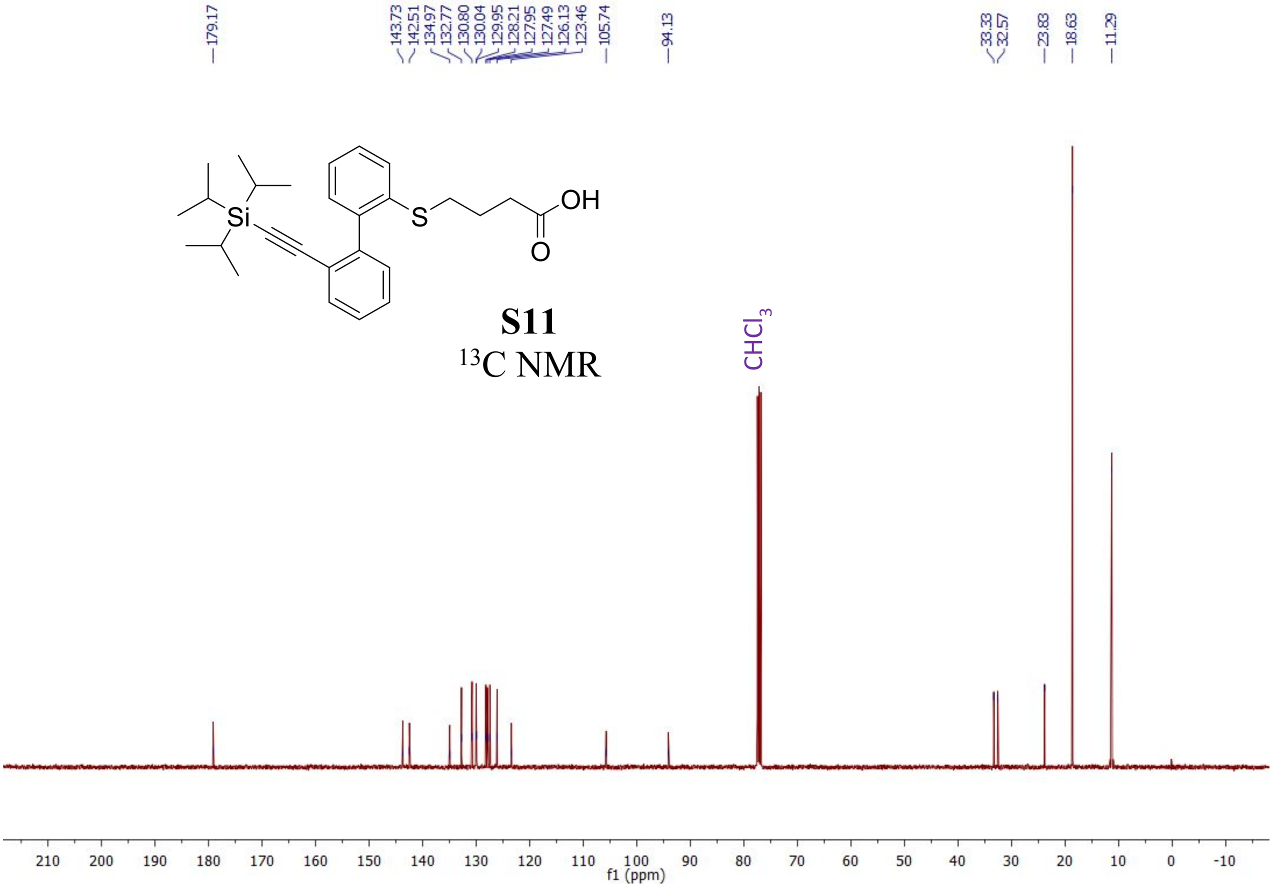

S11

^{13}C NMR

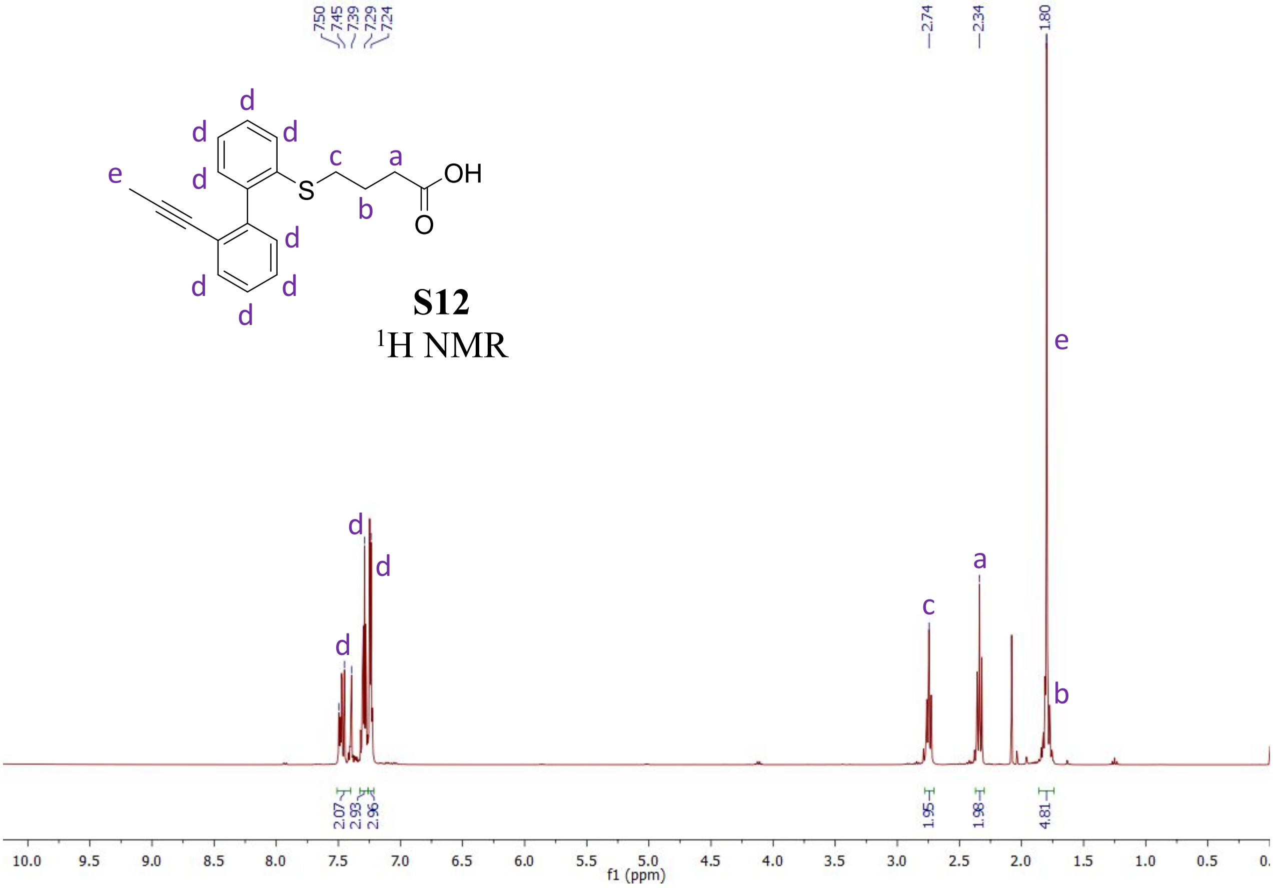

7.50
7.45
7.39
7.29
7.24
2.74
2.34
1.80
S12
1H NMR
OH
O
S
a
b
c
d
e
2.07
2.93
2.96
1.95
1.98
4.81
f1 (ppm)

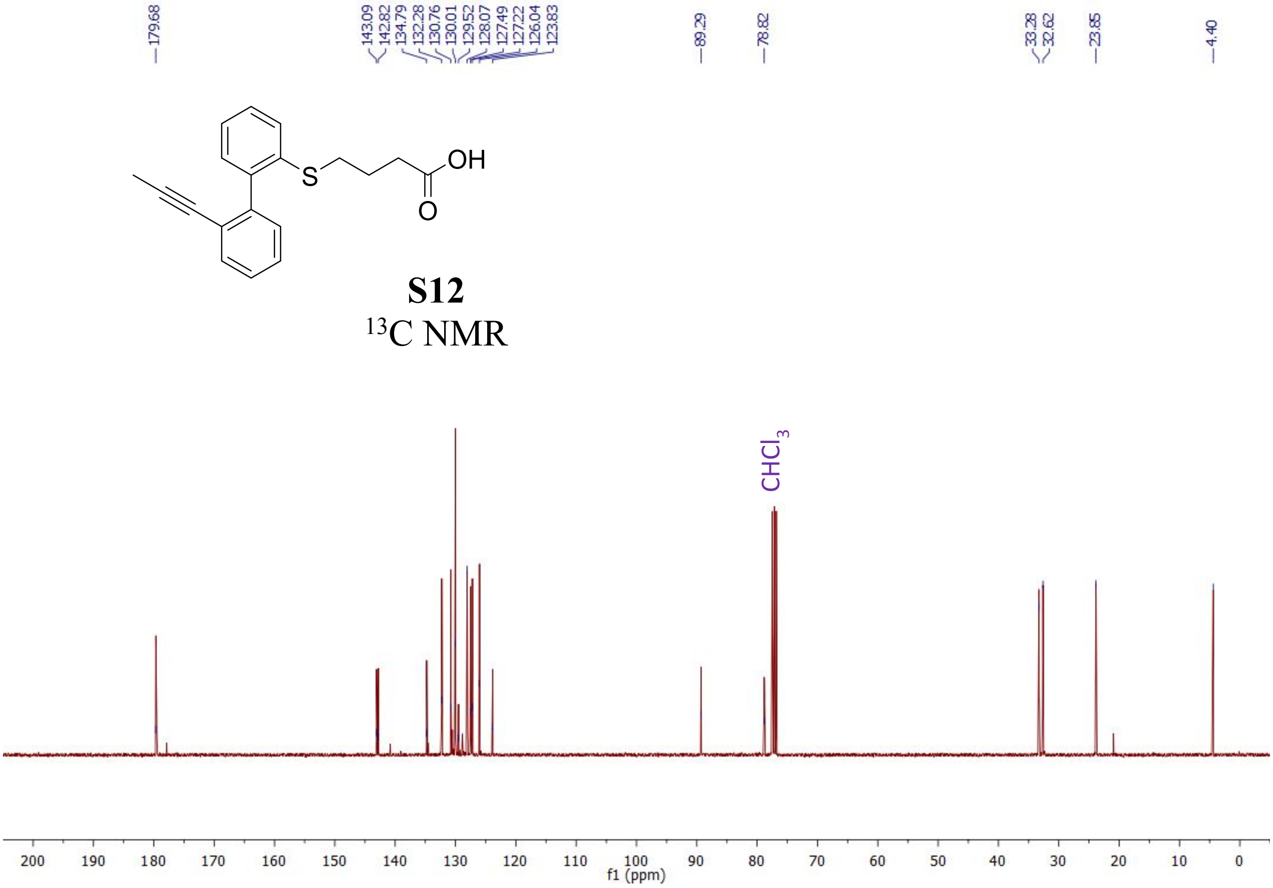

179.68
143.09
142.82
134.79
132.28
130.76
130.01
129.52
128.07
127.49
127.22
126.04
123.83
89.29
78.82
33.28
32.62
23.85
4.40
S12
13C NMR
OH
O
S
CHCl3
f1 (ppm)

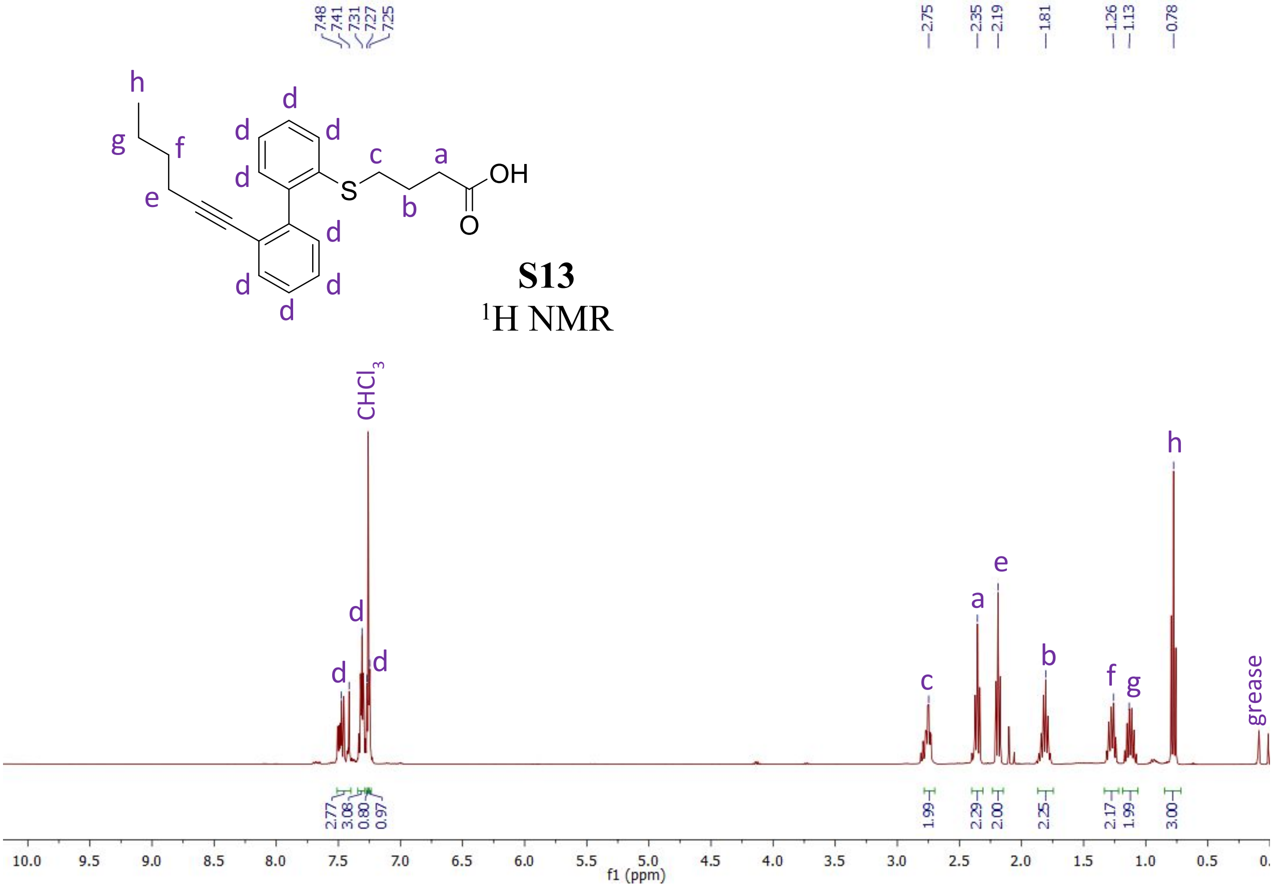
S13
1H NMR
7.48
7.41
7.31
7.27
7.25
2.75
2.35
2.19
1.81
1.26
1.13
0.78
CHCl3
grease
2.77
3.08
0.80
0.97
1.99
2.29
2.00
2.25
2.17
1.99
3.00
f1 (ppm)

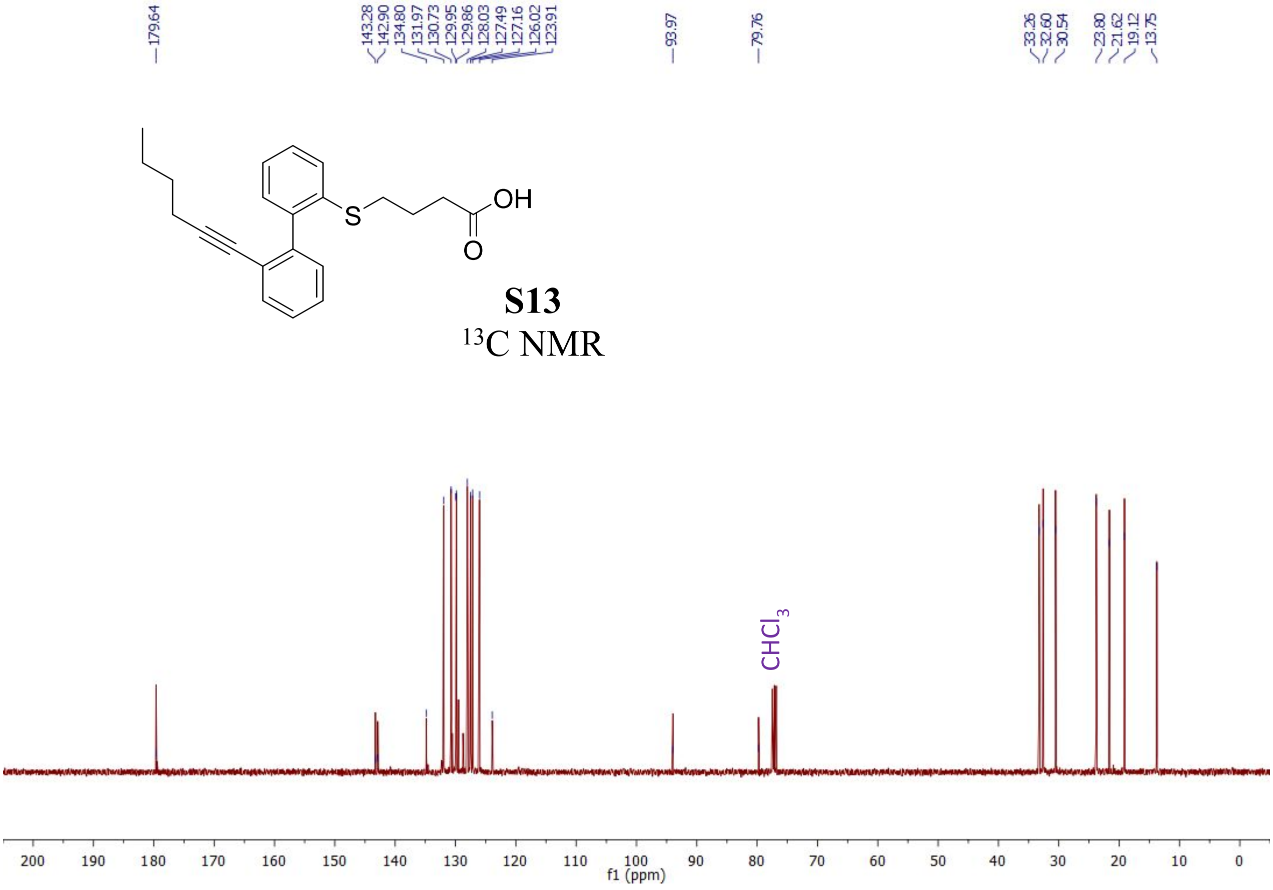
S13
13C NMR
179.64
143.28
142.90
134.80
131.97
130.73
129.95
129.86
128.03
127.49
127.16
126.02
123.91
93.97
79.76
33.26
32.60
30.54
23.80
21.62
19.12
13.75
CHCl3
f1 (ppm)

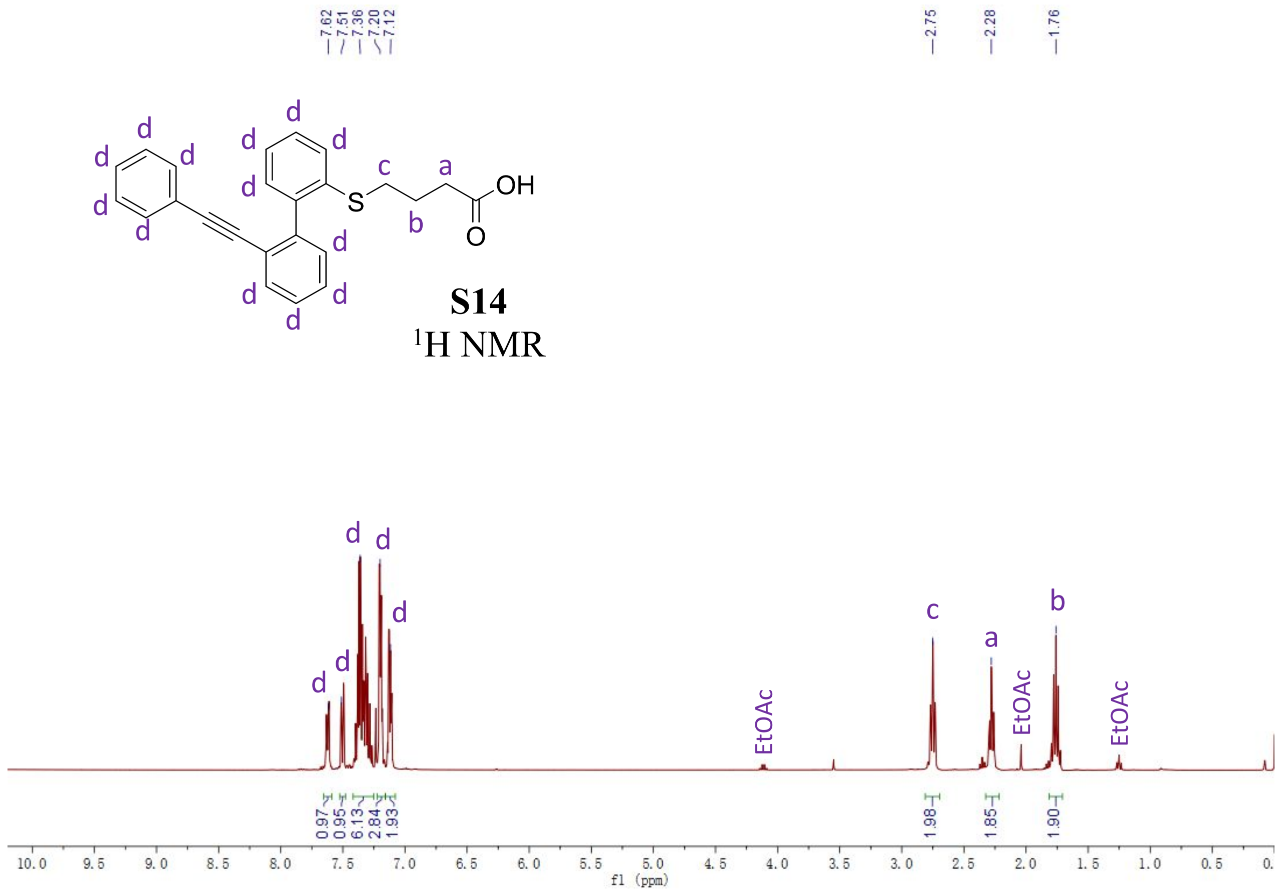
S14
1H NMR
EtOAc
EtOAc
EtOAc
f1 (ppm)

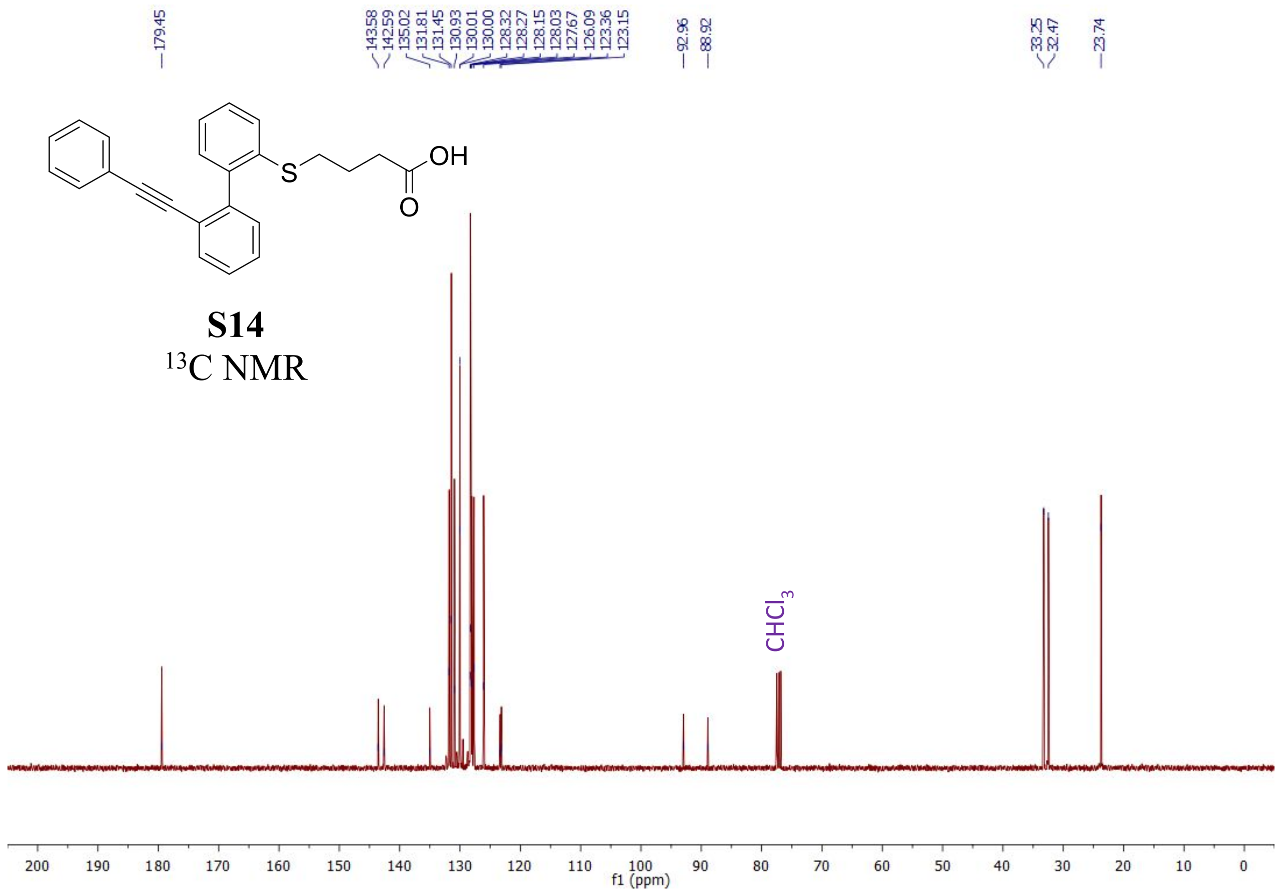
S14
13C NMR
CHCl3
f1 (ppm)

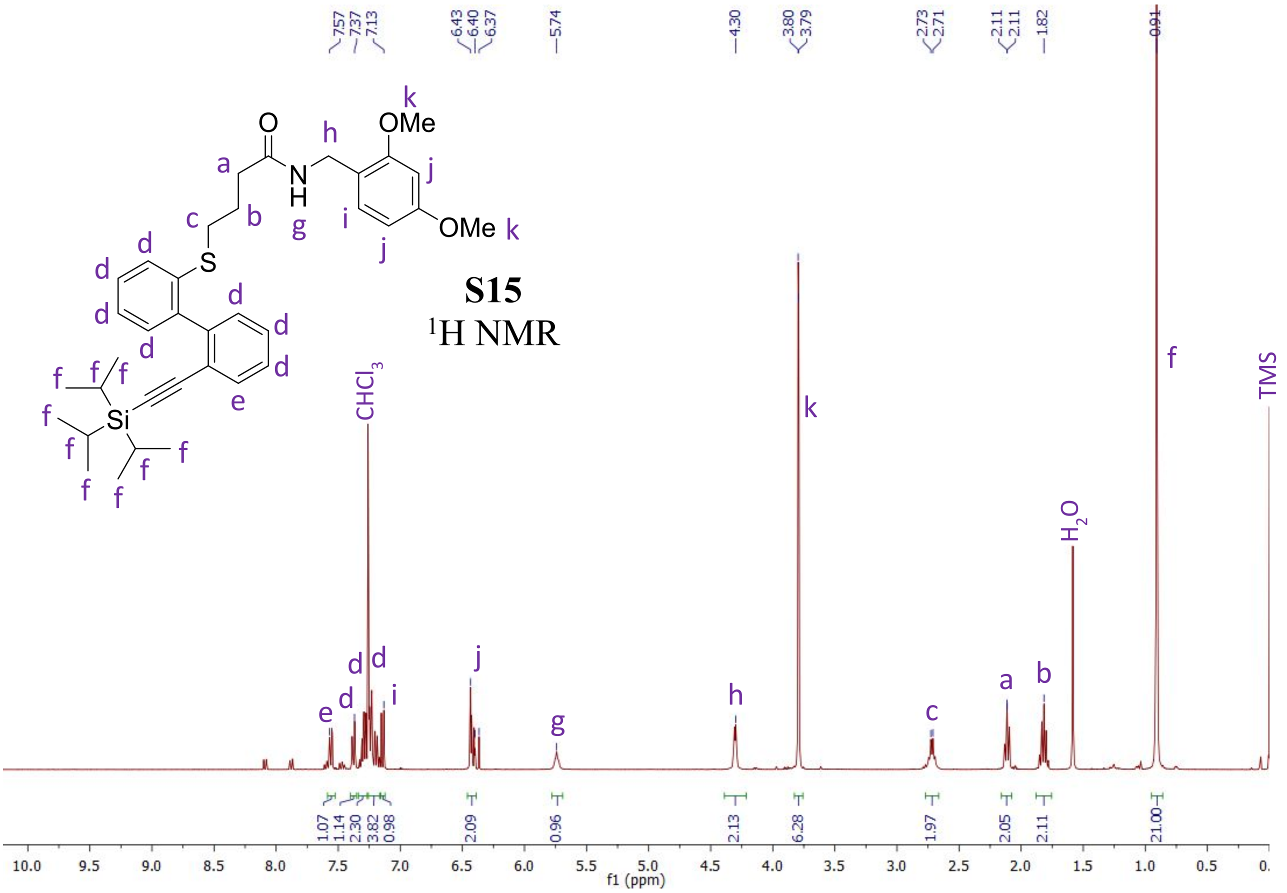
S15
^{1}H NMR
OMe
OMe
NH
O
S
Si
CHCl3
H2O
TMS
f1 (ppm)

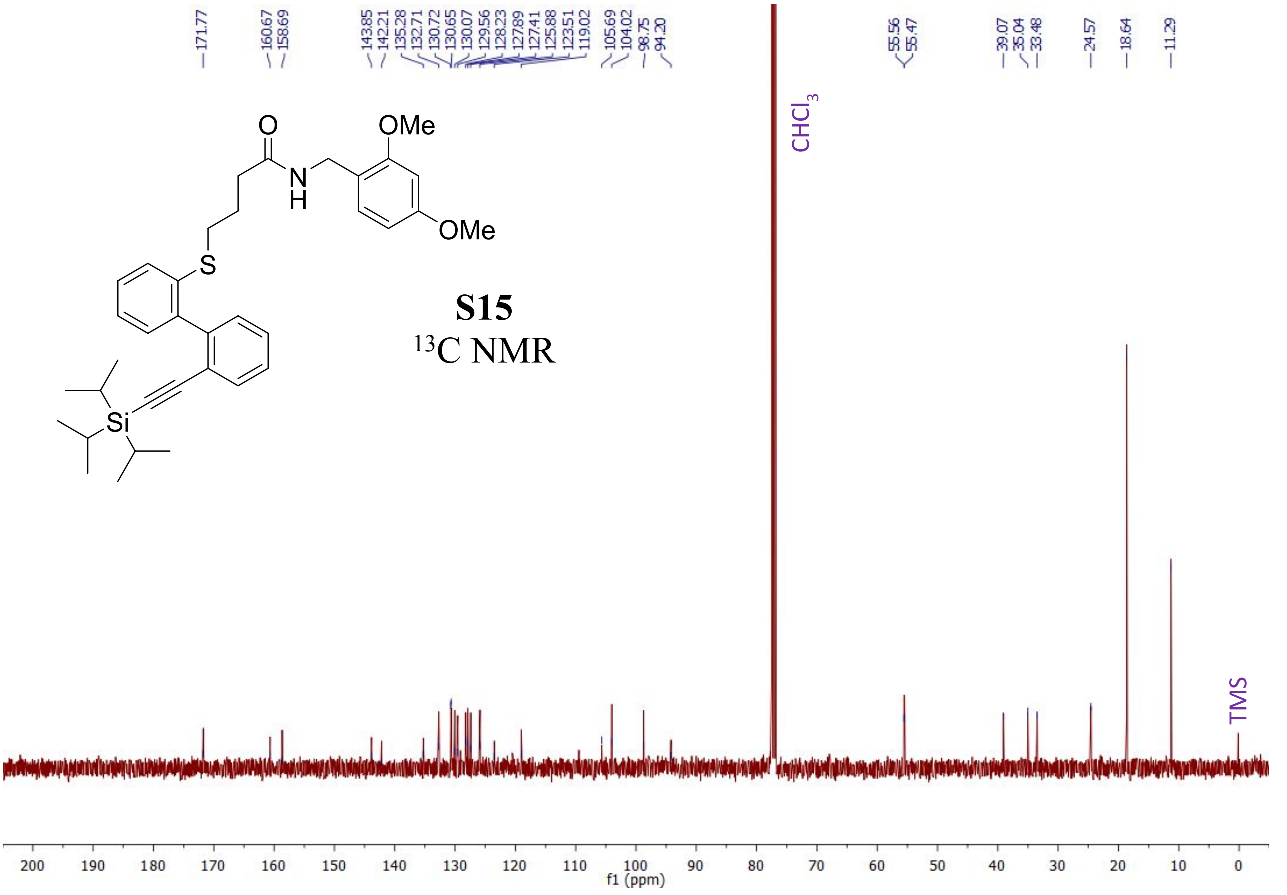
S15
^{13}C NMR
OMe
OMe
NH
O
S
Si
CHCl3
TMS
f1 (ppm)

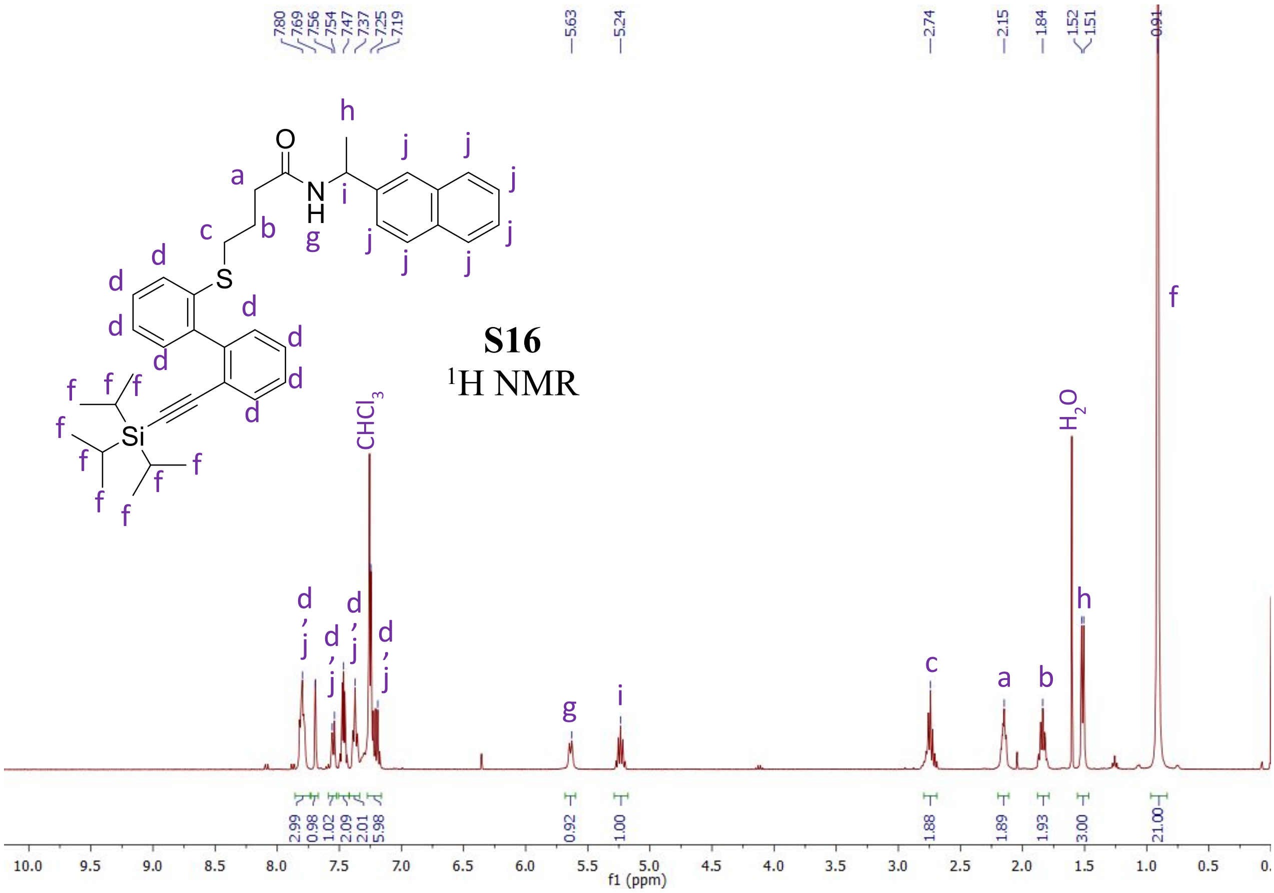

S16
^{1}H NMR

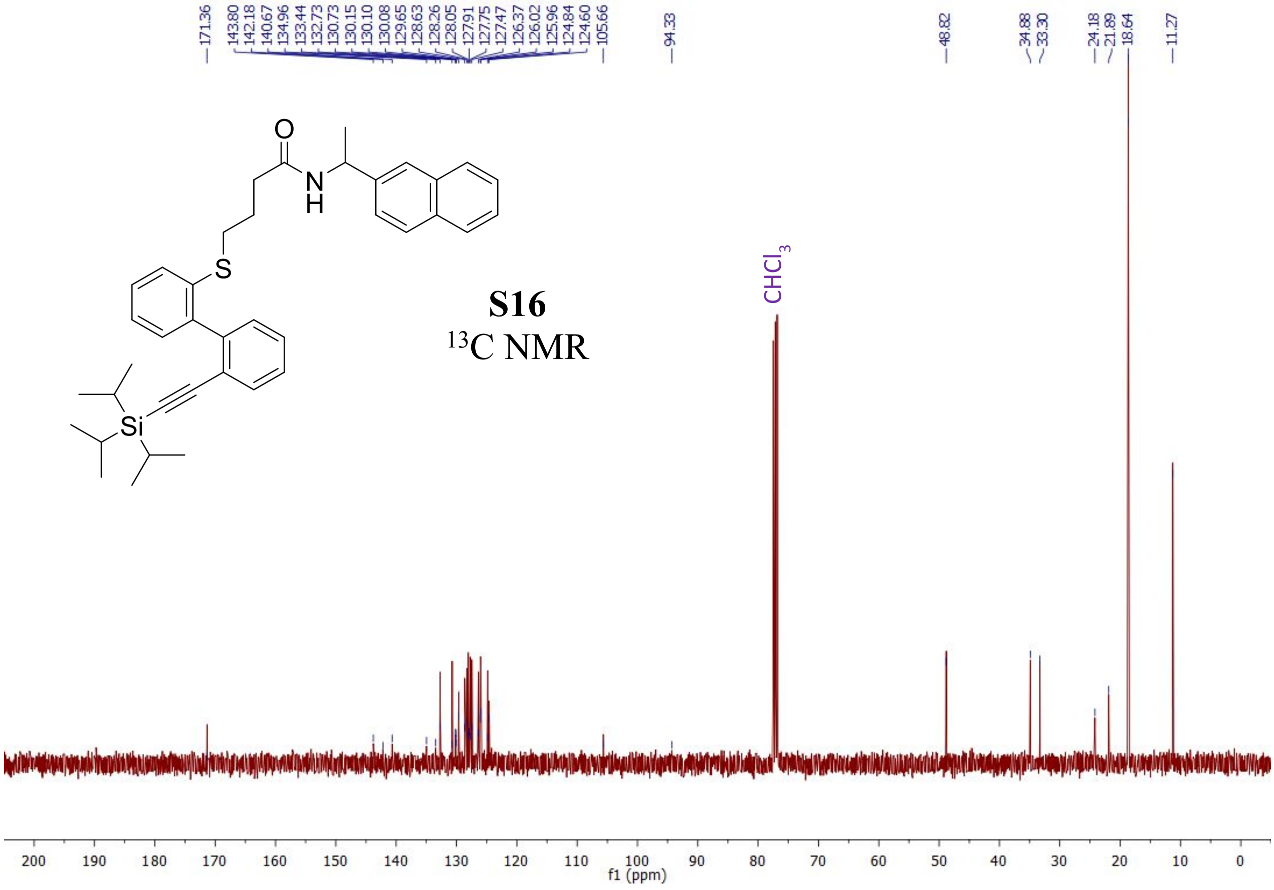

S16
^{13}C NMR

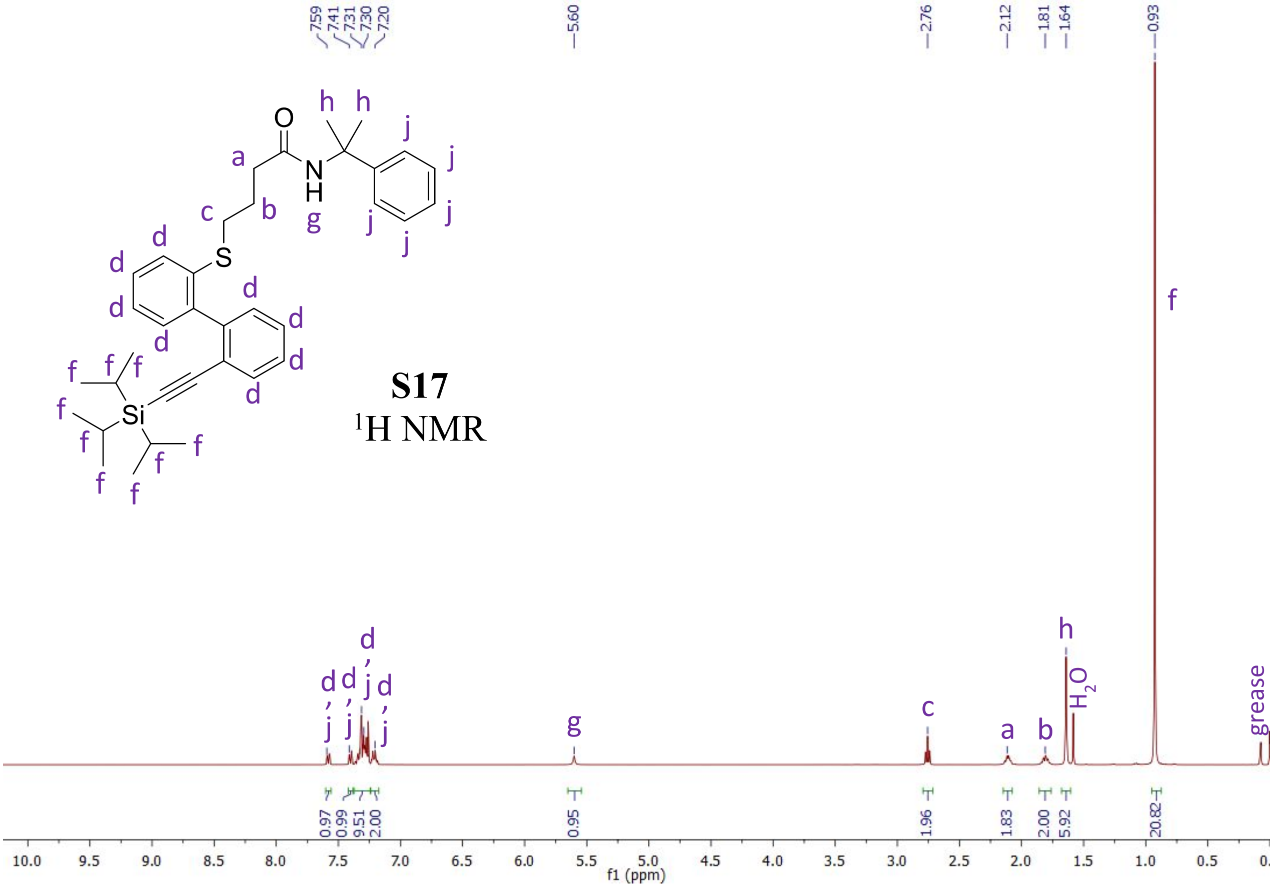
7.59
7.41
7.31
7.30
7.20
5.60
2.76
2.12
1.81
1.64
0.93
S17
^{1}H NMR
0.97
0.99
9.51
2.00
0.95
1.96
1.83
2.00
5.92
20.82
f1 (ppm)

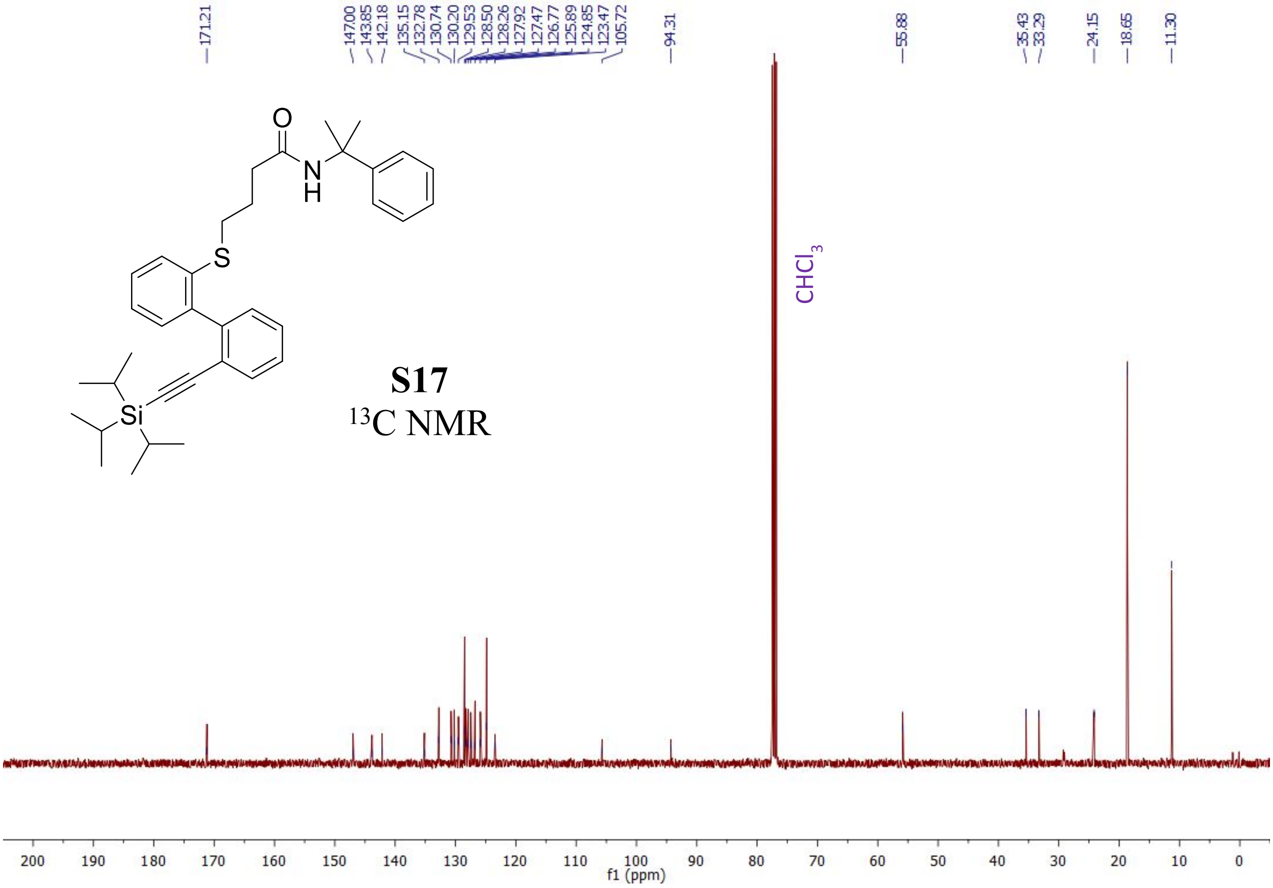
171.21
147.00
143.85
142.18
135.15
132.78
130.74
130.20
129.53
128.50
128.26
127.92
127.47
126.77
125.89
124.85
123.47
105.72
94.31
55.88
35.43
33.29
24.15
18.65
11.30
S17
^{13}C NMR
$CHCl_3$
f1 (ppm)

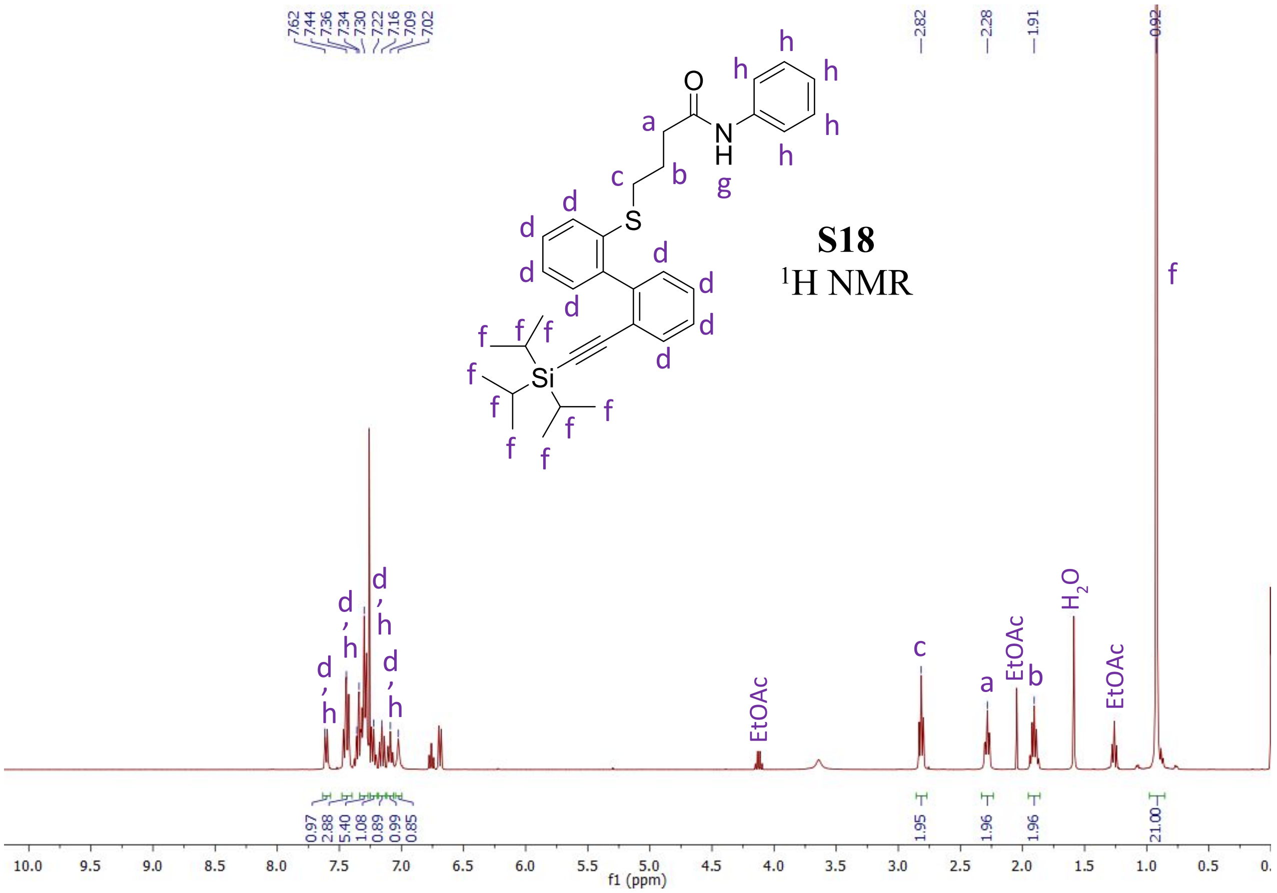
7.62
7.44
7.36
7.34
7.30
7.22
7.16
7.09
7.02
2.82
2.28
1.91
0.92
O
N
H
S
Si
a b c d f g h
S18
^{1}H NMR
EtOAc
H_2O
0.97
2.88
5.40
1.08
0.89
0.99
0.85
1.95
1.96
1.96
21.00
f1 (ppm)

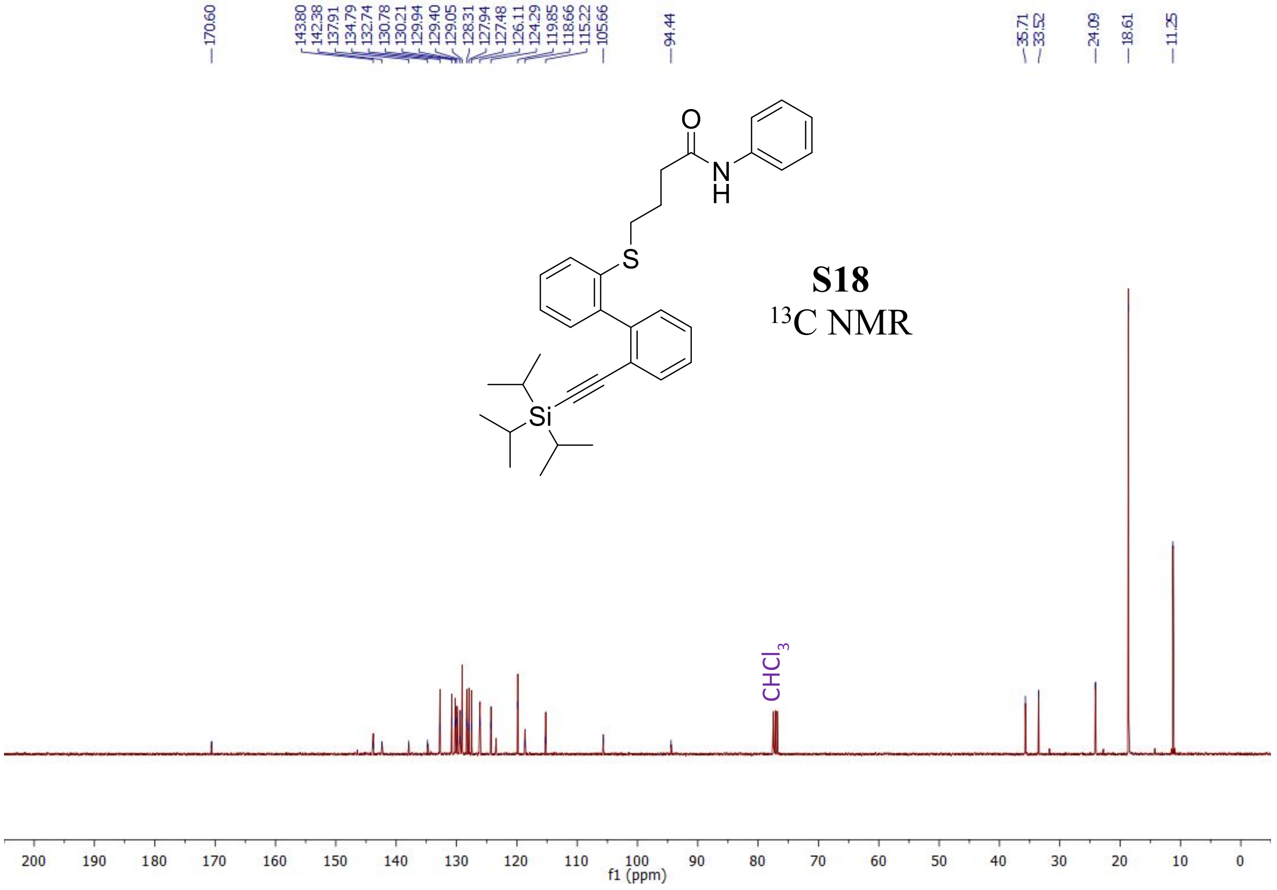
170.60
143.80
142.38
137.91
134.79
132.74
130.78
130.21
129.94
129.40
129.05
128.31
127.94
127.48
126.11
124.29
119.85
118.66
115.22
105.66
94.44
35.71
33.52
24.09
18.61
11.25
O
N
H
S
Si
S18
^{13}C NMR
$CHCl_3$
f1 (ppm)

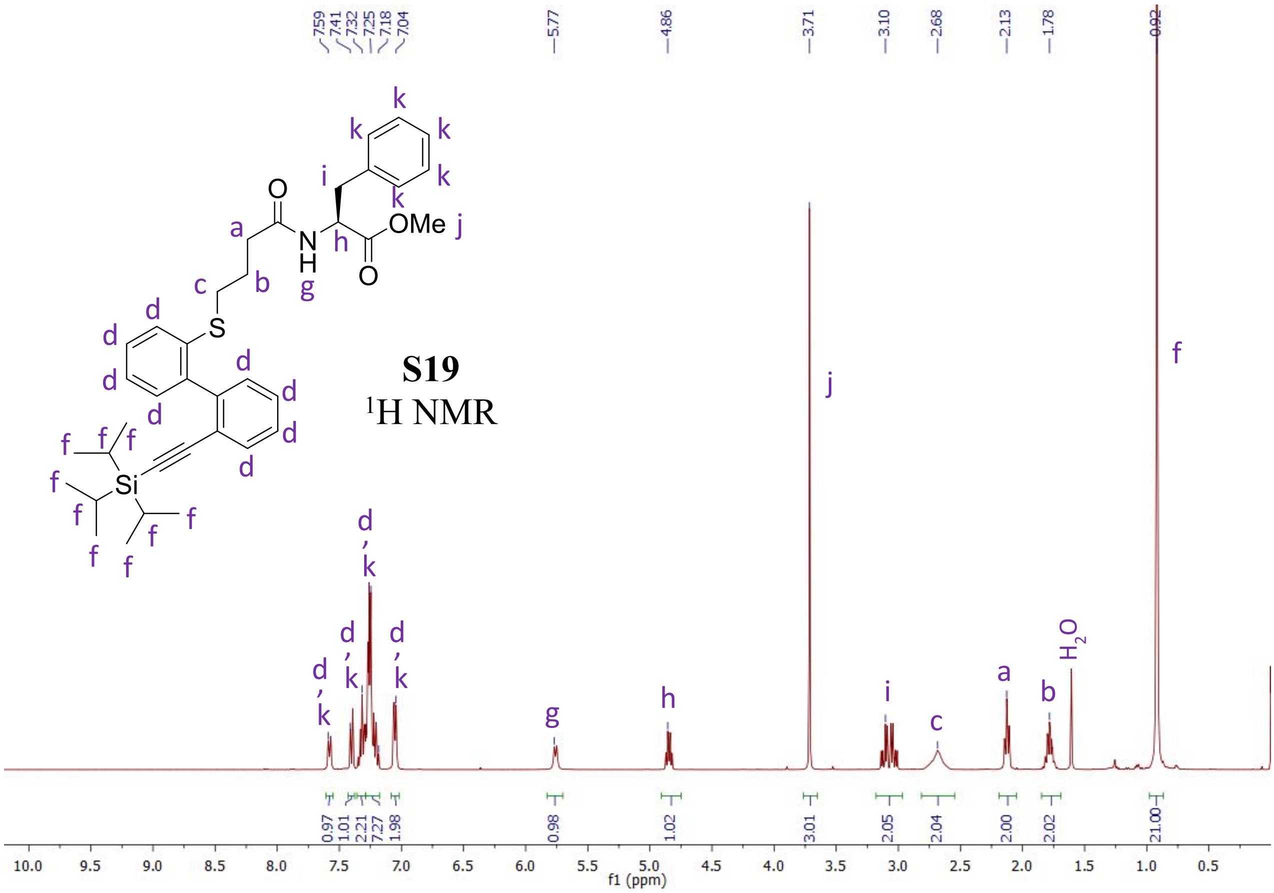
7.59
7.41
7.32
7.25
7.18
7.04
5.77
4.86
3.71
3.10
2.68
2.13
1.78
0.92
S19
^{1}H NMR
0.97
1.01
2.21
7.27
1.98
0.98
1.02
3.01
2.05
2.04
2.00
2.02
21.00
f1 (ppm)

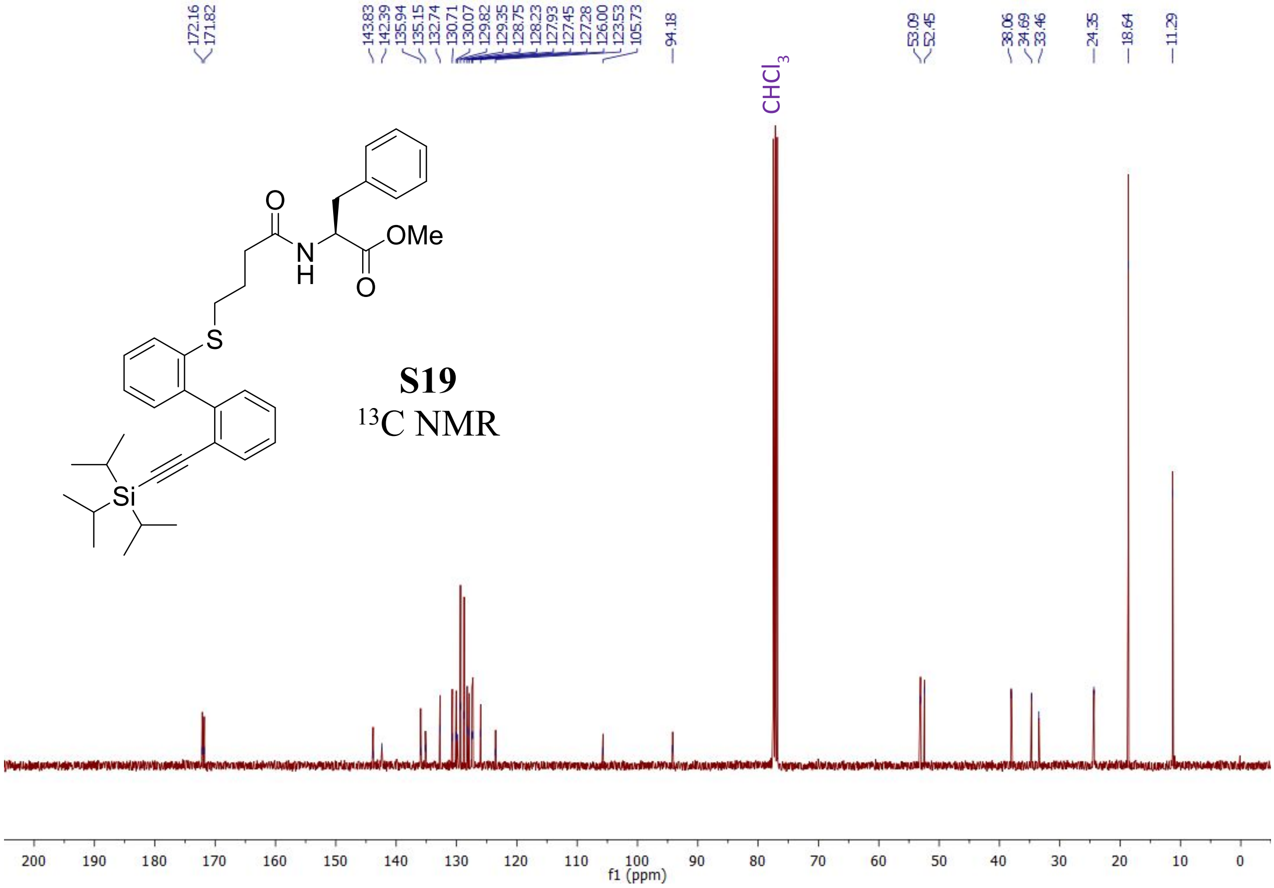
172.16
171.82
143.83
142.39
135.94
135.15
132.74
130.71
130.07
129.82
129.35
128.75
128.23
127.93
127.45
127.28
126.00
123.53
105.73
94.18
53.09
52.45
38.06
34.69
33.46
24.35
18.64
11.29
$CHCl_3$
S19
^{13}C NMR
f1 (ppm)

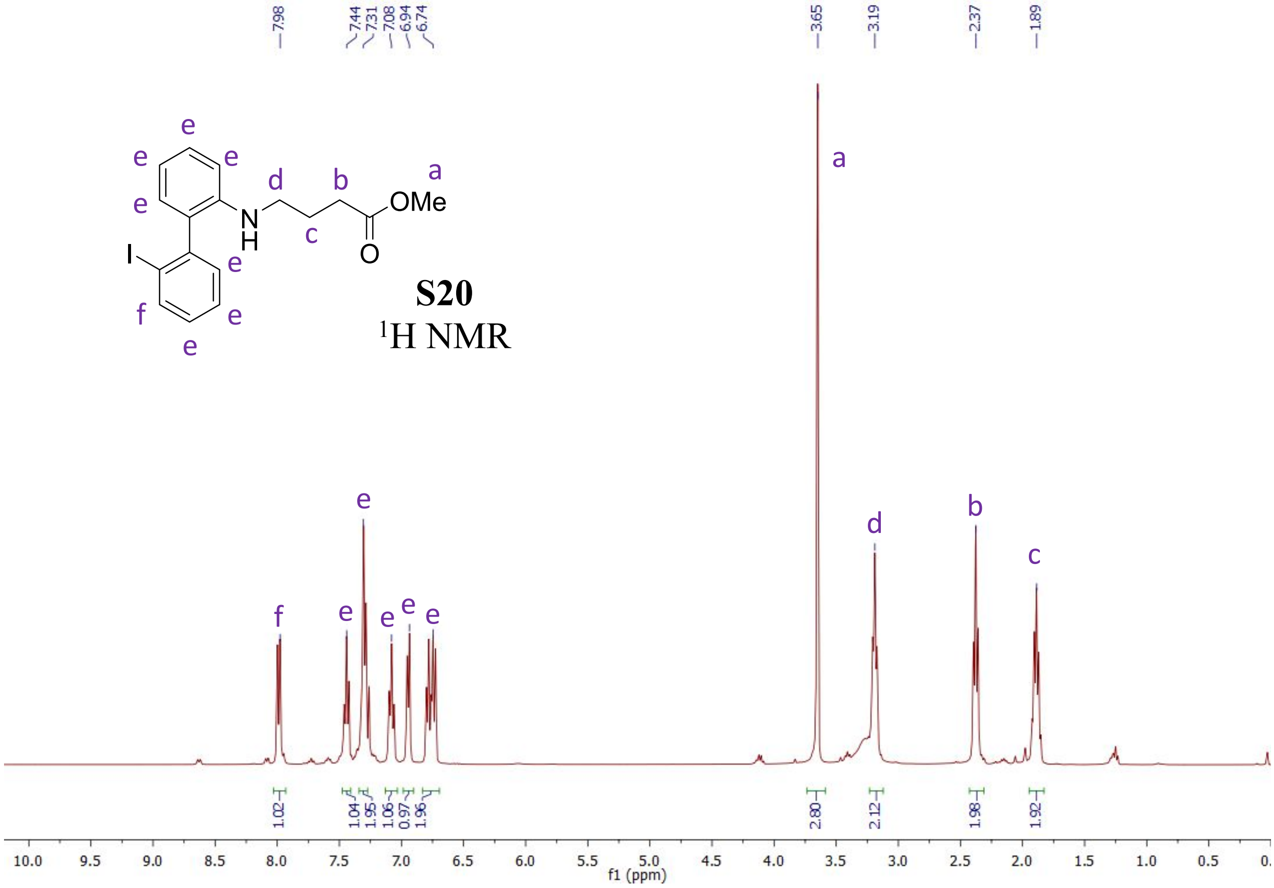

S20
^{1}H NMR

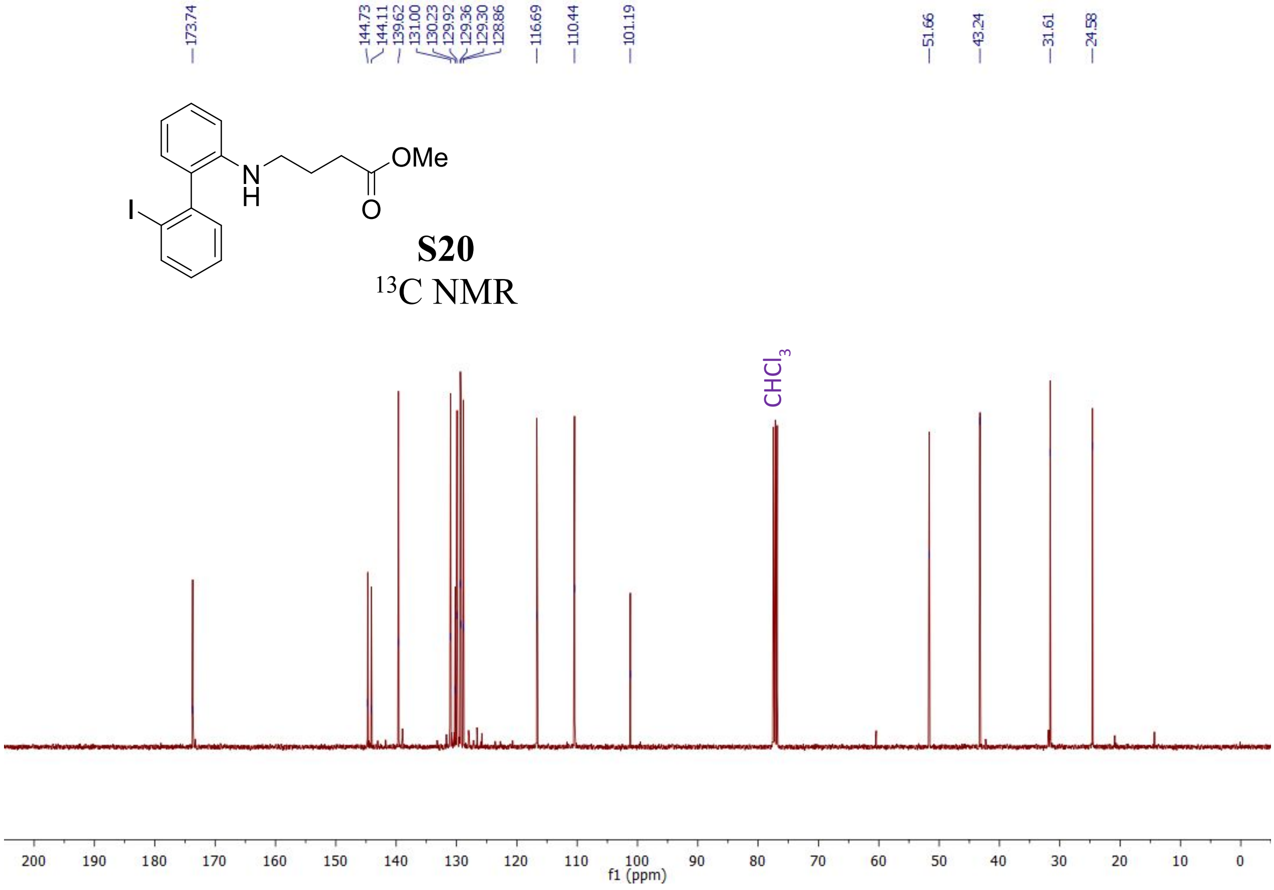

S20
^{13}C NMR

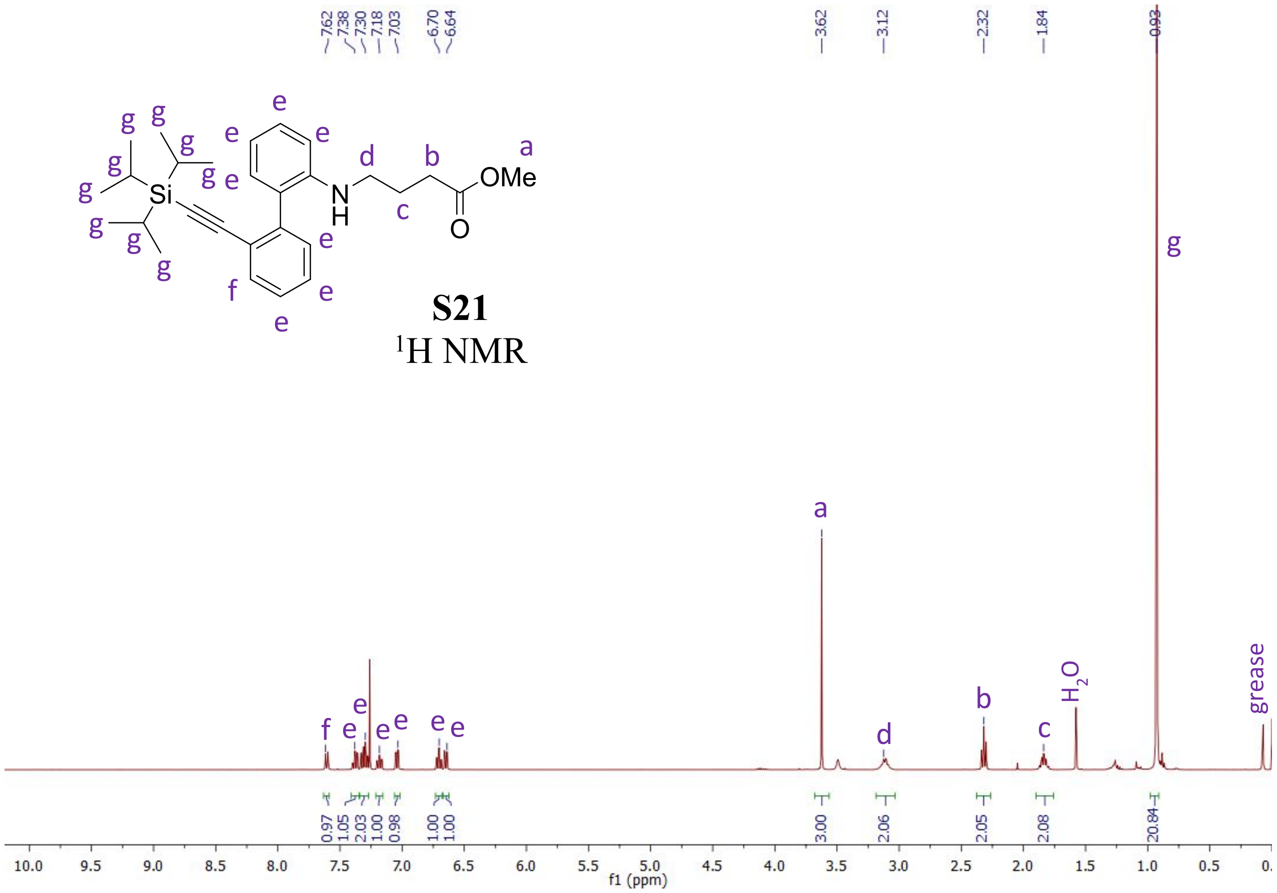
S21
1H NMR
7.62
7.38
7.30
7.18
7.03
6.70
6.64
3.62
3.12
2.32
1.84
0.93
a
b
c
d
e
f
g
H2O
grease
0.97
1.05
2.03
1.00
0.98
1.00
1.00
3.00
2.06
2.05
2.08
20.84
f1 (ppm)

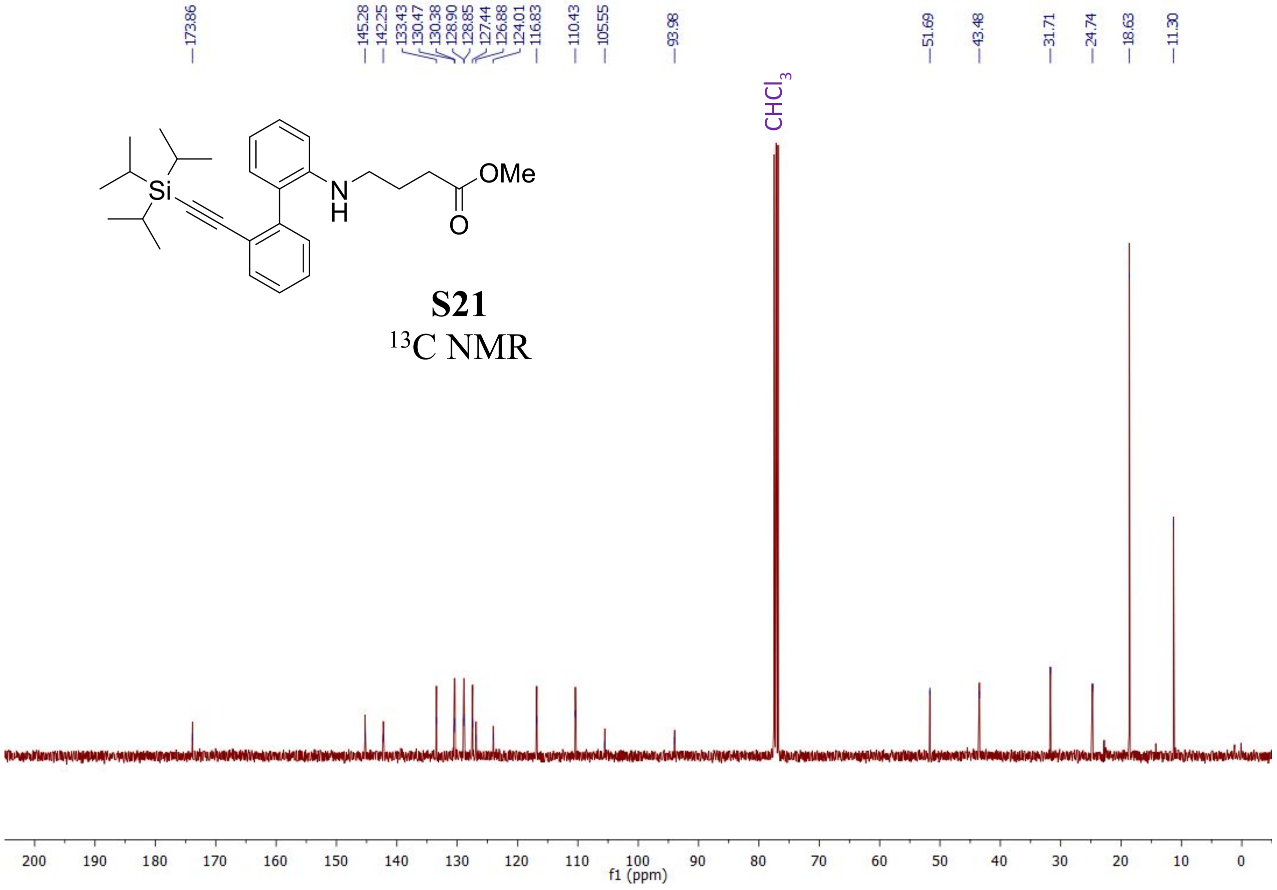
S21
13C NMR
173.86
145.28
142.25
133.43
130.47
130.38
128.90
128.85
127.44
126.88
124.01
116.83
110.43
105.55
93.98
51.69
43.48
31.71
24.74
18.63
11.30
CHCl3
f1 (ppm)

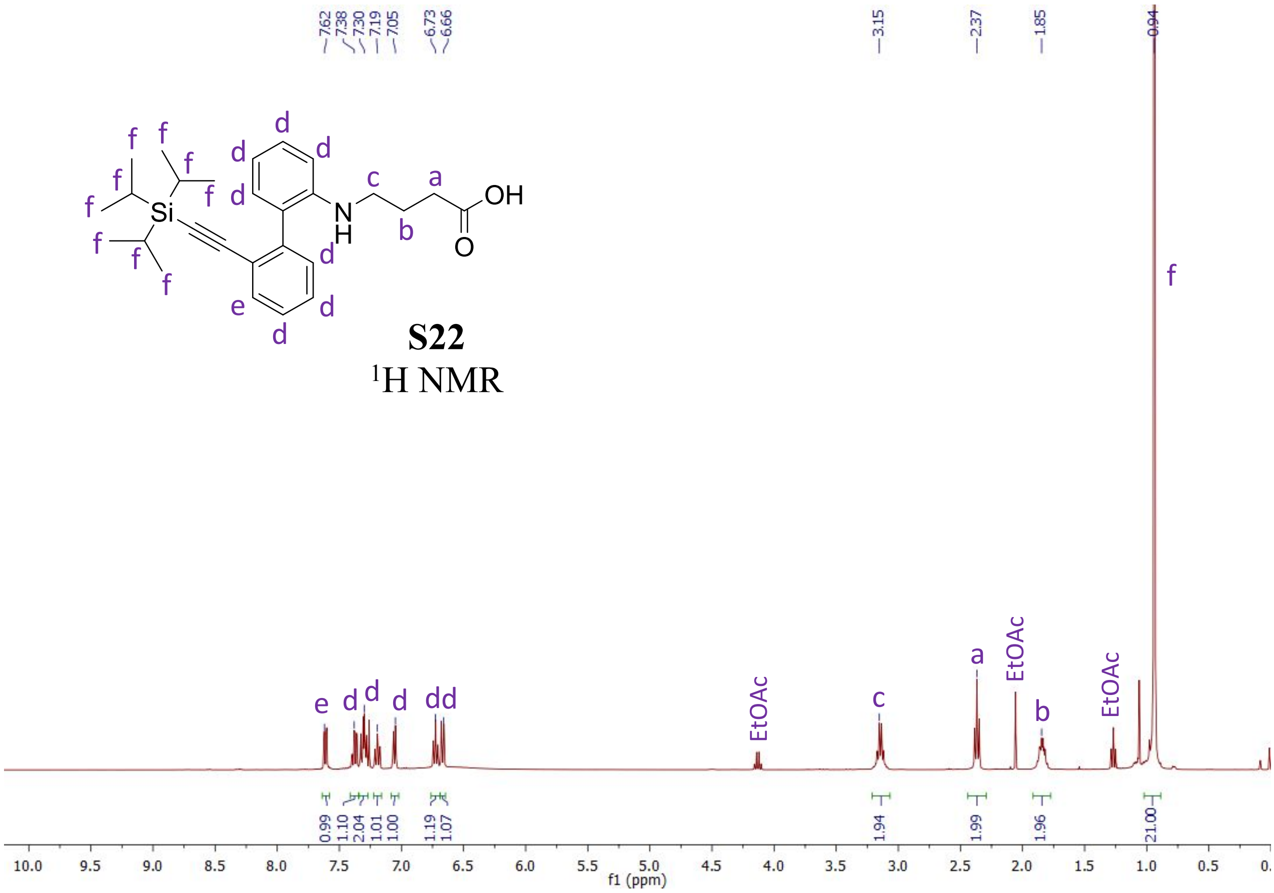

S22
^{1}H NMR

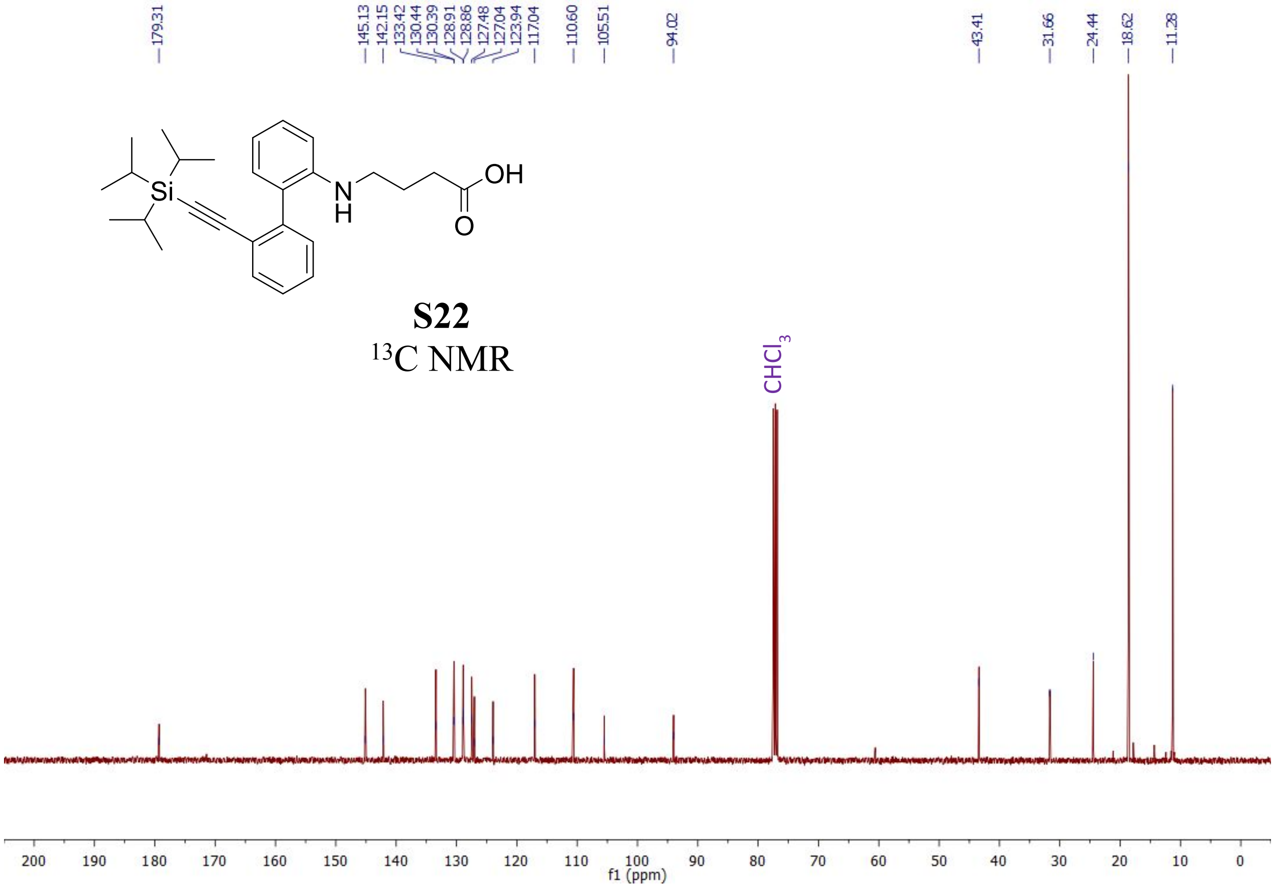

S22
^{13}C NMR

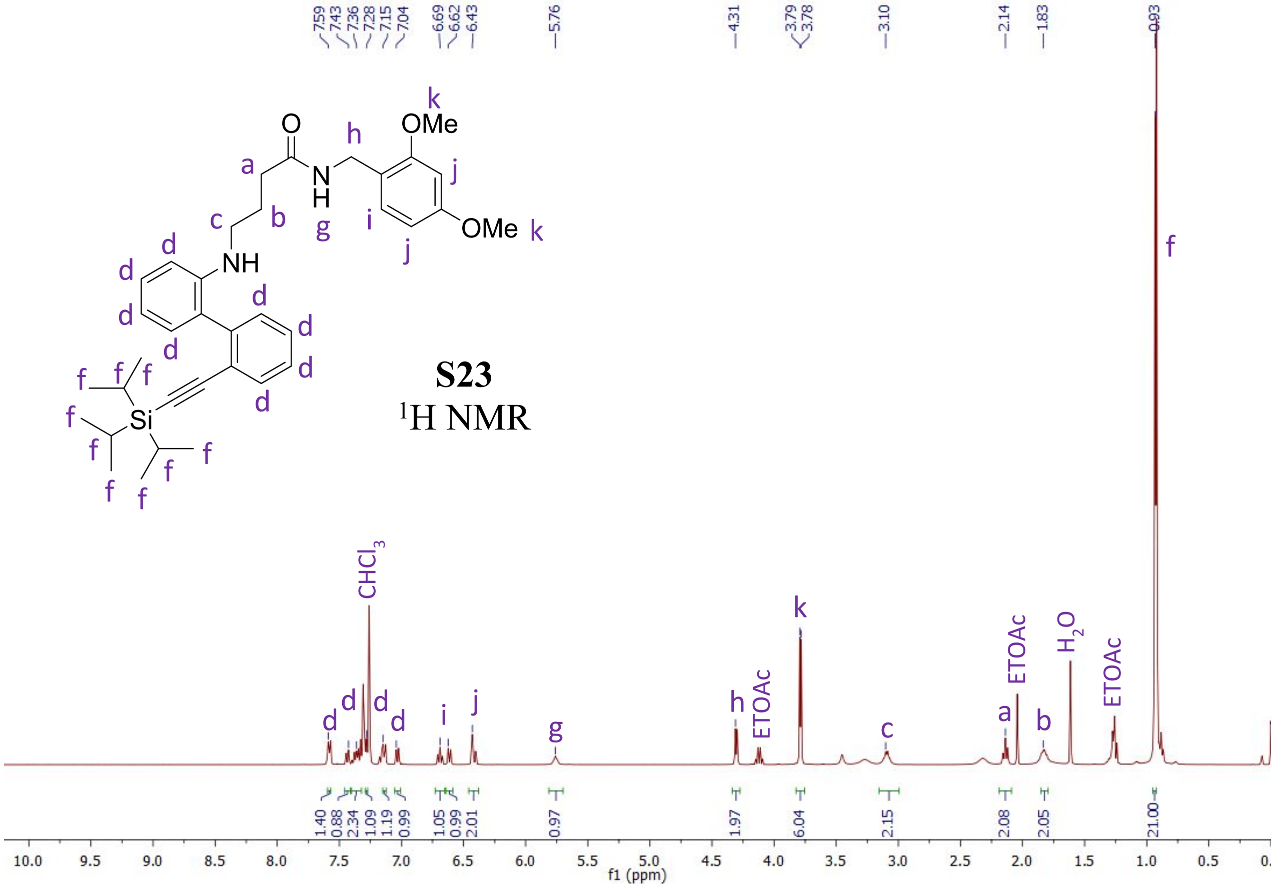
S23
¹H NMR
CHCl3
ETOAc
H2O
f (ppm)

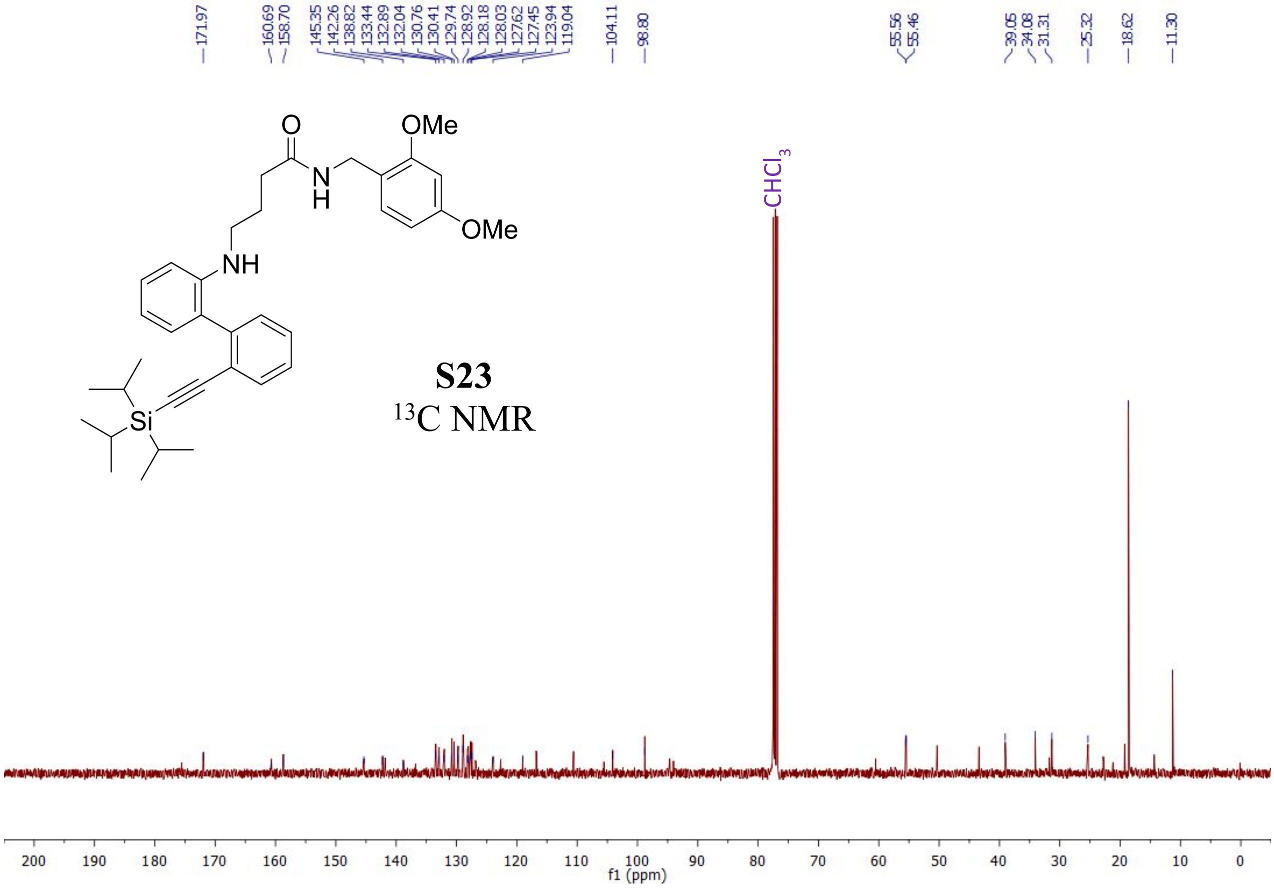
S23
¹³C NMR
CHCl3
f1 (ppm)

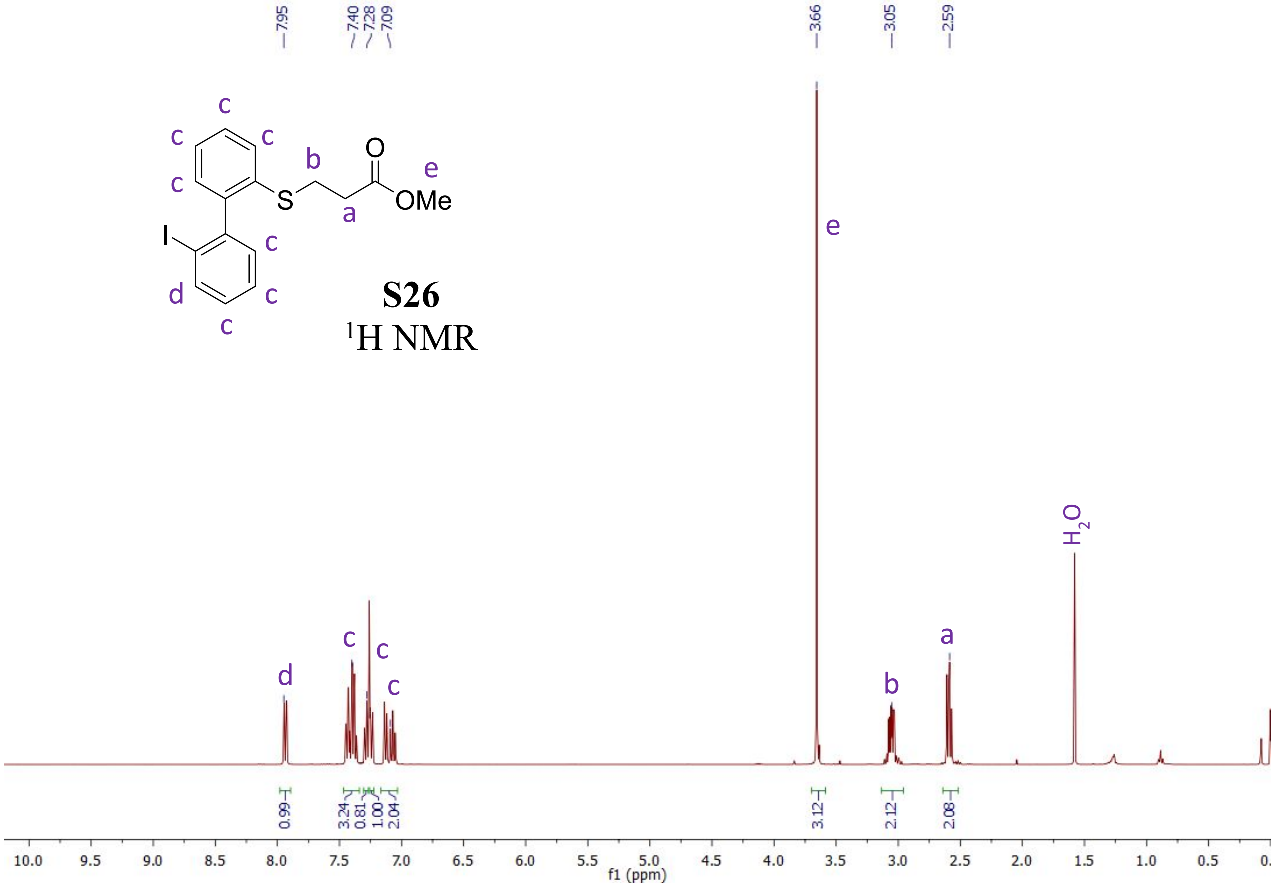
7.95
7.40
7.28
7.09
3.66
3.05
2.59
S26
1H NMR
OMe
H2O
0.99
3.24
0.81
1.00
2.04
3.12
2.12
2.08
f1 (ppm)

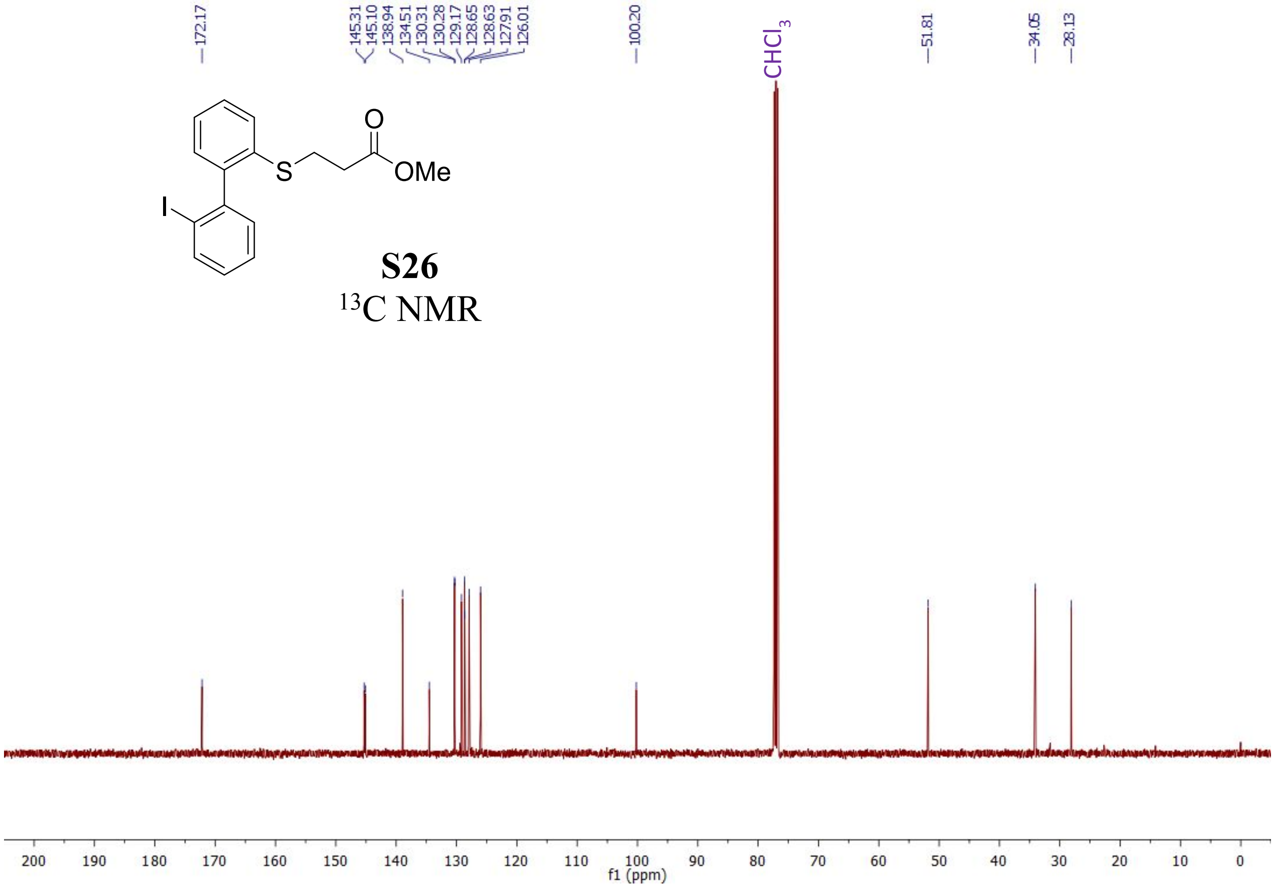
172.17
145.31
145.10
138.94
134.51
130.31
130.28
129.17
128.65
128.63
127.91
126.01
100.20
CHCl3
51.81
34.05
28.13
S26
13C NMR
OMe
f1 (ppm)

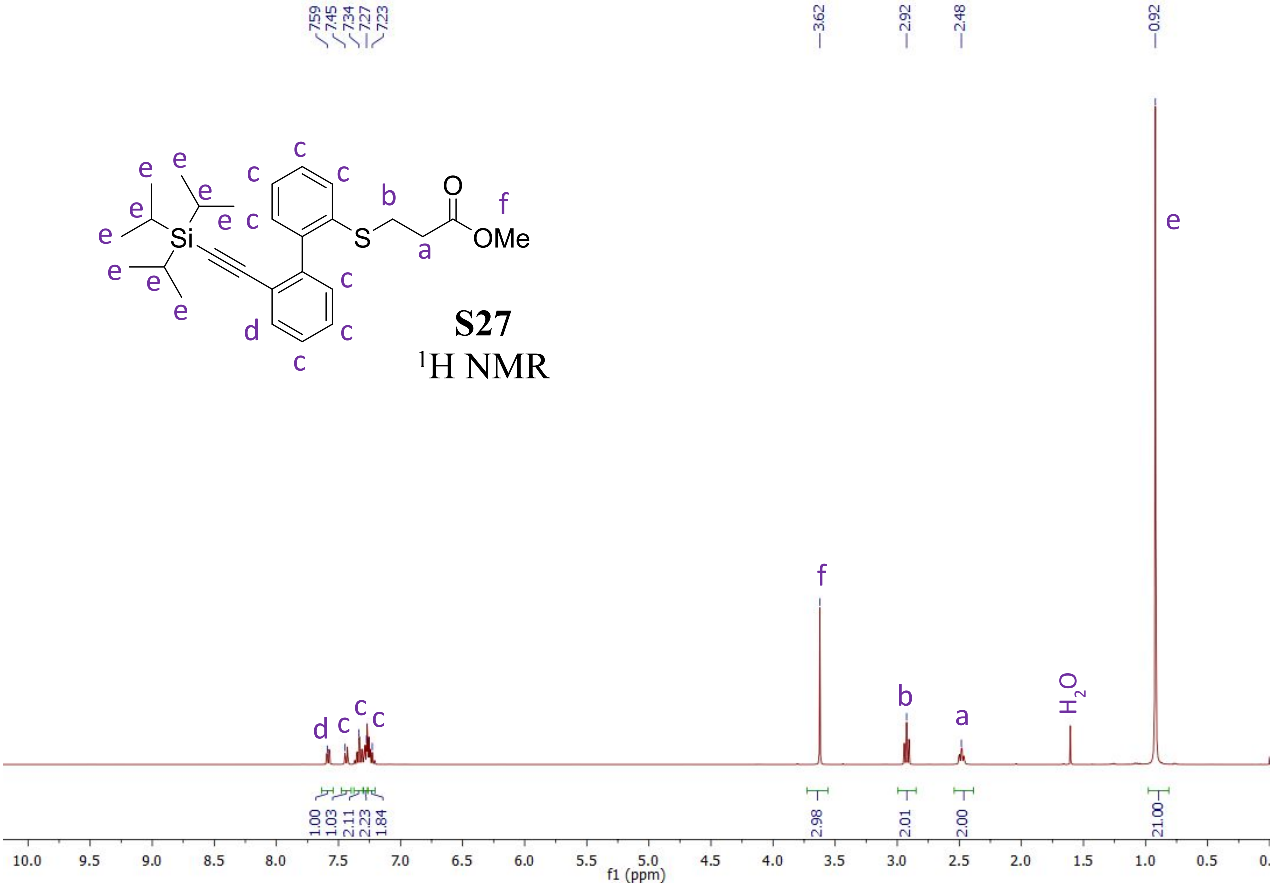
7.59
7.45
7.34
7.27
7.23
3.62
2.92
2.48
0.92
S27
^{1}H NMR
f
b
a
H_2O
e
1.00
1.03
2.11
2.23
1.84
2.98
2.01
2.00
21.00
f1 (ppm)

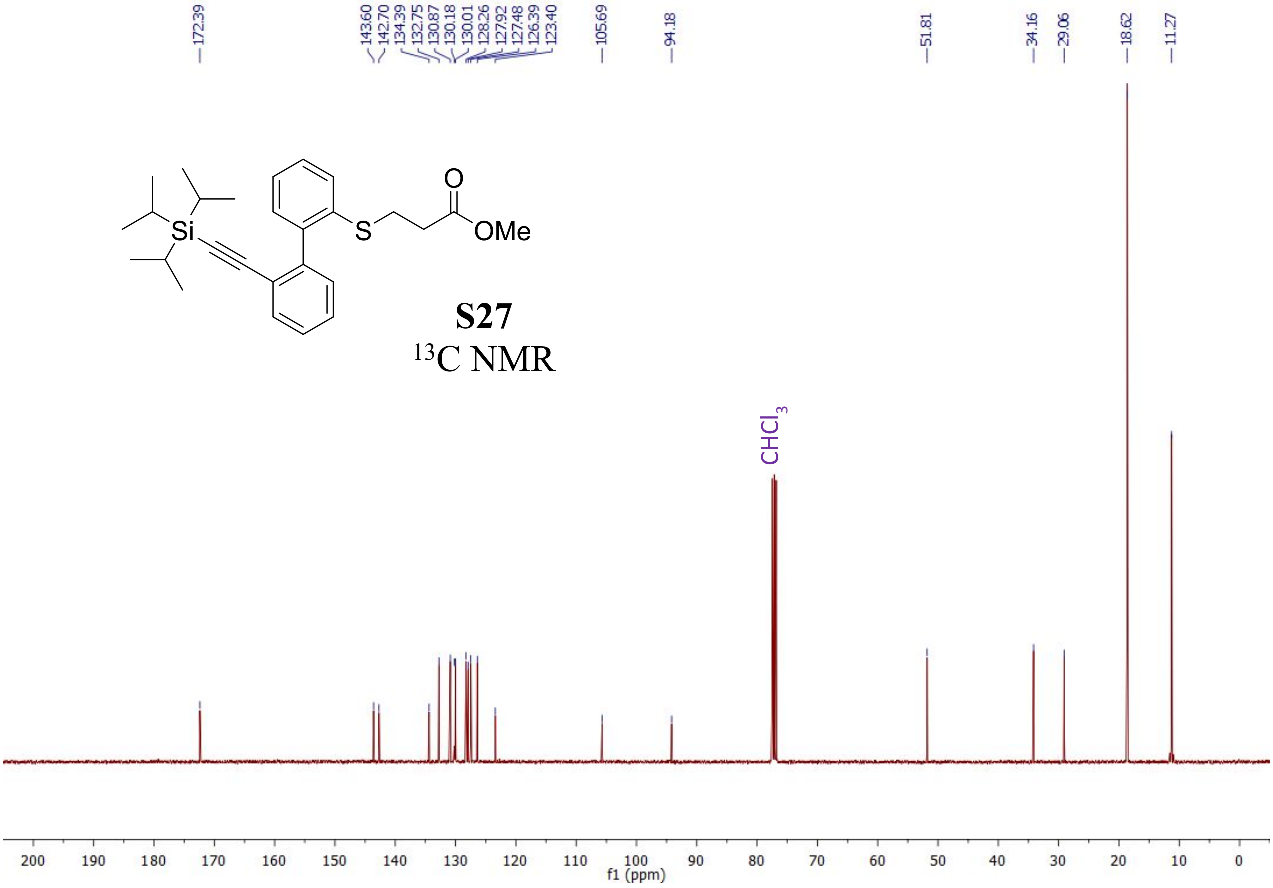
172.39
143.60
142.70
134.39
132.75
130.87
130.18
130.01
128.26
127.92
127.48
126.39
123.40
105.69
94.18
51.81
34.16
29.06
18.62
11.27
S27
^{13}C NMR
$CHCl_3$
f1 (ppm)

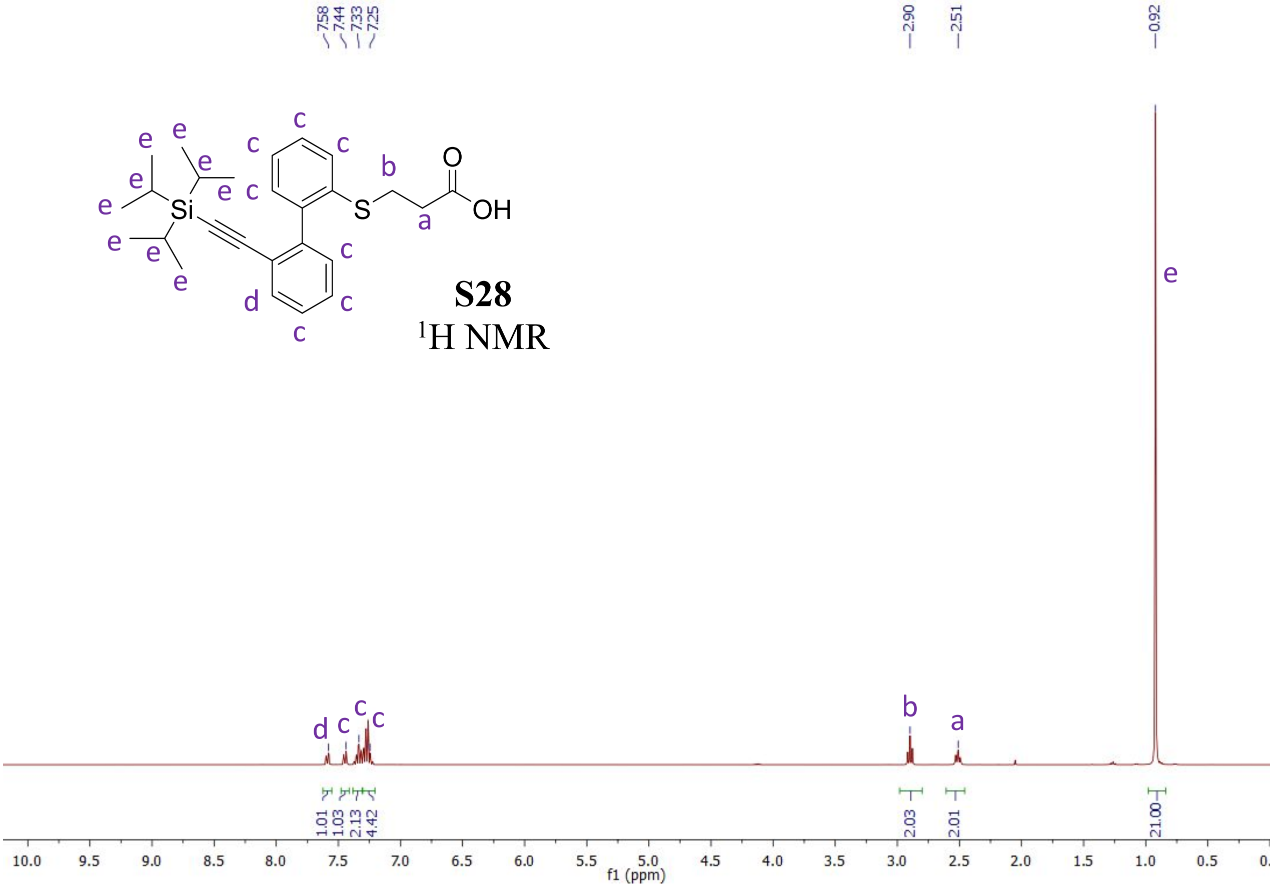
7.58
7.44
7.33
7.25
2.90
2.51
0.92
S28
^{1}H NMR
1.01
1.03
2.13
4.42
2.03
2.01
21.00
f1 (ppm)

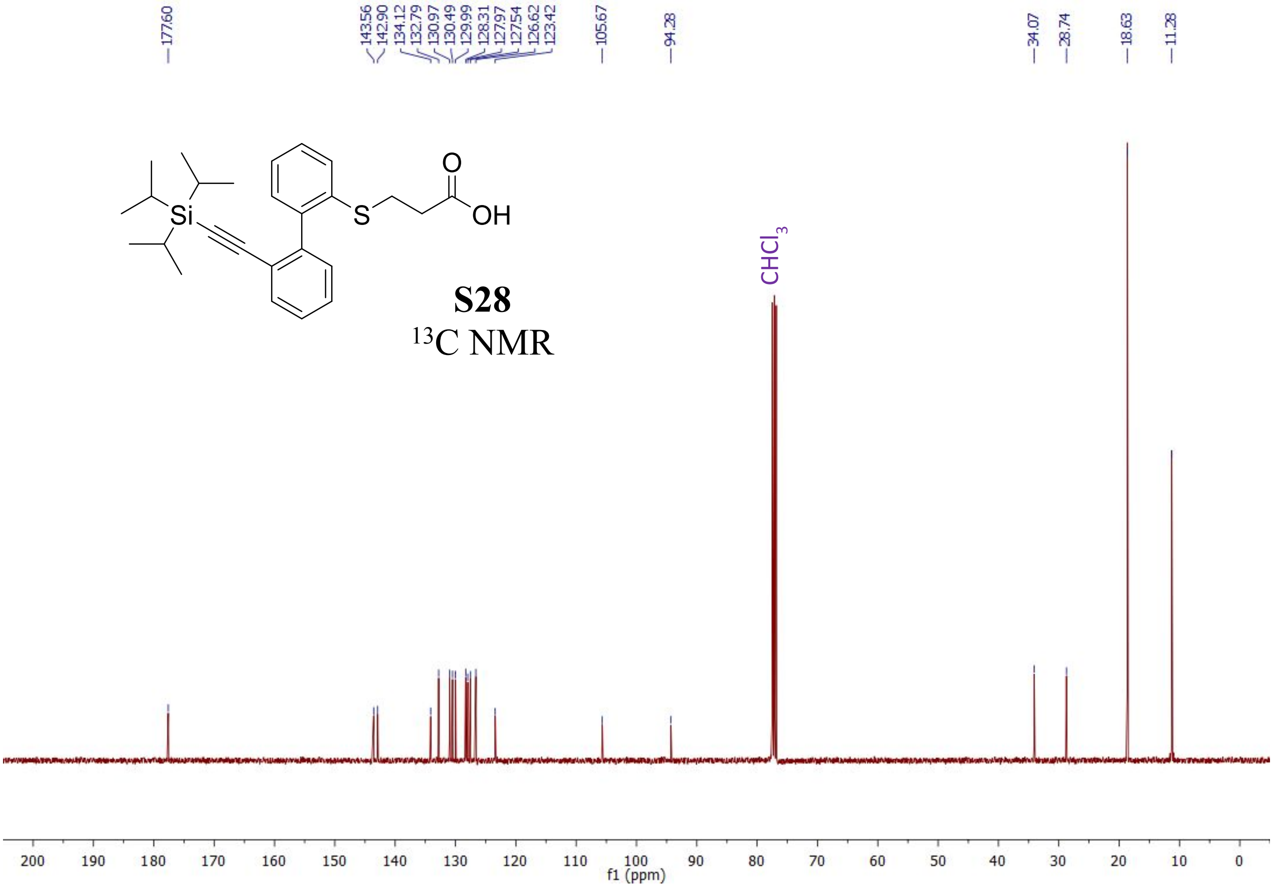
177.60
143.56
142.90
134.12
132.79
130.97
130.49
129.99
128.31
127.97
127.54
126.62
123.42
105.67
94.28
34.07
28.74
18.63
11.28
S28
^{13}C NMR
$CHCl_3$
f1 (ppm)

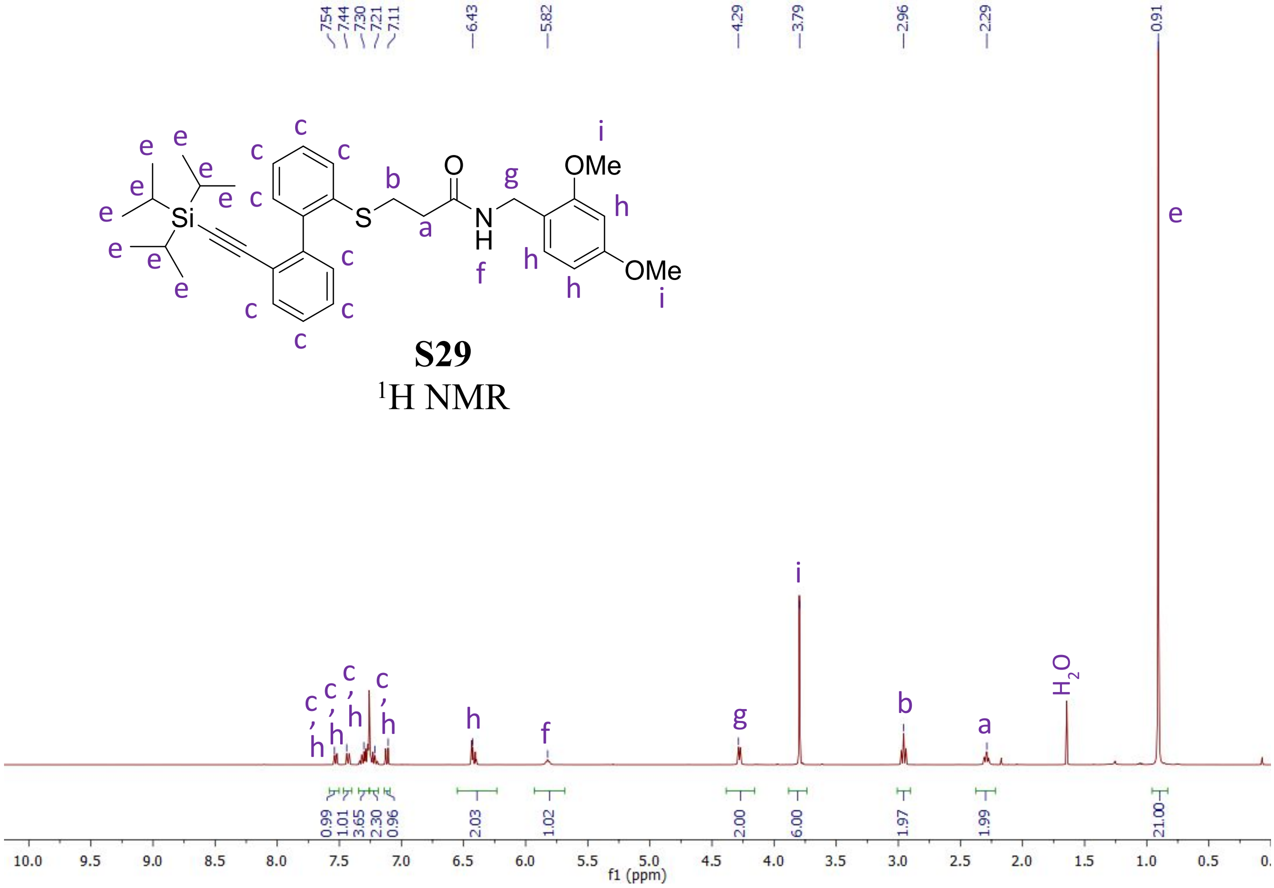
7.54
7.44
7.30
7.21
7.11
6.43
5.82
4.29
3.79
2.96
2.29
0.91
S29
1H NMR
0.99
1.01
3.65
2.30
0.96
2.03
1.02
2.00
6.00
1.97
1.99
21.00
f1 (ppm)

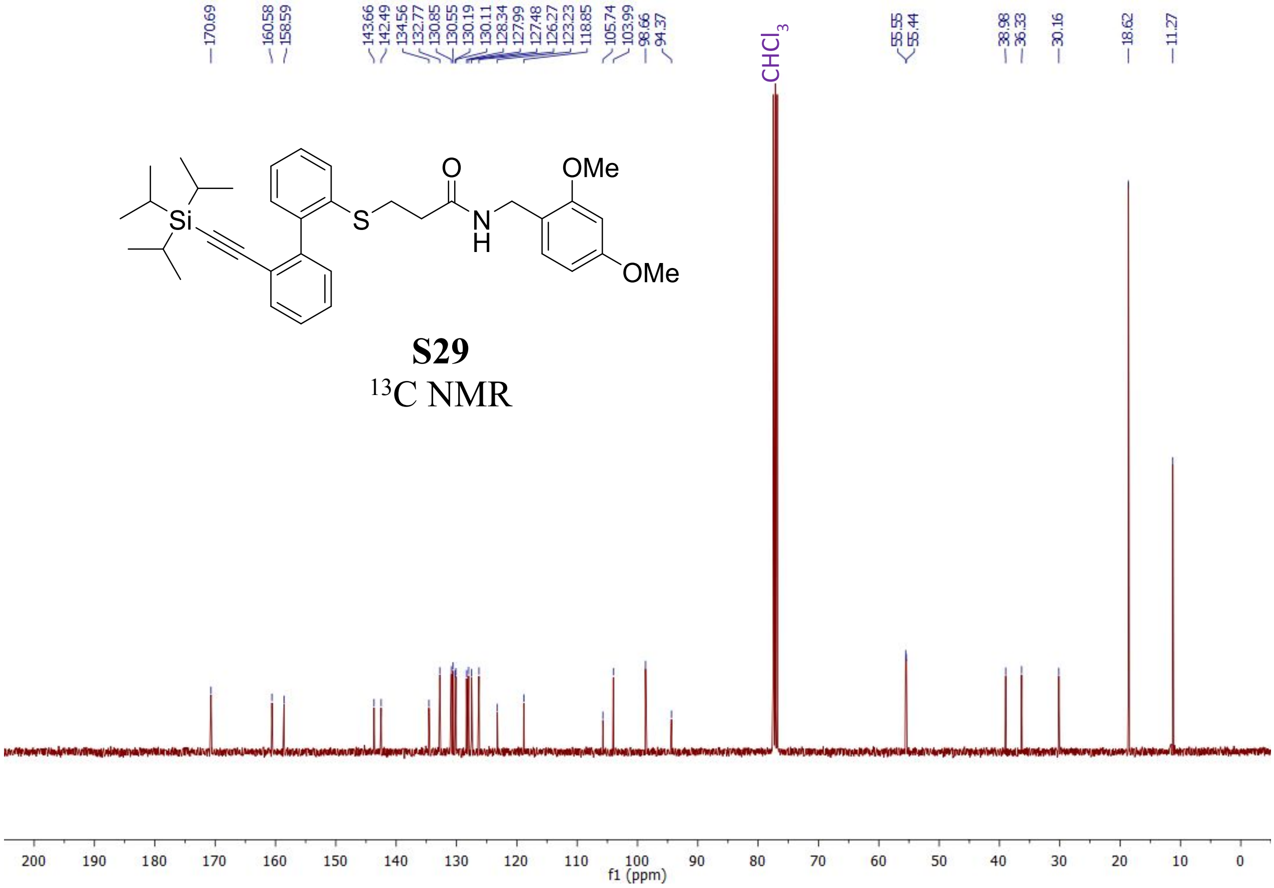
170.69
160.58
158.59
143.66
142.49
134.56
132.77
130.85
130.55
130.19
130.11
128.34
127.99
127.48
126.27
123.23
118.85
105.74
103.99
98.66
94.37
CHCl3
55.55
55.44
38.98
36.33
30.16
18.62
11.27
S29
13C NMR
f1 (ppm)

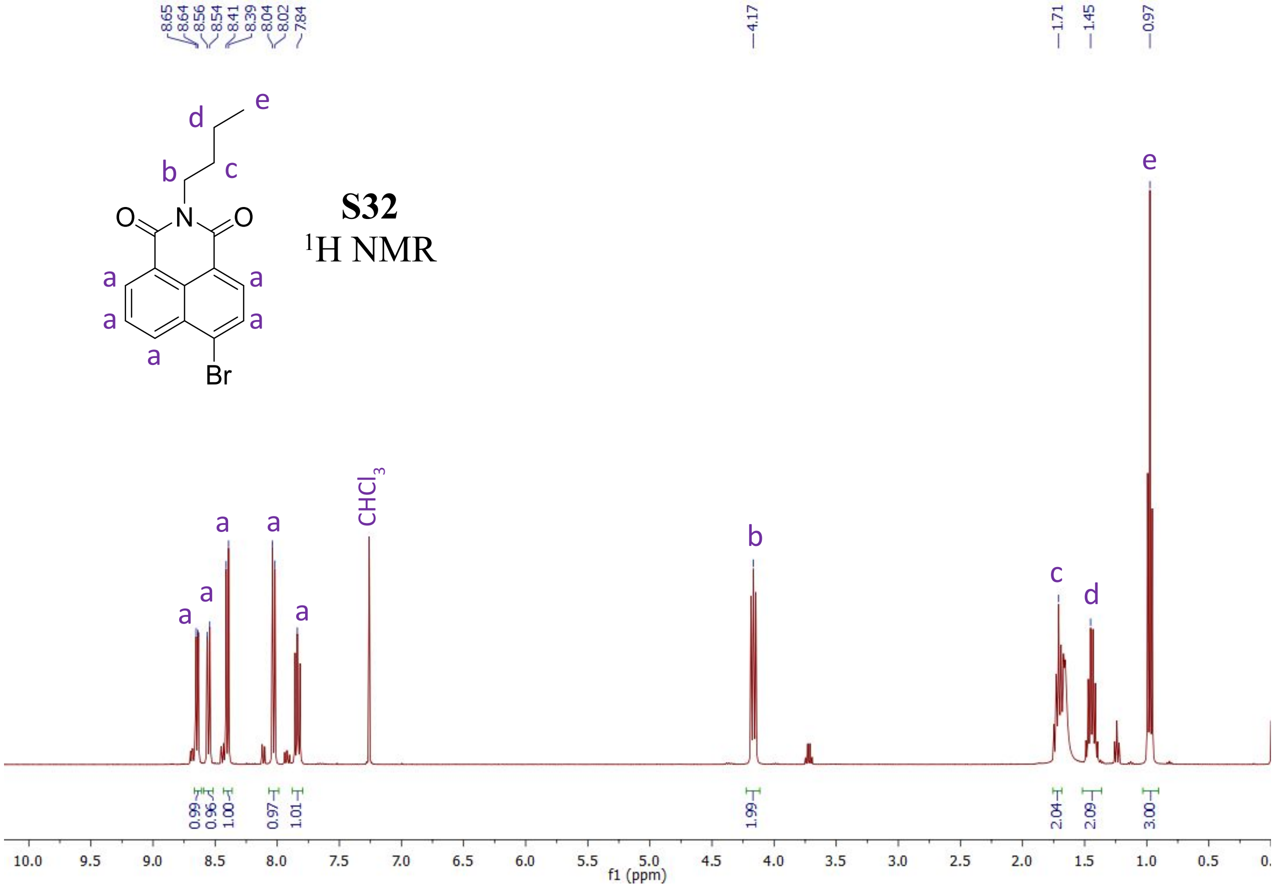
S32
1H NMR
CHCl3
a
b
c
d
e
8.65
8.64
8.56
8.54
8.41
8.39
8.04
8.02
7.84
4.17
1.71
1.45
0.97
0.99
0.96
1.00
0.97
1.01
1.99
2.04
2.09
3.00
f1 (ppm)

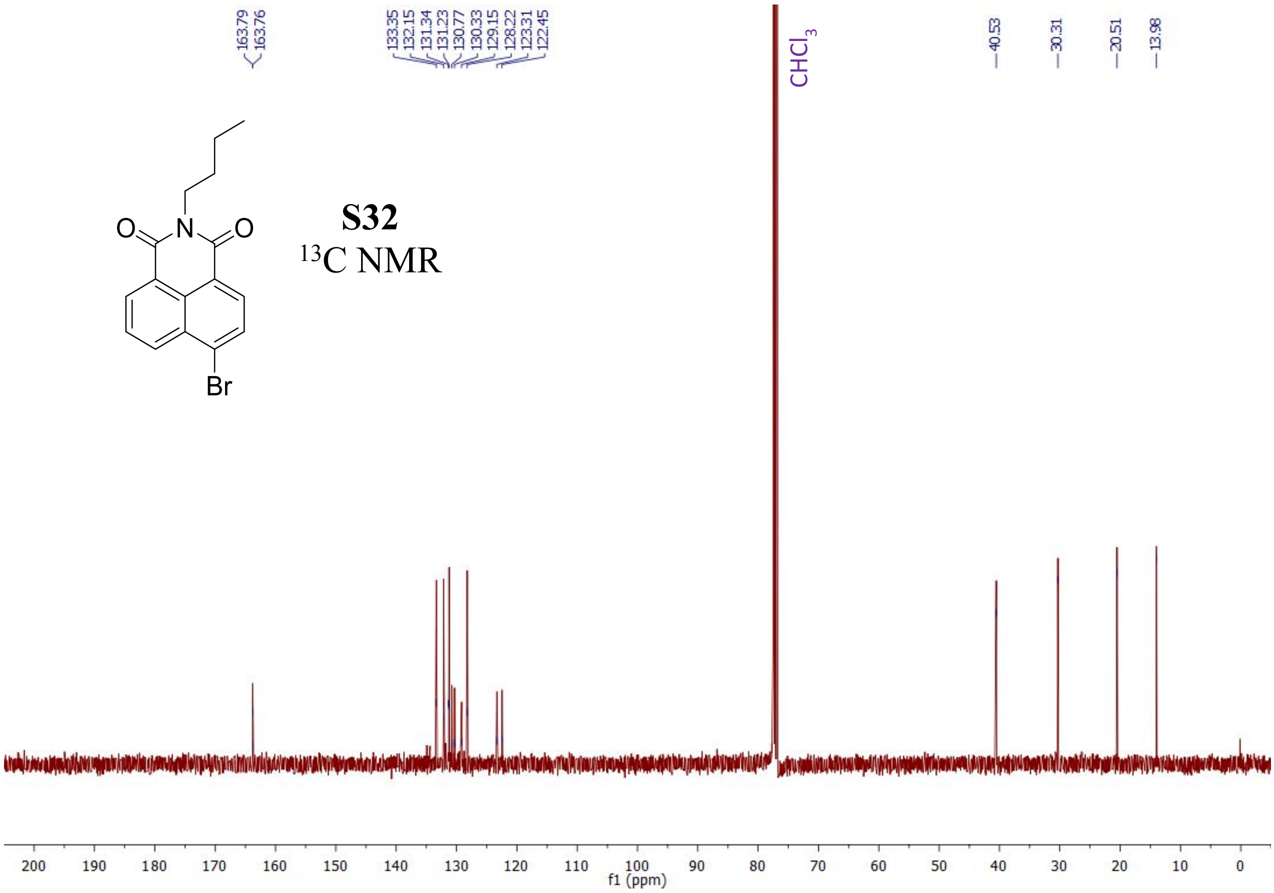
S32
13C NMR
CHCl3
163.79
163.76
133.35
132.15
131.34
131.23
130.77
130.33
129.15
128.22
123.31
122.45
40.53
30.31
20.51
13.98
f1 (ppm)

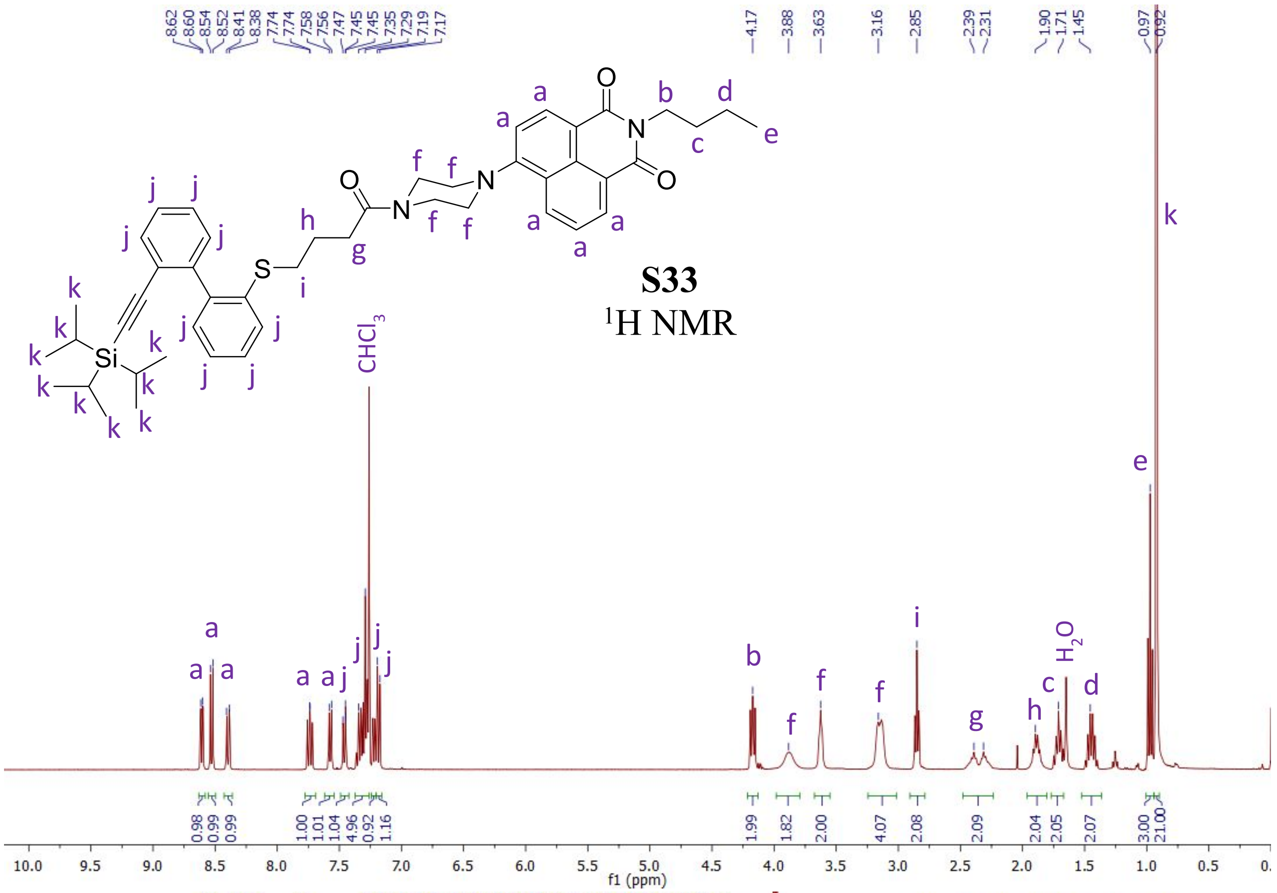

8.62 8.60 8.54 8.52 8.41 8.38 7.74 7.74 7.58 7.56 7.47 7.45 7.45 7.35 7.29 7.19 7.17
4.17 3.88 3.63 3.16 2.85 2.39 2.31 1.90 1.71 1.45 0.97 0.92
S33
1H NMR
CHCl3
H2O
0.98 0.99 0.99 1.00 1.01 1.04 4.96 0.92 1.16 1.99 1.82 2.00 4.07 2.08 2.09 2.04 2.05 2.07 3.00 21.00
f1 (ppm)

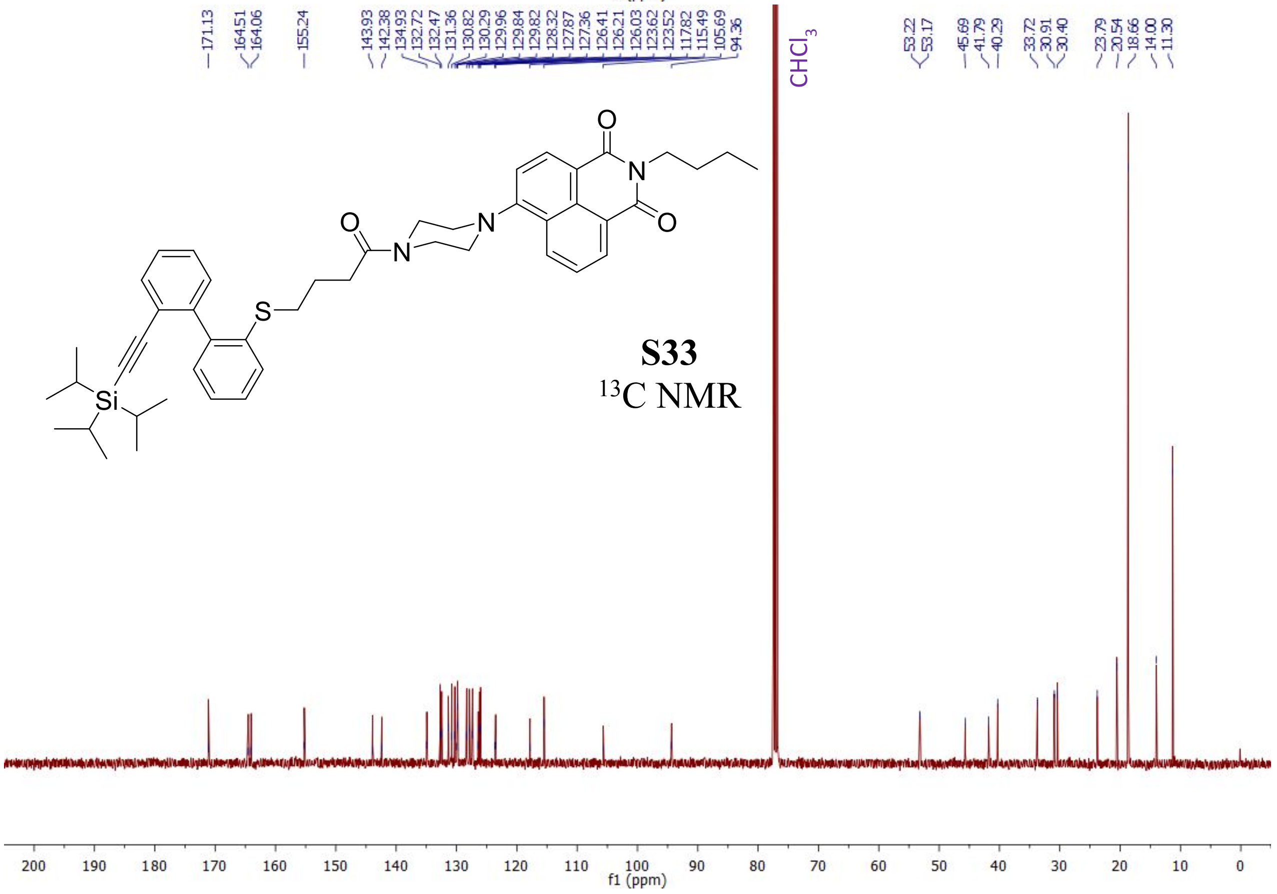

171.13 164.51 164.06 155.24 143.93 142.38 134.93 132.72 132.47 131.36 130.82 130.29 129.96 129.84 129.82 128.32 127.87 127.36 126.41 126.21 126.03 123.62 123.52 117.82 115.49 105.69 94.36
53.22 53.17 45.69 41.79 40.29 33.72 30.91 30.40 23.79 20.54 18.66 14.00 11.30
CHCl3
S33
13C NMR
f1 (ppm)

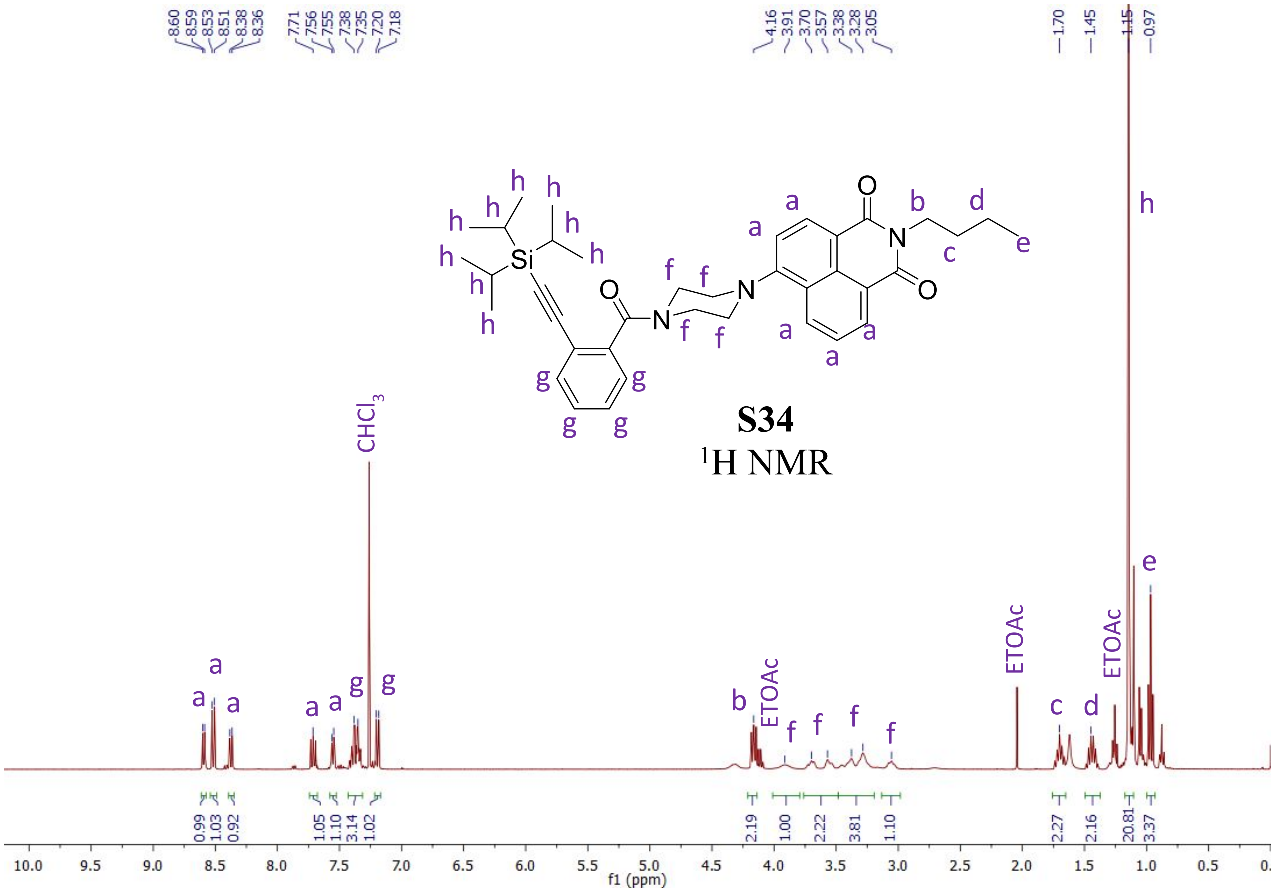
8.60 8.59 8.53 8.51 8.38 8.36
7.71 7.56 7.55 7.38 7.35 7.20 7.18
4.16 3.91 3.70 3.57 3.38 3.28 3.05
1.70
1.45
1.15
0.97
S34
1H NMR
CHCl3
ETOAc
0.99 1.03 0.92 1.05 1.10 3.14 1.02
2.19 1.00 2.22 3.81 1.10
2.27 2.16 20.81 3.37
f1 (ppm)

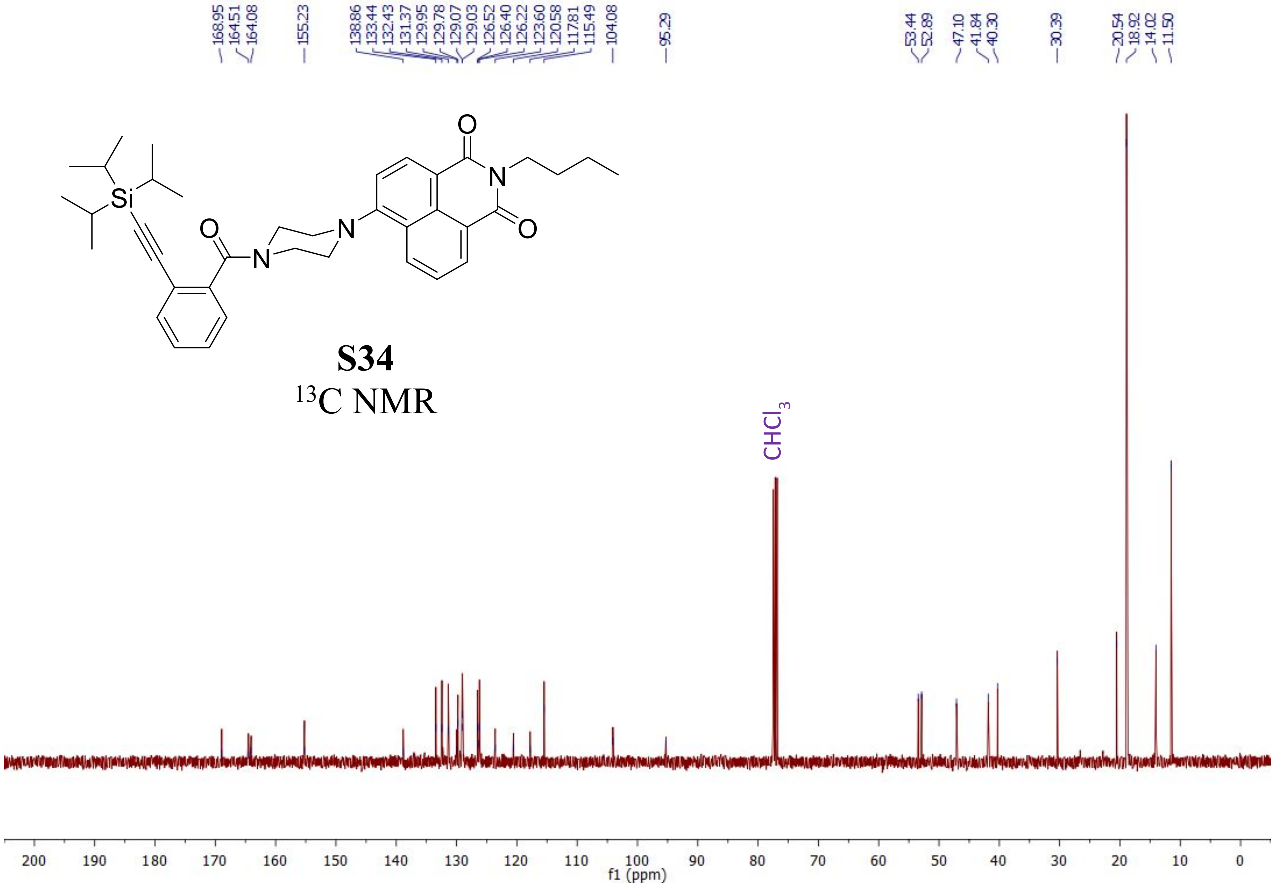
168.95 164.51 164.08
155.23
138.86 133.44 132.43 131.37 129.95 129.78 129.07 129.03 126.52 126.40 126.22 123.60 120.58 117.81 115.49
104.08
95.29
53.44 52.89
47.10 41.84 40.30
30.39
20.54 18.92 14.02 11.50
S34
13C NMR
CHCl3
f1 (ppm)

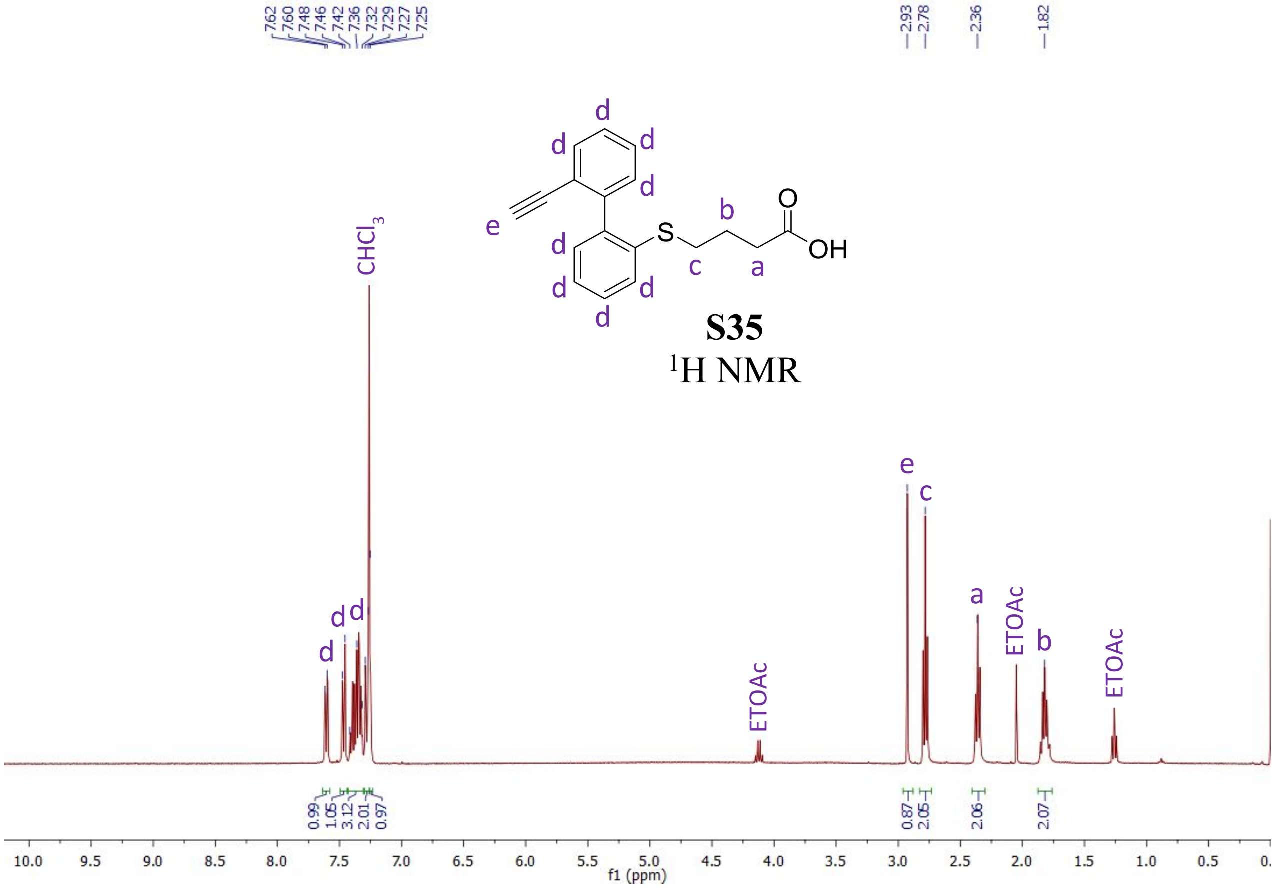

7.62
7.60
7.48
7.46
7.42
7.36
7.32
7.29
7.27
7.25
2.93
2.78
2.36
1.82
S35
1H NMR
CHCl3
ETOAc
0.99
1.05
3.12
2.01
0.97
0.87
2.05
2.06
2.07
f1 (ppm)

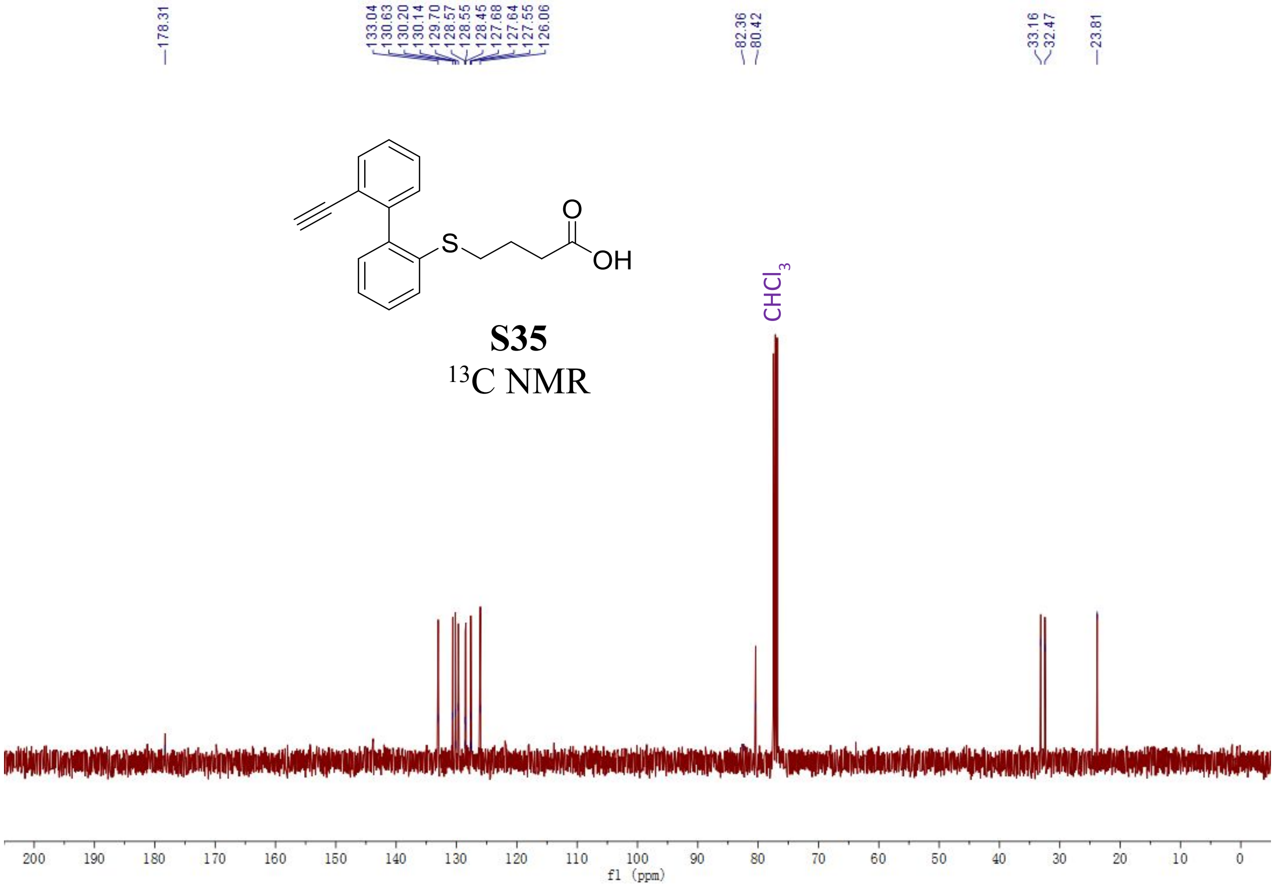

178.31
133.04
130.63
130.20
130.14
129.70
128.57
128.55
128.45
127.68
127.64
127.55
126.06
82.36
80.42
33.16
32.47
23.81
S35
13C NMR
CHCl3
f1 (ppm)

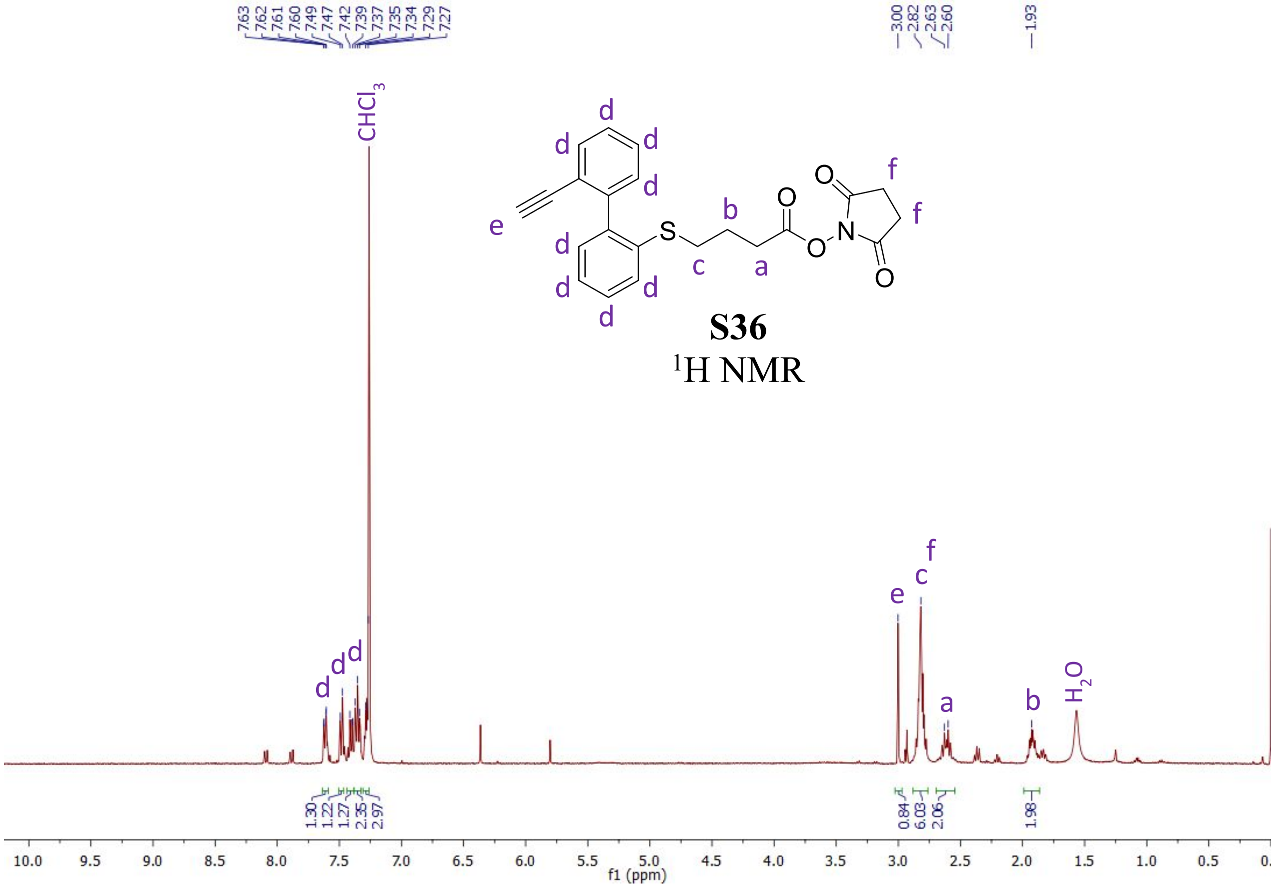
7.63
7.62
7.61
7.60
7.49
7.47
7.42
7.39
7.37
7.35
7.34
7.29
7.27
3.00
2.82
2.63
2.60
1.93
CHCl3
S36
1H NMR
e
c
f
a
b
H2O
1.30
1.22
1.27
2.35
2.97
0.84
6.03
2.06
1.98
f1 (ppm)

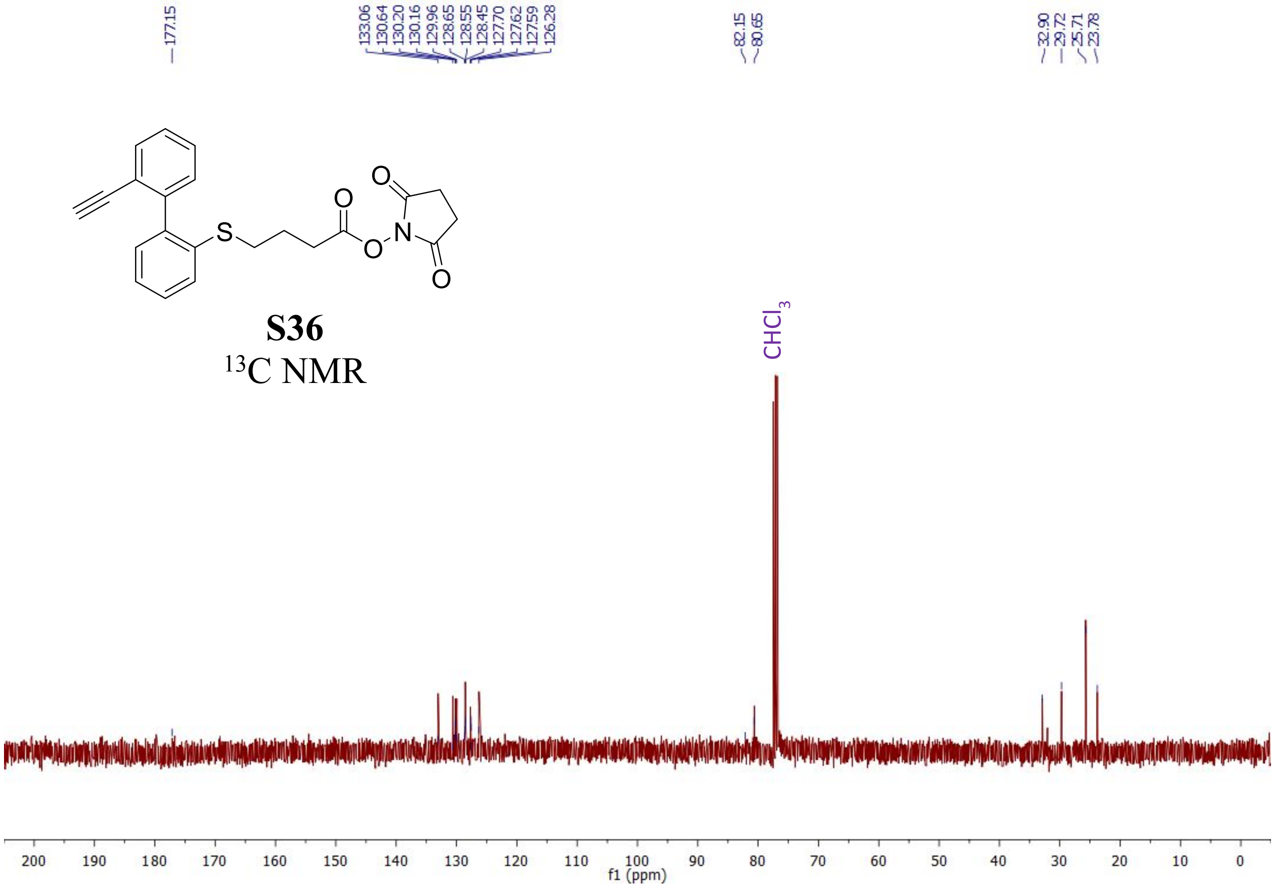
177.15
133.06
130.64
130.20
130.16
129.96
128.65
128.55
128.45
127.70
127.62
127.59
126.28
82.15
80.65
32.90
29.72
25.71
23.78
S36
13C NMR
CHCl3
f1 (ppm)

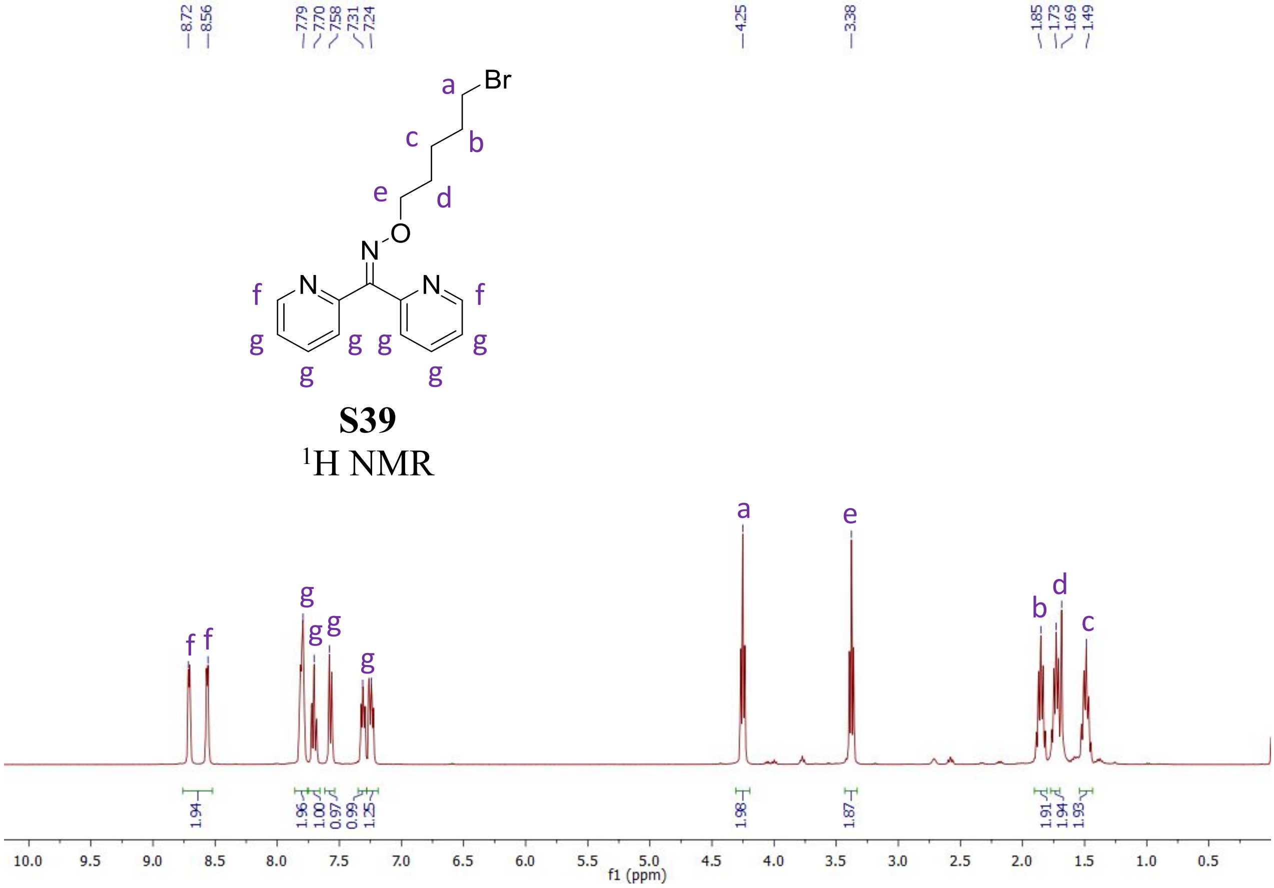
8.72
8.56
7.79
7.70
7.58
7.31
7.24
4.25
3.38
1.85
1.73
1.69
1.49
a
b
c
d
e
f
g
Br
O
N
S39
^{1}H NMR
1.94
1.96
1.00
0.97
0.99
1.25
1.98
1.87
1.91
1.94
1.93
f1 (ppm)

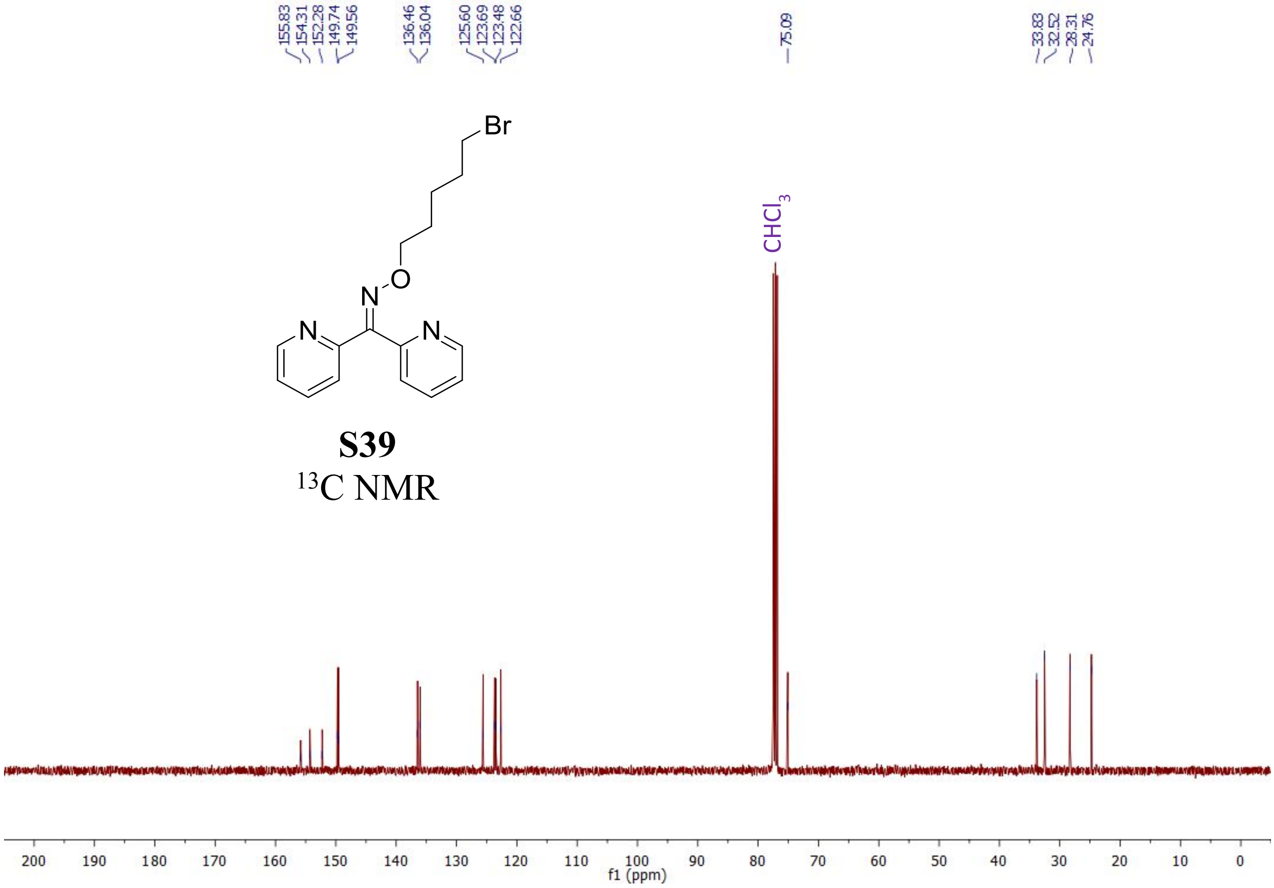
155.83
154.31
152.28
149.74
149.56
136.46
136.04
125.60
123.69
123.48
122.66
75.09
33.83
32.52
28.31
24.76
Br
O
N
$CHCl_3$
S39
^{13}C NMR
f1 (ppm)

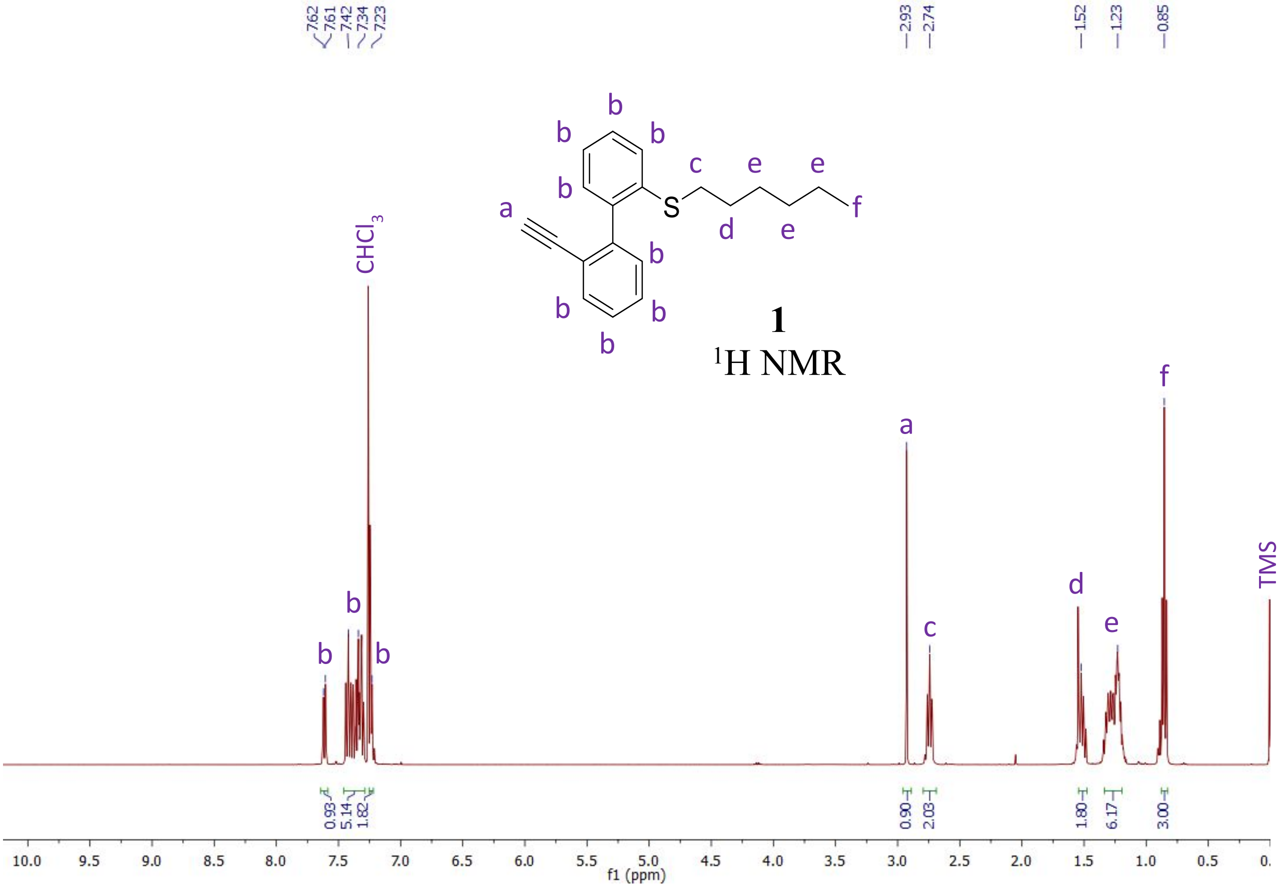
7.62
7.61
7.42
7.34
7.23
2.93
2.74
1.52
1.23
0.85
a
b
c
d
e
f
S
1
¹H NMR
CHCl3
TMS
0.93
5.14
1.82
0.90
2.03
1.80
6.17
3.00
f1 (ppm)

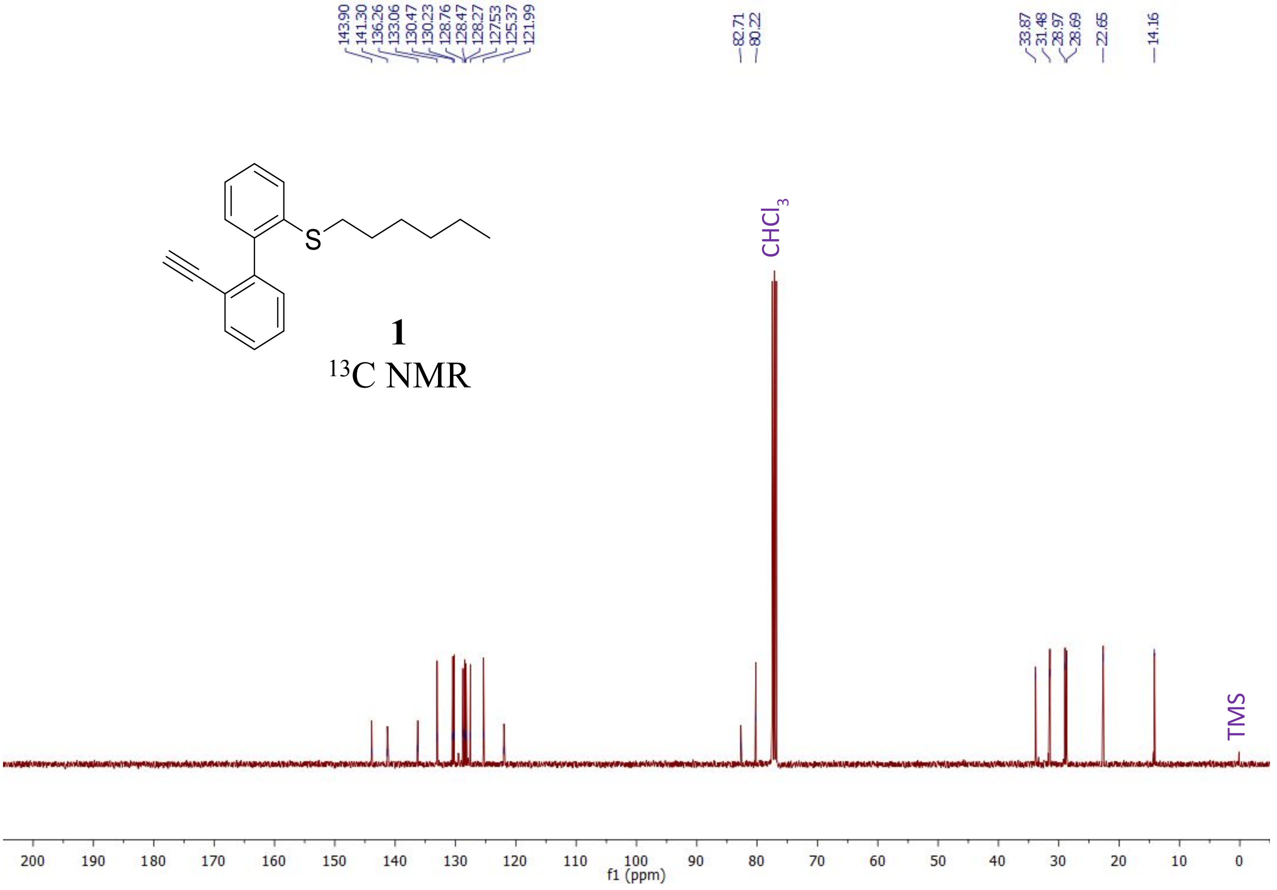
143.90
141.30
136.26
133.06
130.47
130.23
128.76
128.47
128.27
127.53
125.37
121.99
82.71
80.22
33.87
31.48
28.97
28.69
22.65
14.16
S
1
¹³C NMR
CHCl3
TMS
f1 (ppm)

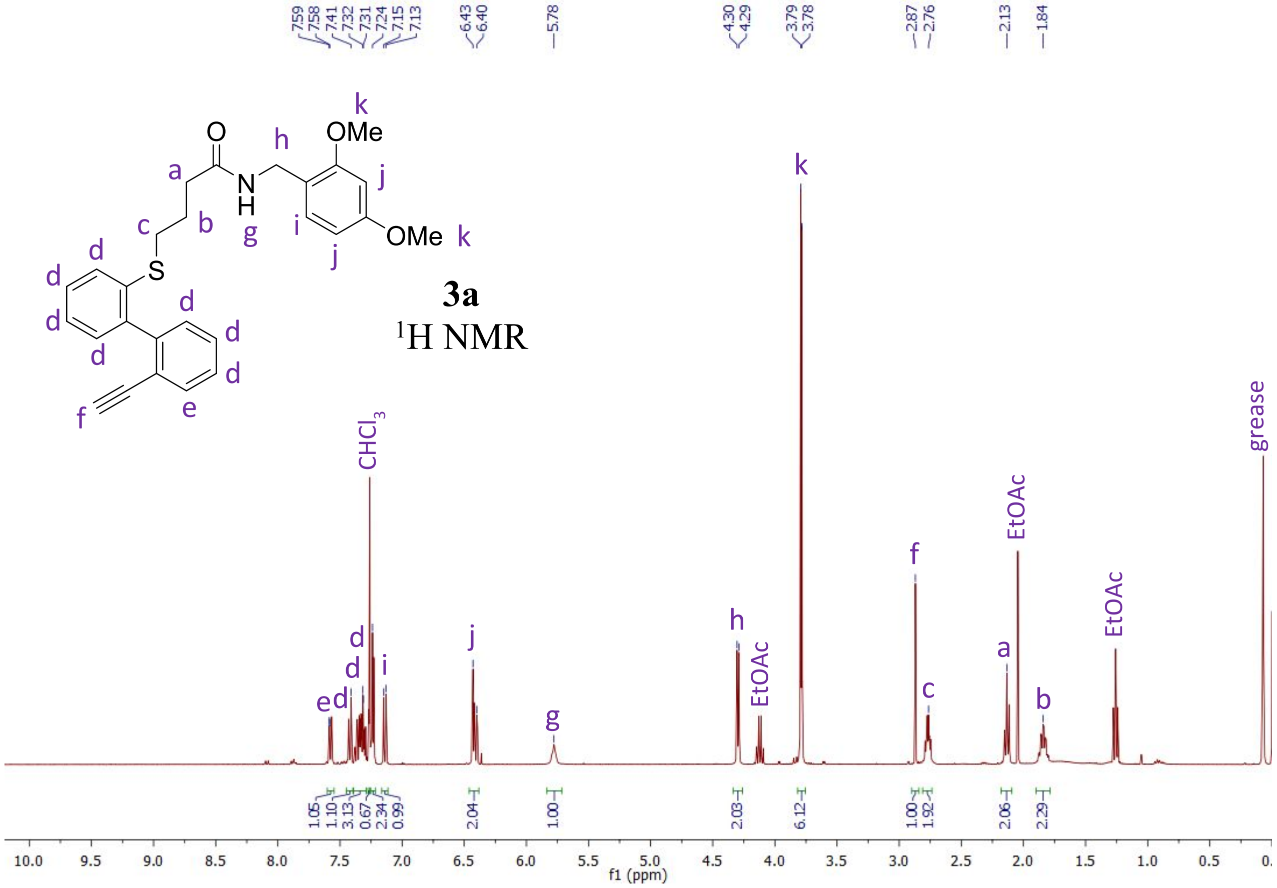
7.59
7.58
7.41
7.32
7.31
7.24
7.15
7.13
6.43
6.40
5.78
4.30
4.29
3.79
3.78
2.87
2.76
2.13
1.84
3a
1H NMR
OMe
OMe
O
N
H
S
a b c d e f g h i j k
CHCl3
EtOAc
grease
1.05
1.10
3.13
0.67
2.34
0.99
2.04
1.00
2.03
6.12
1.00
1.92
2.06
2.29
f1 (ppm)

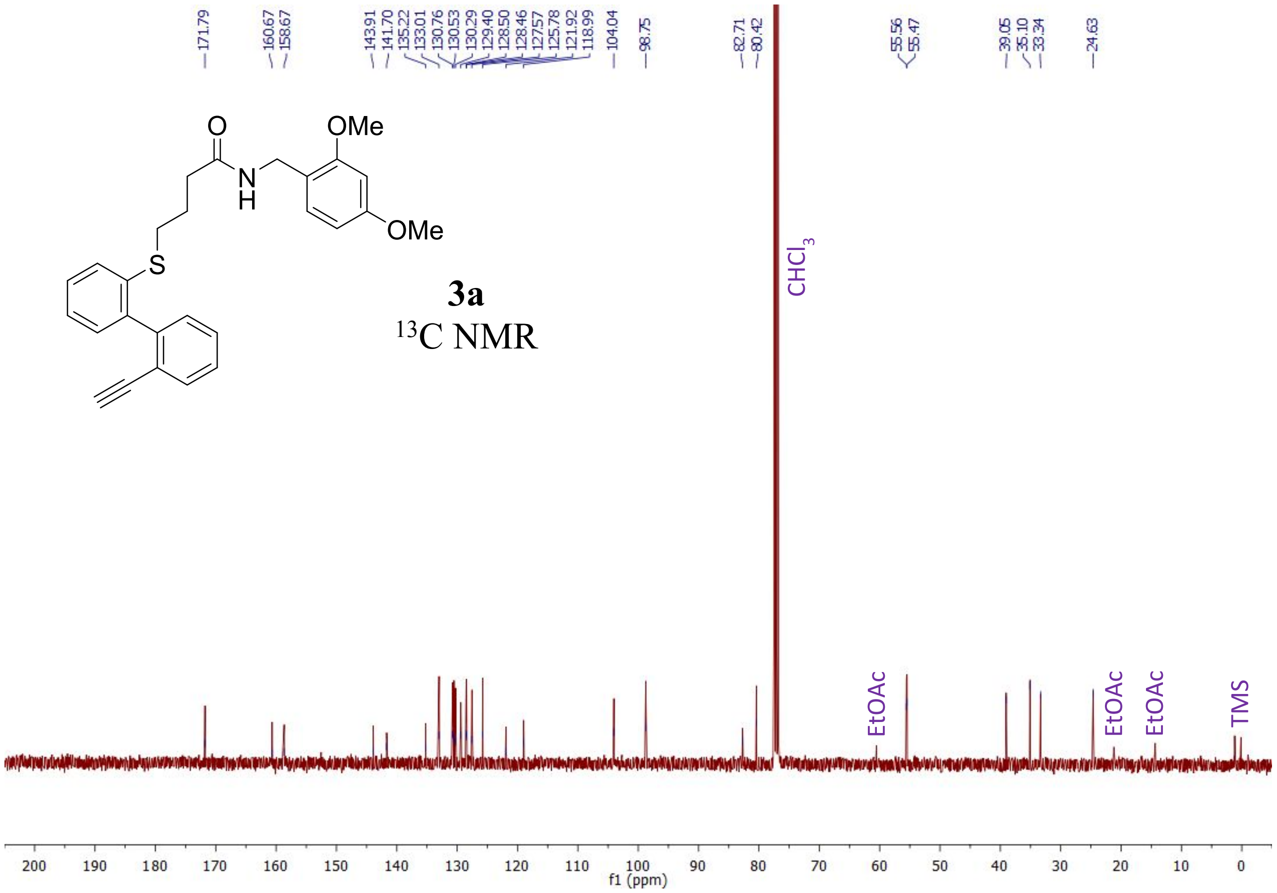
171.79
160.67
158.67
143.91
141.70
135.22
133.01
130.76
130.53
130.29
129.40
128.50
128.46
127.57
125.78
121.92
118.99
104.04
98.75
82.71
80.42
55.56
55.47
39.05
35.10
33.34
24.63
3a
13C NMR
OMe
OMe
O
N
H
S
CHCl3
EtOAc
TMS
f1 (ppm)

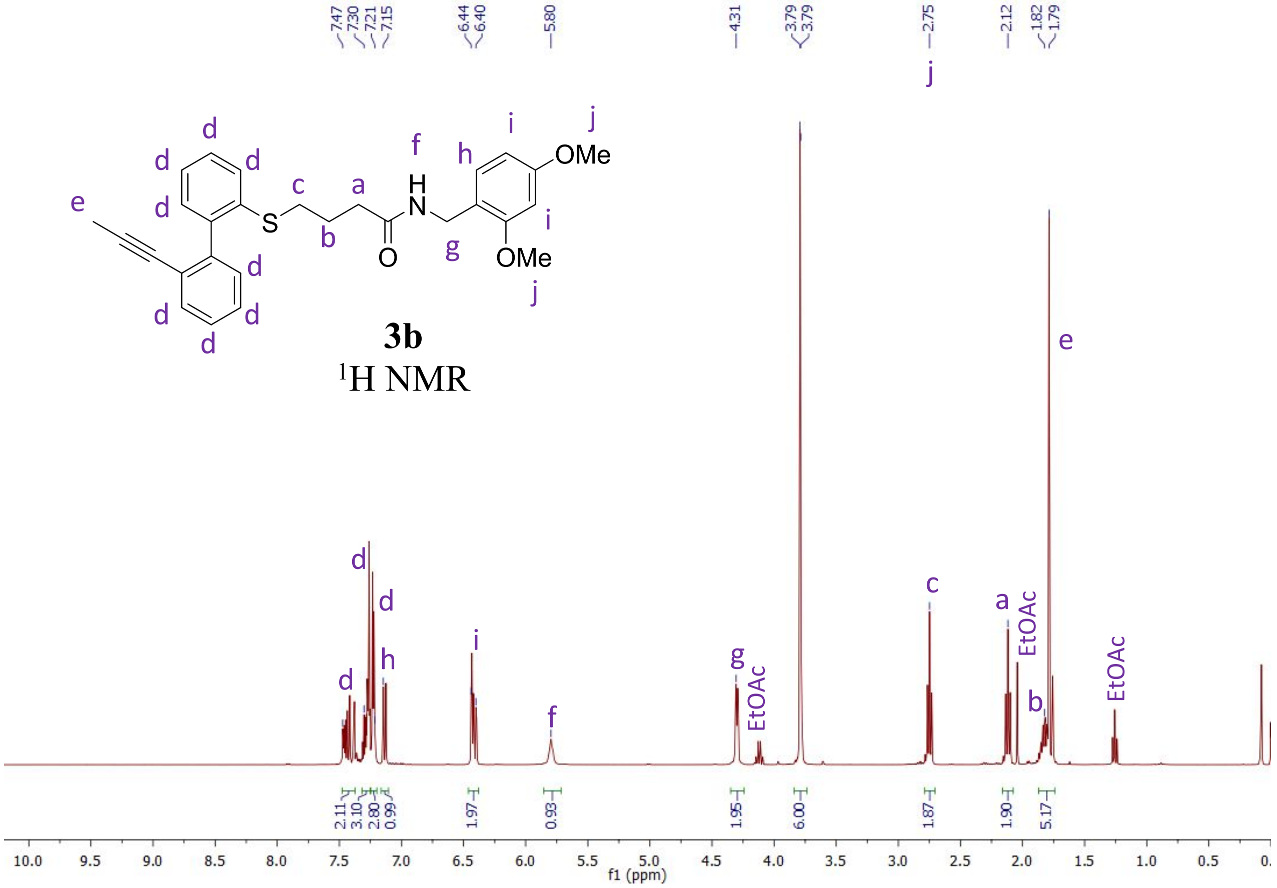

3b

^{1}H NMR

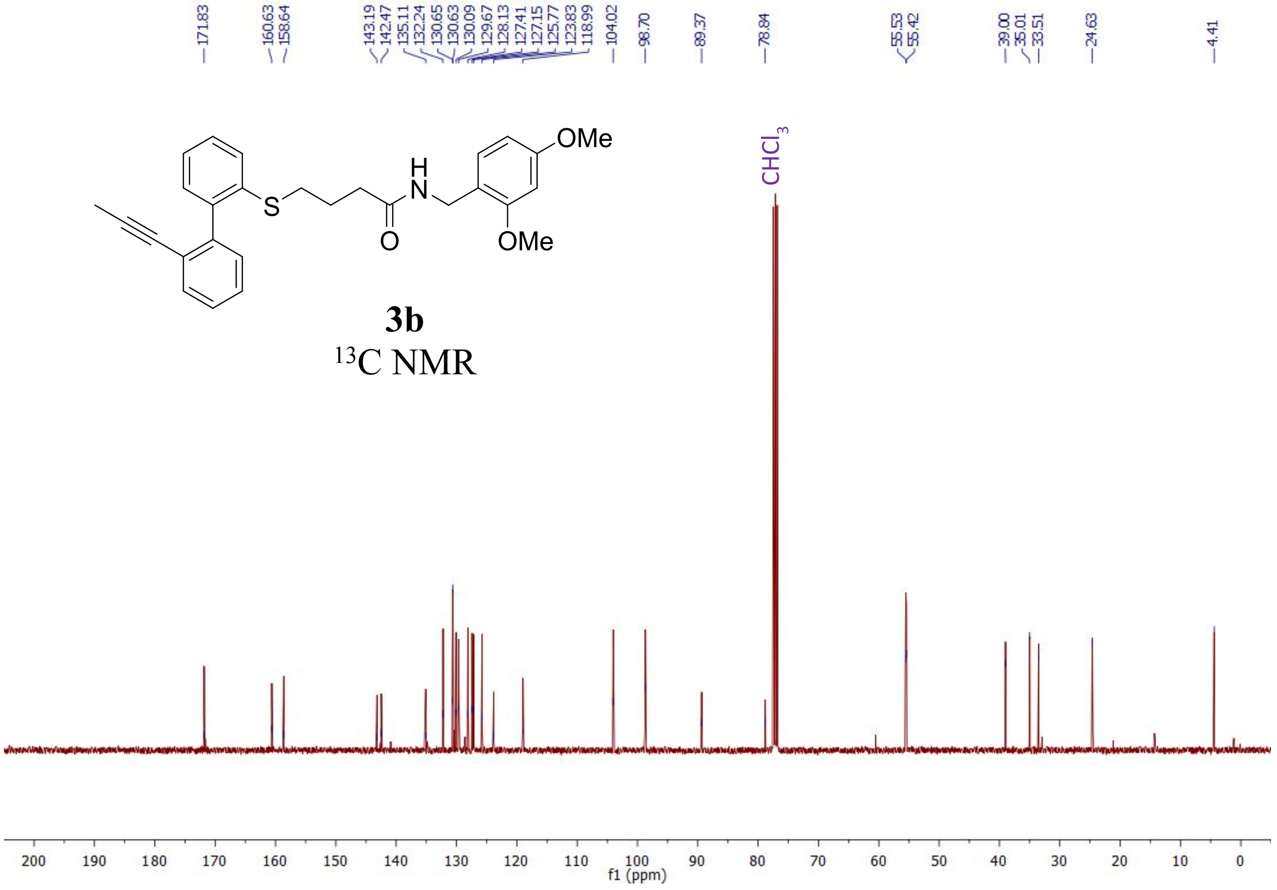

3b

^{13}C NMR

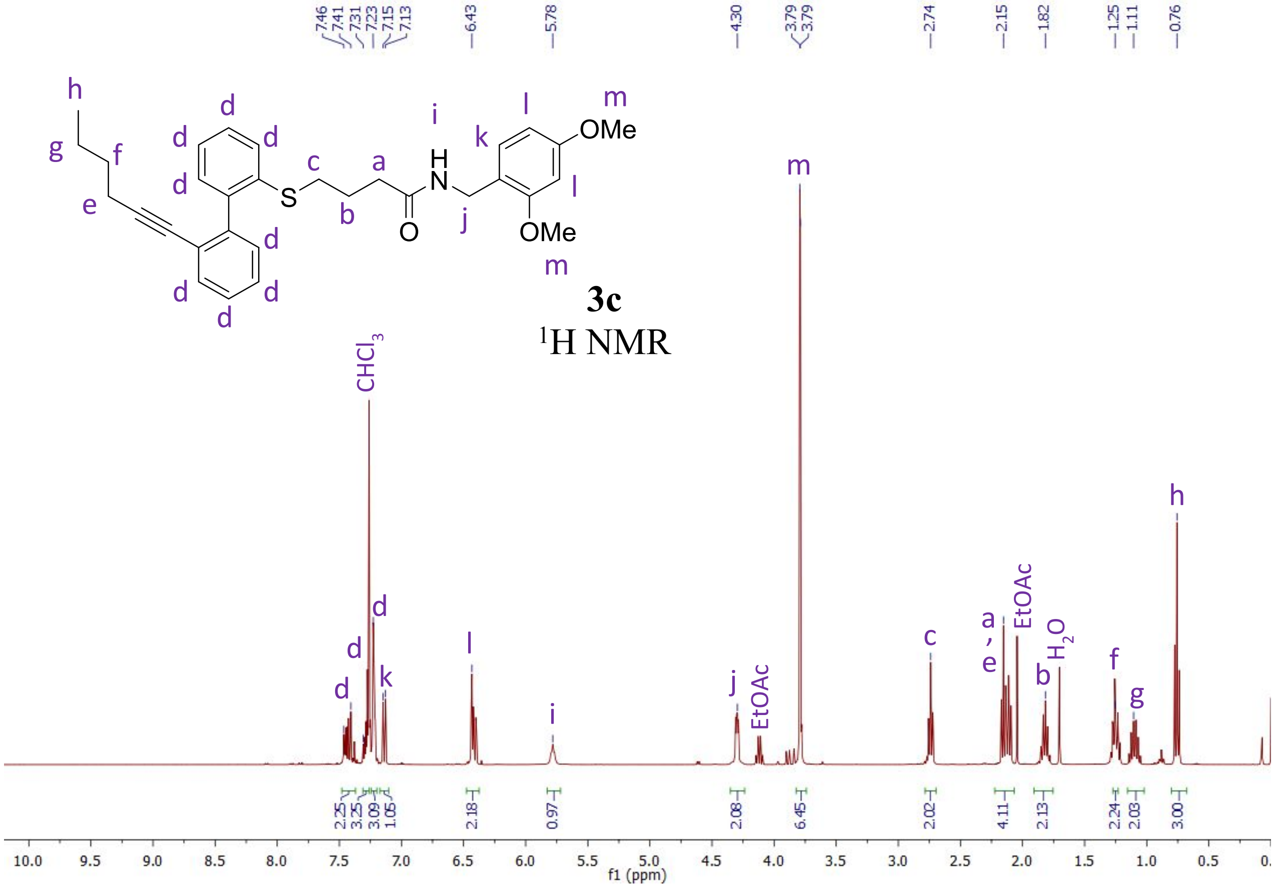
7.46
7.41
7.31
7.23
7.15
7.13
6.43
5.78
4.30
3.79
3.79
2.74
2.15
1.82
1.25
1.11
0.76
OMe
OMe
3c
1H NMR
CHCl3
EtOAc
H2O
2.25
3.25
3.09
1.05
2.18
0.97
2.08
6.45
2.02
4.11
2.13
2.24
2.03
3.00
f1 (ppm)

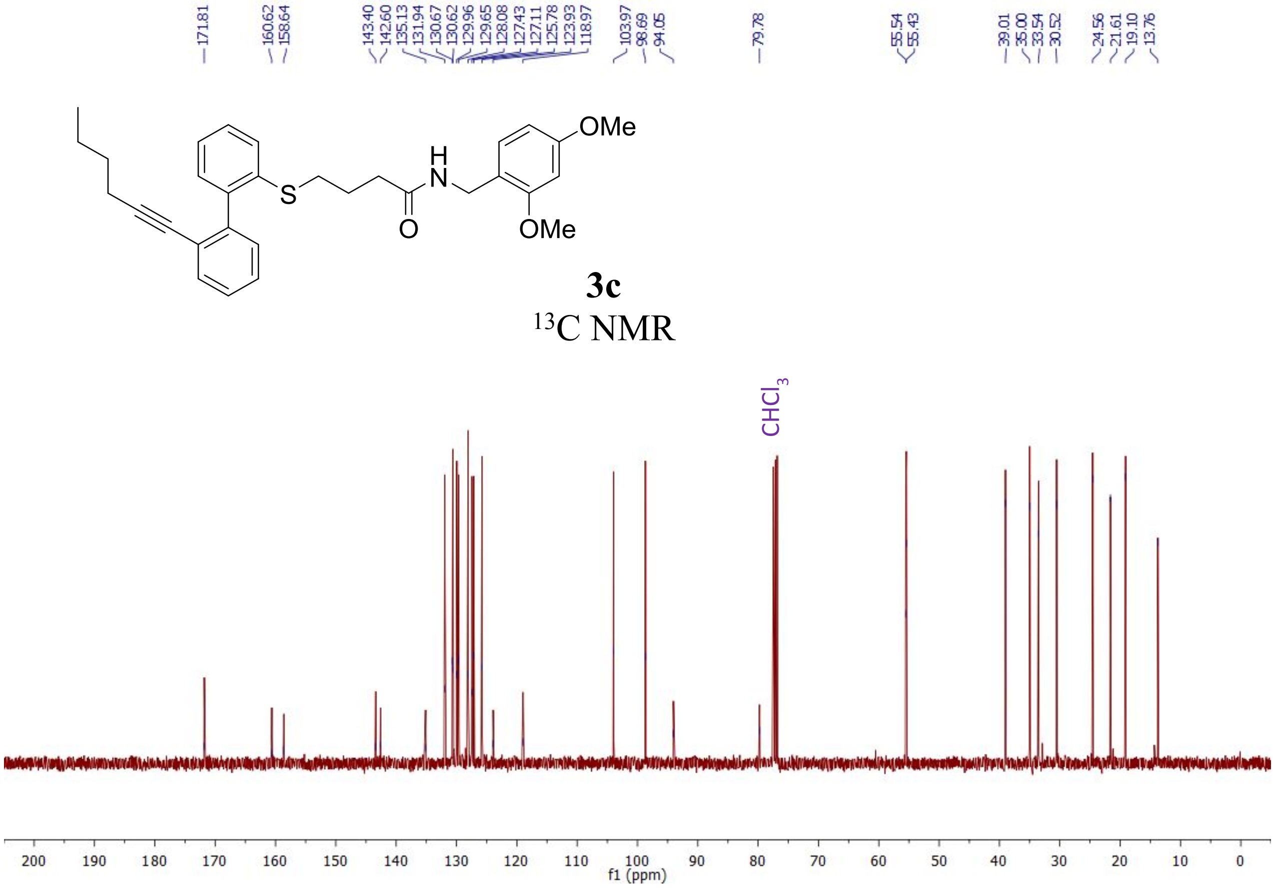
171.81
160.62
158.64
143.40
142.60
135.13
131.94
130.67
130.62
129.96
129.65
128.08
127.43
127.11
125.78
123.93
118.97
103.97
98.69
94.05
79.78
55.54
55.43
39.01
35.00
33.54
30.52
24.56
21.61
19.10
13.76
OMe
OMe
3c
13C NMR
CHCl3
f1 (ppm)

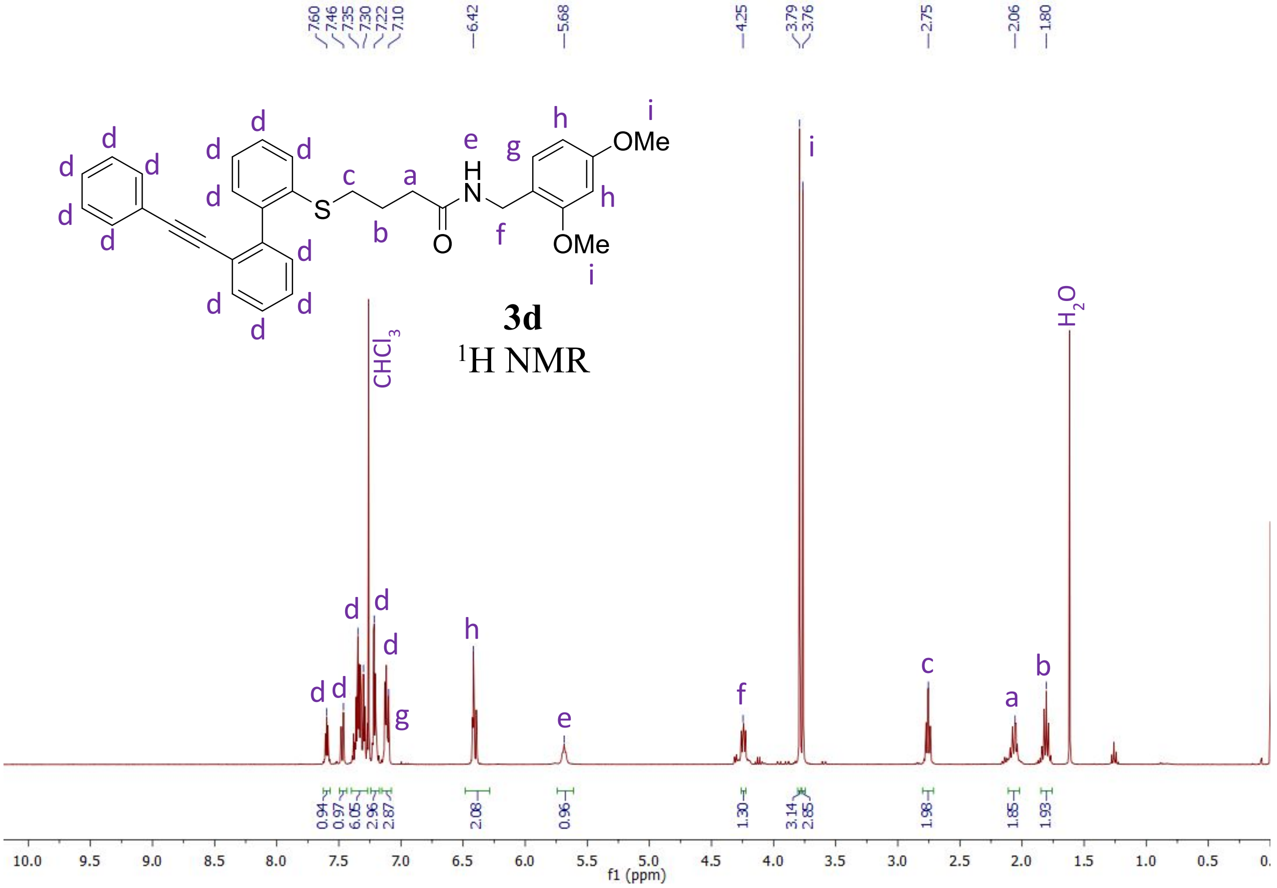

3d
^{1}H NMR
CHCl3
H2O
f1 (ppm)

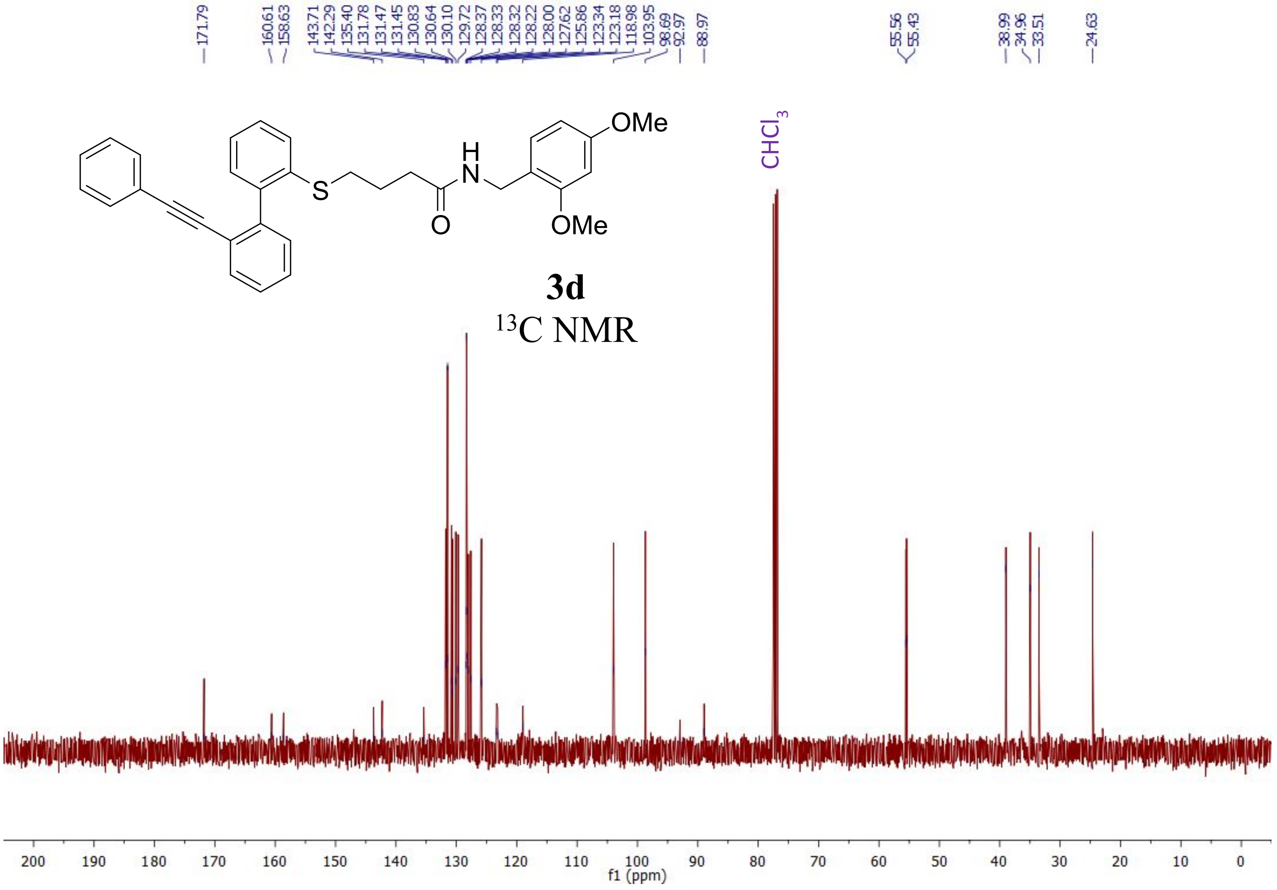

3d
^{13}C NMR
CHCl3
f1 (ppm)

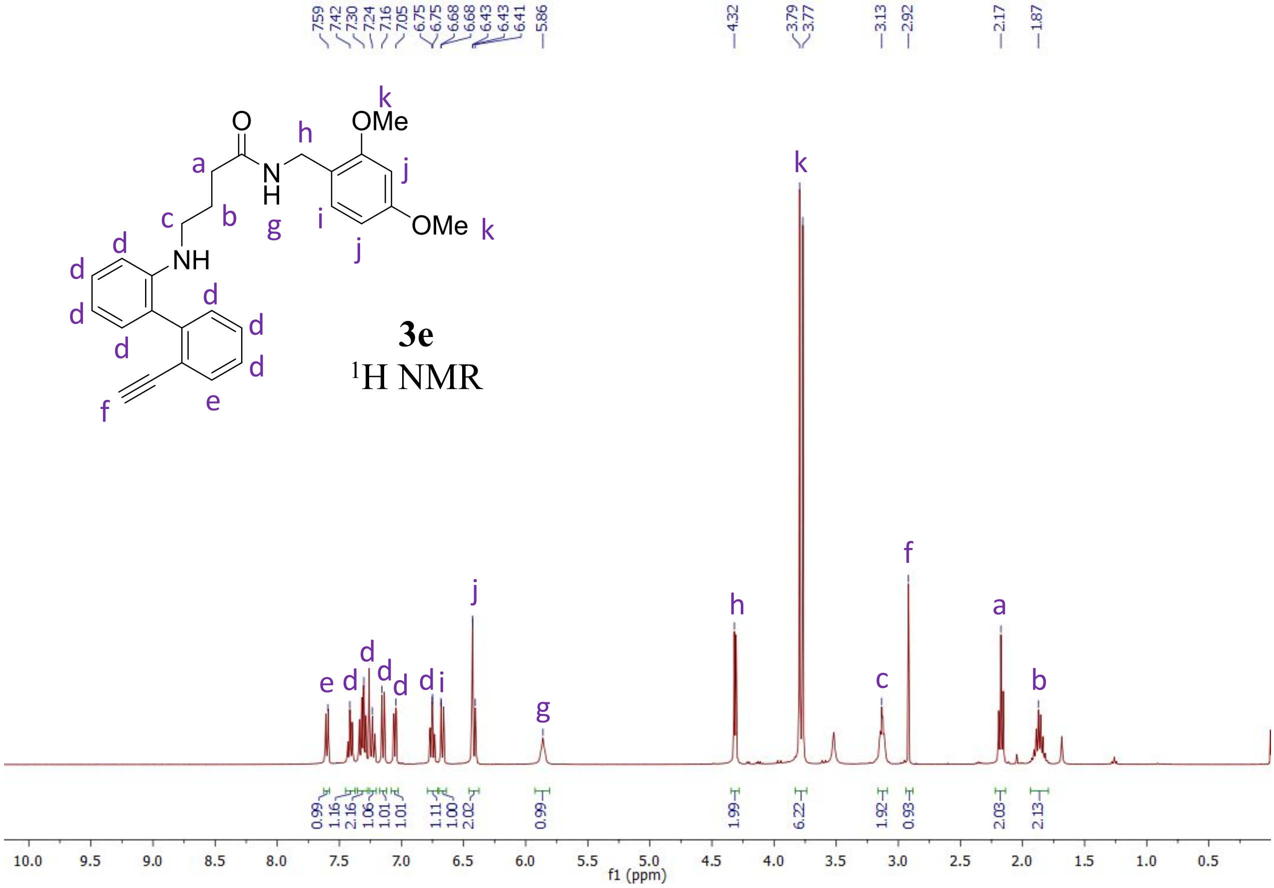
7.59
7.42
7.30
7.24
7.16
7.05
6.75
6.75
6.68
6.68
6.43
6.43
6.41
5.86
4.32
3.79
3.77
3.13
2.92
2.17
1.87
OMe
OMe
NH
3e
¹H NMR
f1 (ppm)

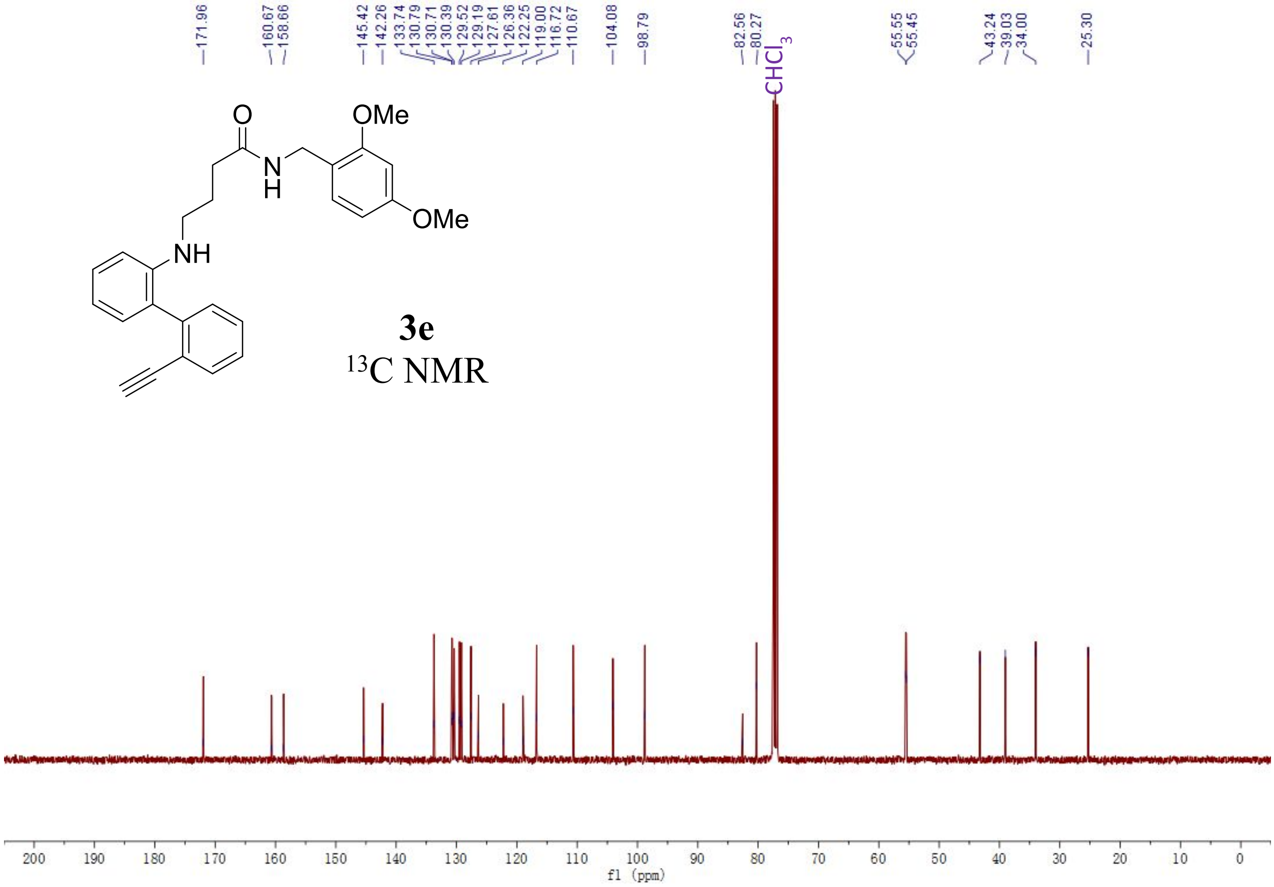
171.96
160.67
158.66
145.42
142.26
133.74
130.79
130.71
130.39
129.52
129.19
127.61
126.36
122.25
119.00
116.72
110.67
104.08
98.79
82.56
80.27
CHCl3
55.55
55.45
43.24
39.03
34.00
25.30
OMe
OMe
NH
3e
¹³C NMR
f1 (ppm)

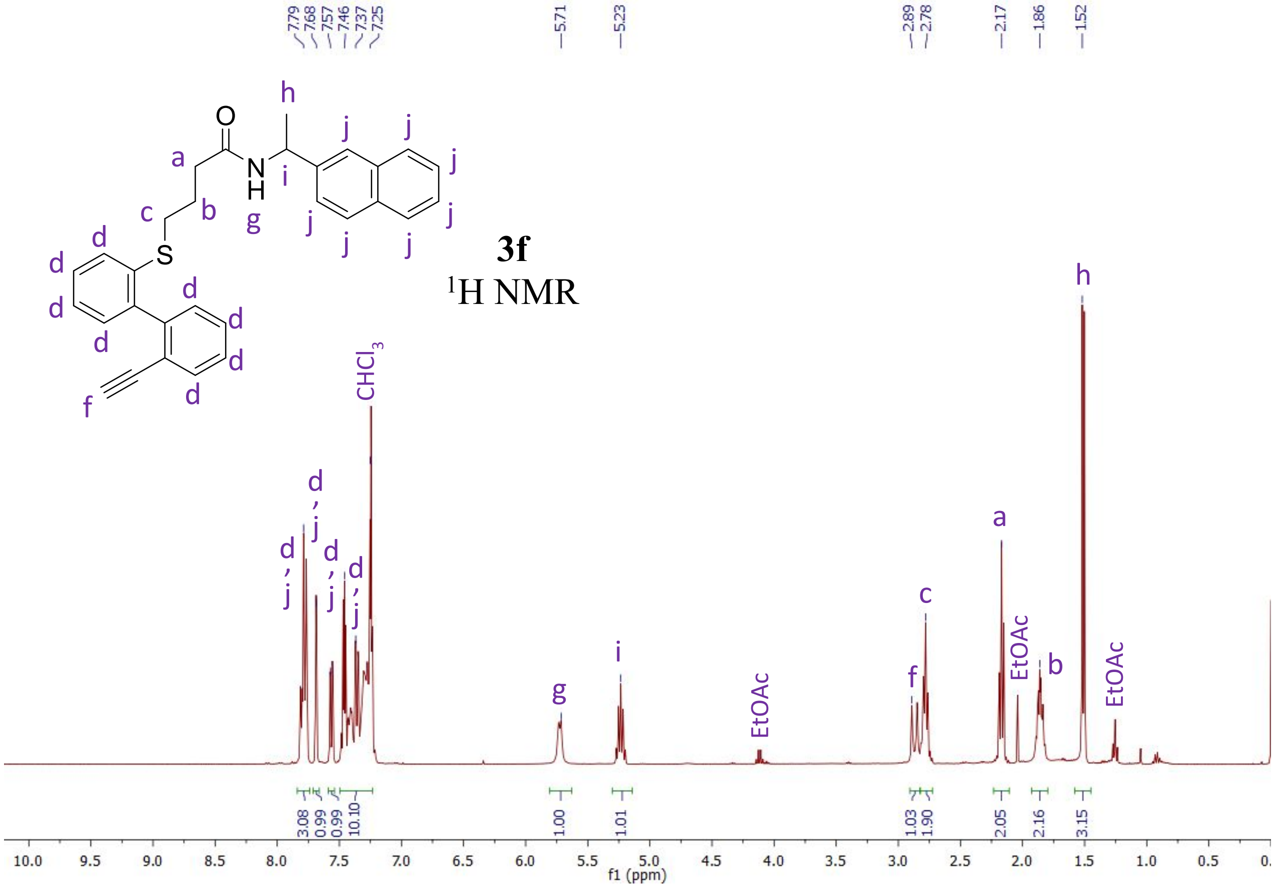
7.79
7.68
7.57
7.46
7.37
7.25
5.71
5.23
2.89
2.78
2.17
1.86
1.52
3f
1H NMR
CHCl3
EtOAc
3.08
0.99
0.99
10.10
1.00
1.01
1.03
1.90
2.05
2.16
3.15
f1 (ppm)

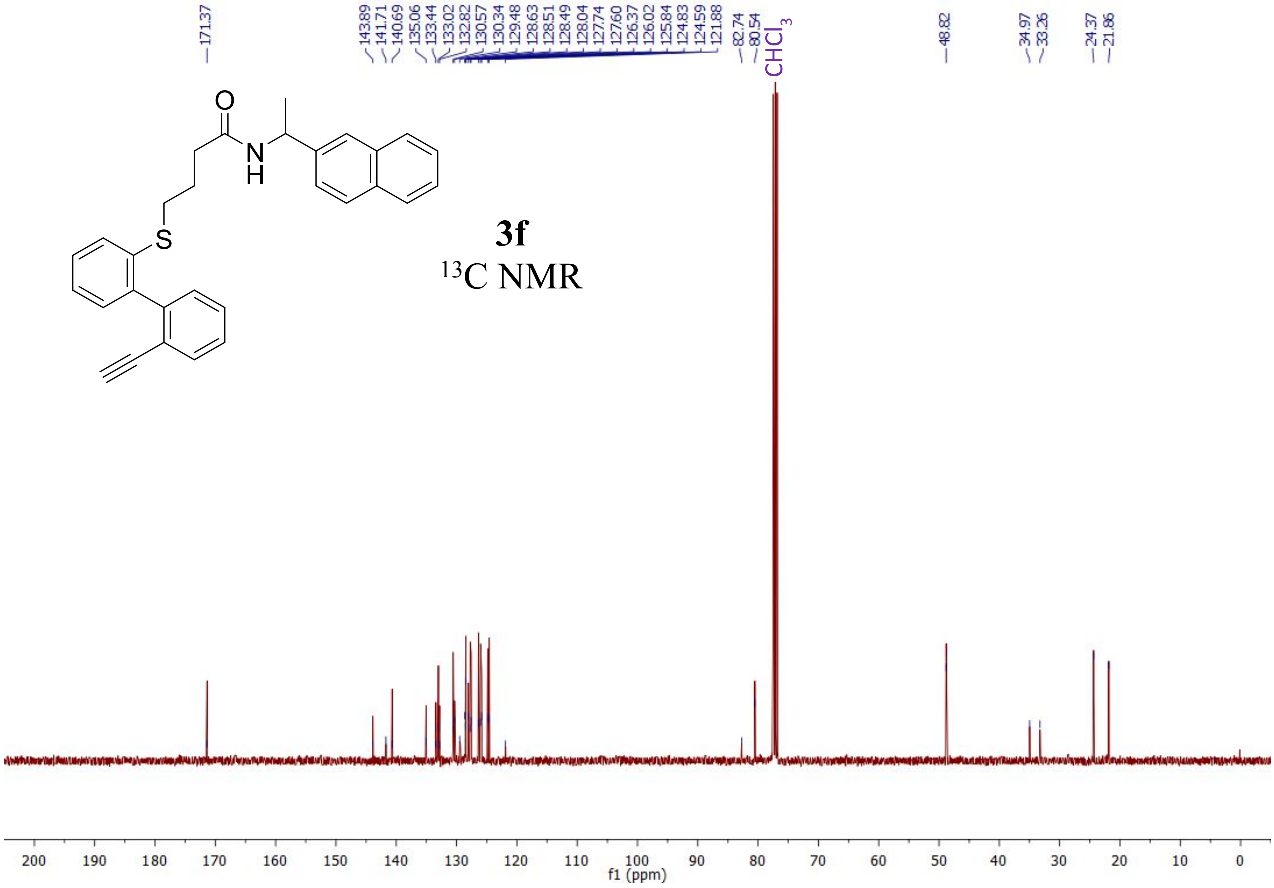
171.37
143.89
141.71
140.69
135.06
133.44
133.02
132.82
130.57
130.34
129.48
128.63
128.51
128.49
128.04
127.74
127.60
126.37
126.02
125.84
124.83
124.59
121.88
82.74
80.54
CHCl3
48.82
34.97
33.26
24.37
21.86
3f
13C NMR
f1 (ppm)

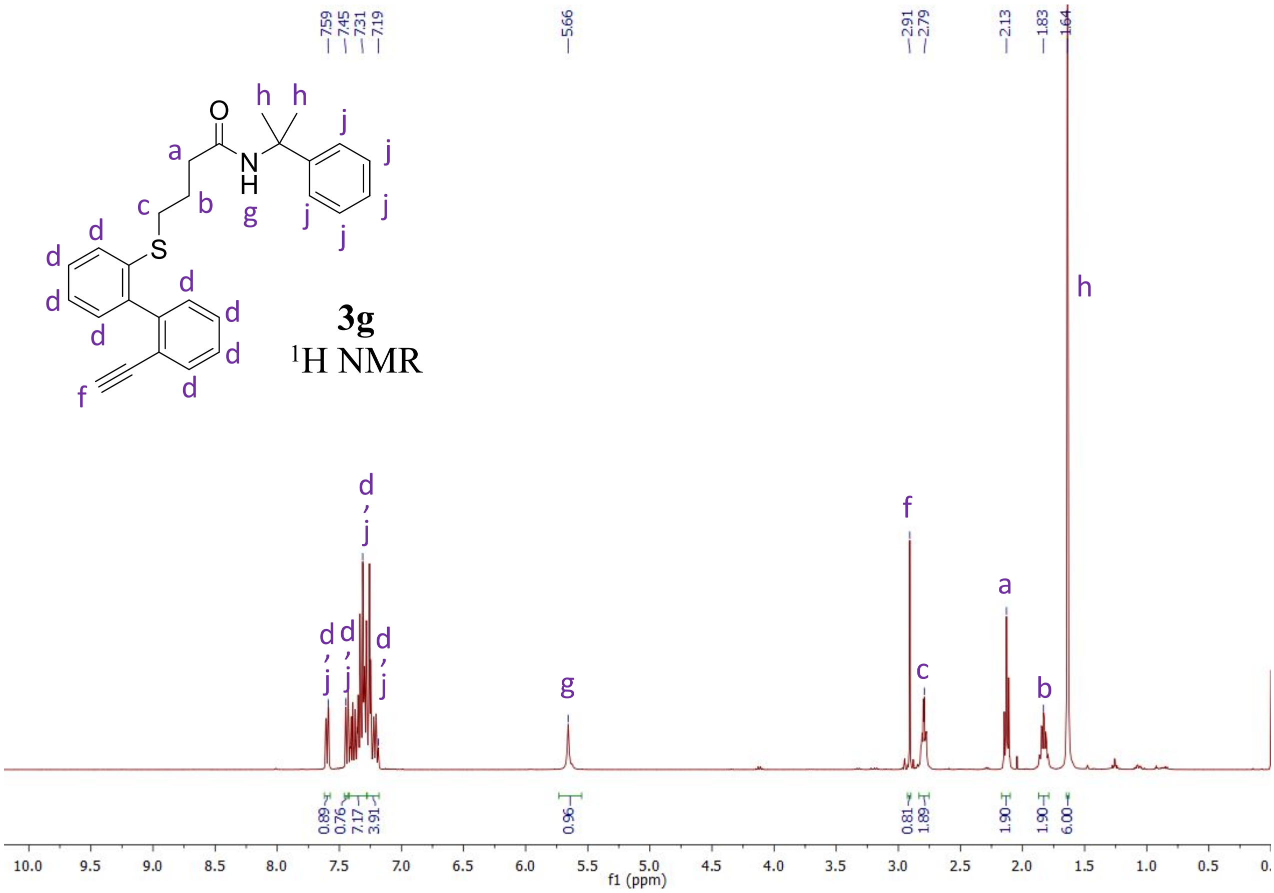
3g
^{1}H NMR
7.59
7.45
7.31
7.19
5.66
2.91
2.79
2.13
1.83
1.64
0.89
0.76
7.17
3.91
0.96
0.81
1.89
1.90
1.90
6.00
f1 (ppm)

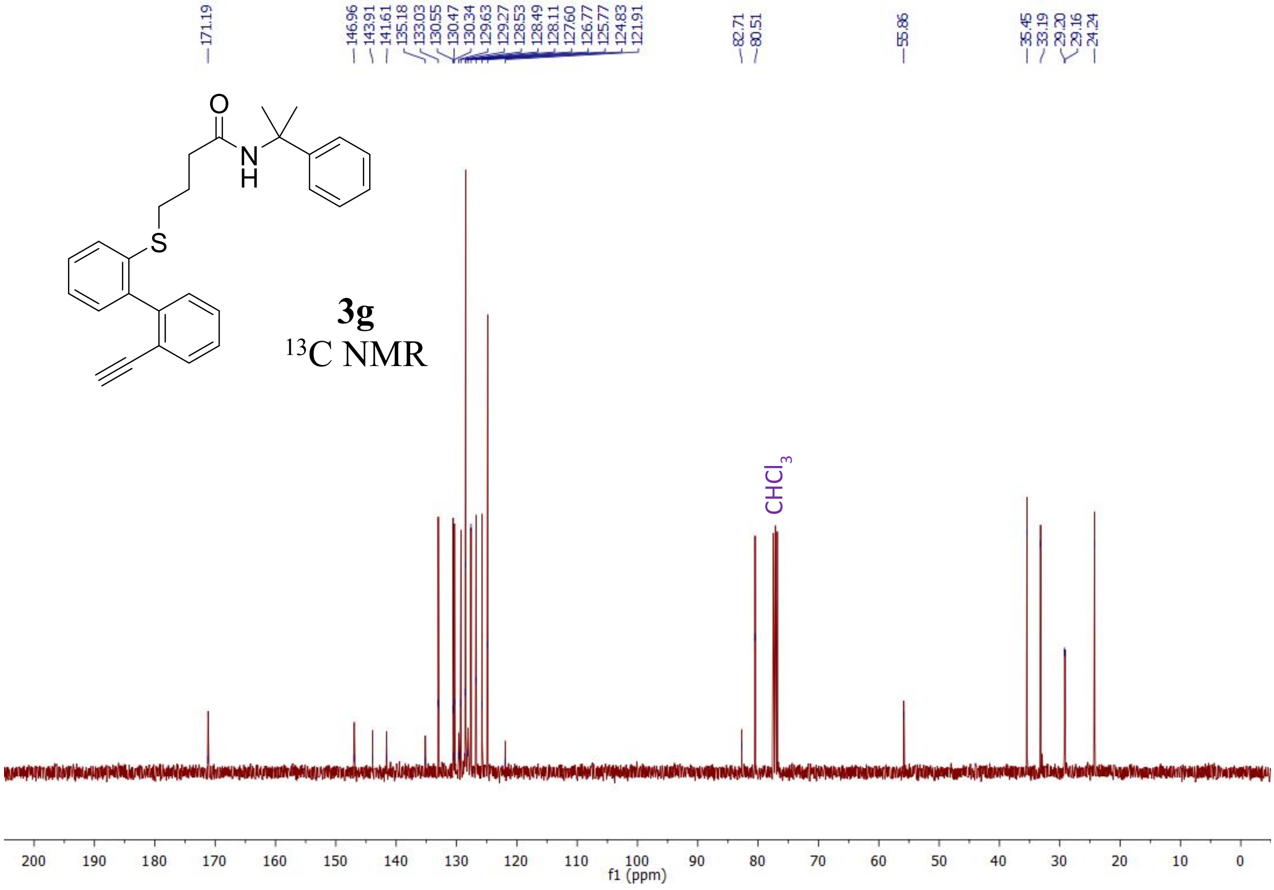
3g
^{13}C NMR
171.19
146.96
143.91
141.61
135.18
133.03
130.55
130.47
130.34
129.63
129.27
128.53
128.49
128.11
127.60
126.77
125.77
124.83
121.91
82.71
80.51
55.86
35.45
33.19
29.20
29.16
24.24
CHCl3
f1 (ppm)

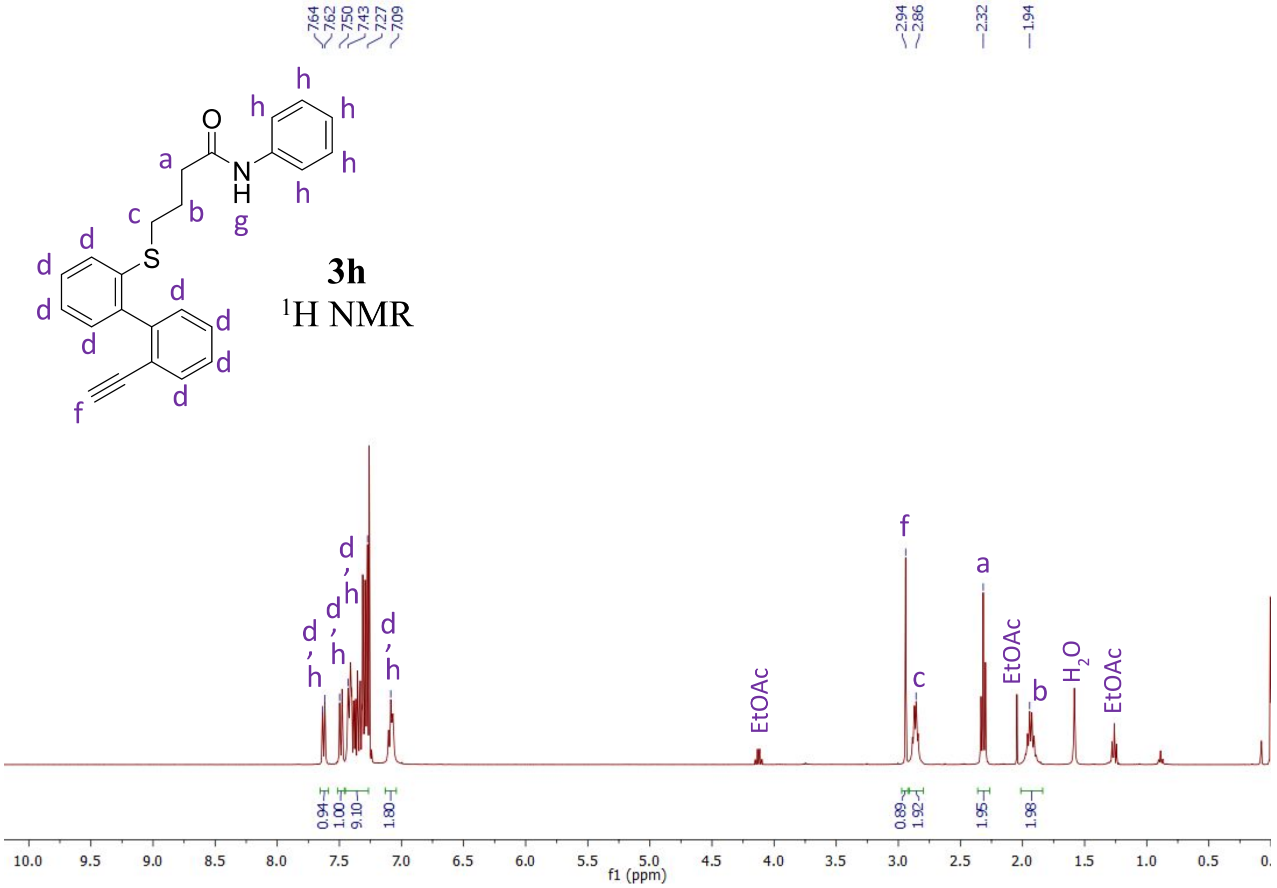
3h
1H NMR
7.64
7.62
7.50
7.43
7.27
7.09
2.94
2.86
2.32
1.94
EtOAc
H2O
0.94
1.00
9.10
1.80
0.89
1.92
1.95
1.98
f1 (ppm)

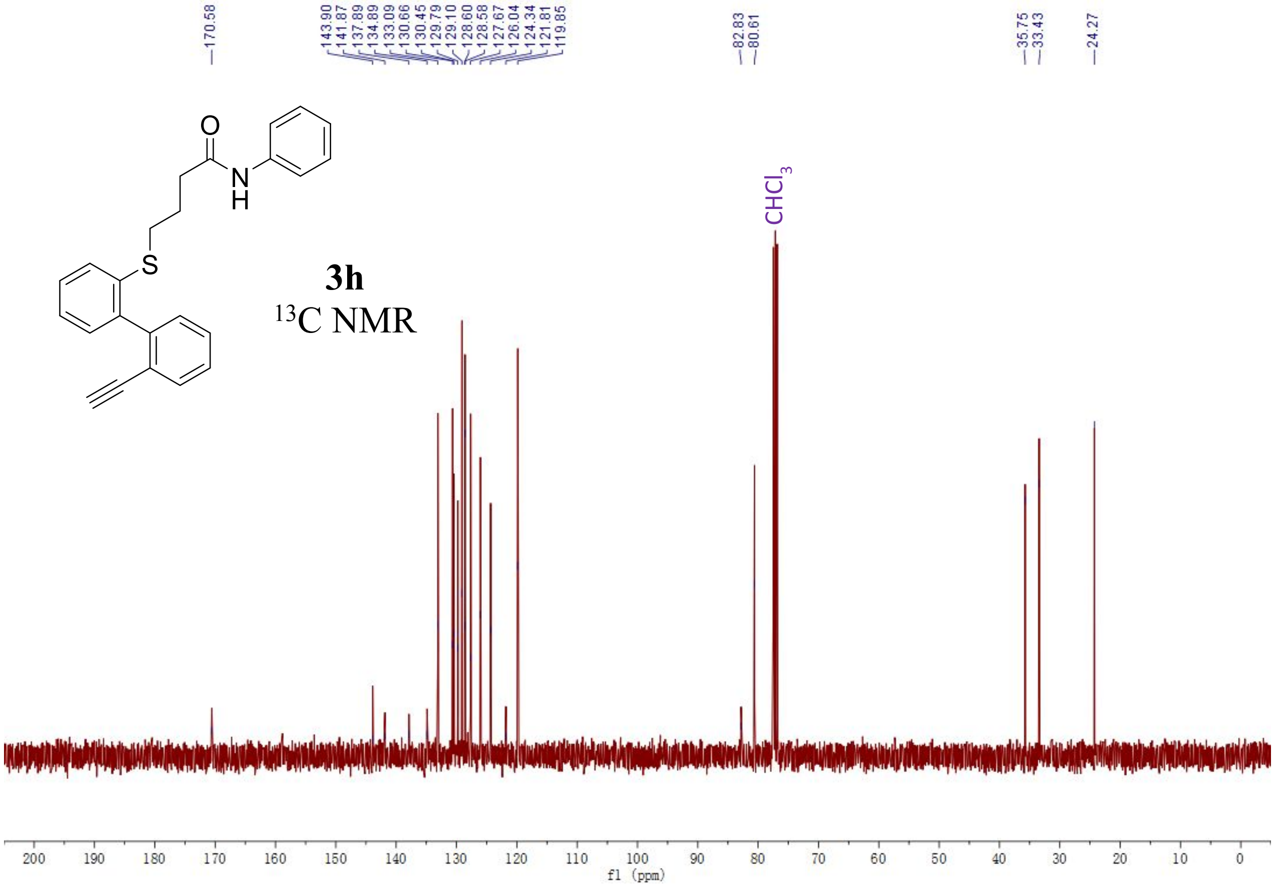
3h
13C NMR
170.58
143.90
141.87
137.89
134.89
133.09
130.66
130.45
129.79
129.10
128.60
128.58
127.67
126.04
124.34
121.81
119.85
82.83
80.61
35.75
33.43
24.27
CHCl3
f1 (ppm)

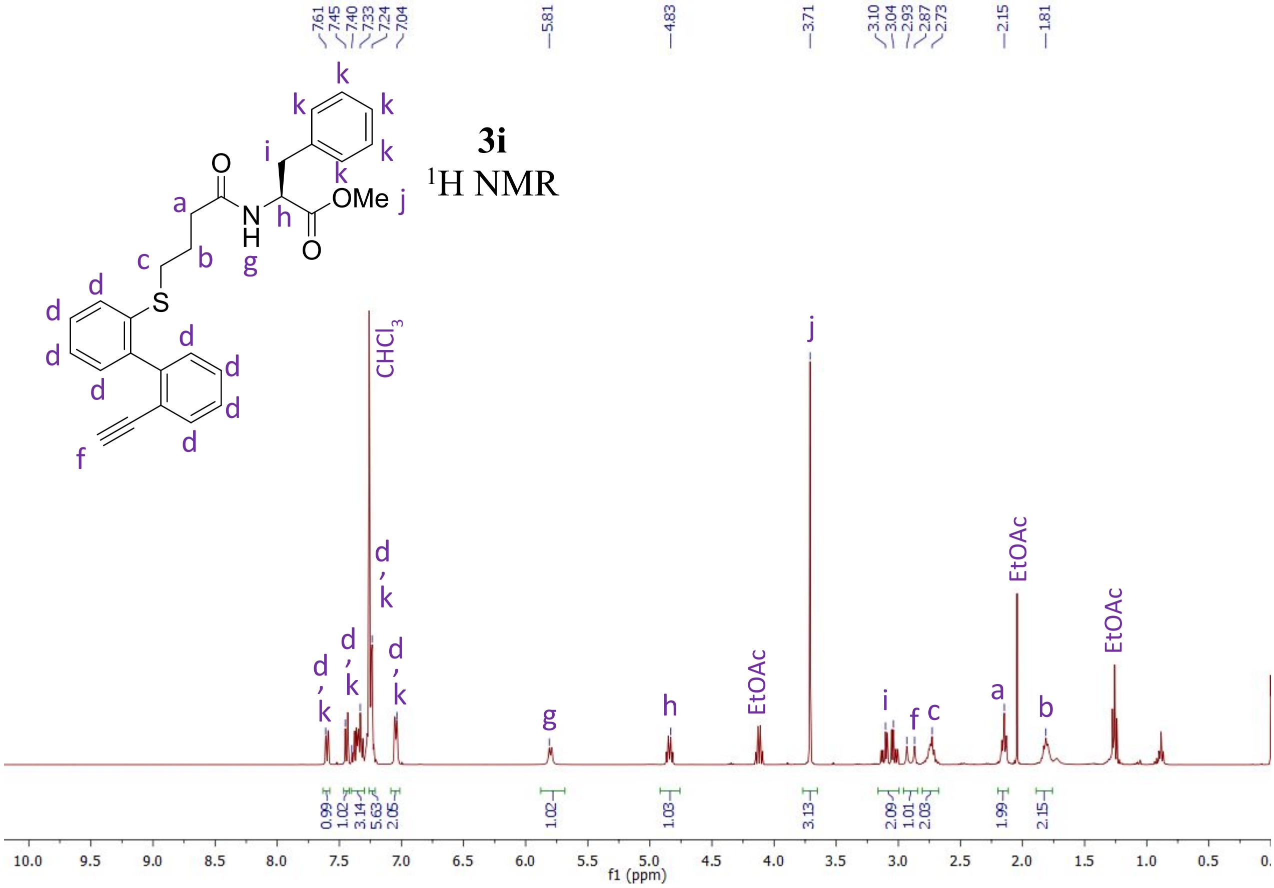

7.61
7.45
7.40
7.33
7.24
7.04
5.81
4.83
3.71
3.10
3.04
2.93
2.87
2.73
2.15
1.81
3i
^{1}H NMR
OMe
O
N
H
S
CHCl$_3$
EtOAc
a b c d e f g h i j k
0.99
1.02
3.14
5.63
2.05
1.02
1.03
3.13
2.09
1.01
2.03
1.99
2.15
f1 (ppm)

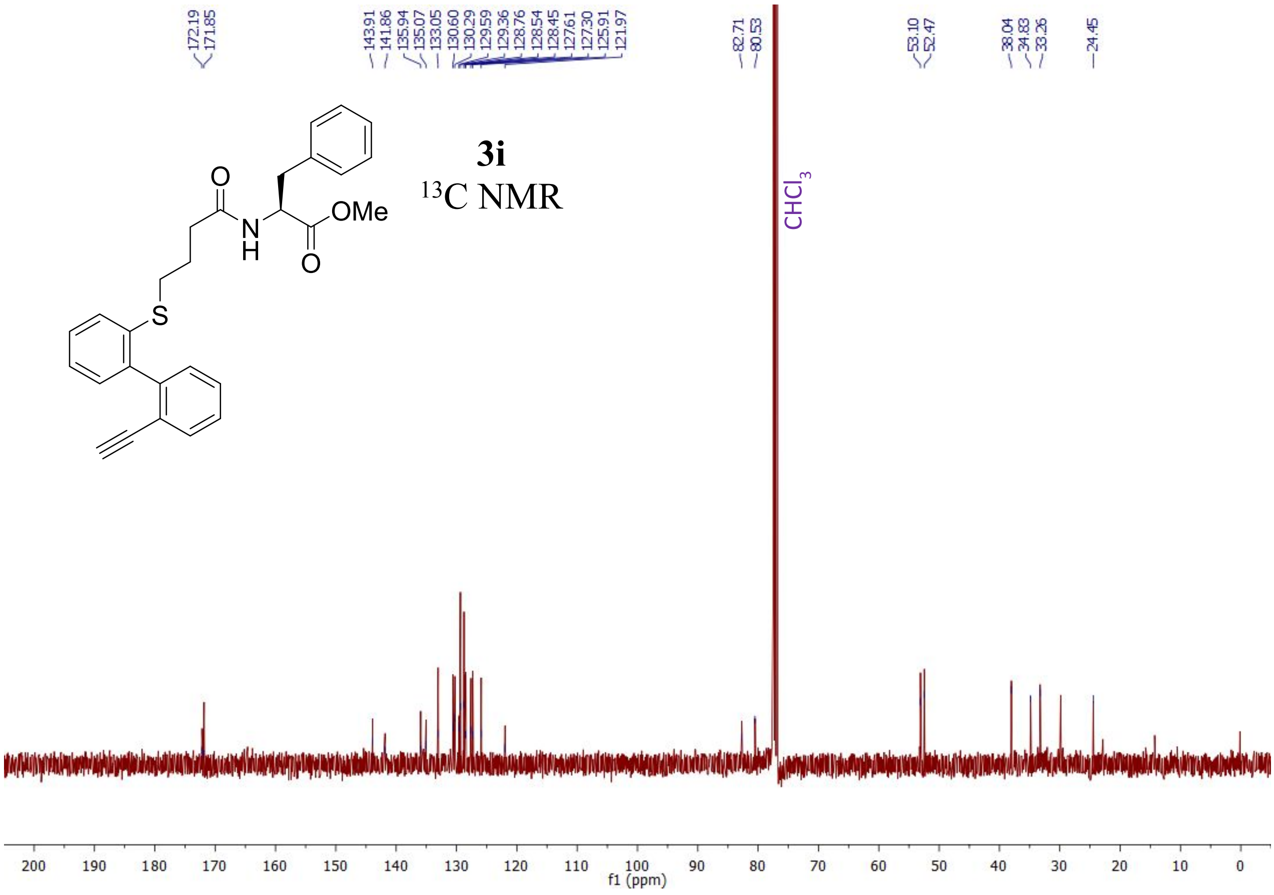

172.19
171.85
143.91
141.86
135.94
135.07
133.05
130.60
130.29
129.59
129.36
128.76
128.54
128.45
127.61
127.30
125.91
121.97
82.71
80.53
53.10
52.47
38.04
34.83
33.26
24.45
3i
^{13}C NMR
OMe
CHCl$_3$
f1 (ppm)

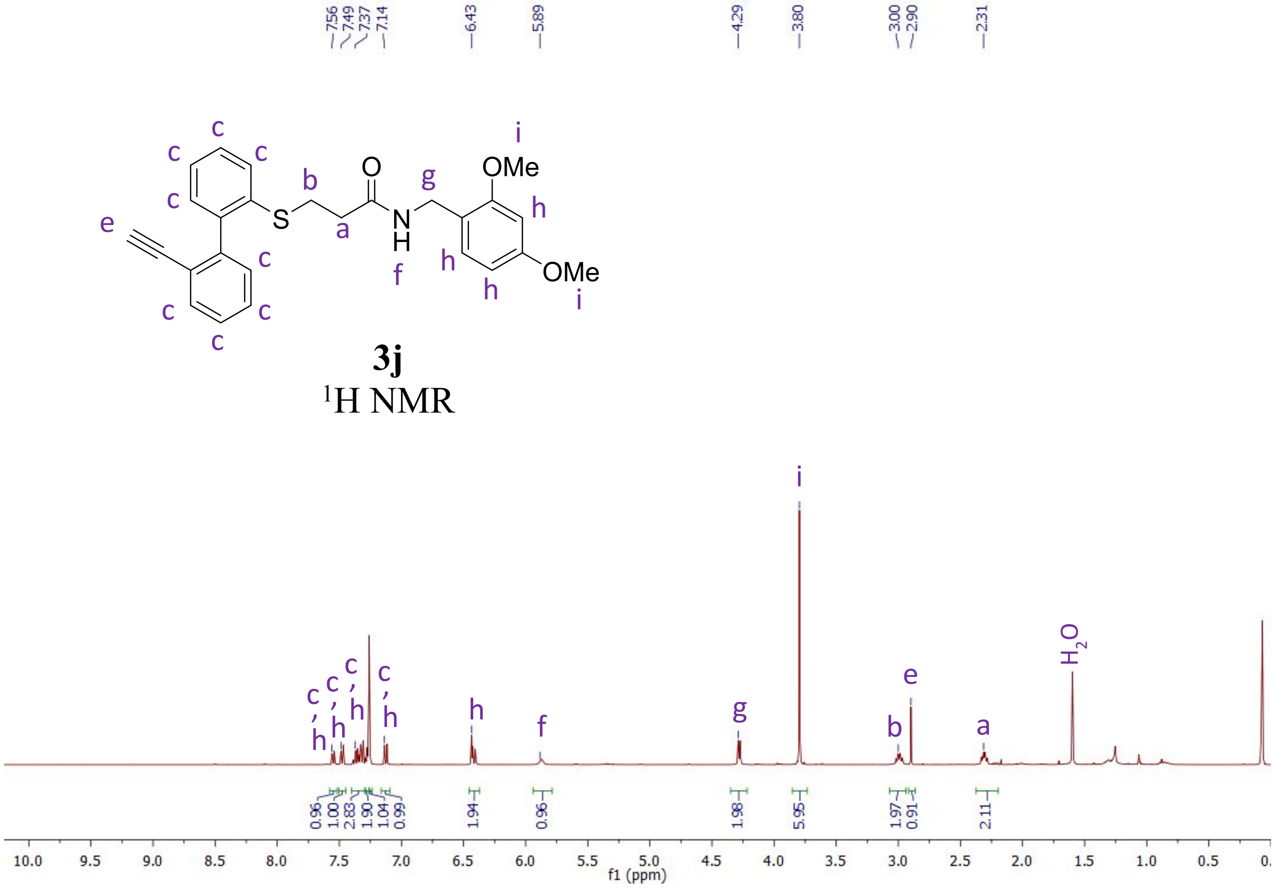
7.56
7.49
7.37
7.14
6.43
5.89
4.29
3.80
3.00
2.90
2.31
3j
1H NMR
i
e
g
b
a
f
h
c
H2O
0.96
1.00
2.83
1.90
1.04
0.99
1.94
0.96
1.98
5.95
1.97
0.91
2.11
f1 (ppm)

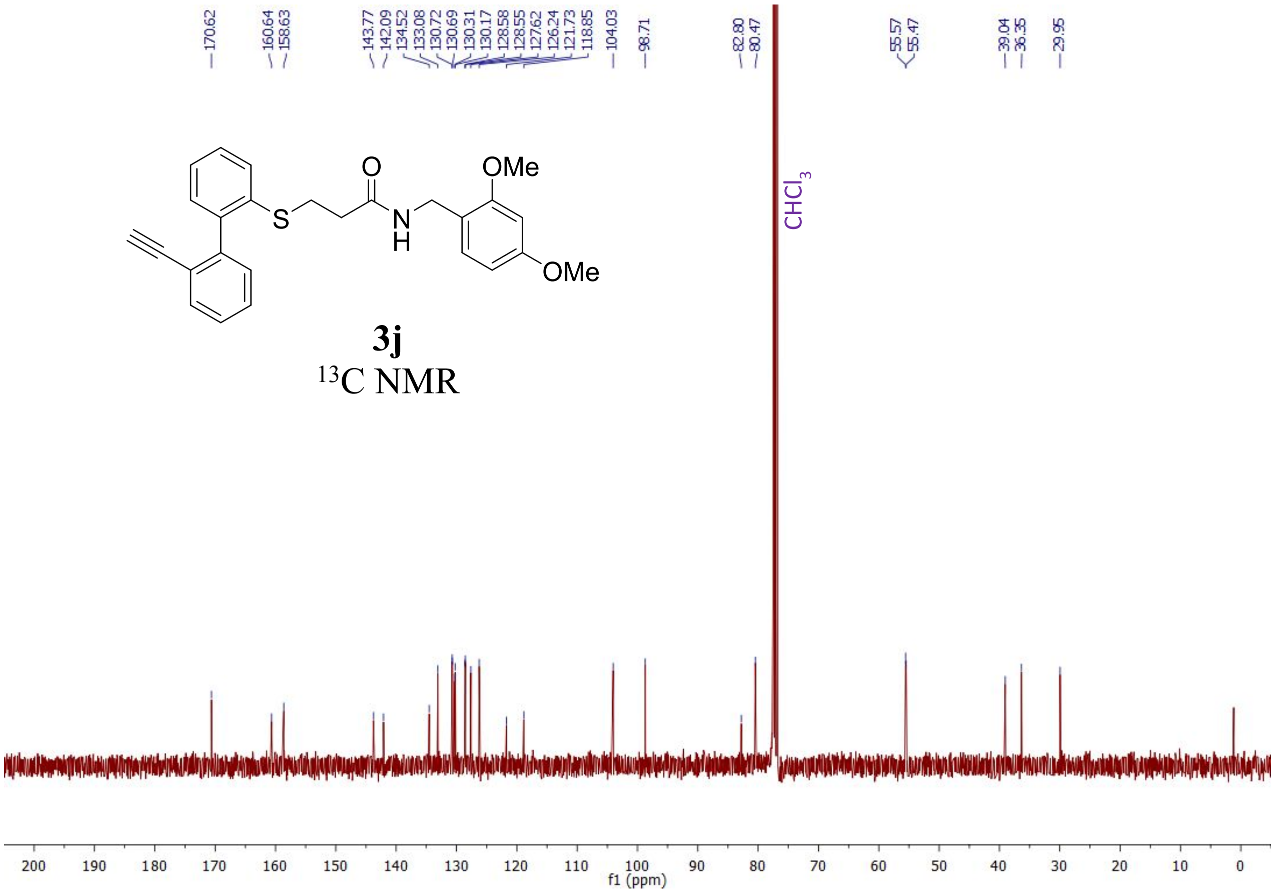
170.62
160.64
158.63
143.77
142.09
134.52
133.08
130.72
130.69
130.31
130.17
128.58
128.55
127.62
126.24
121.73
118.85
104.03
98.71
82.80
80.47
55.57
55.47
39.04
36.35
29.95
3j
13C NMR
CHCl3
f1 (ppm)

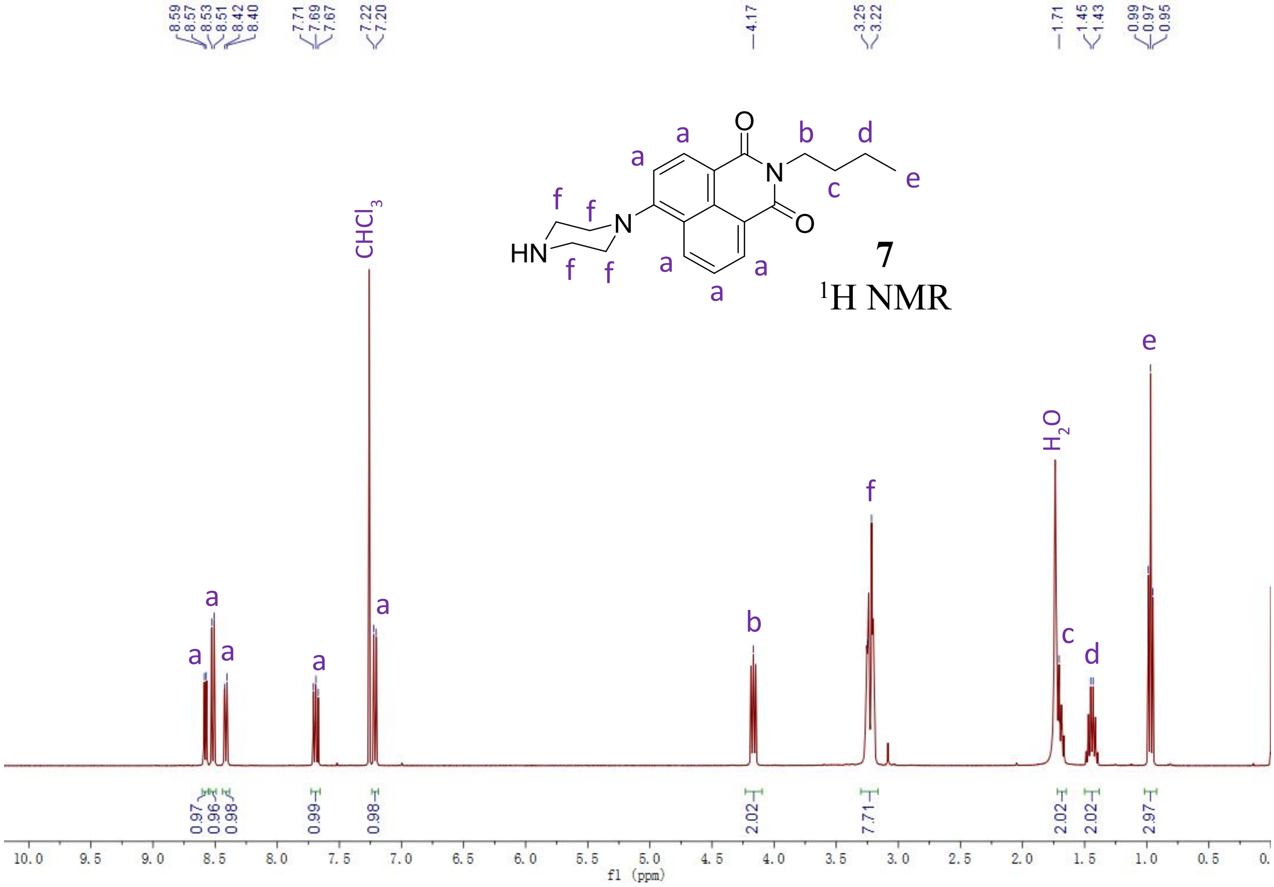
8.59
8.57
8.53
8.51
8.42
8.40
7.71
7.69
7.67
7.22
7.20
4.17
3.25
3.22
1.71
1.45
1.43
0.99
0.97
0.95
7
^{1}H NMR
CHCl$_3$
H_2O
a
b
c
d
e
f
0.97
0.96
0.98
0.99
0.98
2.02
7.71
2.02
2.02
2.97
f1 (ppm)

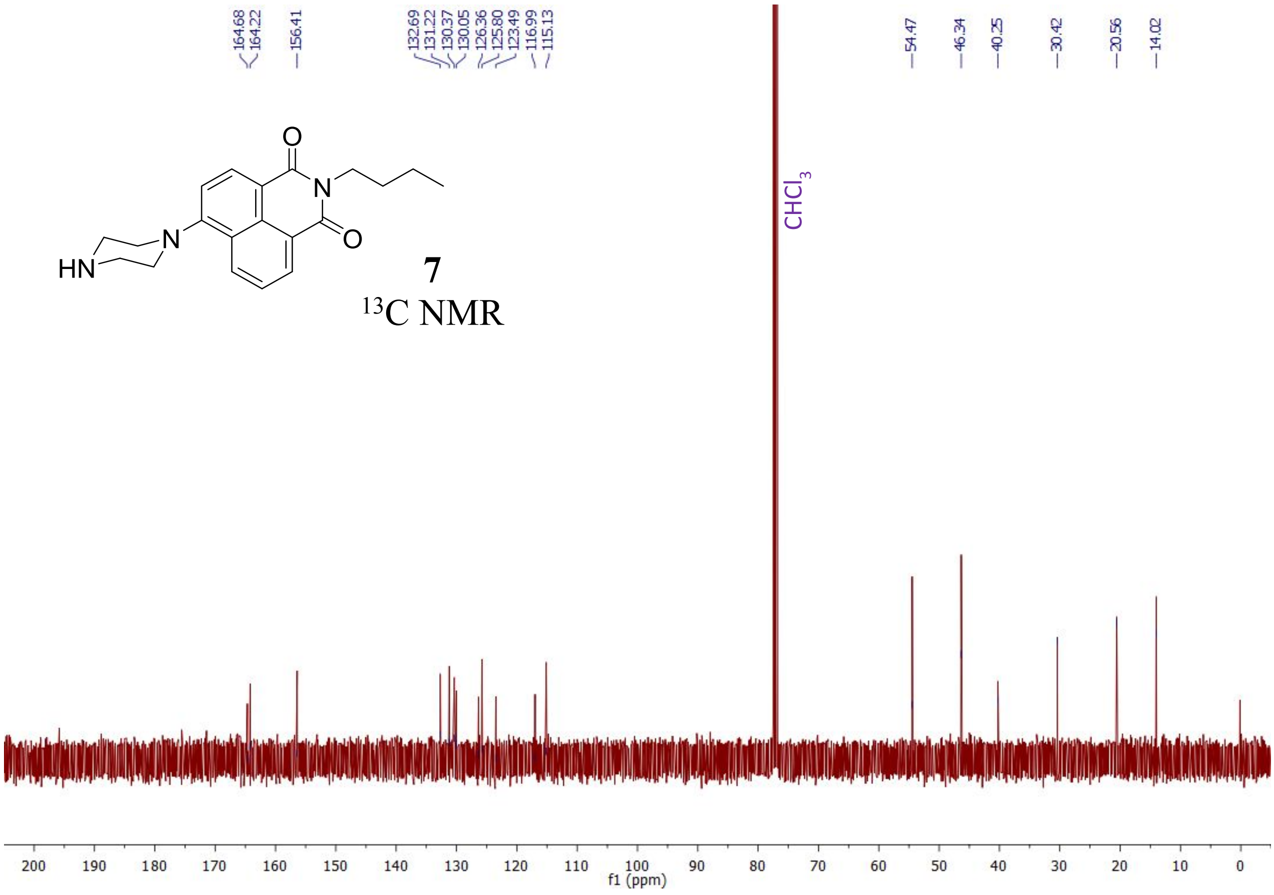
164.68
164.22
156.41
132.69
131.22
130.37
130.05
126.36
125.80
123.49
116.99
115.13
54.47
46.34
40.25
30.42
20.56
14.02
7
^{13}C NMR
CHCl$_3$
f1 (ppm)

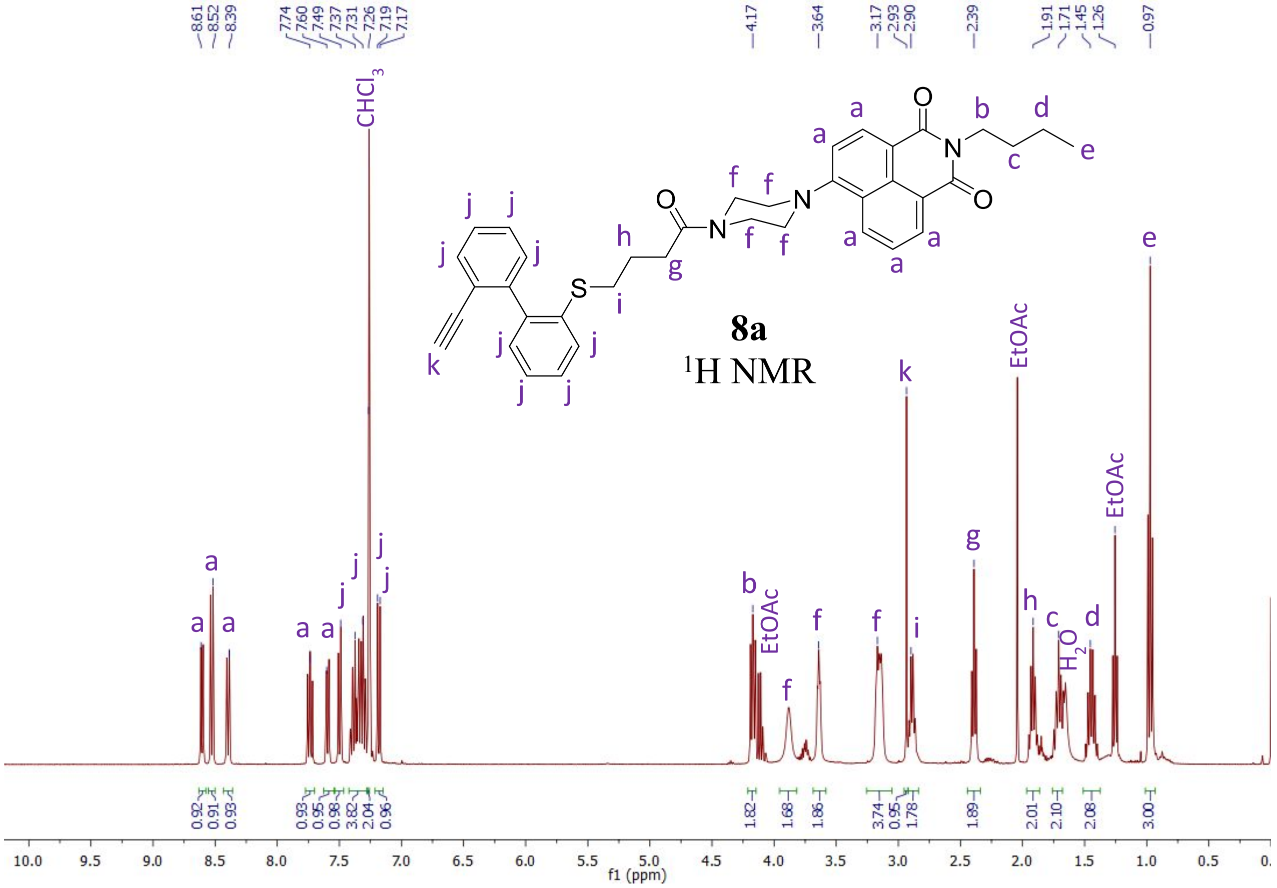
8.61
8.52
8.39
7.74
7.60
7.49
7.37
7.31
7.26
7.19
7.17
4.17
3.64
3.17
2.93
2.90
2.39
1.91
1.71
1.45
1.26
0.97
CHCl3
EtOAc
H2O
8a
1H NMR
f1 (ppm)

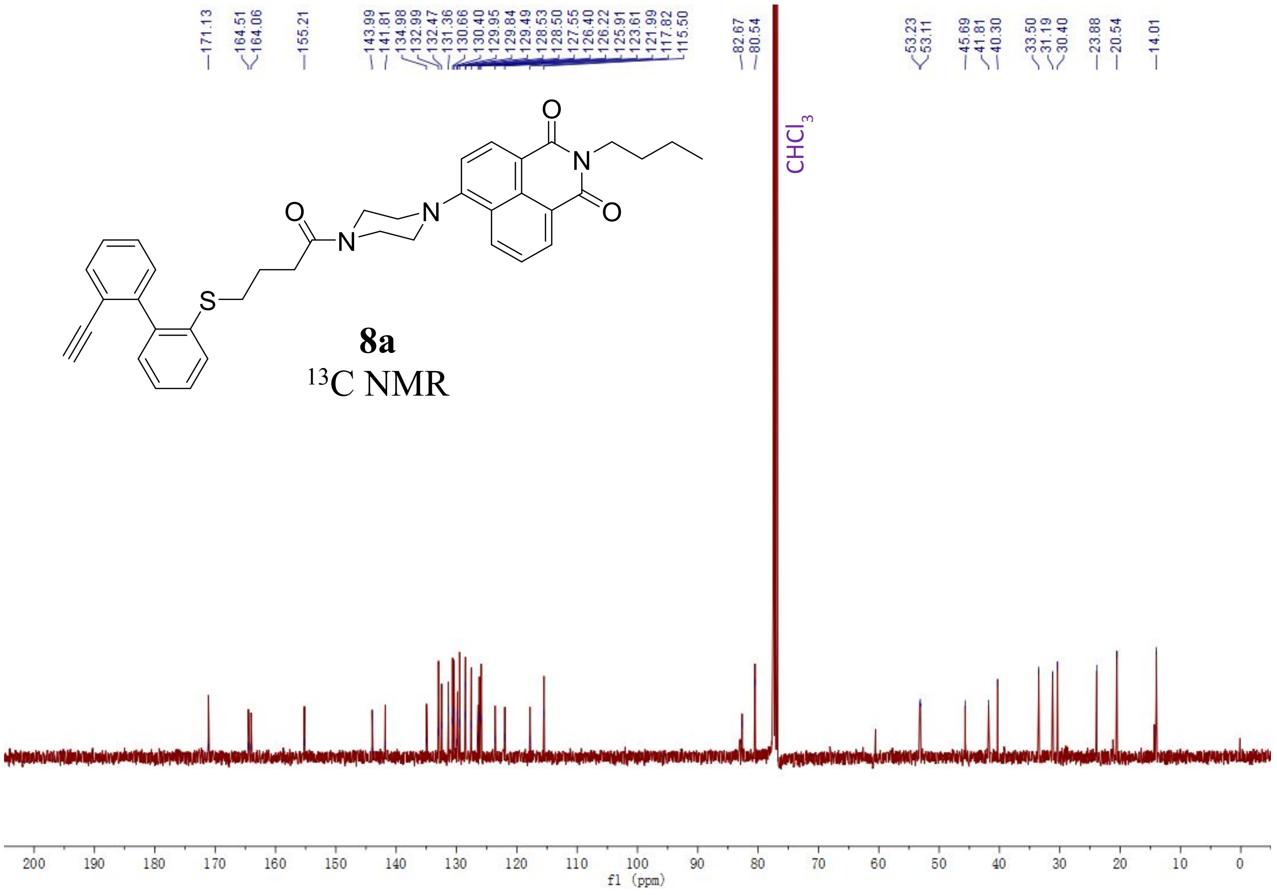
171.13
164.51
164.06
155.21
143.99
141.81
134.98
132.99
132.47
131.36
130.66
130.40
129.95
129.84
129.49
128.53
128.50
127.55
126.40
126.22
125.91
123.61
121.99
117.82
115.50
82.67
80.54
53.23
53.11
45.69
41.81
40.30
33.50
31.19
30.40
23.88
20.54
14.01
CHCl3
8a
13C NMR
f1 (ppm)

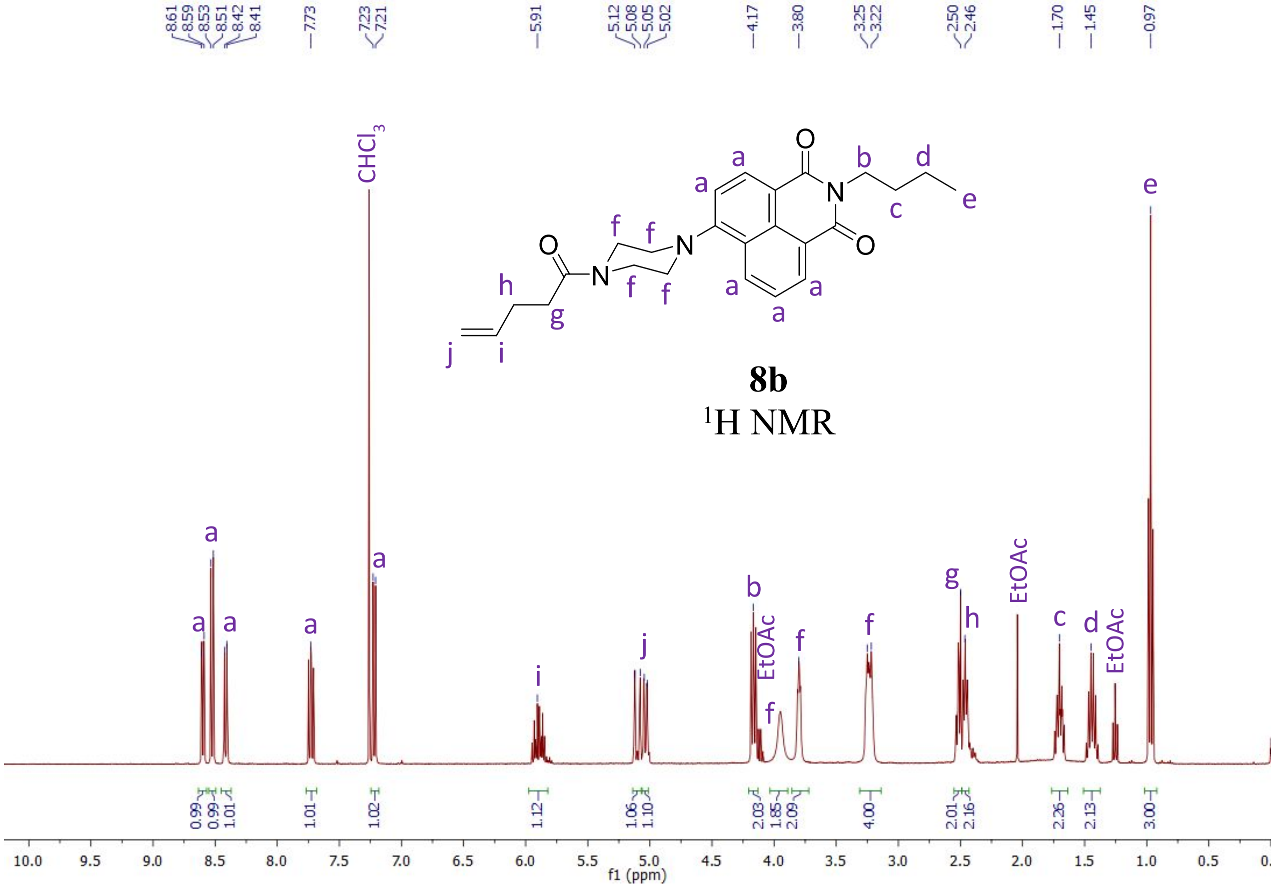

8b

^{1}H NMR

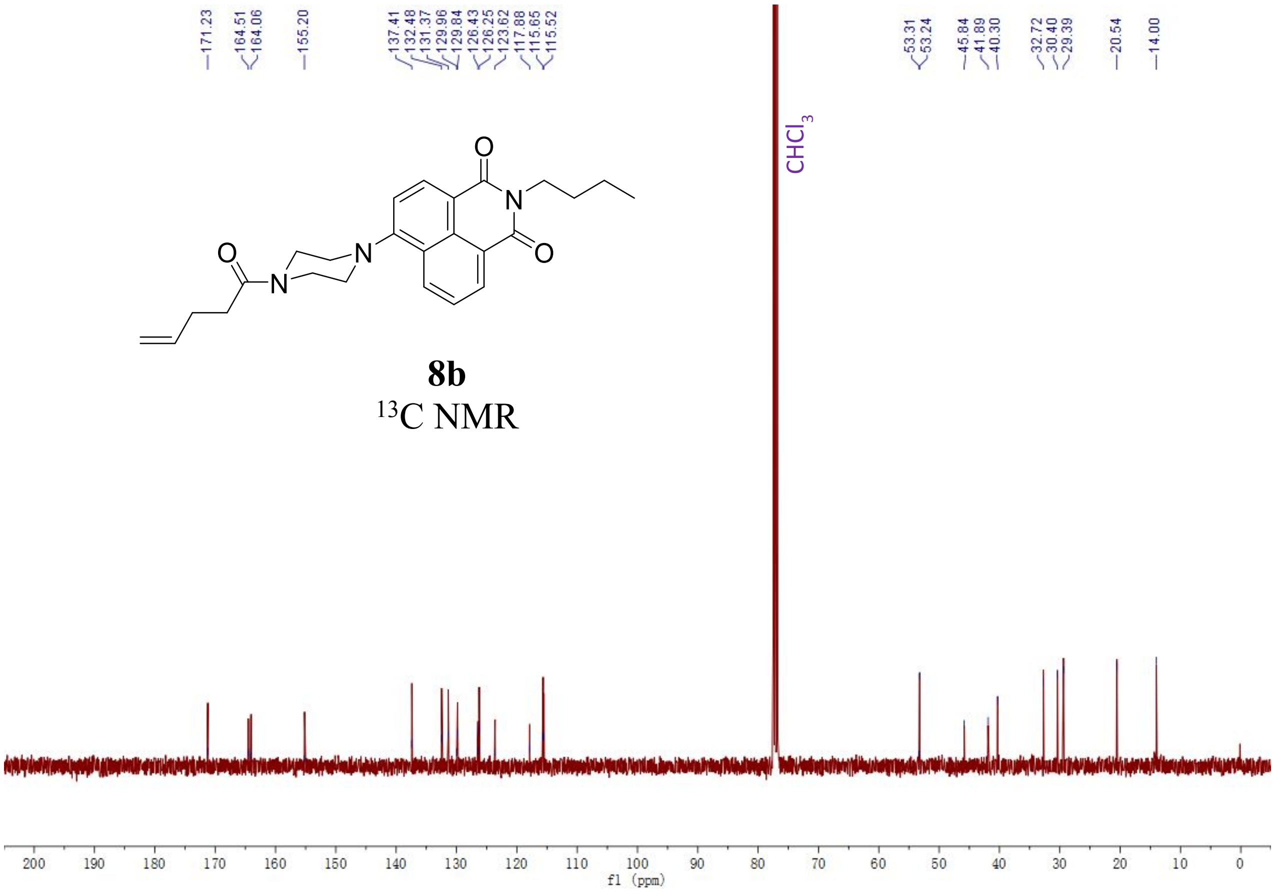

8b

^{13}C NMR

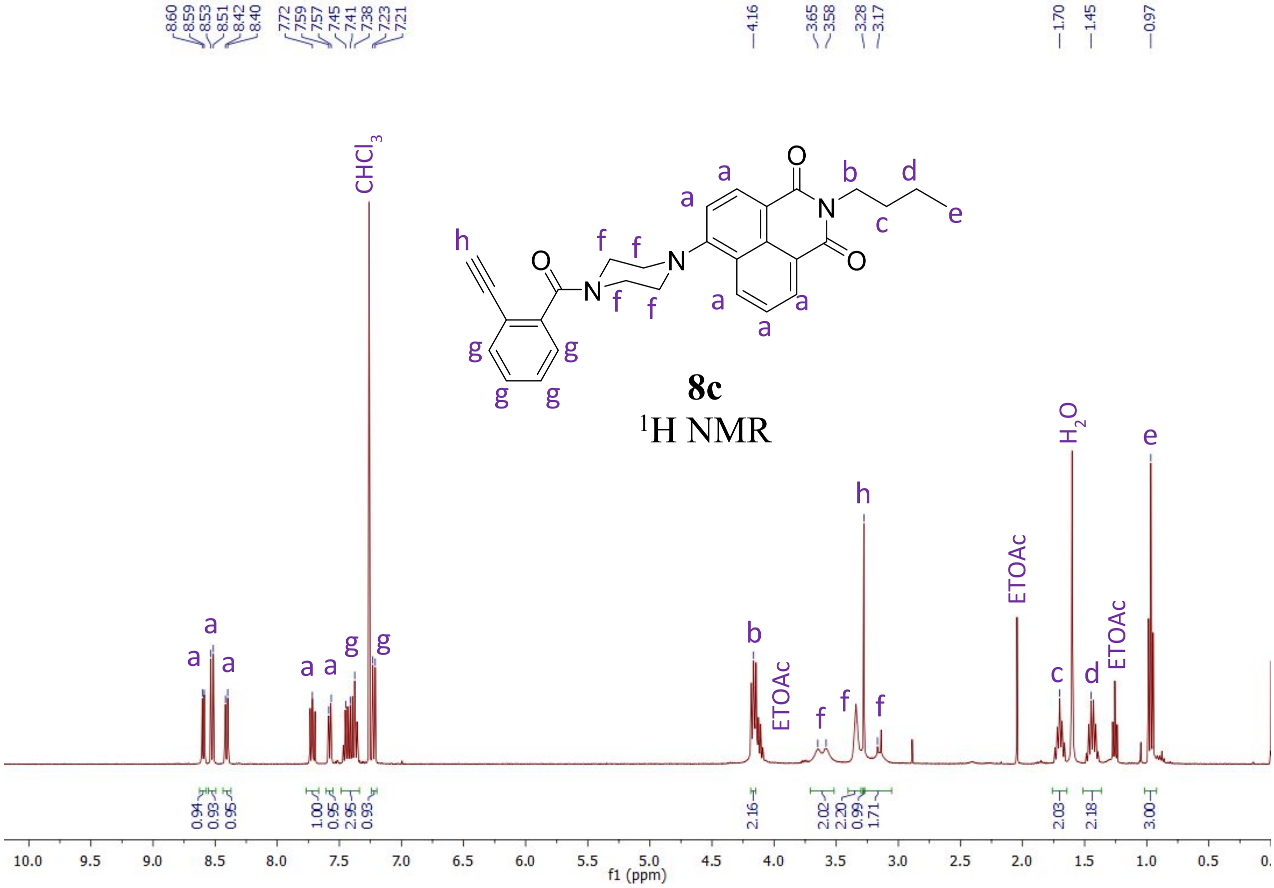
8.60
8.59
8.53
8.51
8.42
8.40
7.72
7.59
7.57
7.45
7.41
7.38
7.23
7.21
4.16
3.65
3.58
3.28
3.17
1.70
1.45
0.97
CHCl3
H2O
ETOAc
8c
1H NMR
f1 (ppm)

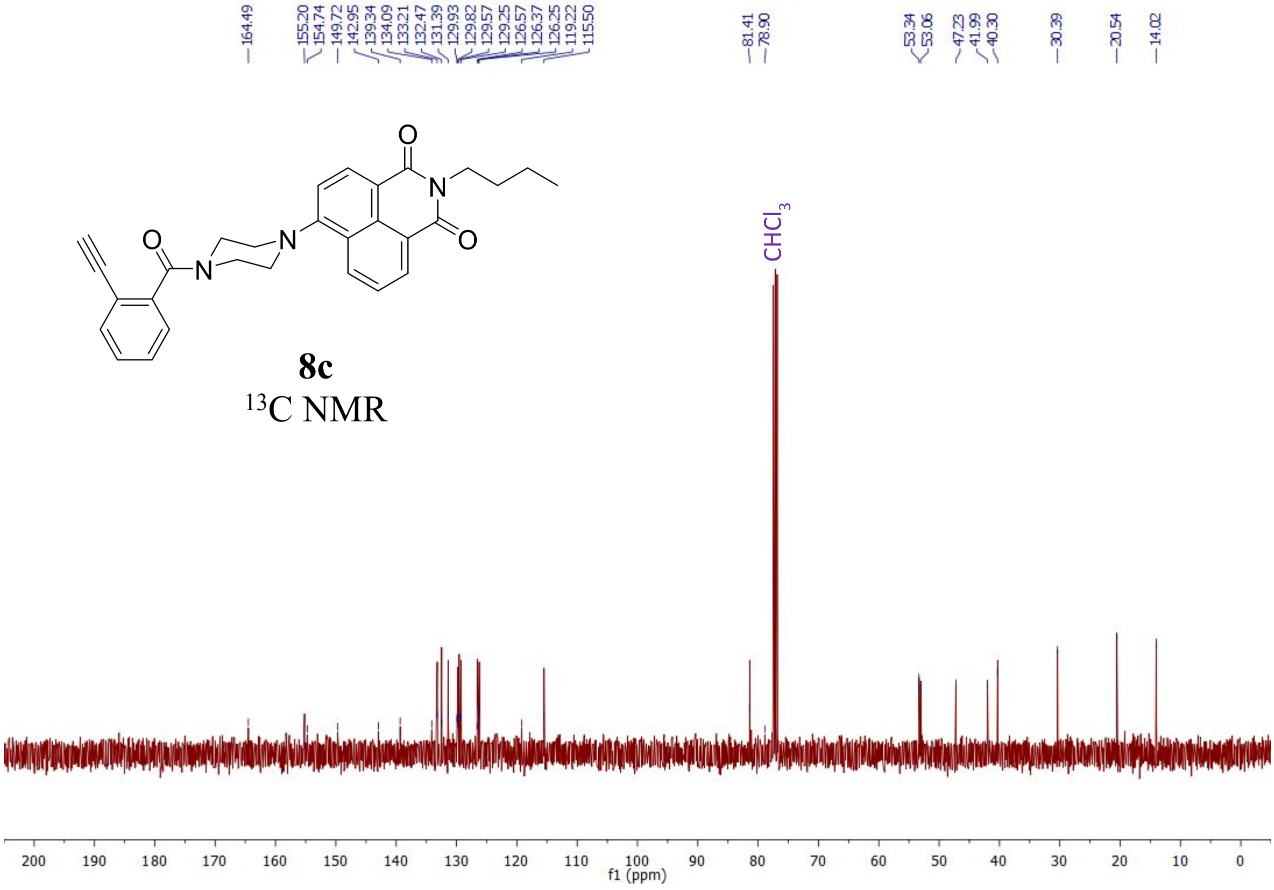
164.49
155.20
154.74
149.72
142.95
139.34
134.09
133.21
132.47
131.39
129.93
129.82
129.57
129.25
126.57
126.37
126.25
119.22
115.50
81.41
78.90
53.34
53.06
47.23
41.99
40.30
30.39
20.54
14.02
CHCl3
8c
13C NMR
f1 (ppm)

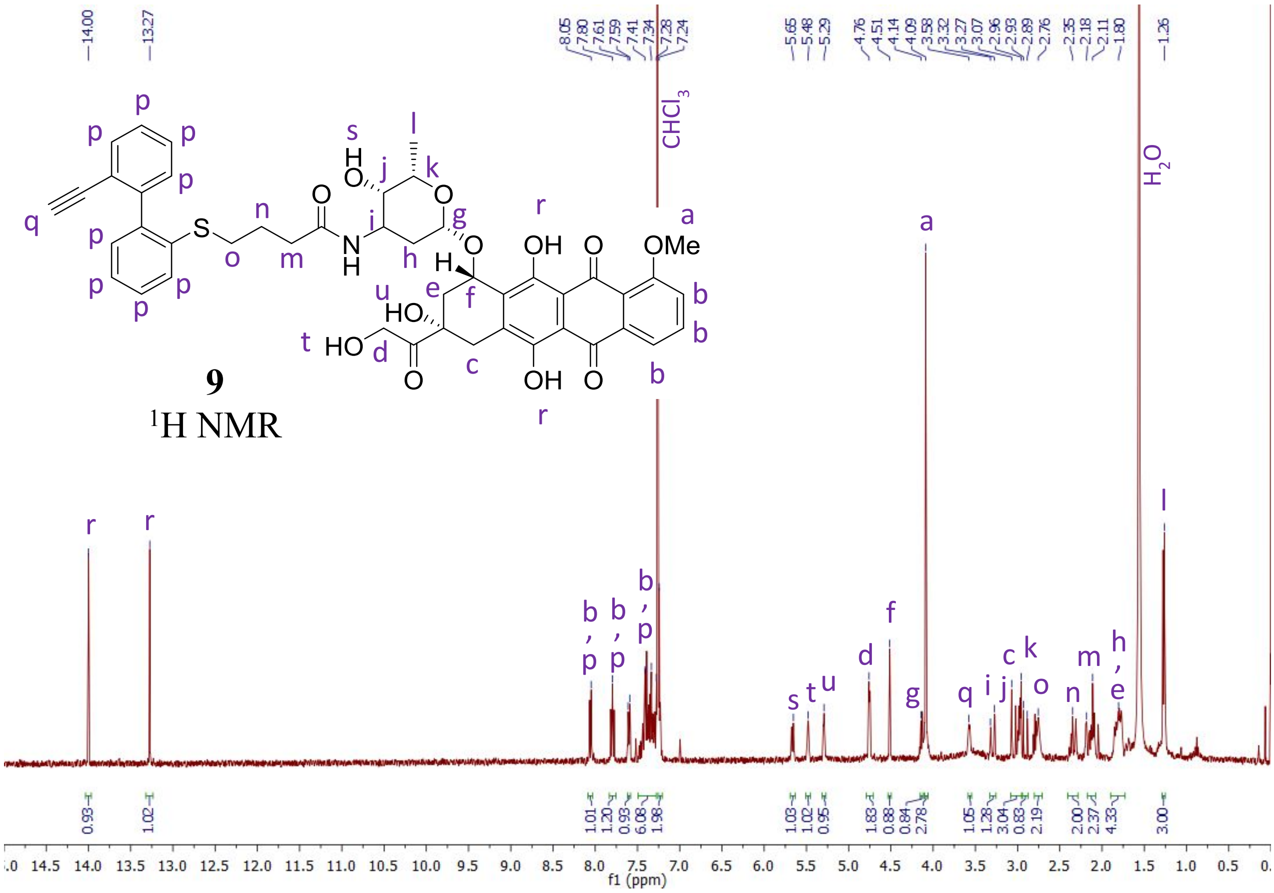
9
1H NMR
CHCl3
H2O
f1 (ppm)

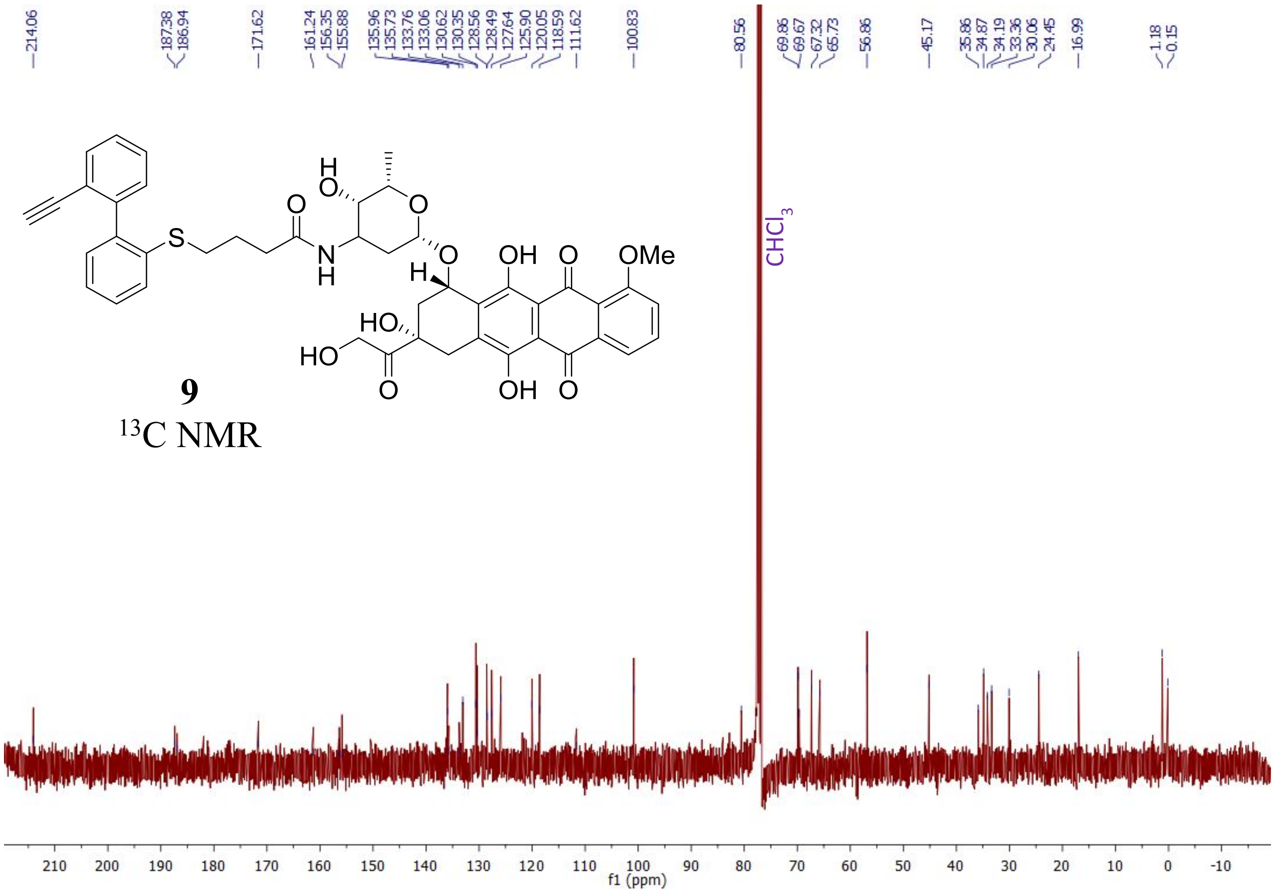
9
13C NMR
CHCl3
f1 (ppm)

9. References

(1) Huang, J.; Liu, Y.; Han, X.; Du, J.; Vong, K. Bioorthogonal gold-catalyzed aldehyde release and its adaptation for prodrug therapy using multivalent lectin-directed artificial metalloenzymes. *JACS Au* **2026**, *6* (1), 389–402.

(2) Rezsnyak, C. E.; Jochen, A.; D., A. J.; and Moncho, S. Reactions of gold(III) complexes with alkenes in aqueous media: generation of bis-(β-hydroxyalkyl)gold(III) complexes. *J. Coord. Chem.* **2013**, *66* (7), 1153-1165.

(3) Unnikrishnan, V. B.; Sabatino, V.; Amorim, F.; Estrada, M. F.; Navo, C. D.; Jimenez-Oses, G.; Fior, R.; Bernardes, G. J. L. Gold(III)-Induced Amide Bond Cleavage In Vivo: A Dual Release Strategy via π-Acid Mediated Allyl Substitution. *J. Am. Chem. Soc.* **2024**, *146* (33), 23240-23251.

(4) Xin, F.; Zhao, J.; Wang, X.; Wang, H.; Wang, H.; Xing, M.; Fu, Y.; Tian, Y.; Tian, Y. Investigating the AIE and water sensing properties of a concise naphthalimide fluorophore. *Spectrochim. Acta A Mol. Biomol. Spectrosc.* **2023**, *296*, 122621.

(5) Fery-Forgues, S.; Lavabre, D. Are Fluorescence Quantum Yields So Tricky to Measure? A Demonstration Using Familiar Stationery Products. *J. Chem. Educ.* **1999**, *76* (9), 1260.

(6) Aminabhavi, T. M.; Gopalakrishna, B. Density, Viscosity, Refractive Index, and Speed of Sound in Aqueous Mixtures of N,N-Dimethylformamide, Dimethyl Sulfoxide, N,N-Dimethylacetamide, Acetonitrile, Ethylene Glycol, Diethylene Glycol, 1,4-Dioxane, Tetrahydrofuran, 2-Methoxyethanol, and 2-Ethoxyethanol at 298.15 K. *J. Chem. Eng. Data.* **1995**, *40* (4), 856-861.

(7) Ravindranath, P. A.; Forli, S.; Goodsell, D. S.; Olson, A. J.; Sanner, M. F. AutoDockFR: Advances in Protein-Ligand Docking with Explicitly Specified Binding Site Flexibility. *PLOS Comput. Biol.* **2015**, *11* (12), e1004586.

(8) Ravindranath, P. A.; Sanner, M. F. AutoSite: an automated approach for pseudo-ligands prediction—from ligand-binding sites identification to predicting key ligand atoms. *Bioinformatics* **2016**, *32* (20), 3142-3149.

(9) Zhang, Y.; Forli, S.; Omelchenko, A.; Sanner, M. F. AutoGridFR: Improvements on AutoDock Affinity Maps and Associated Software Tools. *J. Comput. Chem.* **2019**, *40* (32), 2882-2886.

(10) Trott, O.; Olson, A. J. AutoDock Vina: Improving the speed and accuracy of docking with a new scoring function, efficient optimization, and multithreading. *J. Comput. Chem.* **2010**, *31* (2), 455-461.

(11) Morris, G. M.; Huey, R.; Lindstrom, W.; Sanner, M. F.; Belew, R. K.; Goodsell, D. S.; Olson, A. J. AutoDock4 and AutoDockTools4: Automated docking with selective receptor flexibility. *J. Comput. Chem.* **2009**, *30* (16), 2785-2791.
